# Synthesis and Antiparasitic Activities of New Organometallic Derivatives of the Benzimidazole Anthelmintic Drugs

**DOI:** 10.1002/cbic.70490

**Published:** 2026-08-09

**Authors:** Corentin Ludwig, Aya C. Taki, Joseph J. Byrne, Pierre Mesdom, Olivier Blacque, Cécile Häberli, Trix Zumkehr, Gonzalo Scalese, Leticia Pérez‐Díaz, Dinorah Gambino, Britta Lundström‐Stadelmann, Jennifer Keiser, Robin B. Gasser, Kevin Cariou, Gilles Gasser

**Affiliations:** ^1^ Chimie ParisTech PSL University, CNRS, Institute of Chemistry for Life and Health Sciences, Laboratory for Inorganic Chemical Biology Paris France; ^2^ Department of Veterinary Biosciences Melbourne Veterinary School Faculty of Science The University of Melbourne Parkville Victoria Australia; ^3^ Department of Chemistry University of Zurich Zurich Switzerland; ^4^ Swiss Tropical and Public Health Institute Allschwil Switzerland; ^5^ University of Basel Basel Switzerland; ^6^ Institute of Parasitology Vetsuisse Faculty University of Bern Bern Switzerland; ^7^ Área Química Inorgánica Facultad de Química Universidad de la República Montevideo Uruguay; ^8^ Laboratory Redox Biology of Trypanosomes Institut Pasteur de Montevideo Montevideo Uruguay; ^9^ Sección Genómica Funcional Facultad de Ciencias Universidad de la República Montevideo Uruguay; ^10^ Multidisciplinary Center for Infectious Diseases University of Bern Bern Switzerland

**Keywords:** benzimidazole, bioorganometallic chemistry, leishmaniasis, metals in medicine, neglected tropical diseases (NTDs), parasitic helminths

## Abstract

About 50 years after their development, the benzimidazole anthelmintic drugs still occupy a key role in the treatment of parasitic diseases. Like other anti‐infective drugs, their extensive use has led to resistance development and now requires new solutions to treat these diseases. Organometallic derivatization of existing drugs has become a common strategy to design new drug candidates, which could benefit from enhanced activity against resistant or sensitive strains through the incorporation of an organometallic fragment. In this study, we describe the development of a new series of organometallic derivatives of benzimidazole anthelmintics. Ten derivatives, six of which were organometallic, were synthesized and characterized. In silico and in vitro studies were performed to determine their biological activity. Therefore, they were screened against a range of nematodes and other parasites. Biological activities were found for some of the derivatives against *Strongyloides ratti*, *Ancylostoma ceylanicum*, *Trichuris muris*, *Schistosoma mansoni*, *Echinococcus multilocularis,* or *Leishmania infantum* in similar ranges as the parent anthelmintics. Even if the activity of the benzimidazole scaffold was not systematically enhanced, this strategy is a good starting point for the development of new antiparasitic compounds and for further study of the mode of action of these drugs.

## Graphical abstract

Organometallic derivatization of existing drugs is a common strategy to look for new drug candidates. This work describes the derivatization of the benzimidazole anthelmintic scaffold, from the synthesis of the organometallic derivatives to the evaluation of their biological properties. It highlights a broad range of antiparasitic activities and leaves room for further study of this class of compounds.

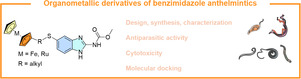

## 1 Introduction

Neglected tropical diseases (NTDs) cause substantial suffering worldwide in impoverished communities. Besides the obvious healthcare burden, these diseases have also deleterious social and economic consequences [1]. Since the 2010s, the World Health Organization (WHO) has encouraged members states and partners to contribute to plans (roadmaps) to prevent, control and eradicate NTDs [2, 3]. These roadmaps set milestones and aim to overcome NTDs on a global scale. The latest report that assesses the situation worldwide indicates slow progress and challenges that are faced to reach a global and ambitious solution to control these diseases [4]. There is no single and common solution to these issues, partly because of the diversity of pathogens that cause many of the 21 NTDs. For instance, leishmaniasis, Chagas disease, and sleeping sickness are caused by various trypanosomatid parasites, while schistosomiasis, echinococcosis, river blindness, and other helminthiasis are caused by diverse flatworms or roundworms. There is only a limited number of drugs that are used to treat these diseases, of which the benzimidazole class plays a crucial role [5, 6].

**FIGURE 1:**
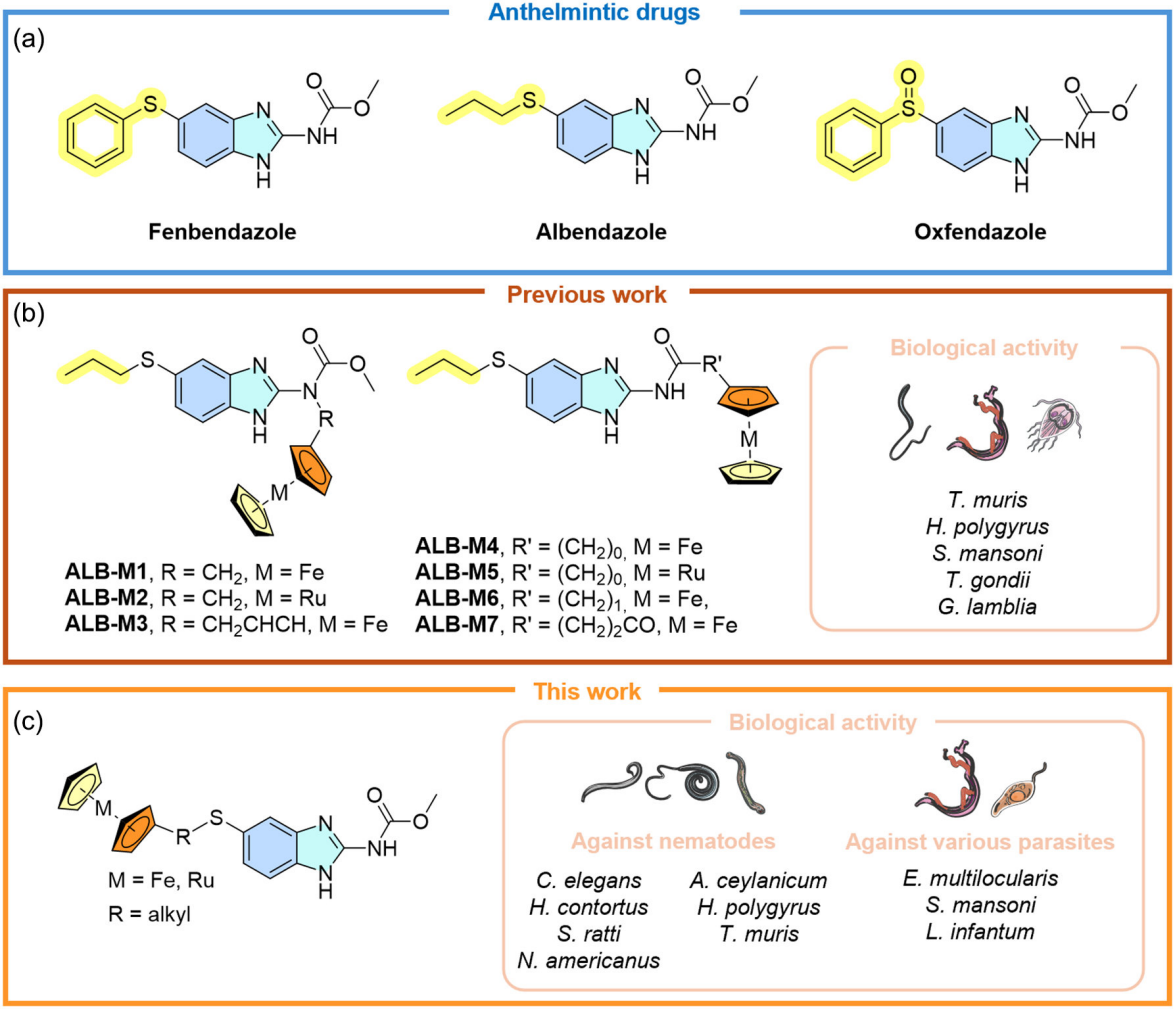
(a) Structures of the anthelmintic drugs fenbendazole, albendazole, and oxfendazole. (b) Structures of the organometallic derivatives of albendazole synthesized by our group in a previous work [9] by nucleophilic substitution and addition elimination. (c) Structure of the new organometallic derivatives studied in this work. Illustrations for the parasites from Servier Medical Art licensed under CC BY 4.0.

**FIGURE 2:**
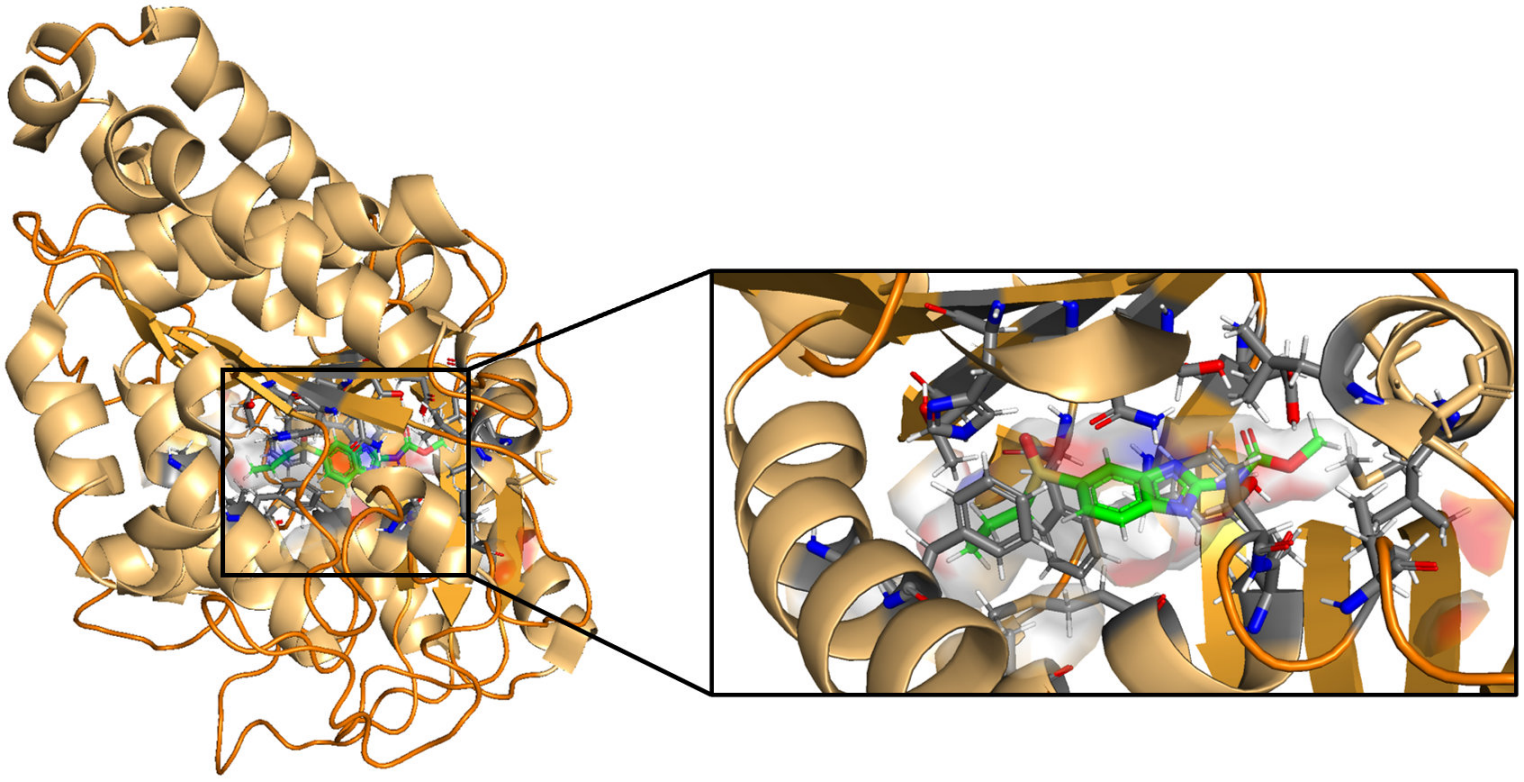
3D representation of *Haemonchus contortus* β‐tubulin isotype β12−16 homology model docked with albendazole sulfoxide by Robinson and coworkers (Model Archive ID: ma‐cso9w) [17]. The secondary structure of the protein is shown in orange. Carbon atoms of the residues in the binding pocket are colored gray, while the carbon atoms of albendazole sulfoxide are shown in green. Oxygen atoms are colored red, and nitrogen atoms are colored blue.

Benzimidazole anthelmintic drugs have been developed since the middle of the twentieth century [7, 8]. Three major benzimidazoles were developed during a short period of time (Figure 1a). While the anthelmintic activity of fenbendazole was discovered by Baeder et al. [10] in 1974, the activity of the corresponding sulfoxide, known as oxfendazole, was discovered 1 year later by Averkin et al. [11] The year after, Theodoride et al. [12] published their results about the anthelmintic activity of albendazole. Benzimidazole anthelmintic drugs inhibit the polymerization of the microtubules by binding selectively to the β‐tubulin of the parasite [7, 13, 16]. Even though this mode of action has been known for several decades, knowledge of the binding of these drugs benefited from more recent docking studies [17, 20]. These studies highlight the key role of the benzimidazole carbamate moiety in the formation of hydrogen bonds with the amino acids of the β‐tubulin. Indeed, the glutamate 198 of β‐tubulin would be determinant for the binding of the benzimidazole carbamates across all modeled species [17, 19, 20]. For instance, Robinson et al. built a homology model of *Haemonchus contortus* β‐tubulin isotype β12−16 (Model Archive ID: ma‐cso9w) [17], which highlights that the carbamate of albendazole sulfoxide is surrounded by polar residues that could be involved in hydrogen bonds like Ser165, Glu198, and Thr237 (Figure 2). On the opposite side of the binding pocket, the alkyl or aryl chains would fit close to lipophilic residues (Ile16, Phe20, Phe167, Phe200, and Val229). Such modeling studies are decisive for the understanding of the mechanisms involved in the resistance against benzimidazole drugs [18, 20], which often rely on mutations in β‐tubulin genes [7, 21].

Organometallic derivatization of existing drugs has become a common method which can be useful to overcome drawbacks faced by the organic analogs [22]. This strategy relies on the incorporation of an organometallic moiety into an identified organic scaffold. To that end, the use of a ferrocenyl moiety is a case in point. Metallocenes have found many applications in medicinal chemistry [23]. Among others, it was used by Biot et al. [24] for the synthesis of ferroquine, an organometallic derivative of the antimalarial drug chloroquine. Not only ferroquine relies on the classical mode of action of chloroquine, which involves the inhibition of hemozoin biomineralization, but also on the production of reactive oxygen species through a Fenton like mechanism [25, 26]. In the presence of hydrogen peroxide in the digestive vacuole of *Plasmodium falciparum*, reactive oxygen species were generated. This second mode of action enabled activity against chloroquine resistant strains and led ferroquine to phase II clinical trial [27]. This pioneering example drew attention to this strategy for the development of many other anti‐infective metallocenyl derivatives [28, 29], but so far no other anti‐infective compound reached the therapeutical potential of ferroquine. Not only the use of a metallocene could be interesting for the redox properties of the metal incorporated, but it could also increase the lipophilicity of the compound or even bring some three‐dimensionality in an existing drug scaffold [23].

Our group previously synthesized organometallic derivatives of albendazole, where the metallocene unit was incorporated by nucleophilic substitution on the benzimidazole carbamate (Figure 1b) or by addition–elimination on the aminobenzimidazole (Figure 1c) [9]. The series of compounds was tested against various helminth and protozoan species and a broad range of activities were found. Among them, **ALB‐M2** and **ALB‐M7** displayed 70% activity at 200 µM against *Trichuris muris* in vitro, which encouraged in vivo tests on *Trichuris muris* infected mice where moderate reduction of the worm burden was observed. Interestingly, **ALB‐M1** and **ALB‐M7** were found to be more active than albendazole against the protozoan *Toxoplasma gondii* in vitro. Hence, this first series of organometallic derivatives appeared as an interesting starting point for the further development of antiparasitic drug candidates.

Contrary to this first strategy (Figure 1b,c), we here chose to substitute the aryl or alkyl moiety of benzimidazole anthelmintics by a metallocene (Figure 1d). Comparison of the structure of fenbendazole, albendazole, and oxfendazole highlights two distinct parts: a common benzimidazole carbamate core, in gray, connected to a lipophilic chain by a sulfur atom, in yellow (Figure 1a). Metallocenes could ideally substitute this lipophilic chain as they are lipophilic organometallic fragments. Fine tuning of the lipophilicity could modulate permeation of the membranes, and the three‐dimensional lipophilic groups could better fill lipophilic pockets in the target. Hence, organometallic derivatization appeared as a promising strategy for the improvement of this class of drug.

Herein, we aimed at designing a synthesis route for new organometallic derivatives of benzimidazole anthelmintics, which resulted in a series of 10 benzimidazole derivatives. A computational study was performed on a β‐tubulin model to evaluate the influence of derivatization on the benzimidazole scaffold. Then, we assessed the biological activity of the compounds through screening against model nematodes, as well as other helminths and parasites.

Screening began with the evaluation of the compounds against *Caenorhabditis elegans*, which was chosen as a free‐living nematode model, and *Haemonchus contortus*, as a parasitic nematode model. This parasite is a highly pathogenic nematode of ruminants that feeds on blood in the gastrointestinal tract of its host and which is a serious threat to livestock farms [30, 31]. The screening was then extended to other parasitic nematodes that are relevant models for the study of soil‐transmitted helminthiases responsible for millions of infections worldwide [32]. *Strongyloides ratti*, a parasitic nematode of rodents commonly used as a laboratory model for *Strongyloides stercoralis* which can easily reproduce in rats and mice [33]; the hookworms *Heligmosomoides polygyrus* [34], *Necator americanus* [35], and *Ancylostoma ceylanicum* [36]; and the whipworm *Trichuris muris*, a parasite of rodents that is used as a model for the study of the parasite *Trichuris trichiura.* [32] Finally, other parasites were included in the screening such as *Echinococcus multilocularis*, a zoonotic fox tapeworm (cestode) that infects rodents (intermediate host) and humans (accidental host). This cestode is responsible for alveolar echinococcosis —a relatively rare but severe disease in humans, dogs and captive monkeys [37, 38]. The compounds were also screened against parasites responsible for schistosomiasis—a NTD that affects more than 50 million people annually [39]. Finally, compound activity was also tested against *Leishmania infantum*, one of the parasite species that is responsible for leishmaniasis—a parasitic disease with more than 350 million people at risk worldwide [40].

## 2 Results and Discussion

### 2.1 Synthesis and Characterization

**SCHEME 1:**
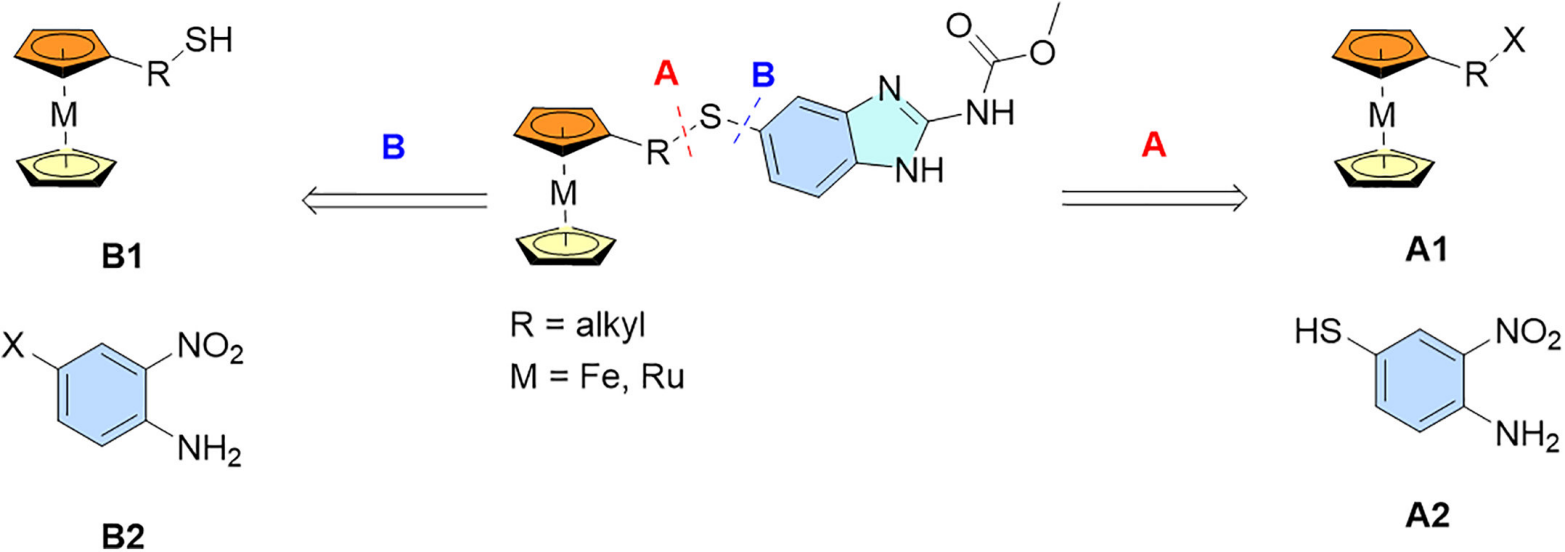
Retrosynthetic planning for the synthesis of organometallic benzimidazole derivatives.

Considering the position at which we want to incorporate organometallic moiety, two main synthetic strategies were identified for the synthesis of the organometallic benzimidazole derivatives (Scheme 1). The reported synthesis of fenbendazole or oxfendazole would support the nucleophilic aromatic substitution of various thiols **B1** on an aryl halide **B2**, but only a few examples of ferrocenylalkylthiols are found in the literature [41]. On the contrary, ferrocenyl building blocks bearing a leaving group (e.g., halide or ammonium salt) are easily available, and often used in the context of the derivatization of existing drugs [29]. Hence, we opted for the synthetic route A instead of the route B.

**SCHEME 2:**
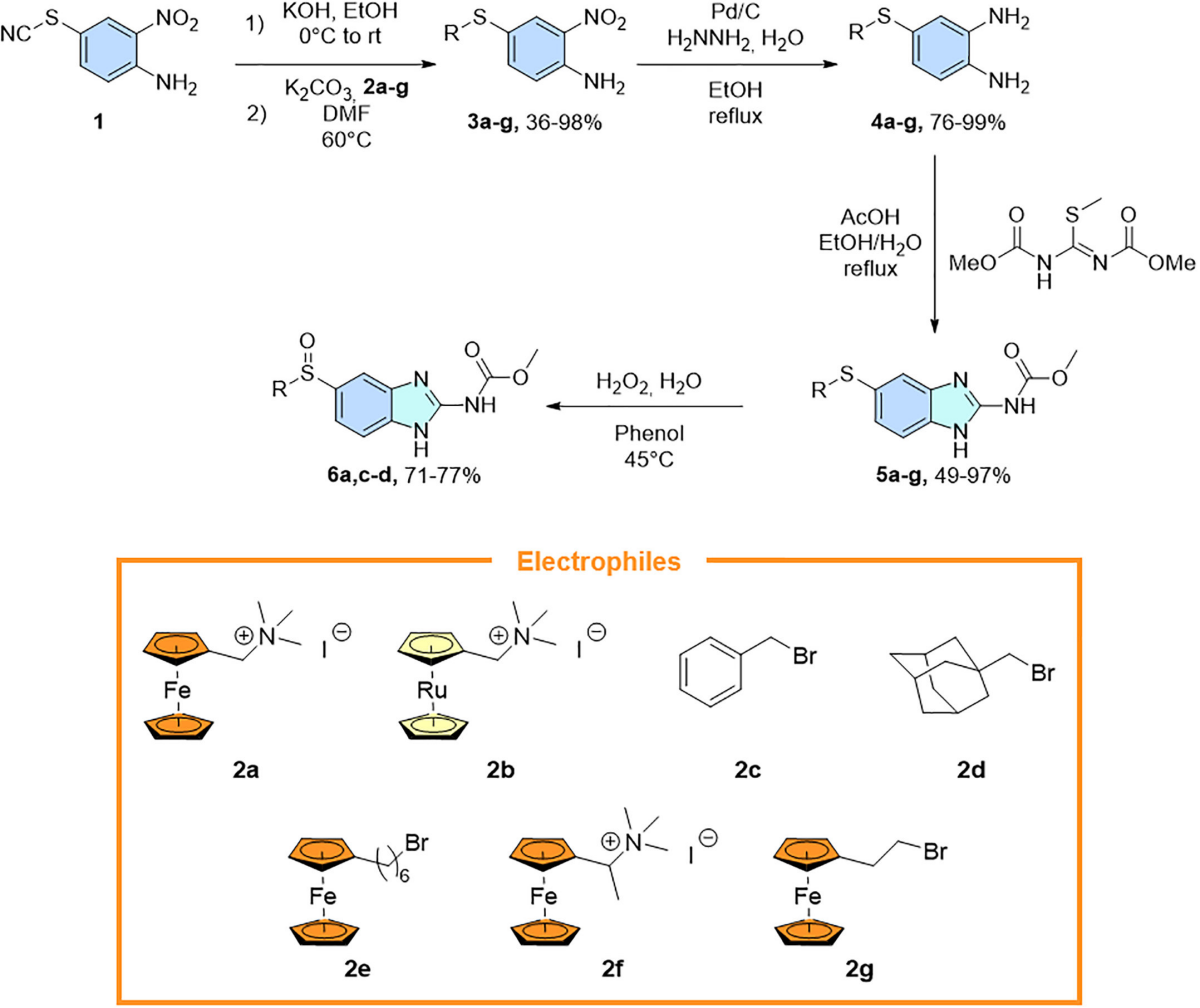
Synthesis of the series of benzimidazole derivatives.

**FIGURE 3:**
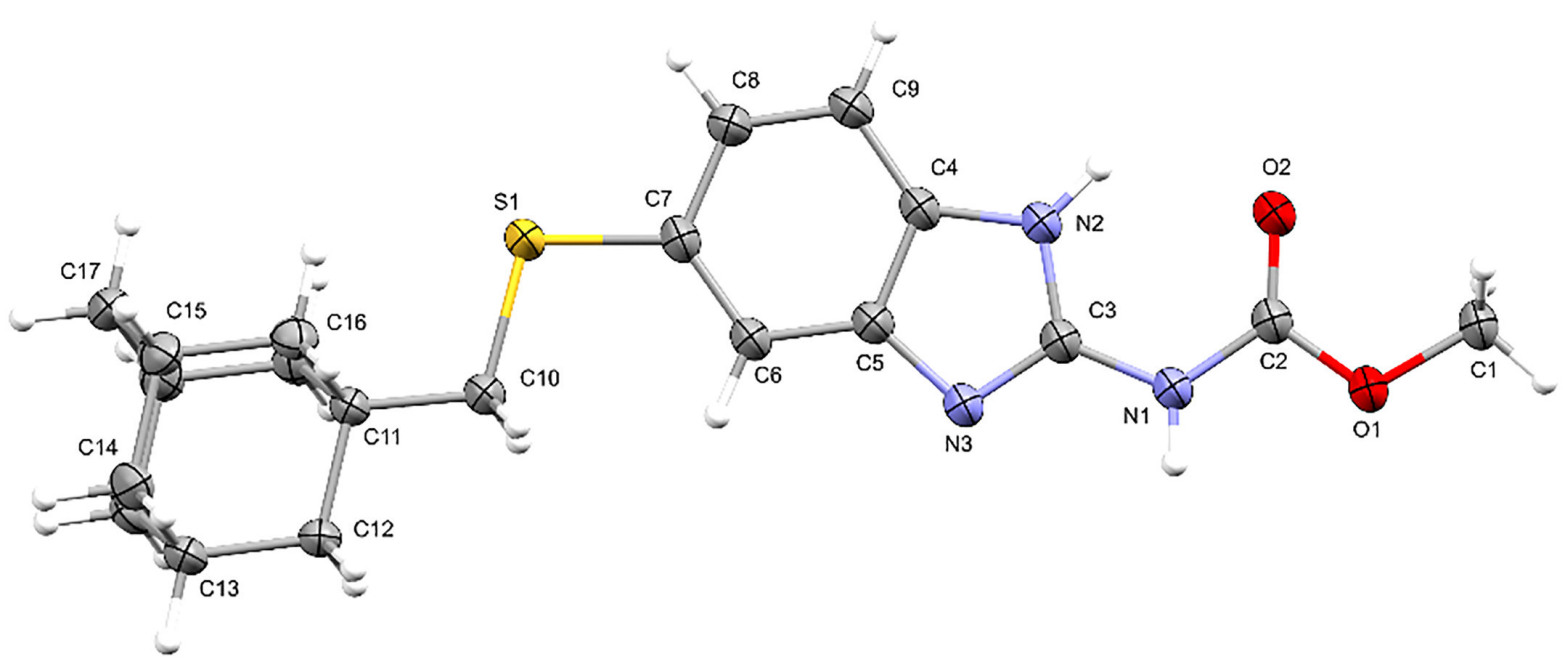
Molecular structure of compound **5d** (one of the two independent molecules). Displacement ellipsoids are drawn at the 50% probability level (CCDC: 2 564 735).

Synthesis of all the derivatives began with 2‐nitro‐4‐thiocyanatoaniline **1**, which was hydrolyzed to the corresponding thiol (Scheme 2) [42]. The freshly prepared thiol was extracted and directly used to perform the nucleophilic substitution on the electrophiles **2a–g** in the presence of K_2_CO_3_ in dimethylformamide (DMF) to obtain the sulfides in moderate to high yields (36%–98%). These electrophiles were chosen to perform a structure–activity relationship (SAR) study. The ferrocenyl building blocks **2a**,**e–g** were chosen to assess whether the length of the alkyl chain between the ferrocenyl group and the benzimidazole carbamate core would change the activity of the compounds. The ruthenocenyl derivative would enable evaluation of the influence of the metal center in the series with comparable sterics and lipophilicity but different redox properties [23]. Also, by taking advantage of bioisosterism relationships, the benzyl and methyl(adamantane) derivatives were chosen for comparison with organic compounds. Indeed, the ferrocenyl group is often used as a bioisostere of the phenyl group [23, 43], and the adamantyl moiety is a common three‐dimensional bioisostere of the phenyl ring [44, 45]. After incorporation of each building block of interest, reduction of the nitro compounds **3a–g** to the amines **4a–g** was performed with Pd/C and hydrazine monohydrate in high yields (76%–99%). Formation of the benzimidazole carbamate core was finally achieved in acidic medium with 1,3‐bis(methoxycarbonyl)‐2‐methyl‐2‐thiopseudourea as a guanidination reagent in moderate to high yield (49%–97%) [11]. Oxidation of the sulfides **5a**,**c**,**d** to the corresponding sulfoxides **6a**,**c**,**d** was performed using hydrogen peroxide (35% solution in water) in phenol [46]. Compounds **5a–g** and **6a**,**c**,**d** were characterized by ^1^H and ^13^C NMR, HR‐MS, and infrared spectroscopy. The molecular structure of **5d** (CCDC: 2 564 735) was confirmed by X‐ray diffraction after crystallization by vapor diffusion using chloroform as a solvent and acetonitrile as an antisolvent (Figure 3) [47]. The purity of each compound was assessed by elemental analysis prior to biological testing. The compounds were all stable in deuterated dimethylsulfoxide (DMSO) over 4 days at room temperature according to the ^1^H NMR spectra, which indicated that they were suitable for the preparation of stock solutions in this solvent.

### 2.2 In Silico Study With a Model of *Haemonchus contortus* β‐Tubulin

**FIGURE 4:**
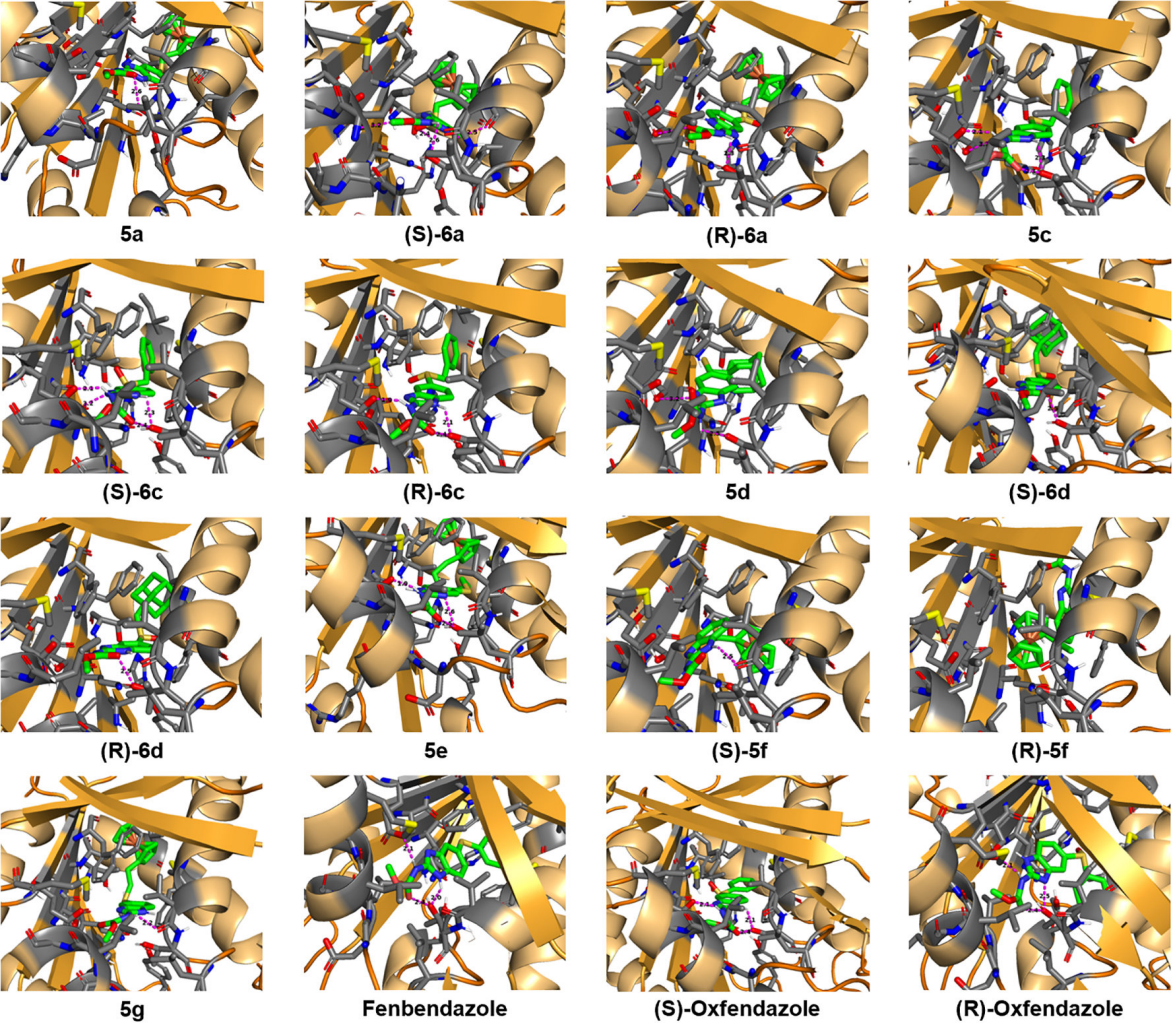
Close‐up views of the benzimidazole anthelmintics docked in the binding pocket of *Haemonchus contortus* β‐tubulin model. The secondary structure of the protein is shown in orange. Carbon atoms of the residues in the binding pocket are colored gray, while the carbon atoms of the benzimidazoles are shown in green. Oxygen atoms are colored red and nitrogen atoms are colored blue. Hydrogen bonds are highlighted with dashed magenta lines and distances are displayed in angstrom.

Computational studies through molecular docking are essential to gain knowledge on the interactions between small molecules and the targeted protein [48]. As mentioned previously, molecular docking was used in several studies to better understand the interaction of the benzimidazole anthelmintics with various models of β‐tubulin [17, 20]. Thus, we decided to evaluate how the structural modifications introduced in our series could affect the binding of the benzimidazole derivatives. To that end, we docked the benzimidazole carbamate studied in this article on a *Haemonchus contortus* β‐tubulin model obtained from Model Archive (Model Archive ID: ma‐cso9w) [17]. Therefore, compounds **5a**,**c–g**, **6a**,**c**,**d**, oxfendazole and fenbendazole were docked in the binding site of the protein with Autodock 4.2 (Figure 4) [49, 50]. While compounds **5f**, **6a**,**c**,**d** and oxfendazole were synthesized as racemic mixtures, both enantiomers were taken into account for the computational models. The ruthenocenyl derivative **5b** was not included in the study because Autodock 4.2 does not include parameters for the ruthenium atom.

The molecular docking protocol was first validated with the docking of albendazole sulfoxide, which is included in the model. It resulted in a binding energy of −9.43 kcal/mol and the root mean square deviation between the poses before and after docking is 1.49 Å. The binding pose selected for each compound was the one with the lowest binding energy (Table 1). All docking results suggest favorable interactions between the model of β‐tubulin and the ligands since all binding energies are negative, but differences are observed depending on the lipophilic chain of the derivatives. Predicted binding energies of oxfendazole and fenbendazole are in the same range and they could make hydrogen bonds with the residues Glu198 and Thr237 in the binding pocket, which was already highlighted in previous docking studies [17, 20]. Similarly, their homologs **5c** and **6c** are also predicted to bind to the protein with comparable binding energies and they would also be able to make hydrogen bonds with these residues, which underlined that the addition of a methylene group would not alter the binding mode of the benzimidazole in the pocket. However, derivatives with three‐dimensional groups displayed higher binding energies, with differences in the range of 5 kcal/‍mol. Adamantyl and ferrocenyl derivatives could fit within the binding pocket of the protein and these fragments are positioned near the lipophilic residues Ile16, Phe20, and Phe167, with the exception of compound **(R)‐5f**. Despite a better filling of the lipophilic part of the binding site, the three‐dimensional groups would force a larger distance between the carbamate and the residue Glu198. Hence, the carbamate would be too far to maintain hydrogen bonds with this residue and could only interact with Thr237. Other hydrogen bonds could also be expected for some of the derivatives with three‐dimensional groups, for instance with Ser165 (**5d**, **6a**) or Val236 (**(S)‐5f**, **5g**), but they would not enable to reach similar binding energies as oxfendazole, fenbendazole or compounds **5c** and **6c**. Interaction with Glu198 would only be maintained for **5e**, as the hexyl chain could be highly folded to better fill the pocket, but it still displays higher binding energies than the parent compounds. This computational study did not highlight any significant difference between the binding energies and poses of the sulfides and the corresponding sulfoxides, nor of the enantiomers of each sulfoxide.

**TABLE 1:** Summary of the binding energies and key interactions for the compounds docked in the binding pocket of *Haemonchus contortus* β‐tubulin model.

Compound	Binding energy, kcal/mol	Key interactions
**(R)‐Oxfendazole**	−8.98	Glu198, Thr237
**(S)‐Oxfendazole**	−10.06	Glu198, Thr237
**Fenbendazole**	−9.83	Glu198, Thr237
**5a**	−5.97	Thr237
**(R)‐6a**	−6.36	Thr237, Ser165
**(S)‐6a**	−6.62	Thr237, Ser165
**5c**	−10.44	Glu198, Thr237, Ser165
**(R)‐6c**	−10.92	Glu198, Thr237
**(S)‐6c**	−10.46	Glu198, Thr237
**5d**	−6.35	Ser165
**(R)‐6d**	−4.86	Thr237
**(S)‐6d**	−6.52	Thr237
**5e**	−5.52	Glu198, Thr237
**(R)‐5f**	−5.44	—
**(S)‐5f**	−6.40	Val236
**5g**	−6.47	Val236

This in silico study provides knowledge on the possible binding poses of the benzimidazole derivatives in this *H. contortus* β‐tubulin model. All the derivatives could fit in the binding pocket of the enzyme and unlike fenbendazole, oxfendazole or the homologs **5c** and **6c**, the three‐dimensional groups better fill the lipophilic area of the binding pocket, but a key hydrogen bond between the protein and the benzimidazole moiety could also be disrupted, which would be associated with an increase in binding energy and would likely be weaker inhibitors. This model provides a basis for the understanding of the binding of the compounds to a given isotype *H. contortus* β‐tubulin model and provides insights on how the structural modifications could modify the activity of the benzimidazole derivatives. Since abundance and expression of β‐tubulin isotypes vary across species, further docking studies on other models would be required to better understand the differences observed from one species to another.

### 2.3 In Vitro Screening of the Benzimidazole Derivatives on *Caenorhabditis elegans* L4s and *Haemonchus contortus* xL3s

First, we assessed the activity of the compounds against model nematodes (Table 2). The activity of compounds was measured as a reduction in motility and/or development in established assays, with statistical significance calculated as described in previous articles [51, 53]. Besides the synthesized organometallic and organic derivatives **5a–g** and **6a,c,d**, fenbendazole and oxfendazole were also tested to compare the activity of the derivatives with that of their respective parent drugs. Monepantel and moxidectin were used as positive controls. In vitro screening of the benzimidazole derivatives was performed against *Caenorhabditis elegans* (fourth‐stage larvae, L4s) [51], which is a relevant laboratory model for the study of the effect of anthelmintic drugs on nematodes even though it is not parasitic [54]. Compound **5g** and fenbendazole were the most active compounds against *C. elegans* L4s with relative IC_50_ reaching 2.3 and 1.4 µM, respectively, while compounds **5a** and **5c** also showed significant motility reduction. The ferrocenyl derivative reached a similar range of activity as the commercial benzimidazole anthelmintic against *C. elegans*. Homologation barely changed the activity of the phenyl or ferrocenyl derivatives. The organometallic compounds **6a**, **5b**, **5f** and oxfendazole showed a milder motility reduction against *C. elegans*. Overall, these biological assays showed that no benefit was obtained from the organometallic derivatization against *C. elegans*. Then, *Haemonchus contortus* (exsheathed third‐stage larvae, xL3s) was chosen as parasitic nematode model [52, 53], as β‐tubulin polymerization is known to be inhibited by benzimidazole anthelmintics [15]. The relevance of this model is reinforced by the widespread resistance of *H. contortus* to benzimidazole anthelmintic drugs due to extensive use [31]. Unfortunately, no compound showed a significant motility reduction in *H. contortus* xL3s, which is consistent with previous studies that included albendazole sulfoxide [55], although compounds **5c** and fenbendazole markedly inhibited larval development and induced curved and coiled phenotypes. Especially, the organometallic derivatives failed to induce nematocidal effect.

**TABLE 2:** Activity of organometallic benzimidazole derivatives against *Caenorhabditis elegans* L4s and *Haemonchus contortus* xL3. Effect in *Caenorhabditis elegans* after 40 h and in *Haemonchus contortus* after 168 h. Data represent the mean values and standard deviations of three individual assays, each using biological triplicates.

Compounds	*Caenorhabditis elegans* L4s		*Haemonchus contortus* xL3s			
	**IC**_**50**_ **L4 motility inhibition**, ‍**µM (SD)**	**MMI;** a **L4 motility inhibition, % (SD)**	**IC**_**50**_ **xL3 motility inhibition, µM (SD)**	**MMI;** a **xL3 motility inhibition, % (SD)**	**MIC;** b **larval development inhibition, µM (SD)**	**Abnormal phenotype,** **50 µM,** ** % ±SD**
**5a**	8.9 (6.8)	65.9 (14.8)	>50	14.3 (8.4)	>50	—
**6a**	~50	53.6 (14.4)	>50	11.2 (9.3)	>50	—
**5b**	9.1 (3.6)	54.4 (14.8)	>50	12.7 (3.0)	>50	—
**5c**	14.6 (3.2)	72.7 (9.7)	>50	22.6 (14.4)	1.9 (0.2)	*Cur*,^c^ *Coi* d (72 ± 3)
**6c**	>50	9.5 (20.2)	>50	19.3 (9.0)	50 (0.0)	—
**5d**	>50	30.8 (7.2)	>50	0.0 (15.6)	>50	—
**6d**	>50	0.0 (13.7)	>50	0.0 (6.4)	>50	—
**5e**	>50	33.8 (25.5)	>50	17.8 (6.7)	>50	—
**5f**	4.5 (2.2)	47.7 (14.9)	>50	5.4 (15.2)	>50	—
**5g**	2.3 (0.9)	59.2 (8.7)	>50	0.4 (17.4)	>50	—
**Oxfendazole**	25.2 (21.5)	40.8 (8.1)	>50	25.0 (13.9)	33.3 (4.2)	—
**Fenbendazole**	1.4 (0.5)	59.1 (16.5)	>50	5.0 (12.6)	1.5 (0.2)	*Coi* d (75 ± 1.7)
**Monepantel**	0.1 (0.03)	66.2 (21.2)	0.3 (0.06)	83.1 (6.8)	0.3 (0.09)	*Coi* d (95 ± 0)
**Moxidectin**	0.1 (0.05)	99.8 (0.4)	17.2 (11.2)	79.9 (7.2)	>50	—

a MMI: maximum motility inhibition (the highest inhibition level observed) at the highest concentration tested (50 µM).

b MIC: minimum inhibitory concentration (100% inhibition observed).

c *Cur*: Curved phenotype.

d *Coi*: Coiled phenotype.

Poor correlation between the in silico model and the in vitro assays was observed because of the low activity of the compounds. Compounds **5c**, **6c**, oxfendazole, and fenbendazole were found to have the lowest binding energies against *H. contortus* β‐tubulin model and these compounds also exhibited low activity against larval development. While the model did not predict any major difference between the sulfides and the sulfoxides, the minimum inhibitory concentrations increased by 20 folds with this functional group modification. Consistently with the computational study, the derivatives with three‐dimensional lipophilic chains (adamantyl, metallocenyl) were less potent inhibitors than the organic phenyl derivatives.

### 2.4 Screening Against Other Nematodes and *Schistosoma mansoni*

Following the results against *C. elegans* L4s and *H. contortus* xL3s, the screening was extended to other nematodes and *Schistosoma mansoni* (Table 3). Previous screening of the commercially available benzimidazole anthelmintics, among which fenbendazole and oxfendazole, against *Heligmosomoides polygyrus*, *Strongyloides ratti*, *Necator americanus*, *Ancylostoma ceylanicum*, and *Trichuris muris* showed that neither of them was strongly active in vitro against the larval or adult stages of these parasites [56]. Similarly to the results obtained on these nematodes, previous studies showed that *Schistosoma* spp. are not susceptible to benzimidazole anthelmintics [57, 58]. In order to know whether there are benefits from organometallic derivatization of this class of drugs, the activity of the derivatives was assessed against the above‐mentioned parasites by the evaluation of their viabilities after treatment. The effect of the compounds was measured at the same concentration for all the nematodes (10 µM). Organometallic derivatization did not improve the nematocidal effects of the benzimidazole scaffold. In the best cases, organometallic derivatives retained the activity of the parent organic drug fenbendazole. Indeed, the ferrocenyl derivatives **6a** and **5e** showed moderate activity against *S. ratti*, in the same range as fenbendazole. Similarly, the ferrocenyl derivatives **5e** and **5g** displayed mild activity against *A. ceylanicum* L3s, in the same range as fenbendazole and its homolog **5c**. The whole series of compounds and the commercially available oxfendazole and fenbendazole displayed moderate activity against *T. muris*. On the contrary, no significant activity was observed against *H. polygyrus* L3s or *N. americanus* L3s.

**TABLE 3:** Screening of organometallic benzimidazoles derivatives on various parasitic nematodes and *Schistosoma mansoni*. Effect in % after 72 h, all treatment wells were normalized to untreated controls. Compounds were tested in technical triplicates in two independent experiments for NTS, *Heligmosomoides polygyrus* L3s, *Necator americanus* L3s, *Anylostoma ceylanicum* L3s, and *Strongyloides ratti* L3s. Compounds were tested in technical duplicates in two independent experiments for *Schistosoma mansoni* adult and *Trichuris muris*.

Compounds	NTS	NTS	***Schistosoma mansoni*** **adult**	***Schistosomamansoni*** **adult**	***H*** ***eligmosomoides*** ***polygyrus*** **L3s**	***S*** ***trongyloides*** ***ratti*** **L3s**	*N**ecator** americanus* **L3s**	*A**ncylostoma** ceylanicum* **L3s**	***Trichuris muris*** **adult**
	Effect in % (SD), 10 µM	**Effect in % (SD), 1** **µM**	**Effect in % (SD),10 µM**	**Effect in % (SD),1 µM**	**Effect in % (SD), 10 µM**	**Effect in % (SD),** ** 10 µM**	**Effect in % (SD), 10 µM**	**Effect in % (SD), 10 µM**	**Effect in % (SD), 10 µM**
**5a**	100.0 (0.0)	45.83 (0.0)	81.25 (6.3)	28.15 (3.1)	13.7 (5.4)	0.0 (0.0)	0.0 (0.0)	27.1 (1.0)	40.0 (0.0)
**6a**	52.08 (6.3)	—	—	—	5.5 (0.4)	42.5 (2.2)	0.0 (0.0)	1.1 (4.9)	45.0 (5.0)
**5b**	100.0 (0.0)	37.5 (4.2)	75.0 (0.0)	28.15 (3.1)	9.0 (4.6)	0.0 (0.0)	0.0 (0.0)	22.2 (3.9)	42.5 (2.5)
**5c**	37.5 (0.0)	—	—	—	1.5 (0.3)	16.2 (1.6)	9.4 (8.7)	54.6 (2.2)	50. (0.0)
**6c**	37.5 (0.0)	—	—	—	7.4 (1.6)	9.4 (1.2)	0.0 (0.0)	22.3 (2.3)	40.0 (0.0)
**5d**	39.58 (2.1)	—	—	—	5.3 (1.8)	25.2 (1.3)	0.0 (0.0)	1.8 (3.7)	32.5 (2.5)
**6d**	39.58 (2.1)	—	—	—	11.9 (2.2)	0.0 (0.0)	0.0 (0.0)	15.2 (2.1)	45.0 (0.0)
**5e**	39.58 (2.1)	—	—	—	5.0 (0.8)	46.8 (8.4)	2.1 (3.0)	40.3 (0.2)	45.0 (0.0)
**5f**	79.17 (4.2)	43.75 (2.1)	59.4 (3.1)	—	8.6 (6.1)	15.5 (2.6)	0.0 (0.0)	22.6 (0.7)	45.0 (5.0)
**5g**	47.92 (2.1)	—	—	—	7.2 (0.2)	25.7 (7.8)	15.4 (5.9)	42.1 (4.7)	35.0 (0.0)
**Oxfendazole**	39.58 (2.1)	—	—	—	5.4 (2.4)	18.2 (0.1)	18.5 (0.3)	27.3 (6.5)	45.0 (0.0)
**Fenbendazole**	60.42 (2.1)	—	—	—	4.9 (0.6)	42.0 (6.7)	2.7 (1.9)	44.4 (0.8)	50.25 (2.5)

In addition to these parasitic nematod‍es, the series was tested against *S. ‍mansoni*. The organometallic sulfides **5a**, **5b,** and **5f** were active against newly transformed schistosomula (NTS) and adult *S. mansoni* displaying 81.25%, 75%, and 59.4% activities against the latter stage, respectively after 72 h at 10 µM. These three organometallic compounds were still mildly active against NTS and *S. mansoni* at 1 µM. Even if these three organometallic derivatives displayed enhanced activities compared to the benzimidazole drugs, no evidence was found for a metal‐based mode of action, which would be responsible of the activity against *Schistosoma* spp.

### 2.5 Screening Against *Echinococcus multilocularis*

Following the results obtained on nematodes and trematodes, we decided to determine their activity on *Echinococcus multilocularis*, a parasitic cestode responsible for alveolar echinococcosis. Patients diagnosed with alveolar echinococcosis usually undergo long treatments with the benzimidazole anthelmintic drug albendazole that is not curative for the disease [59]. Other compounds of this family of drugs also displayed in vitro or in vivo activity against *E. multilocularis*, making it a relevant starting point for the development of new anthelmintic drugs [60, 61]. The parasiticidal activity of the compounds was evaluated using germinal layer cell toxicity assay [62]. None of the compounds were active against *E. multilocularis* germinal layer cells, except for oxfendazole at high concentrations (Table 4).

**TABLE 4:** Screening of organometallic benzimidazoles derivatives on *Echinococcus multilocularis* germinal layer cells. Each compound was incubated with 15 AU of germinal layer cells. Mean effects relative to the DMSO control (100%) after 5 days of drug incubation are given including SDs of four technical replica (technical duplicates for the controls oxfendazole and fenbendazole).

Compound	Relative germinal layer cell viability (SD), 50 µM	Relative germinal layer cell viability (SD), 20 µM	Relative germinal layer cell viability (SD), 10 µM	Relative germinal layer cell viability (SD), 1 µM	Relative germinal layer cell viability (SD), 0.1 µM
**5a**	195.75 (44.5)	179 (11.6)	170.75 (47.16)	—	—
**6a**	162.5 (17.79)	211.25 (51.35)	168.5 (43.55)	—	—
**5b**	274 (59.36)	207 (43.81)	169.5 (32.79)	—	—
**5c**	150.75 (10.87)	117.25 (12.97)	116.25 (82.64)	—	—
**6c**	187.25 (41.52)	188.75 (30.54)	109.5 (58.13)	—	—
**5d**	181 (46.28)	155.75 (13.79)	152 (53.6)	—	—
**6d**	151.75(22.5)	133.25 (32.6)	166.5 (36.67)	—	—
**5e**	213 (48.9)	223.75 (58.73)	170.5 (30.69)	—	—
**5f**	204.5 (28.62)	187.5 (52.67)	164.5 (21.0)	—	—
**5g**	167.75 (19.12)	166.25 (19.84)	175.5 (43.45)	—	—
**Oxfendazole**	—	—	11 (15.56)	179.5 (125.16)	82.5 (38.89)
**Fenbendazole**	—	—	100 (4.24)	77 (1.41)	87 (9.9)

Further testing of the compounds against *E. multilocularis* metacestode vesicles by damage‐marker release assay (phosphoglucose‐isomerase assay) [63] showed activity of oxfendazole and the organometallic sulfoxide **6a** (>20%) after 12 days of incubation (Table 5). Consequently, while the organometallic modifications did not yield the anticipated boost in potency against *E. multilocularis*, these findings provide valuable insights into the structural requirements needed to target this resilient parasite.

**TABLE 5:** Screening of organometallic benzimidazoles derivatives on *Echinococcus multilocularis* metacestode vesicles. Each compound and concentration were tested on 20–30 metacestode vesicles. Mean effects of the Triton x‐100 control (100%) after 5 and 12 days of drug incubation are given including SDs of two independent biological replica, each assessed in technical triplicates.

Compounds	PGI release in % (SD), 50 µM, 5d incubation	PGI release in % (SD), 20 µM, 5d incubation	PGI release in % (SD), 10 µM, 5d incubation	PGI release in % (SD), 50 µM, 12d incubation	PGI release in % (SD), 20 µM, 12d incubation	PGI release in % (SD), 10 µM, 12d incubation
**5a**	−1.60 (1.98)	−1.61 (0.00)	−2.08 (0.72)	7.53 (4.91)	11.37 (11.21)	14.05 (5.42)
**6a**	−0.55 (3.44)	6.02 (4.83)	−1.93 (0.87)	6.41 (1.15)	57.13 (14.56)	2.69 (0.46)
**5b**	1.40 (2.15)	−0.76 (1.98)	−1.09 (1.11)	8.46 (6.20)	4.40 (1.11)	3.71 (1.50)
**5c**	−1.67 (0.47)	−0.47 (0.98)	−0.87 (2.22)	2.98 (0.46)	2.74 (1.10)	6.70 (7.59)
**6c**	−1.22 (0.32)	−2.14 (2.20)	−2.42 (0.02)	15.57 (12.49)	6.60 (7.47)	1.89 (1.35)
**5d**	−2.20 (0.43)	−1.95 (1.66)	−2.21 (1.44)	1.85 (1.81)	1.54 (2.69)	1.80 (1.72)
**6d**	−0.94 (1.52)	−1.25 (2.31)	−0.64 (1.14)	1.80 (0.01)	1.47 (0.41)	0.60 (2.00)
**5e**	−0.78 (1.15)	−1.55 (0.12)	−1.71 (0.73)	2.51 (0.17)	2.18 (1.72)	3.07 (1.69)
**5f**	1.81 (1.40)	−1.48 (0.07)	−1.18 (1.38)	14.33 (9.01)	1.36 (0.59)	1.53 (1.47)
**5g**	−1.47 (0.47)	−0.81 (1.52)	0.52 (1.06)	1.64 (0.84)	4.12 (2.66)	6.24 (0.74)

	**PGI release in % (SD), 10 µM, 5d incubation**	**PGI release in % (SD), 1 µM, 5d incubation**	**PGI release in % (SD), 0.1 µM, 5d incubation**	**PGI release in % (SD), 10 µM, 12d incubation**	**PGI release in % (SD), 1 µM, 12d incubation**	**PGI release in % (SD), 0.1 µM, 12d incubation**
**Oxfendazole**	2.15 (0.00)	0.28 (0.88)	−0.54 (1.41)	39.88 (3.12)	9.11 (7.14)	3.25 (1.91)
**Fenbendazole**	−1.02 (0.31)	−1.64 (0.12)	−1.68 (0.13)	17.47 (17.62)	8.01 (5.18)	10.08 (0.50)

### 2.6 Screening Against *Leishmania*

Benzimidazole anthelmintic drugs were already tested for their activities against *Leishmania species*, but failed to induce leishmanicidal effect [64, 65]. The antileishmanial potential of the synthesized derivatives (**5a–g**, **6a,c,d**), alongside the parent drugs fenbendazole and oxfendazole, was initially evaluated against *L. infantum* promastigotes using a resazurin‐based viability assay [66, 68]. An initial screening was conducted at a fixed concentration of 5 µM, which represents a stringent threshold for identifying promising hits in early‐stage drug discovery. At this concentration, no significant leishmanicidal effect was observed, with parasite viability remaining comparable to that of the negative controls (Table 6). To further probe the activity, the concentration was increased to 50 µM. Under these conditions, the sulfoxide **6d** exhibited a potent leishmanicidal effect, reducing parasite viability to baseline levels. In contrast, the sulfide **5f** showed moderate activity, with viability reaching approximately 50% (Table 6). The remaining derivatives failed to induce significant mortality, even at this higher concentration.

**TABLE 6:** Screening of organometallic benzimidazole derivatives against *L. infantum* promastigotes and their cytotoxicity against murine macrophages J774. Data represent the mean values and standard deviations of three independent biological replicates.

Compounds	*Leishmania infantum* promastigotes		Murine macrophages J774
	% parasites viability (SD)		% cell viability (SD)
	5 µM	50 µM	50 µM
**5a**	85.0 (1.4)	99.5 (5.9)	—
**6a**	88.2 (7.7)	96.1 (6.6)	—
**5b**	84.5 (6.1)	78.7 (5.0)	—
**5c**	87.6 (1.9)	70.2 (7.6)	—
**6c**	108.0 (11.7)	93.2 (2.3)	—
**5d**	98.0 (22.1)	81.3 (6.5)	—
**6d**	116.5 (14.7)	5.5 (1.3)	4.0 (0.5)
**5e**	103.9 (9.1)	101.6 (10.1)	—
**5f**	97.6 (5.3)	60.4 (11.2)	1.0 (0.2)
**5g**	82.4 (6.8)	73.6 (7.3)	—
**Oxfendazole**	95.4 (8.2)	73.6 (7.3)	—
**Fenbendazole**	104.7 (19.5)	84.9 (6.8)	—

To determine whether these compounds might exert a leishmanostatic effect, arresting replication without inducing immediate cell death, growth kinetics were monitored over a 5‐day period. The growth curves for all inactive compounds were indistinguishable from those of the negative controls (Figure S129), confirming that, with the exceptions of **6d** and **5f**, this series possesses neither leishmanicidal nor leishmanostatic activity against *L. infantum* promastigotes under the conditions tested.

Given that *Leishmania* parasites invade nucleated host cells, the cytotoxicity of the active compounds (**6d** and **5f**) was assessed against the J774 murine macrophage cell line. High selectivity is a critical prerequisite for hit‐to‐lead progression, with a selectivity index (SI) of at least 10 typically required for kinetoplastid infections [69]. Both compounds were found to be toxic to the host cells at 50 µM (Table 6). Consequently, the antileishmanial activity observed at this concentration is likely attributable to nonspecific cytotoxicity rather than a targeted antiparasitic mechanism. Due to this poor selectivity and inherent host‐cell toxicity, further evaluation in an intracellular amastigote infection model was deemed unwarranted, as the compounds do not meet the safety criteria necessary for effective intracellular parasite clearance. While these results indicate that derivatization with adamantane and ferrocene moieties can impart some activity against *Leishmania* promastigotes, further structural optimization is required to enhance the safety profile and selectivity of this benzimidazole‐based series.

### 2.7 In Vitro Cytotoxicity Against Healthy Human Cells

The cytotoxicity of the benzimidazole derivatives was evaluated on noncancerous retinal pigment epithelium cells (RPE‐1) to compare their toxicity toward parasites with their toxicity toward healthy human cells. Half‐maximal inhibitory concentration (IC_50_) values were determined by fluorimetric cell viability assay using resazurin as an indicator of the cell viability (Table 7). All sulfoxide derivatives displayed higher IC_50_ values than the corresponding sulfides, like the controls oxfendazole and fenbendazole, associated with a roughly 10‐fold decrease in IC_50_ values. In both cases, the IC_50_ values of the organometallic compounds remain in the same range as those of parent compounds, namely fenbendazole and oxfendazole. In addition, the benzimidazole derivatives displayed cytotoxicity values in the micromolar range, at which moderate antiparasitic activities were found. Indeed, IC_50_ values were found to be close to 10 µM for the sulfides **5a–d,f,g**. Hence, these cytotoxicity results would qualify the antiparasitic potential of the compounds. For instance, the antischistosomal activities of **5a**, **5b,** and **5f** were found down to 1 µM, while they exhibited cytotoxicity values of about 5 µM. A similar conclusion can be reached based on the comparison of the antileishmanial activities and cytotoxicity profiles of compounds **5f** and **6d**. Therefore, these results could be related to a lack of selectivity between the host's cells and the parasites.

**TABLE 7:** IC_50_ values of the benzimidazole derivatives against retinal pigment epithelium cells (RPE‐1). Mean values are given, including SDs from three independent biological replicates, each in technical triplicate.

**C** **ompound**	**IC** **_50_** **,** **µM (SD)**
**5a**	5.3 (0.5)
**6a**	62.5 (20.5)
**5b**	3.3 (0.3)
**5c**	15.7 (2.8)
**6c**	>100
**5d**	10.3 (0.5)
**6d**	94.9 (9.1)
**5e**	34.1 (1.0)
**5f**	6.9 (0.8)
**5g**	1.2 (0.1)
**Oxfendazole**	>100
**Fenbendazole**	5.7 (0.8)
**Cisplatin**	>100

### 2.8 Structure–Activity Relationship Study

**FIGURE 5:**
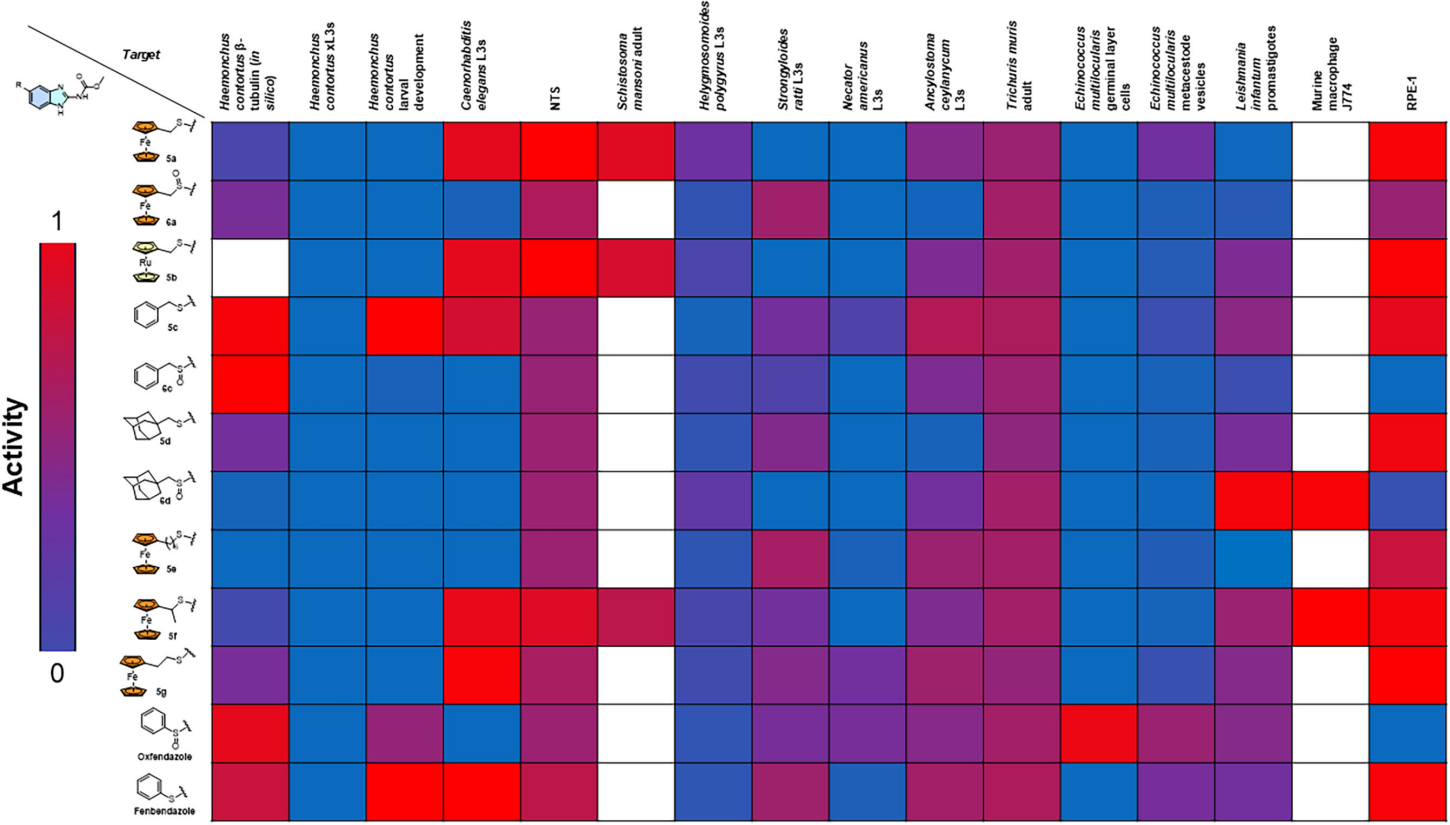
Heatmap representation of the structure–activity relationship for the benzimidazole derivatives. Normalized activities are represented on a color gradient from, not active (0, blue) to active against parasites or toxic against mammalian cells (1, red). All effects were normalized with respect to the column or used as such for percentages. White cells represent not determined activities or toxicities.

Thanks to the structural diversity of the compounds investigated in this work, we expected to gain insights into the influence of the organometallic moieties, the length of the alkyl chain linker, the oxidation of the sulfide, and both the two‐ and three‐dimensional geometries on the biological activity of the benzimidazole derivatives. To that extent, activities were normalized and represented as a heatmap for comparison of the activities of the benzimidazole derivatives [70], which revealed only few SAR trends for some of the targeted species (Figure 5).

The parent benzimidazole drug fenbendazole exhibited one of the most favorable activity profiles across the panel of parasites studied in this work. Homologation of a single carbon to compound **5c** would not change much its activity. Organometallic derivatization with the replacement of the phenyl ring of compound **5c** by the ferrocenyl group of compound **5a** did not systematically improve the activity of the benzimidazole derivatives. There were only a few targets against which the activity was improved, such as *Schistosoma* spp., and others against which the activity decreased such as *S. ratti* L3s or *L. infantum* promastigotes. Similarities between the activities of the ferrocenyl derivative **5a** and the ruthenocenyl derivative **5b** were found. Contrary to ferroquine, for which differences in activity were observed between the ruthenocenyl and ferrocenyl analogs [26], this lack of difference would indicate that no redox activity is involved in the mode of action of these metallocenyl benzimidazole derivatives. The metallocenes could also have been used as three‐dimensional groups, which could better fill the active site of a target than two‐dimensional phenyl rings. However, the adamantyl derivative **5d** was poorly active and there were several differences in activity compared to the metallocenyl derivatives. If similarities with metallocenes were noticed, this compound could have provided clues on the role of the three‐dimensional structure of the metallocenes. Finally, compound **5g** and compound **5f** showed that homologation and ramification of the ferrocenyl derivatives, would not change the activity of the compounds, contrary to longer homologation (**5e**), which decreased significantly the overall activity. More differences were observed when switching from sulfides to sulfoxides. For example, the sulfide **5a** is more active than the sulfoxide **6a** against *C. elegans* L3s or NTS, but also more cytotoxic against RPE‐1 cells. Analogous relationships were observed with the sulfide **5c** and the sulfoxide **6c** or fenbendazole and oxfendazole. An exception was found with the sulfoxide **6d**, which was the most active compound against * L. infantum* promastigotes.

In general, only few conclusions can be drawn from the SAR study, and no general trend was found across the organisms against which the benzimidazole derivatives were tested. The activity is highly dependent on targeted parasites and varies with both organic and organometallic modifications. Importantly, there are only few examples where the organometallic derivatization improved the activity among the series of derivatives, and this method did not clearly improve the activity of benzimidazole anthelmintics scaffolds. In addition, when the organometallic derivatives were active, no definitive evidence were found in favor of a metal‐based mode of action of the compound.

## 3 Conclusions

This study aimed at designing new organometallic derivatives of the benzimidazole anthelmintic drugs, with the replacement of common lipophilic motifs that are found in the structures of the commercially available drugs that belong to this family. The lipophilic groups of these drugs were substituted by metallocenes, which could be at the origin of a unique metal‐based mode of action, in addition to the activity of the parent class of drug. A single route enabled the synthesis of 10 new benzimidazole carbamates, including six organometallic compounds (i.e., ferrocenyl and ruthenocenyl).

An in silico study was performed with a model of *H. contortus* β‐tubulin to confirm that the structural modifications undertaken in this series (three‐dimensional groups, additional methylene) would not prevent the compounds from fitting in the binding site of the targeted protein but the three‐dimensional chains (adamantyl and ferrocenyl) are associated with an increase in binding energy, which would indicate that they would be weaker inhibitors.

Biological activity of the benzimidazole derivatives was evaluated by screening against various nematodes. A broad range of activities were found depending on the species, but the organometallic derivatization only retained the activity of the parent drugs and failed to improve it. The benzimidazole derivatives **5a–c,f,g,** and **6a** showed activities that are in the same range as oxfendazole or fenbendazole against the free‐living nematode *C. elegans*. All compounds failed to induce nematocidal effect in the parasitic nematode *H. contortus*, but fenbendazole and its homolog **5c** induced abnormal larval development. Extending the screening to other parasitic nematode species enabled us to find biological activity for several organometallic derivatives in the same range as the benzimidazole drugs. At 10 µM, like fenbendazole, compounds **5e** and **6a** were active against *S. ratti*, compounds **5c,e,g** were active against *A. ceylanicum* and the whole series was mildly active against *T. muris*. No compounds were active against *H. polygyrus* or *N. americanus*.

Finally, the screening was extended to other parasites. Benefits of the organometallic derivatization were found against *Schistosoma mansoni*, as the ferrocenyl and ruthenocenyl **5a,b,f** displayed activity against larval and adult stages of *Schistosoma mansoni* in the micromolar range but no proof was found accounting for a specific role of the metal in the antiparasitic effect. Similarly, the ferrocenyl derivative **6a** was active against *E* metacestode vesicles, but none of the organometallic derivatives showed activities against *E. multilocularis* germinal layer cells, including stem cells, as observed for other previously tested benzimidazole carbamates. The lack of activity of currently used benzimidazoles against parasite stem cells is considered to underlie their predominantly parasitostatic rather than parasiticidal effect in patients with alveolar echinococcosis [71]. Finally, compounds **6d** and **5f** showed activity against *L. infantum* promastigotes at 50 µM but they are also cytotoxic against the host cells at this concentration, which makes them unsuitable for further antileishmanial assays.

The cytotoxicity of the benzimidazole derivatives was determined against healthy human cells (RPE‐1), and IC_50_ values were found in the micromolar range like the antiparasitic effects. Such results could suggest a lack of selectivity against parasites and would be a limiting factor for further therapeutic development.

Further studies would be necessary to better understand the mechanisms of action of these compounds against each organism, especially to understand the role of the organometallic moieties when these derivatives displayed higher parasiticidal effects than the benzimidazole drugs. Improved knowledge on the mechanism of action and the targets would be required to enhance the activity of the organometallic benzimidazole derivatives.

## Author Contributions

**C.L.:** designed, synthesized, characterized the compounds, and performed docking study. **C.L.** and **P.M.:** performed cytotoxicity on RPE‐1 cells. **A.C.T.** and **J.J.B.:** performed the screening against *C. elegans* and *H. contortus*. **O.B.:** undertook crystallographic studies. **C.H.:** performed the screening against *A. ceylanicum*, *H. polygyrus*, *N. americanus*, *S. mansoni*, *S. ratti*, and *T. muris*. **T.Z.:** performed the screening against *E. multilocularis*. **G.S.** and **L.P.:** performed the screening against *L. infantum*. **D.G.**, **B.L.S.**, and **J.K.:** revised the manuscript. **J.K.**, **R.B.G.**, **K.C.**, and **G.G.:** conceived the project, supervised and revised the manuscript.

## Funding

This study was supported by Direction Générale de l’Armement, Agence Nationale de la Recherche, Australian Research Council, and Oz Omics Pty Ltd.

## Conflicts of Interest

The authors declare no conflicts of interest.

## Supporting information

Supplementary Material

## Data Availability

All data associated with this study are present in the article or the Supporting Information.
