# Supplementary material for: Synthesis and Antiparasitic Activities of New Organometallic Derivatives of the Benzimidazole Anthelmintic Drugs
Source: Chembiochem. 2026 Aug 9;27(15):e70490. doi: 10.1002/cbic.70490 (PMC13453072; doi:10.1002/cbic.70490)
Supplement: Supplementary file 1 [file CBIC-27-e70490-s001.pdf]

Supplementary information

Synthesis and Antiparasitic Activities of New Organometallic Derivatives of the Benzimidazole Anthelmintic Drugs

Corentin Ludwig,[a] , Aya C. Taki,[b] Joseph J. Byrne,[b] Pierre Mesdom,[a] Olivier Blacque,[c] Cécile Häberli,[d,e] Trix Zumkehr,[f] Gonzalo Scalese,[h,i,j] Leticia Pérez-Díaz,[j] Dinorah Gambino,[h] Britta Lundström-Stadelmann,[f,g] Jennifer Keiser,[d,e,*] Robin B. Gasser,[b,*] Kevin Cariou,[a,*] and Gilles Gasser[a,*]

a) Chimie ParisTech, PSL University, CNRS, Institute of Chemistry for Life and Health Sciences, Laboratory for Inorganic Chemical Biology, 75005 Paris, France.
b) Department of Veterinary Biosciences, Melbourne Veterinary School, Faculty of Sciences, The University of Melbourne, Parkville, Victoria 3010, Australia.
c) Department of Chemistry, University of Zurich, Winterthurerstrasse 190, 8057 Zurich, Switzerland.
d) Swiss Tropical and Public Health Institute, Kreutzstrasse 2, Allschwil, Switzerland.
e) University of Basel, Basel, Switzerland.
f) Institute of Parasitology, Vetsuisse Faculty, University of Bern, Bern, Switzerland.
g) Multidisciplinary Center for Infectious Diseases, University of Bern, Bern, Switzerland.
h) Área Química Inorgánica, Facultad de Química, Universidad de la República, 11800 Montevideo, Uruguay.
i) Laboratory Redox Biology of Trypanosomes, Institut Pasteur de Montevideo, 11400 Montevideo, Uruguay.
j) Sección Genómica Funcional, Facultad de Ciencias, Universidad de la República, 11400 Montevideo, Uruguay.

Table of contents

1. Experimental section in chemistry ... *3*

1.1. Materials and methods in chemistry ... 3

1.2. Abbreviations ... 4

1.3. Synthesis and characterization of the metallocenyl building blocks ... 5

1.4. Synthesis and characterization of the benzimidazole derivatives ... 15

1.5. NMR spectra for characterization ... 41

1.6. ^{1}H NMR spectra for stability in DMSO ... 86

1.7. Infrared spectra ... 91

1.8. X-ray crystallography data ... 103

1.9. Molecular docking ... 117

2. Experimental section in biology ... *121*

2.1. Production and preparation of *C. elegans* and *H. contortus* larvae for bioassays ... 121

2.2. Bioassay for the evaluation of anthelmintic activity using larvae of *C. elegans* or *H. contortus* ... 122

2.3. Ethics for NTS, S. mansoni, T. muris, H. polygyrus, N. americanus, A. ceylanicum and S. ratti. ... 122

2.4. *In vitro* evaluation on newly transformed schistosomula (NTS) ... 123

2.5. *In vitro* evaluation on adult *S. mansoni* ... 123

2.6. *In vitro* evaluation on adult *T. muris* ... 124

2.7. *In vitro* evaluation on *H. polygyrus* (L3) ... 124

2.8. In vitro evaluation on N. americanus (L3) and A. ceylanicum L3 ... 125

2.9. *In vitro* evaluation on *S. ratti* (L3) ... 125

2.10. Ethics, strain maintenance and culture of *E. multilocularis* ... 126

2.11. *In vitro* evaluation on *E. multilocularis* germinal layer cells ... 126

2.12. *In vitro* evaluation on *E. multilocularis* metacestode vesicles ... 127

2.13. *L. infantum* parasites ... 128

2.14. In vitro screening against promastigotes of L. infantum ... 128

2.15. *L. infantum* promastigotes proliferation ... 129

2.16. Murine macrophages J774 ... 129

2.17. RPE-1 Cells culture ... 130

2.18. Cell viability assay (RPE-1) ... 130

2.19. Biological drawings ... 131

3. References ... *132*

1. Experimental section in chemistry

1.1. Materials and methods in chemistry

All the manipulations that required the use of an inert atmosphere were performed using regular Schlenk methods. Commercially available reagents and solvents were used without further purification. Evaporation of solvents *in vacuo* was done with a rotary evaporator at 40°C. Thin layer chromatography (TLC) was performed using silica gel 60 F-254 (Merck) plates with detection of spots being achieved by exposure to UV light. Purification by column chromatography were performed using Combi*Flash*® Rf and Agela silica flash columns (4 g to 120 g). Purification by preparative TLC were performed using silica gel 60 plates (Merck).

NMR spectra were recorded in deuterated solvents on Bruker AV-400 (^{1}H, 400 MHz; ^{13}C, 101 MHz) at room temperature. The chemical shifts, δ, are reported in ppm (parts per million). The residual solvent peaks have been used as an internal reference ($CDCl_3$, residual peak: 7.26 ppm (^{1}H) and 77.16 ppm (^{13}C); $(CD_3)_2SO$, residual peak: 2.50 ppm (^{1}H) and 39.52 ppm (^{13}C).[1] All the deuterated solvents were purchased from Eurisotop with ≥99.8 % deuterium level. The abbreviations for the peak multiplicities are as follows: s (singlet), d (doublet), t (triplet), q (quadruplet), br s (broad singlet), and m (multiplet).

Mass spectrometry was performed with Thermo Scientific (Thermo Fisher Scientific) DSQ II for Electron Ionization (20 eV) and Chemical Ionization (NH_3) or Electrospray ionization-mass spectrometry (ESI-MS) experiments were measured using a Thermo Scientific (Thermo Fisher Scientific) LTQ-Orbitrap XL instrumentation in positive mode. Atmospheric pressure chemical ionization- mass spectrometry (APCI-HRMS) were performed on an Agilent Q-TOF 6546 coupled to an Agilent 1260 Infinity II HPLC.

Infrared spectroscopic measurements were carried out on a Thermo Fisher Nicolet IS 20 spectrometer as solid deposit on KBr.

Known intermediates compounds were identified by NMR spectrometry (^{1}H). New compounds were identified by NMR spectrometry (^{1}H, ^{13}C), mass spectrometry and infrared spectroscopy.

Purity of the intermediate compounds (**3**, **4**) was established by NMR spectrometry and purity of the final compounds (**5**, **6**) was established by elemental analysis.

N,N-dimethylaminomethylferrocene, ruthenocene were bought from TCI Europe NV. (Hydroxyethyl)ferrocene was bought from BLD Pharmatech.

1.2. Abbreviations

DCM	Dichloromethane
DMF	Dimethylformamide
DMAP	*N,N*-Dimethylaminopyridine
EtOAc	Ethyl acetate
THF	Tetrahydrofuran
*t*BuOK	Potassium *tert*-butoxide
*t*BuLi	*tert*-butyllithium

1.3. Synthesis and characterization of the metallocenyl building blocks

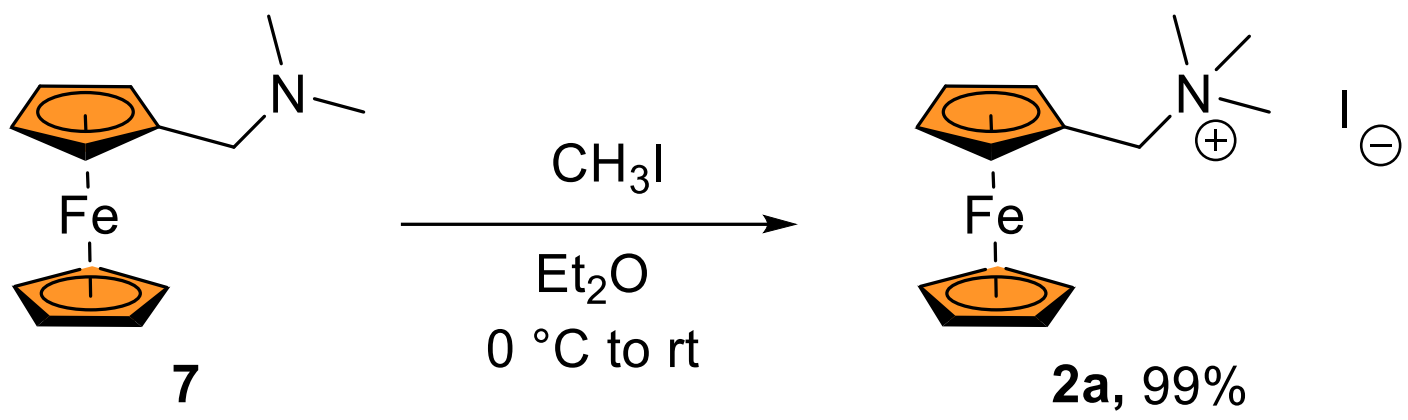

Scheme S1: Synthetic pathway for **2a**.

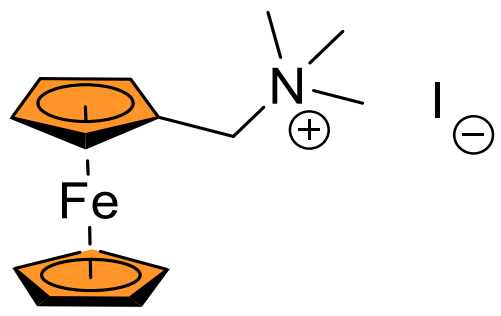

2a

((Ferrocenyl)methyl)trimethylammonium iodide **2a** was synthesized following an adapted literature procedure.[2] *N,N*-(Dimethylaminomethyl)ferrocene **7** (5 mmol, 1 mL) was dissolved in Et_2O (60 mL) at 0 °C and a solution of iodomethane (25 mmol, 1.5 mL) in Et_2O (15 mL) was added dropwise to the reaction flask. The reaction mixture was stirred for 2 hours and allowed to warm up to room temperature. Upon reaction, a yellow precipitate formed. Büchner filtration was performed to recover the yellow solid, which was further washed with Et_2O (3 x 10 mL). The residue was collected and dried under reduced pressure to afford a yellow powder (1.96 g, 99% yield).

^{1}H NMR (400 MHz, D_2O): δ 4.56 (t, *J* = 2.3 Hz, 2H, C_5H_4), 4.47 (t, *J* = 1.8 Hz, 2H, C_5H_4), 4.44 (s, 2H, CH_2), 4.33 (s, 5H, C_5H_5), 3.00 (s, 9H, 3 x CH_3).

The spectral data corresponds to previously reported data.[2]

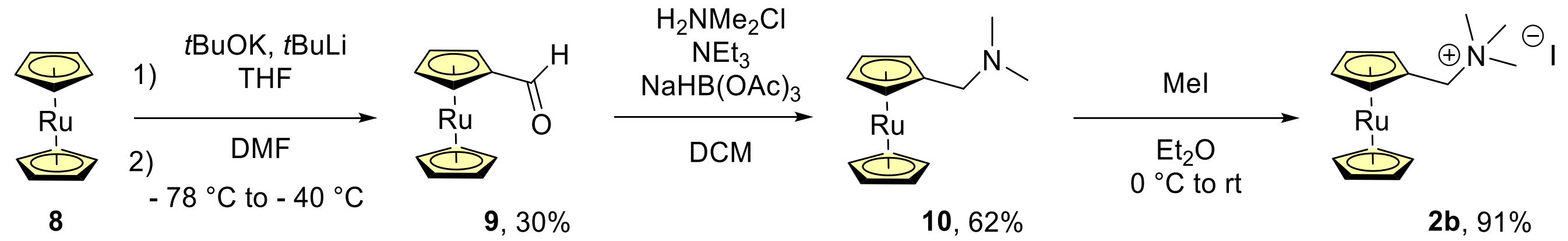

Scheme S2: Synthetic pathway for **2b**.

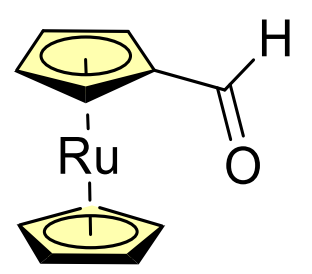

9

Compound **9** was synthesized following an adapted literature procedure.[3] In a dry flask, ruthenocene **8** (2.15 mmol, 498 mg) and *t*BuOK (1.075 mmol, 126 mg) were set under N_2 atmosphere and dissolved in dry THF (50 mL). The reaction mixture was cooled to – 78 °C and a solution of *t*BuLi in pentane (4.3 mmol, 4 mL, 1.1 M) was added. Following the addition, the reaction mixture was stirred from – 78 °C to – 45 °C within 1 hour. As it reached – 45 °C, dry DMF (0.4 mL) was added to the reaction mixture, which was left stirring for 16 hours and to reach room temperature. The reaction mixture was quenched with water (50 mL), THF was removed under reduced pressure, and the resulting aqueous layer was extracted with DCM (3 x 30 mL). The organic layers were combined, dried over $MgSO_4$ and filtered. The solvent was evaporated under reduced pressure to afford a yellowish residue, which was purified by flash column chromatography, using a 12 g silica column, and a gradient of EtOAc in cyclohexane (0 to 50%). Eluting at 10 % EtOAc in cyclohexane the product was a yellow solid (168 mg, 30% yield).

^{1}H NMR (400 MHz, $CDCl_3$) δ 9.73 (s, 1H, CHO), 5.09 (t, *J* = 1.9 Hz, 2H, C_5H_4), 4.88 (t, *J* = 1.8 Hz, 2H, C_5H_4), 4.65 (s, 5H, C_5H_5).

The spectral data corresponds to previously reported data.[3]

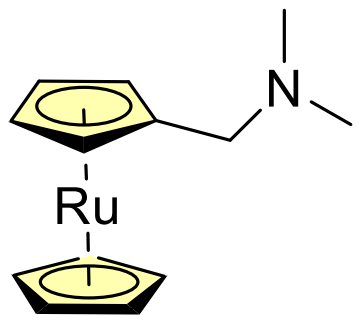

10

Compound **10** was synthesized following an adapted literature procedure.[4] To a solution of compound **9** (0.65 mmol, 170 mg) in DCM (5 mL), dimethylammonium chloride (0.85 mmol, 70 mg) and triethylamine (0.85 mmol, 0.1 mL) were added. The mixture was stirred for 1 hour at room temperature. Then, $NaHB(OAc)_3$ (1.95 mmol, 413 mg) was added portionwise and the mixture was stirred for 16 hours to consume all the starting material **9**. The reaction mixture was then poured onto water (10 mL) and stirred. NaOH (25 % weight aqueous solution) was added dropwise to basify the mixture to pH = 12. Extraction was performed with DCM (3 x 15 mL) and the combined organic layers were dried over $MgSO_4$. Solvent was removed and the collected residue was purified by flash column chromatography using 4 g silica column, and a gradient of MeOH in DCM (0 to 10%). Eluting at 10 % MeOH in DCM, the product was isolated as a yellowish oil (115 mg, 62% yield).

^{1}H NMR (400 MHz, $CDCl_3$) δ 4.57 (t, *J* = 1.7 Hz, 2H, C_5H_4), 4.49 – 4.45 (m, 7H, C_5H_4 and C_5H_5), 3.16 (s, 2H, CH_2), 2.23 (s, 6H, 2 x CH_3).

The spectral data corresponds to previously reported data.[4]

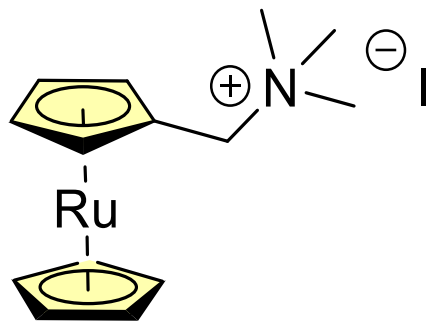

2b

Compound **2b** was synthesized following an adapted literature procedure.[2] Compound **10** (0.39 mmol, 115 mg) was dissolved in Et_2O (5 mL) at 0 °C and a solution of iodomethane (2 mmol, 0.15 mL) in Et_2O (1.5 mL) was added dropwise to the reaction flask. Once the addition of the solution was over, the reaction mixture was allowed to warm up to room temperature over 2 hours. Upon reaction, a pale yellowish precipitate was formed. Büchner filtration was performed to recover a white solid, which was further washed with Et_2O (2 x 10 mL). The recovered residue was further dried under reduced pressure to afford a white powder (153 mg, 91% yield).

^{1}H NMR (400 MHz, $(CD_3)_2SO$) δ 4.88 (t, *J* = 1.7 Hz, 2H, C_5H_4), 4.74 (t, *J* = 1.7 Hz, 2H, C_5H_4), 4.63 (s, 5H, C_5H_5), 4.12 (s, 2H, CH_2), 2.95 (s, 9H, 3 x CH_3).

The spectral data corresponds to previously reported data.[5]

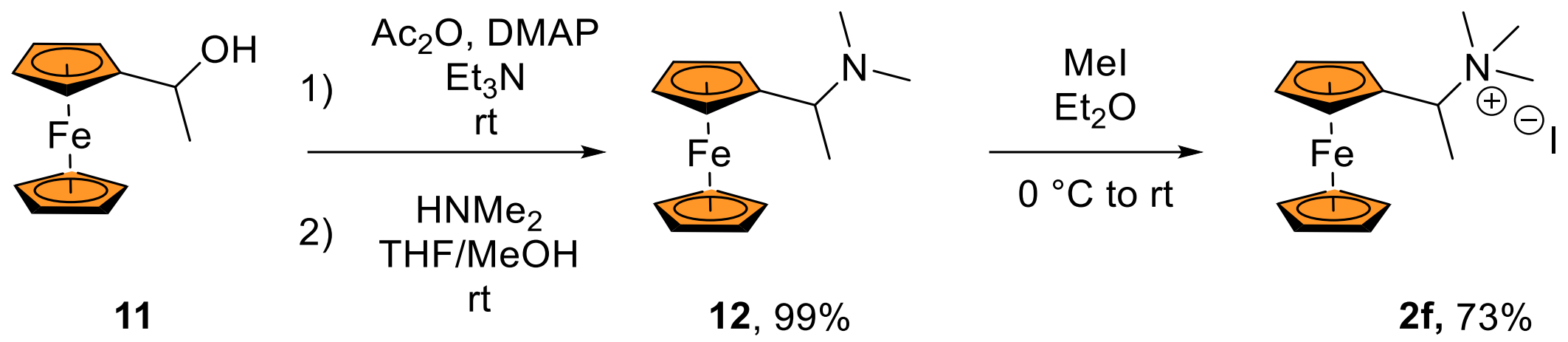

Scheme S3: Synthetic pathway for **2f**.

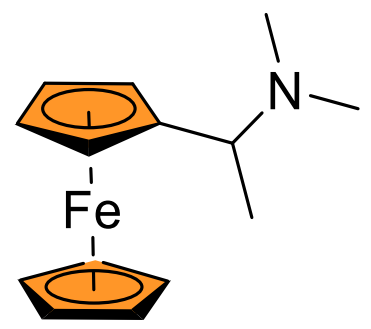

12

Compound **12** was synthesized following an adapted literature procedure.[6] (Hydroxyethyl)ferrocene **11** (2 mmol, 460 mg) was dissolved in triethylamine (3.5 mL). To the reaction mixture, *N,N*-dimethylaminopyridine (0.12 mmol, 15 mg) and acetic acid anhydride (20 mmol, 1.9 mL) were added. It was stirred for 16 hours at room temperature. The reaction mixture was then diluted in THF (15 mL) and a solution of dimethylamine (40 mmol, 20 mL, 2 M solution in MeOH) was added dropwise to the reaction mixture with an addition funnel. It was further stirred for 24 hours. The reaction mixture was poured onto Et_2O and water (50 mL : 50 mL), and an extraction was performed with Et_2O. The organic layer was filtered and dried over $MgSO_4$. The product was obtained as a dark orange oil (558 mg, 99%).

^{1}H NMR (400 MHz, $CDCl_3$) δ 4.23 – 4.07 (m, 9H, C_5H_4 and C_5H_5), 3.73 (q, J = 6.9 Hz, 1H, CH), 2.13 (s, 6H, $N(CH_3)_2$), 1.49 (d, J = 6.9 Hz, 3H, CH_3).

The spectral data corresponds to previously reported data.[6]

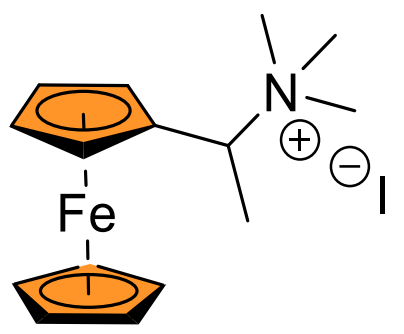

2f

Compound **2f** was synthesized following an adapted literature procedure.[2] Compound **12** (2 mmol, 558 mg) was dissolved in Et_2O (10 mL) at 0 °C and a solution of iodomethane (10 mmol, 0.6 mL) in Et_2O (5 mL) was added dropwise to the reaction flask. Once the addition of the solution ws over, the reaction mixture was stirred for 3 hours and allowed to warm up to room temperature. Upon addition, a yellow precipitate was formed. Büchner filtration was performed to recover the yellow solid, which was further washed with Et_2O (2 x 10 mL). The collected residue was further dried under reduced pressure to afford a yellow solid (585 mg, 73% yield).

^{1}H NMR (400 MHz, $(CD_3)_2SO$) δ 4.60 – 4.51 (m, 3H), 4.47 – 4.41 (m, 1H), 4.41 – 4.36 (m, 1H), 4.26 (s, 5H, C_5H_5), 2.85 (s, 9H, $N(CH_3)_3$), 1.82 (dt, *J* = 6.9, 1.9 Hz, 3H, CH_3). **^{13}C NMR** (101 MHz, $(CD_3)_2SO$) δ 80.3, 71.7, 70.4, 69.4, 69.3, 68.7, 68.4, 50.0, 14.7. **IR** (cm^{-1}): 3092, 3013, 2971, 2738, 2458, 1645, 1473, 1414, 1398, 1366, 1305, 1253, 1236, 1105, 1024, 1000, 816. **MS** (ESI): m/z = 258.10 ($[M\text{-}I\text{-}CH_3+H]^+$).

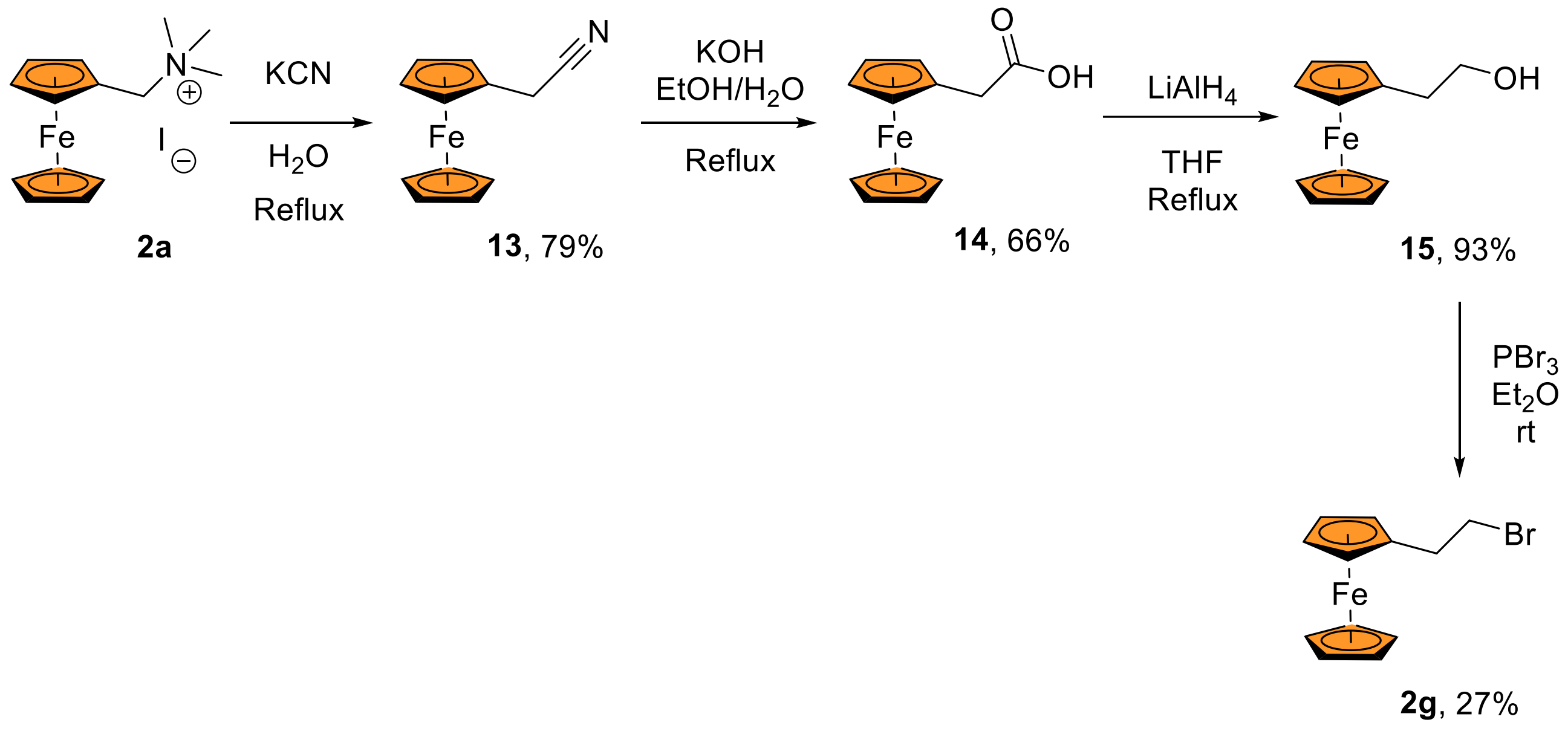

Scheme S4: Synthetic pathway for **2g**.

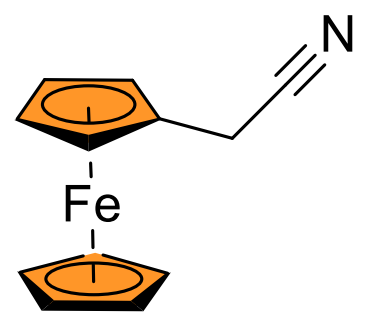

13

Compound **13** was synthesized following an adapted literature procedure.[7] Potassium cyanide (22.6 mmol, 1.47 g) was dissolved in water (15 mL) and stirred vigorously for 30 minutes. To this aqueous solution, compound **2a** was added (3.9 mmol, 1.5 g) and the mixture was refluxed for 24 hours. The reaction mixture was cooled to room temperature and extracted with Et_2O. The organic layers were dried over $MgSO_4$, filtered and concentrated under reduced pressure to afford an orange oil (700 mg, 79% yield).

^{1}H NMR (400 MHz, $CDCl_3$) δ 4.26 – 4.21 (m, 7H, C_5H_5 and C_5H_4), 4.17 (dd, J = 3.3, 1.4 Hz, 2H, C_5H_4), 3.41 (s, 2H, CH_2).

The spectral data corresponds to previously reported data.[8]

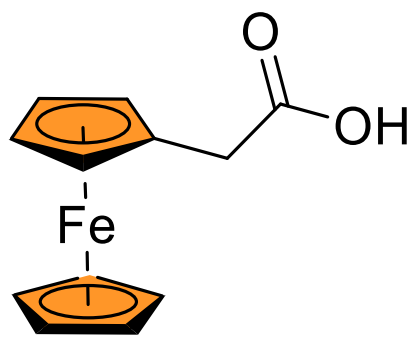

14

Compound **14** was synthesized following an adapted literature procedure.[9] A solution of compound **13** (3 mmol, 680 mg) in EtOH (7 mL) was mixed with a solution of potassium hydroxide (30 mmol, 1.68 g) in water (17 mL). The mixture was refluxed overnight. Ethanol was then removed under reduced pressure and the resulting aqueous mixture was diluted with water (20 mL). The aqueous layer was washed with Et_2O (3 x 30 mL). The aqueous layer was then acidified with H_3PO_4 until both a colour change (from orange to yellow) and a precipitate were observed. Büchner filtration was performed, and the residue was washed with water and further dried under reduced pressure. A dark yellow solid (500 mg, 66% yield) was recovered.

^{1}H NMR (400 MHz, $CDCl_3$) δ 4.25 – 4.19 (m, 2H, C_5H_4), 4.17 – 4.09 (m, 7H, C_5H_5 and C_5H_4), 3.43 (s, 2H, CH_2). COOH not seen.

The spectral data corresponds to previously reported data.[8]

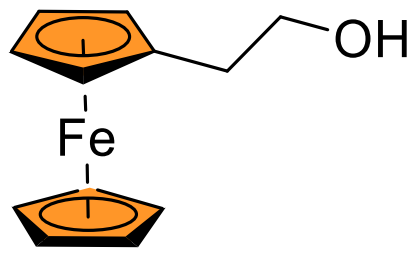

15

Compound **15** was synthesized following an adapted literature procedure.[9] $LiAlH_4$ (7.3 mmol, 279 mg) was set under N_2 atmosphere in a two-necked round bottom flask and dissolved in dry THF (10 mL). The suspension was refluxed for 15 minutes. In another flask, a solution of compound **14** (2 mmol, 500 mg) in dry THF (10 mL) was prepared under N_2 atmosphere. The solution was added dropwise to the suspension of $LiAlH_4$. The reaction mixture was refluxed until consumption of the starting material **14**. The reaction mixture was cooled to 0 °C and quenched with an aqueous solution of NaOH (1 mL, 1 M) and water (10 mL). It was filtered and the filtrate was extracted with Et_2O (2 x 10 mL). The organic layers were combined, dried over $MgSO_4$, filtered and the solvent was removed under reduced pressure to afford a dark orange oil (439 mg, 93% yield).

^{1}H NMR (400 MHz, $CDCl_3$) δ 4.12 (s, 5H, C_5H_5), 4.12 (d, *J* = 1.8 Hz, 2H, C_5H_4), 4.10 (d, *J* = 1.8 Hz, 2H, C_5H_4), 3.81 – 3.70 (m, 2H, CH_2), 2.60 (t, *J* = 6.5 Hz, 2H, CH_2). OH not seen.

The spectral data corresponds to previously reported data.[10]

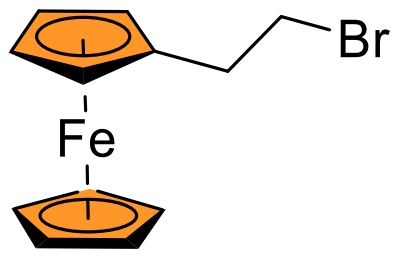

2g

Compound **2g** was synthesized following an adapted literature procedure.[9] Compound **15** (1.9 mmol, 440 mg) was dissolved in dry Et_2O (5 mL) under N_2 atmosphere and PBr_3 (0.2 mL) was added dropwise to the reaction mixture. It was stirred at room temperature for 2 hours. The reaction was quenched with a saturated $NaHCO_3$ aqueous solution at 0 °C, extracted with Et_2O (3 x 10 mL) and dried over $MgSO_4$ and filtered. The resulting residue was purified by flash column chromatography, using a 12 g silica column, and a gradient of EtOAc in cyclohexane (0 to 100%). Eluting with cyclohexane, the product was isolated as an orange solid (150 mg, 27% yield).

^{1}H NMR (400 MHz, $CDCl_3$) δ 4.19 – 4.08 (m, 9H, C_5H_4 and C_5H_5), 3.45 (t, *J* = 7.3 Hz, 2H, CH_2), 2.91 (t, *J* = 7.7 Hz, 2H, CH_2).

The spectral data corresponds to previously reported data.[9]

1.4. Synthesis and characterization of the benzimidazole derivatives

General Procedure A

To a 1 M solution of KOH in EtOH (KOH, 85% weight: 0.7 g ; EtOH: 10 mL) cooled at 0 °C, 2-nitro-4-thiocyanatoaniline (1 equivalent) was added portionwise. The reaction mixture warmed up to room temperature over 30 minutes. A solution of H_2SO_4 in EtOH (10 % volume) was slowly added to the reaction mixture. After addition of the acidic solution, the color of the reaction medium changed from dark purple to dark orange. EtOH was removed under reduced pressure and a dark orange liquid was recovered. It was poured onto water (30 mL) and an extraction was performed with EtOAc (3 x 30 mL). The organic layers were combined, dried with $MgSO_4$ and filtered. The solvent was removed under reduced pressure to afford a dark red solid. The solid was then dissolved in dry and degassed DMF (5 mL). K_2CO_3 (5 equivalents) and the electrophile **2a-g** (1 equivalent) were added to the solution. Upon addition of K_2CO_3 to the solution, the color of the reaction mixture changed from dark orange to dark purple. The reaction mixture was stirred overnight at 60 °C. DMF was removed under reduced pressure. The residue was dissolved in 10 mL of water, extracted with EtOAc (3 x 10 mL). The organic layers were combined, dried over $MgSO_4$ and filtered.

General Procedure B

To a solution of nitroaniline **3a-g** (1 equivalent) in EtOH, Pd/C (5 mol%) was added. The solution was refluxed and then hydrazine monohydrate (10 equivalents) was added portionwise over 30 minutes. The reflux was performed until consumption of the starting material. The reaction mixture was filtered over celite, and the residue was washed with EtOAc (20 mL). Water was added to the filtrate (10 mL), and the solvents were removed under reduced pressure. The resulting aqueous solution was extracted with EtOAc (3 x 10 mL) the organic layers were combined, dried with $MgSO_4$ and filtered. The solvent was removed under reduced pressure.

General Procedure C

Diaminophenylene **4a-g** (1 equivalent) and 1,3-bis(methoxycarbonyl)-2-methyl-2-thiopseudourea (1 equivalent) were dissolved in $EtOH/H_2O$ (1:1) mixture and acetic acid (1.1 equivalents) was added. The mixture was refluxed overnight. The reaction mixture was diluted with a large excess of water to form

a precipitate, which is recovered by Büchner filtration. The residue was further dried under reduced pressure.

General Procedure D

Crystals of phenol (12 equivalent) were melted by warming to 45°C. The thioether **5a,c-d** was dissolved in the liquid phenol and the solution was degassed with N_2. Solution of 35 % H_2O_2 in H_2O (0.02 mL per 0.1 mmol) was slowly added to the reaction mixture, and it was stirred for 5 minutes. The crude was poured onto a mixture EtOAc (10 mL), a saturated Na_2SO_3 aqueous solution (10 mL) and 10 % NaOH solution (10 mL). It was stirred for 5 minutes then extracted with EtOAc (3 x 10 mL). The organic layers were combined, dried over $MgSO_4$ and filtered.

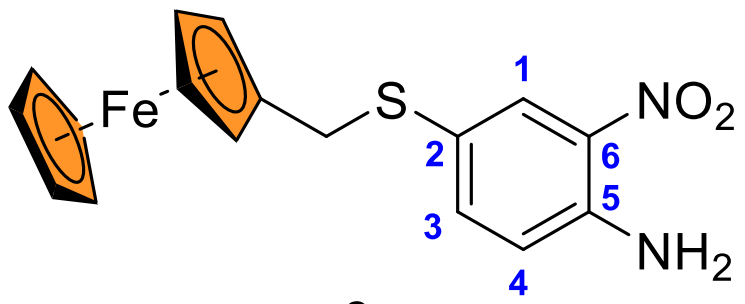

3a

Compound **3a** was prepared following the general procedure **A**, using in the first step 2-nitro-4-thiocyanatoaniline (2 mmol, 390 mg) and 10 mL of 1 M KOH in EtOH solution. The red residue obtained was taken to the second step with K_2CO_3 (10 mmol, 1.4 g), **2a** (2 mmol, 800 mg) and DMF (5 mL). The crude mixture was purified by flash column chromatography, using a 20 g silica column and a gradient of EtOAc in heptane (0 to 30 %). Eluting at 20 % EtOAc in heptane the product was obtained as a red oil (361 mg, 49 % yield).

^{1}H NMR (400 MHz, $CDCl_3$) δ 8.15 (d, *J* = 2.1 Hz, 1H, H1), 7.29 (dd, *J* = 8.6, 2.1 Hz, 1H, H3), 6.71 (d, *J* = 8.7 Hz, 1H, H4), 6.08 (br s, 2H, NH_2), 4.13 (s, 5H, C_5H_5), 4.09 (t, *J* = 1.9 Hz, 2H, C_5H_4), 4.06 (t, *J* = 1.8 Hz, 2H, C_5H_4), 3.81 (s, 2H, CH_2). **^{13}C NMR** (101 MHz, $CDCl_3$) δ 144.0 (C5), 140.4 (C3), 130.0 (C1), 125.3 (C6), 123.5 (C2), 119.2 (C4), 84.3 (C_5H_4), 68.9 (C_5H_4), 68.3 (C_5H_4), 37.0 (CH_2). **IR** (cm^{-1}): 3474, 3363, 3335, 2919, 1626, 1582, 1554, 1502, 1450, 1402, 1360, 1334, 1235, 1173, 1104, 1038, 1023, 1002, 824. **MS** (CI): m/z = 368.10 ($[M]^+$).

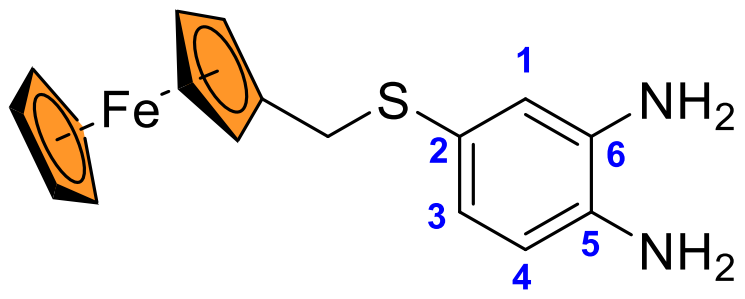

4a

Compound **4a** was prepared following the general procedure **B**, using **3a** (350 mg, 0.95 mmol) and Pd/C (0.005 mmol, 4 mg) in EtOH (10 mL), followed by the addition of hydrazine monohydrate (3.2 mL, 10 mmol). A solid deposit was prepared and the crude mixture was purified by flash column chromatography, using a 20 g silica column and a gradient of EtOAc in cyclohexane (0 to 80 %). Eluting at 50 % EtOAc in cyclohexane, the product was obtained as a dark orange oil (245 mg, 76 % yield).

^{1}H NMR (400 MHz, $CDCl_3$) δ 6.79 – 6.73 (m, 2H, H1 and H3), 6.61 (dd, *J* = 7.6, 0.6 Hz, 1H, H4), 4.11 (s, 5H, C_5H_5), 4.09 – 4.06 (m, 4H, C_5H_4), 3.78 (s, 2H, CH_2), 3.38 (br s, 4H, 2 x NH_2). **^{13}C NMR** (101 MHz, $CDCl_3$) δ 135.0 (C6), 134.6 (C5), 126.1 (C2), 125.0 (C3), 121.2 (C4), 117.0 (C1), 85.2 (C_5H_4), 68.8 (C_5H_4), 68.8 (C_5H_5), 68.0 (C_5H_4), 37.3 (CH_2). **IR** (cm^{-1}): 3193, 3089, 2921, 1579, 1501, 1417, 1281, 1240, 1104, 1037, 1022, 1000, 822, 819. **MS** (CI): m/z = 339.15 ($[M+H]^+$).

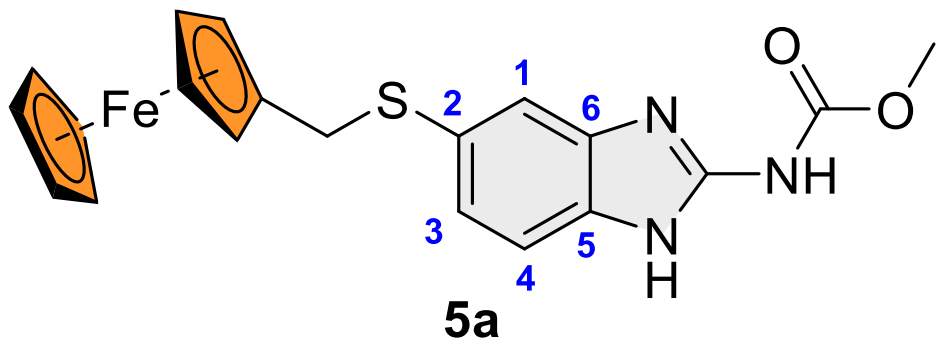

5a

Compound **5a** was prepared following the general procedure **C**, using **4a** (240 mg, 0.64 mmol), 1,3-bis(methoxycarbonyl)-2-methyl-2-thiopseudourea (140 mg, 0.64 mmol) and acetic acid (0.05 mL, 0.8 mmol) in EtOH/H_2O (2.5 mL : 2.5 mL). The crude residue was purified by flash column chromatography using a 12 g silica column, and a gradient of MeOH in DCM (0 to 5%). Eluting at 2.5 % MeOH in DCM, the product was isolated as a yellow solid (225 mg, 83 % yield).

^{1}H NMR (400 MHz, $(CD_3)_2SO$) δ 11.62 (br s, 2H, 2 x NH), 7.41 (dd, *J* = 1.8, 0.6 Hz, 1H, H1), 7.33 (dd, *J* = 8.3, 0.6 Hz, 1H, H4), 7.09 (dd, *J* = 8.3, 1.8 Hz, 1H, H3), 4.15 (s, 5H, C_5H_5), 4.10 (t, *J* = 1.8 Hz, 2H, C_5H_4), 4.07 (t, *J* = 1.8 Hz, 2H, C_5H_4), 3.93 (s, 2H, CH_2), 3.76 (s, 3H, CH_3). **^{13}C NMR** (101 MHz, $(CD_3)_2SO$) δ 154.8, 147.8, 127.1, 124.4 (C3), 116.3 (C2), 114.0 (C4), 84.7, 68.6 (C_5H_5), 68.5 (C_5H_4), 67.5 (C_5H_4), 52.5 (CH_3), 35.6 (CH_2). **IR** (cm^{-1}): 3330, 3091, 2672, 1706, 1630, 1588, 1523, 1460, 1326, 1272, 1225, 1195, 1121, 1104, 1022, 1000, 957, 923, 861, 761. **HRMS** (APCI): Found, m/z = 420.0667 ($[M+H]^+$). Calculated for $C_{20}H_{19}FeN_3O_2S$, 419.0597. **Elemental analysis**: Calculated for $C_{20}H_{19}FeN_3O_2S$ = C, 57.02; H, 4.55; N, 9.97. Found = C, 56.88; H, 4.43; N, 10.10.

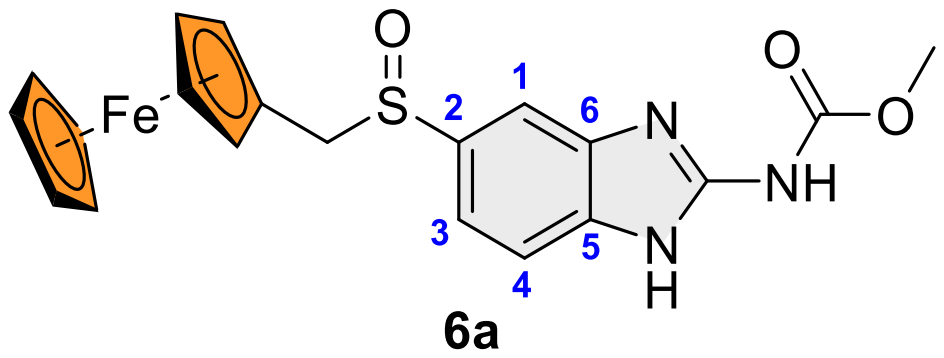

6a

Compound **6a** was prepared following the general procedure **D** with **5a** (50 mg, 0.12 mmol), H_2O_2 aqueous solution (0.02 mL) and phenol (0.1 mL). The residue obtained was purified by flash column chromatography using a 4 g silica column, and a gradient of MeOH in DCM (0 to 10%). Eluting at 5% MeOH in DCM, the product was isolated as an orange solid (38 mg, 71% yield).

^{1}H NMR (400 MHz, $(CD_3)_2SO$) δ 11.84 (br s, 2H, 2 x NH), 7.57 (d, *J* = 1.7 Hz, 1H, H1), 7.51 (d, *J* = 8.2 Hz, 1H, H4), 7.23 (dd, *J* = 8.2, 1.7 Hz, 1H, H3), 4.13 (s, 5H, C_5H_5), 4.09 (dt, *J* = 2.4, 1.3 Hz, 2H, C_5H_4), 4.00 (dt, *J* = 2.4, 1.5 Hz, 2H, C_5H_4), 3.91 (d, *J* = 13.2 Hz, 1H, CH_2), 3.84 (d, *J* = 13.2 Hz, 1H, CH_2), 3.77 (s, 3H, CH_3). **^{13}C NMR** (101 MHz, $(CD_3)_2SO$) δ 154.4, 148.5, 135.9, 117.1 (H3), 110.0 (H1), 76.8 (C_5H_4), 69.7 (C_5H_4), 69.5 (C_5H_4), 68.7 (C_5H_5), 68.2 (C_5H_4), 68.1 (C_5H_4), 59.2 (CH_2), 52.6 (CH_3). **IR** (cm^{-1}): 3326, 3090, 2670, 1730, 1639, 1589, 1523, 1453, 1428, 1374, 1272, 1225, 1197, 1130, 1052, 925, 870, 818, 767. **HRMS** (APCI): Found, m/z = 436.0612 ($[M+H]^+$). Calculated for $C_{20}H_{19}FeN_3O_3S$, 435.0543. **Elemental analysis**: Calculated for $C_{20}H_{19}FeN_3O_3S$ = C, 54.93; H, 4.38; N, 9.61. Found = C, 53.97; H, 4.31; N, 9.27.

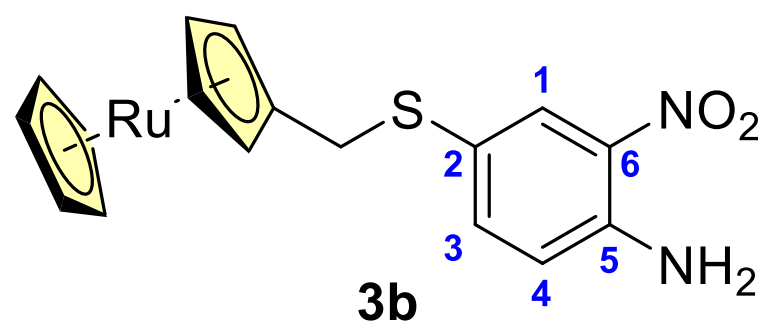

3b

Compound **3b** was synthesized following the general procedure **A**, using in the first step 2-nitro-4-thiocyanatoaniline (180 mg, 0.5 mmol) and 2.5 mL of 1 M KOH in EtOH solution. The red residue obtained (200 mg) was taken to the second step with K_2CO_3 (241 mg, 1.75 mmol) and **2b** (60 mg, 0.35 mmol) in DMF (5 mL). Purification was performed by flash column chromatography using a 20 g silica column, and a gradient of EtOAc in cyclohexane (0 to 50%). Eluting at 15% EtOAc in cyclohexane, the product was isolated as a red solid (76 mg, 52% yield).

^{1}H NMR (400 MHz, $CDCl_3$) δ 8.15 (d, *J* = 2.1 Hz, 1H, H1), 7.33 (dd, *J* = 8.7, 2.2 Hz, 1H, H3), 6.74 (d, *J* = 8.7 Hz, 1H, H4), 6.13 (br s, 2H, NH_2), 4.52 (s, 5H, C_5H_5), 4.50 (t, *J* = 1.7 Hz, 2H, C_5H_4), 4.45 (d, *J* = 1.7 Hz, 2H, C_5H_5), 3.66 (s, 2H, CH_2). **^{13}C NMR** (101 MHz, $CDCl_3$) δ 144.0 (C5), 140.3 (C3), 132.2 (C6), 130.0 (C1), 123.6 (C2), 119.3 (C4), 87.5 (C_5H_4), 71.4 (C_5H_4), 71.0 (C_5H_5), 70.3 (C_5H_4), 36.7 (CH_2). **IR** (cm^{-1}): 3485, 3369, 3170, 3097, 2972, 2924, 2868, 1623, 1587, 1551, 1467, 1402, 1352, 1333, 1246, 1167, 1121, 1078, 898, 818. **MS** (CI): m/z = 415.03 ($[M+H]^+$).

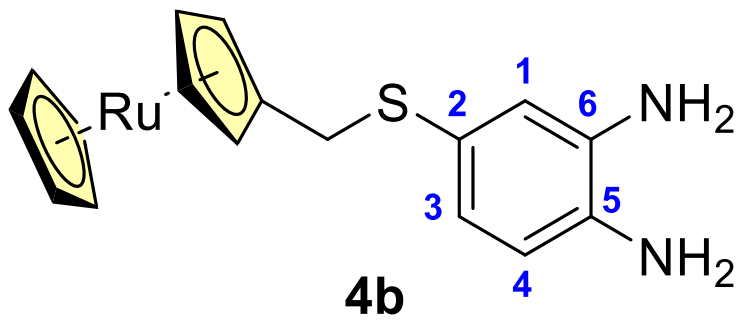

4b

Compound **4b** was synthesized following the general procedure **B**, using **3b** (177 mg, 0.42 mmol), Pd/C (2 mg, 0.02 mmol) in EtOH (10 mL), followed by the addition of hydrazine monohydrate (1.15 mL, 4.2 mmol). The resulting white solid was not further purified (160 mg, 99% yield).

^{1}H NMR (400 MHz, $CDCl_3$) δ 6.83 – 6.73 (m, 2H), 6.66 – 6.58 (m, 1H), 4.54 – 4.50 (m, 7H, C_5H_4 and C_5H_5), 4.44 (t, *J* = 1.7 Hz, 2H, C_5H_4), 3.62 (s, 2H, CH_2), 3.21 (br s, 4H, 2 x NH_2). **^{13}C NMR** (101 MHz, $CDCl_3$) δ 135.0 (C6), 134.6 (C5), 126.0 (C2), 125.0 (C3), 121.1 (C4), 117.0 (C1), 88.5 (C_5H_5), 71.4 (C_5H_5), 70.9 (C_5H_5), 70.1 (C_5H_4), 37.0 (CH_2). **IR** (cm^{-1}): 3386, 3351, 3308, 2963, 2924, 1618, 1577, 1502, 1448, 1419, 1276, 1262, 1245, 1163, 1099, 1022, 965, 912, 808. **MS** (CI): m/z = 385.09 ($[M+H]^+$).

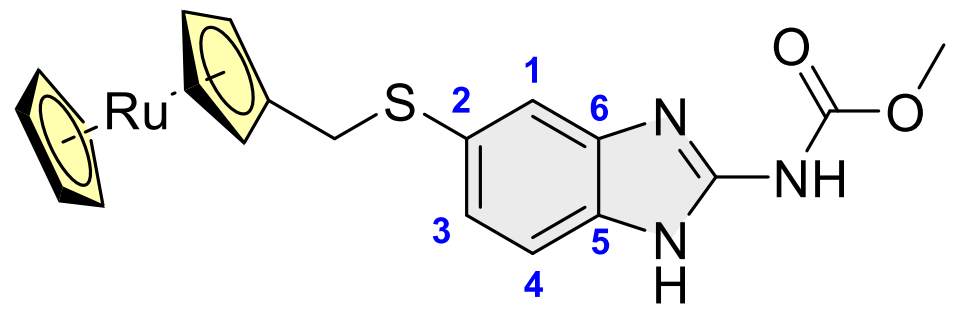

5b

Compound **5b** was prepared following the general procedure **C**, using **4b** (160 mg, 0.42 mmol), 1,3-bis(methoxycarbonyl)-2-methyl-2-thiopseudourea (84 mg, 0.42 mmol) and acetic acid (0.04 mL, 0.5 mmol) in EtOH/H_2O (7.5 mL : 7.5 mL). The crude residue was purified by flash column chromatography using a 20 g silica column, and a gradient of MeOH in DCM (0 to 10%). Eluting at 5% MeOH in DCM, the product was isolated as a white solid (95 mg, 49% yield).

^{1}H NMR (400 MHz, $(CD_3)_2SO$) δ 11.60 (br s, 2H, 2 x NH), 7.41 (d, *J* = 1.7 Hz, 1H, H1), 7.33 (d, *J* = 8.2 Hz, 1H, H4), 7.09 (dd, *J* = 8.3, 1.8 Hz, 1H, H3), 4.55 – 4.51 (m, 7H, C_5H_4 and C_5H_5), 4.43 (t, *J* = 1.7 Hz, 2H, C_5H_4), 3.77 – 3.72 (m, 5H, CH_2 and CH_3). **^{13}C NMR** (101 MHz, $(CD_3)_2SO$) δ 154.8 (CO), 147.8, 127.1, 124.5, 115.5 (C1), 87.9 (C_5H_4), 71.1 (C_5H_4), 70.8 (C_5H_5), 69.9 (C_5H_4), 52.5 (CH_3), 35.4 (CH_2). **IR** (cm^{-1}): 3396, 3097, 3015, 2950, 2905, 2759, 1719, 1654, 1597, 1521, 1460, 1441, 1416, 1279, 1231.73, 1195, 1099, 864, 810, 760. **HRMS** (APCI): Found, m/z = 462.0350 ($[M+H]^+$). Calculated for $C_{20}H_{19}RuN_3O_2S$, 461.0276. **Elemental analysis**: Calculated for $C_{20}H_{19}RuN_3O_2S$ = C, 51.49; H, 4.11; N, 9.01. Found = C, 51.12; H, 3.80; N, 8.89.

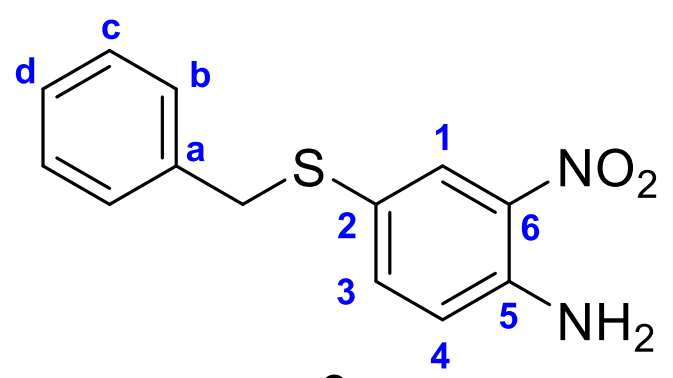

3c

Compound **3c** was synthesized following the general procedure **A**, using in the first step 2-nitro-4-thiocyanatoaniline (500 mg, 2.5 mmol) and 12.5 mL of 1 M KOH in EtOH solution. The red residue obtained (600 mg) was taken to the second step with K_2CO_3 (1.725 g, 12.5 mmol) and benzyl bromide (0.55 mL, 3.75 mmol) in DMF (5 mL). Purification was performed by flash column chromatography using a 20 g silica column, and a gradient of EtOAc in cyclohexane (0 to 90%). Eluting at 20% EtOAc in cyclohexane, the product was isolated as a red solid (422 mg, 65% yield).

^{1}H NMR (400 MHz, $CDCl_3$) δ 8.10 (d, *J* = 2.1 Hz, 1H, H1), 7.33 – 7.15 (m, 6H, Ha-d and H3), 6.67 (d, *J* = 8.7 Hz, 1H, H4), 6.08 (br s, 2H, NH_2), 3.99 (s, 2H, CH_2). **^{13}C NMR** (101 MHz, $CDCl_3$) δ 144.1, 140.3, 137.5, 132.2, 130.3, 129.1 (Cc), 128.6 (Cb), 127.4, 122.7 (C2), 119.2, 41.1 (CH_2). **IR** (cm^{-1}): 3476, 3354, 3169, 3084, 3064, 3028, 2927, 1939, 1654, 1618, 1600, 1560, 1549, 1497, 1452, 1400, 1357, 1336, 1268, 1244, 1203, 1165, 1111, 1069, 896, 829. **MS** (CI): m/z = 261.19 ($[M+H]^+$).

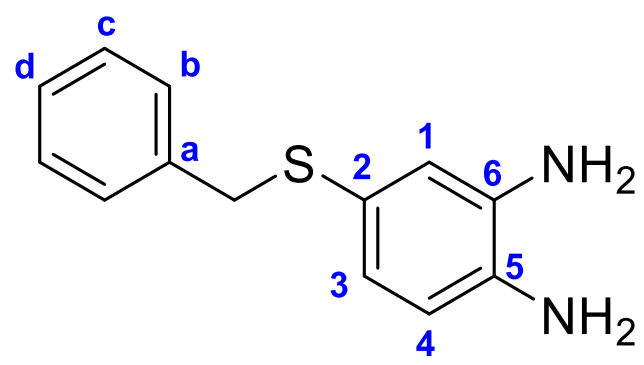

4c

Compound **4c** was synthesized following the general procedure **B**, using **3c** (400 mg, 1.5 mmol), Pd/C (8 mg, 0.07 mmol) in EtOH (15 mL), followed by the addition of hydrazine monohydrate (4.5 mL, 15 mmol). The resulting brown oil was not further purified (340 mg, 96% yield).

^{1}H NMR (400 MHz, $CDCl_3$) δ 7.31 – 7.16 (m, 5H), 6.76 – 6.66 (m, 2H), 6.58 (d, *J* = 7.9 Hz, 1H), 3.97 (s, 2H, CH_2), 3.37 (s, 4H, 2 x NH_2). **^{13}C NMR** (101 MHz, $CDCl_3$) δ 138.5, 135.0, 134.7, 129.0 (Cc), 128.4 (Cd), 127.0 (Hd), 125.3, 125.0 (H3), 121.2 (H4), 117.0 (H1), 41.5 (CH_2). **IR** (cm^{-1}): 3329, 3059, 3026, 2969, 1617, 1579, 1501, 1452, 1368, 1283, 1237, 1199, 1154, 1069, 1027, 912, 808. **MS** (CI): m/z = 231.19 ($[M+H]^+$).

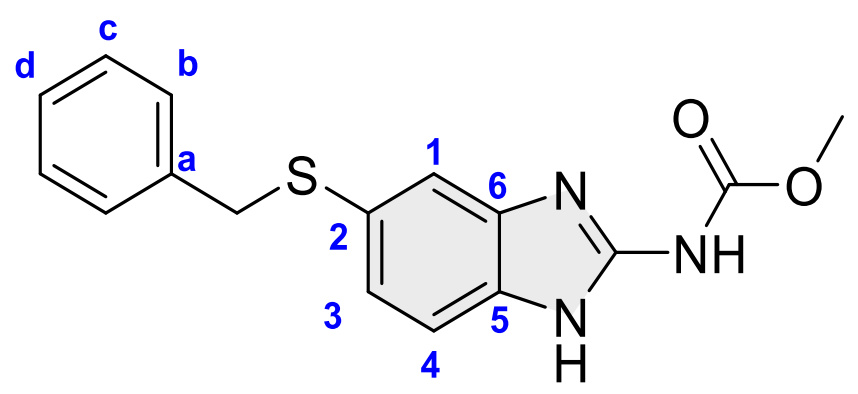

5c

Compound **5c** was prepared following the general procedure **C**, using **4c** (340 mg, 1.5 mmol), 1,3-bis(methoxycarbonyl)-2-methyl-2-thiopseudourea (310 mg, 1.5 mmol) and acetic acid (0.1 mL, 1.6 mmol) in EtOH/H_2O (5 mL : 5 mL). The crude residue was purified by flash column chromatography using a 12 g silica column, and a gradient of MeOH in DCM (0 to 5 %). Eluting at 2.5% MeOH in DCM, the product formed a white precipitate in the tubes of the column chromatography after a few minutes. Removing the solvent afforded a white solid (270 mg, 57% yield).

^{1}H NMR (400 MHz, $(CD_3)_2SO$) δ 11.66 (br s, 2H, 2 x NH), 7.39 (d, *J* = 1.7 Hz, 1H, H1), 7.34 – 7.30 (m, 1H), 7.29 – 7.15 (m, 5H), 7.08 (dd, *J* = 8.3, 1.8 Hz, 1H, H4), 4.13 (s, 2H, CH_2), 3.75 (s, 3H, CH_3). **^{13}C NMR** (101 MHz, $(CD_3)_2SO$) δ 155.2 (CO), 148.3, 138.6, 129.2 (Cc), 128.7 (Cd), 127.3, 126.9, 124.7 (C3), 116.8 (C1), 114.5, 52.9 (CH_3), 39.1 (CH_2). **IR** (cm^{-1}): 3360, 2952, 2673, 1706, 1637, 1592, 1522, 1493, 1460, 1443, 1424, 1322, 1272, 1226, 1195, 1099, 922. **HRMS** (APCI): Found, m/z = 313.0888 ($[M+H]^+$). Calculated for $C_{16}H_{15}N_3O_2S$, 314.0962. **Elemental analysis**: Calculated for $C_{16}H_{15}N_3O_2S$ = C, 61.32; H, 4.82; N, 13.41. Found = C, 60.85; H, 4.85; N, 13.26.

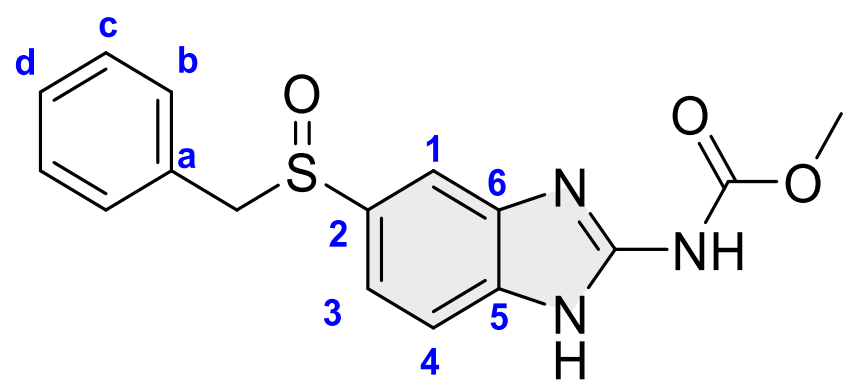

6c

Compound **6c** was prepared following the general procedure **D** with **5c** (62 mg, 0.2 mmol), H_2O_2 aqueous solution (0.04 mL) and phenol (0.2 mL). The residue obtained after work-up was purified by flash column chromatography using a 4 g silica column, and a gradient of MeOH in DCM (0 to 5%). Eluting at 4% MeOH in DCM, the product was isolated as a white solid (47 mg, 71% yield).

^{1}H NMR (400 MHz, $(CD_3)_2SO$) δ 11.81 (br s, 2H, 2 x NH), 7.55 (d, *J* = 1.7 Hz, 1H, H1), 7.51 (d, *J* = 8.3 Hz, 1H, H4), 7.31 – 7.20 (m, 4H), 7.12 – 7.03 (m, 2H), 4.18 (d, *J* = 12.6 Hz, 1H, CH_2), 4.07 (d, *J* = 12.6 Hz, 1H, CH_2), 3.77 (s, 3H, CH_3). **^{13}C NMR** (101 MHz, $(CD_3)_2SO$) δ 154.4 (CO), 148.6, 135.3, 130.8, 130.3 (Cc), 128.1 (Cd), 127.7, 117.1, 113.3, 110.8, 62.6 (CH_2), 52.6 (CH_3). **IR** (cm^{-1}): 3320, 3028, 2953, 2733, 1701, 1647, 1592, 1526, 1495, 1453, 1429, 1268, 1223, 1198, 1102, 1054, 1030, 865, 803. **HRMS** (APCI): Found, m/z = 330.0912 ($[M+H]^+$). Calculated for $C_{16}H_{15}N_3O_3S$, 329.0838. **Elemental analysis**: Calculated for $C_{16}H_{15}N_3O_3S$ = C, 58.35; H, 4.59; N, 12.76. Found = C, 57.67; H, 4.69; N, 12.41.

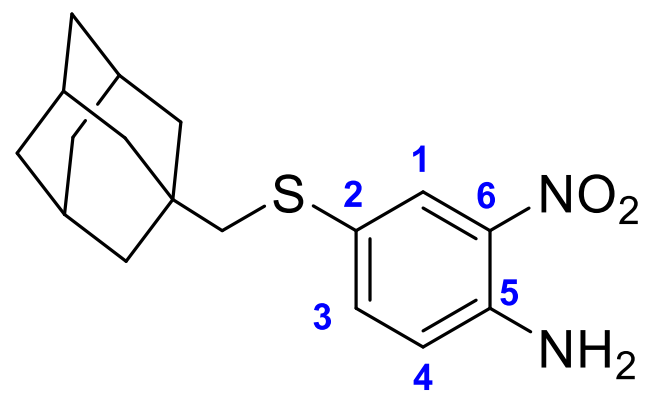

3d

Compound **3d** was synthesized following the general procedure **A**, using in the first step 2-nitro-4-thiocyanatoaniline (500 mg, 2.5 mmol) and 12.5 mL of 1 M KOH in EtOH solution. The crude residue (600 mg) was taken to the second step with K_2CO_3 (1.725 g, 12.5 mmol) and 1-(bromomethyl)adamantane (860 mg, 3.75 mmol) in DMF (5 mL). Purification was performed by flash column chromatography using a 20 g silica column, and a gradient of EtOAc in cyclohexane (0 to 90%). Eluting at 20% EtOAc in cyclohexane, the product was isolated as a red solid (283 mg, 36% yield).

^{1}H NMR (400 MHz, $CDCl_3$) δ 8.14 (d, *J* = 2.2 Hz, 1H, H1), 7.39 (dd, *J* = 8.7, 2.2 Hz, 1H, H3), 6.74 (d, *J* = 8.7 Hz, 1H, H4), 6.06 (br s, 2H, NH_2), 2.70 (s, 2H, CH_2), 1.98 (p, *J* = 3.0 Hz, 3H, $C_{10}H_{15}$), 1.74 – 1.57 (m, 12H, $C_{10}H_{15}$). **^{13}C NMR** (101 MHz, $CDCl_3$) δ 143.3 (C5), 138.9 (C3), 132.3 (C6), 127.6 (C1), 126.2 (C2), 119.4 (C4), 50.9, 42.0, 36.9, 34.5, 28.6. **IR** (cm^{-1}): 3484, 3359, 2900, 2846, 1624, 1579, 1551, 1498, 1449, 1398, 1356, 1336, 1314, 1280, 1240, 1172, 1115, 1080, 900, 815. **MS** (CI): m/z = 319.28 ($[M+H]^+$).

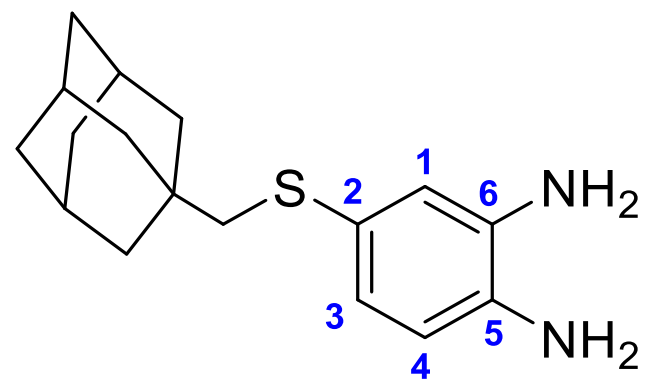

4d

Compound **4d** was synthesized following the general procedure **B**, using **3d** (280 mg, 0.9 mmol), Pd/C (5 mg, 0.045 mmol) in EtOH (10 mL), followed by the addition of hydrazine monohydrate (3 mL, 9 mmol). The resulting brown oil was not further purified (260 mg, 99% yield).

^{1}H NMR (400 MHz, $CDCl_3$) δ 6.82 – 6.74 (m, 2H, H1 and H3), 6.61 (dd, *J* = 8.0, 0.9 Hz, 1H, H4), 3.88 (br s, 1H, NH_2), 3.37 (br s, 3H, NH_2), 2.67 (s, 2H, CH_2), 1.96 (s, 3H, $C_{10}H_{15}$), 1.73 – 1.54 (m, 12H, $C_{10}H_{15}$). **^{13}C NMR** (101 MHz, $CDCl_3$) δ 135.3 (C6), 133.6 (C5), 128.3 (C2), 123.4 (C3), 119.7 (C4), 117.2 (C1), 51.4, 42.0, 37.0, 34.4, 28.7. **IR** (cm^{-1}): 3203, 2899, 2844, 1667, 1579, 1502, 1450, 1360, 1343, 1314, 1279, 1249, 1097, 1016, 805. **MS** (CI): m/z = 289.26 ($[M+H]^+$).

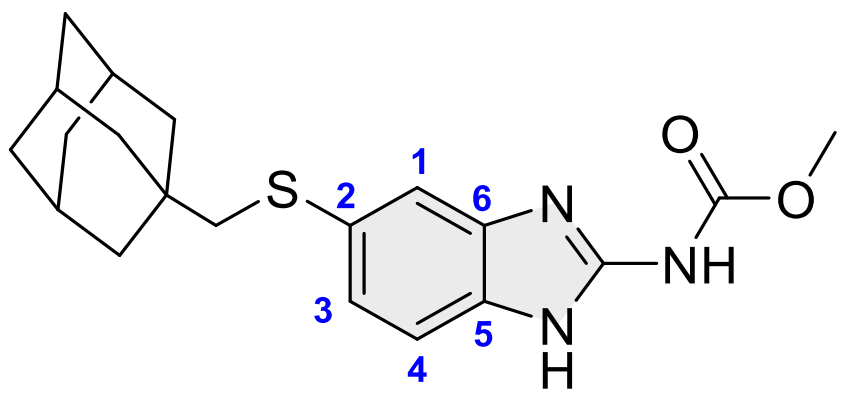

5d

Compound **5d** was prepared following the general procedure **C**, using **4d** (260 mg, 0.9 mmol), 1,3-bis(methoxycarbonyl)-2-methyl-2-thiopseudourea (180 mg, 0.9 mmol) and acetic acid (0.05 mL, 1 mmol) in EtOH/H_2O (5 mL : 5 mL). The crude residue was purified by flash column chromatography using a 12 g silica column, and a gradient of MeOH in DCM (0 to 5%). Eluting at 5% MeOH in DCM, the product was isolated as a white solid (251 mg, 75% yield).

Crystals were obtained by vapor diffusion. In a small vial, **5d** was dissolved in $CHCl_3$ (5 mg in 0.5 mL). It was then set in a larger vial containing acetonitrile (2.5 mL) and left at 20°C for 14 days.

^{1}H NMR (400 MHz, $(CD_3)_2SO$) δ 11.62 (br s, 2H, 2 x NH), 7.43 (d, *J* = 1.7 Hz, 1H, H1), 7.31 (d, *J* = 8.2 Hz, 1H, H4), 7.11 (dd, *J* = 8.3, 1.8 Hz, 1H, H3), 3.75 (s, 3H, CH_3), 2.75 (s, 2H, CH_2), 1.93 (m, 3H, $C_{10}H_{15}$), 1.74 – 1.50 (m, 12H, $C_{10}H_{15}$). **^{13}C NMR** (101 MHz, $(CD_3)_2SO$) δ 154.8 (CO), 147.8, 128.9, 123.6 (C3), 115.1 (C1), 114.2 (C4), 52.5 (CH_3), 49.9 (CH_2), 41.3 (CH_2, $C_{10}H_{15}$), 36.3 (CH_2, $C_{10}H_{15}$), 33.9 (Cq, $C_{10}H_{15}$), 27.9 (CH, $C_{10}H_{15}$). **IR** (cm^{-1}): 3418, 3397, 2894, 2846, 1710, 1654, 1603, 1525, 1474, 1450, 1430, 1359, 1343, 1316, 1295, 1284, 1235, 1193, 1096, 929, 853. **HRMS** (APCI): Found, m/z = 372.1749 ($[M+H]^+$). Calculated for $C_{20}H_{25}FeN_3O_2S$, 371.1667. **Elemental analysis**: Calculated for $C_{20}H_{25}N_3O_2S$ = C, 64.66; H, 6.78; N, 11.31. Found = C, 63.93 ; H, 6.96; N, 11.16.

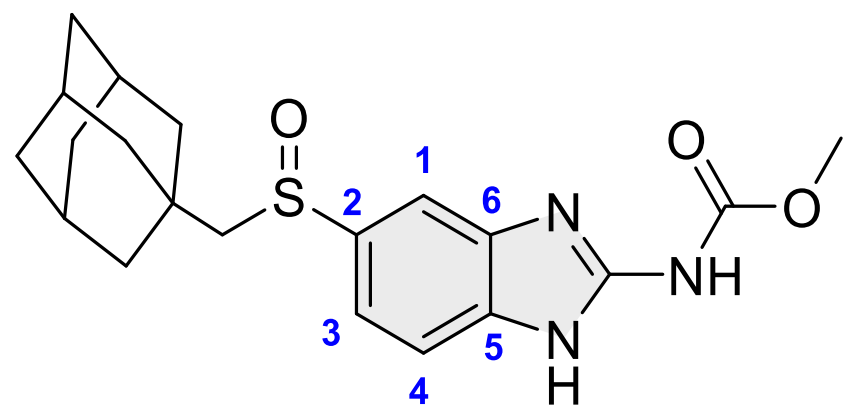

6d

Compound **6d** was prepared following the general procedure **D** with **6c** (75 mg, 0.2 mmol), H_2O_2 aqueous solution (0.04 mL) and phenol (0.2 mL). The residue obtained after work-up was purified by flash column chromatography using a 4 g silica column, and a gradient of MeOH in DCM (0 to 5%). Eluting at 4% MeOH in DCM, the product was isolated as a white solid (60 mg, 77% yield).

^{1}H NMR (400 MHz, $(CD_3)_2SO$) δ 11.82 (s, 2H, 2 x NH), 7.69 (d, *J* = 1.7 Hz, 1H, H1), 7.54 (d, *J* = 8.3 Hz, 1H, H4), 7.29 (dd, *J* = 8.2, 1.7 Hz, 1H, H3), 3.77 (s, 3H, CH_3), 2.59 (d, *J* = 13.6 Hz, 1H, CH_2), 2.50 (m, 1H, CH_2), 2.01 – 1.94 (m, 3H, $C_{10}H_{15}$), 1.82 – 1.60 (m, 12H, $C_{10}H_{15}$). **^{13}C NMR** (101 MHz, $(CD_3)_2SO$) δ 154.5 (CO), 148.6, 137.8, 137.4, 132.3, 116.5 (C3), 73.3 (CH_2), 52.6 (CH_3), 41.8 (CH_2, $C_{10}H_{15}$), 36.1 (CH_2, $C_{10}H_{15}$), 33.4 (Cq, $C_{10}H_{15}$), 27.8 (CH, $C_{10}H_{15}$). **IR** (cm^{-1}): 3304, 2899, 2845, 1729, 1706, 1637, 1590, 1522, 1451, 1273, 1224, 1199, 1098, 1054, 1030, 807. **HRMS** (APCI): Found, m/z = 388.1700 ($[M+H]^+$). Calculated for $C_{20}H_{25}N_3O_3S$, 387.1626 g/mol. **Elemental analysis**: Calculated for $C_{20}H_{25}N_3O_3S$ = C, 61.99; H, 6.50; N, 10.84. Found = C, 60.78; H, 6.70; N, 10.65.

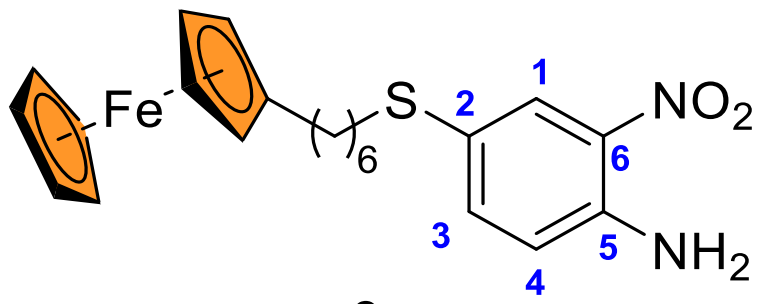

3e

Compound **3e** was prepared following the general procedure **A**, using in the first step 2-nitro-4-thiocyanatoaniline (10 mmol, 1950 mg) and 45 mL of 1 M KOH in EtOH solution. A red residue (2.1 g) is obtained, and taken to the second step without further purification. Part of the residue obtained in the first step (200 mg), K_2CO_3 (2.5 mmol, 345 mg) and **2e** (0.6 mmol, 209 mg) are dissolved in DMF (5 mL). The crude mixture was deposited on silica and purified by column flash chromatography using a 20 g silica column and a gradient of EtOAc in heptane (0 to 30%). Eluting at 20% EtOAc in heptane the product was obtained as a red oil (232 mg, 88% yield).

^{1}H NMR (400 MHz, $CDCl_3$) δ 8.16 (d, *J* = 2.2 Hz, 1H, H1), 7.39 (dd, *J* = 8.7, 2.2 Hz, 1H, H3), 6.75 (d, *J* = 8.7, 1H, H4), 6.08 (br s, 2H, NH_2), 4.09 (s, 5H, C_5H_5), 4.05 (m, 4H, C_5H_4), 2.83 (t, *J* = 7.3 Hz, 2H, CH_2), 2.34 – 2.25 (m, 2H, CH_2), 1.65 – 1.23 (m, 6H, CH_2). **^{13}C NMR** (101 MHz, $CDCl_3$) δ 143.7 (C5), 139.2 (C3), 132.3 (C6), 128.5 (C1), 124.0 (C2), 119.5 (C6), 89.6 (C_5H_4), 68.7 (C_5H_5), 68.3 (C_5H_4), 67.2 (C_5H_4), 35.7 (CH_2), 31.1 (CH_2), 29.6 (CH_2), 29.2 (CH_2), 29.2 (CH_2), 28.6 (CH_2). **IR** (cm^{-1}): 3486, 3369, 3090, 2927, 2852, 1623, 1586, 1551, 1506, 1465, 1402, 1352, 1333, 1275, 1247, 1167, 1121, 1104, 999, 898, 818. **MS** (ESI): m/z = 439.1 ($[M+H]^+$).

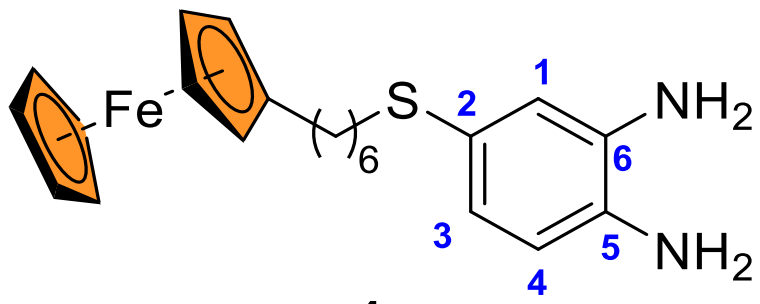

4e

Compound **4e** was synthesized following the general procedure **B**, using **3e** (232 mg, 0.52 mmol), Pd/C (3 mg, 0.028 mmol) in EtOH (7.5 mL), followed by the addition of hydrazine monohydrate (1.6 mL, 5.2 mmol). The resulting dark red oil was not further purified (214 mg, 99% yield).

^{1}H NMR (400 MHz, $CDCl_3$) δ 6.82 – 6.74 (m, 2H, H1 and H3), 6.62 (d, *J* = 8.4 Hz, 1H, H4), 4.08 (s, 5H, C_5H_5), 4.05 – 4.02 (m, 4H, C_5H_4), 3.47 (br s, 4H, 2 x NH_2), 2.79 (t, *J* = 7.4 Hz, 2H, CH_2), 2.29 (t, *J* = 7.6 Hz, 2H, CH_2), 1.64 – 1.20 (m, 8H, CH_2). **^{13}C NMR** (101 MHz, $CDCl_3$) δ 135.1 (C6), 134.0 (C5), 125.8 (C2), 123.8 (C3), 120.1 (C4), 117.0 (C1), 89.4 (C_5H_4), 68.5 (C_5H_5), 68.1 (C_5H_4), 67.0 (C_5H_4), 35.9 (CH_2), 31.0 (CH_2), 29.5 (CH_2), 29.4 (CH_2), 29.2 (CH_2), 28.6 (CH_2). **IR** (cm^{-1}): 3335, 3090, 2926, 2852, 1618, 1579, 1501, 1421, 1279, 1241, 1104, 1039, 1021, 999, 914, 815. **MS** (ESI): m/z = 205.1 ($[M+2H]^{2+}$).

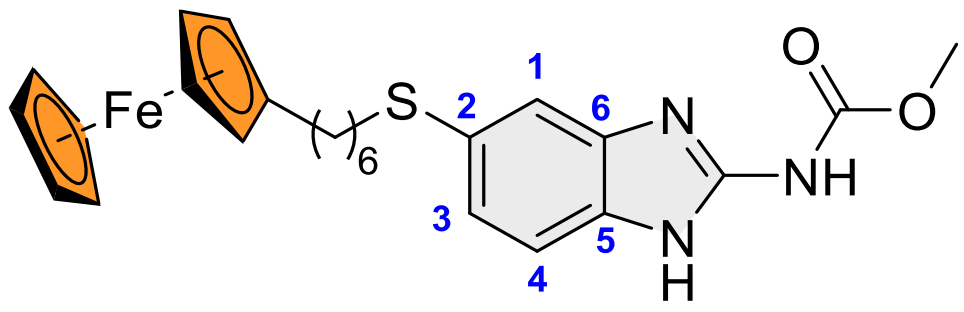

5e

Compound **5e** was prepared following the general procedure **C**, using **4e** (214 mg, 0.5 mmol), 1,3-bis(methoxycarbonyl)-2-methyl-2-thiopseudourea (103 mg, 0.5 mmol) and acetic acid (0.03 mL, 0.55 mmol) in EtOH/H_2O (5 mL : 5 mL). The crude residue was purified by flash column chromatography using a 20 g silica column and a gradient of MeOH in DCM (0 to 5%). Eluting at 5% MeOH, the product was isolated as a light orange solid (153 mg, 62% yield).

^{1}H NMR (400 MHz, $(CD_3)_2SO$) δ 11.61 (s, 2H, 2 x NH), 7.43 (d, *J* = 1.7 Hz, 1H, H1), 7.34 (dd, *J* = 8.2, 0.6 Hz, 1H, H4), 7.10 (dd, *J* = 8.2, 1.7 Hz, 1H, H3), 4.06 (s, 5H), 4.03 (t, *J* = 1.8 Hz, 2H, C_5H_4), 4.00 (t, *J* = 1.8 Hz, 2H, C_5H_4), 3.75 (s, 3H, CH_3), 2.87 (t, *J* = 7.2 Hz, 2H, CH_2), 2.24 (t, *J* = 8.0 Hz, 2H, CH_2), 1.58 – 1.47 (m, 2H, CH_2), 1.45 – 1.33 (m, 4H, CH_2), 1.31 – 1.21 (m, 2H, CH_2). **^{13}C NMR** (101 MHz, $(CD_3)_2SO$) δ 154.8 (CO), 147.8, 126.8, 124.0 (H3), 115.8 (H1), 114.1 (H4), 88.8 (C_5H_4), 68.2 (C_5H_5), 67.7 (C_5H_4), 66.7 (C_5H_4), 52.5 (CH_3), 34.6 (CH_2), 30.3 (CH_2), 28.8 (CH_2), 28.7 (CH_2), 28.4 (CH_2), 27.8 (CH_2). **IR** (cm^{-1}): 3324, 3090, 2926, 2851, 2690, 1707, 1636, 1590, 1522, 1443, 1325, 1271, 1225, 1196, 1104, 1021, 1000, 958, 924, 864, 806. **HRMS** (APCI): Found, m/z = 490.1453 ($[M+H]^+$). Calculated for $C_{25}H_{29}FeN_3O_2S$, 489.1377. **Elemental analysis**: Calculated for $C_{25}H_{29}FeN_3O_2S$ = C, 61.10; H, 4.95; N, 8.55. Found = C, 61.17; H, 5.93; N, 8.60.

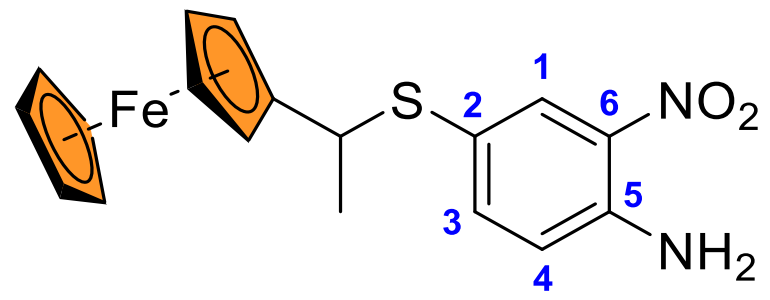

3f

Compound **3f** was prepared following the general procedure **A**, using in the first step 2-nitro-4-thiocyanatoaniline (10 mmol, 1950 mg) and 45 mL of 1 M KOH in EtOH solution. A red residue (2.1 g) is obtained, and taken to the second step without further purification. Part of the residue obtained in the first step (200 mg), K_2CO_3 (5 mmol, 690 mg) and **2f** (1 mmol, 400 mg) are dissolved in DMF (5 mL). The crude mixture was deposited on silica and purified by flash column chromatography using a 20 g silica column and a gradient of EtOAc in heptane (0 to 30%). Eluting at 20% EtOAc in heptane the product was obtained as a dark red solid (305 mg, 79% yield).

^{1}H NMR (400 MHz, $CDCl_3$) δ 8.13 (d, *J* = 2.1 Hz, 1H, H1), 7.22 (dd, *J* = 8.6, 2.1 Hz, 1H, H3), 6.67 (d, *J* = 8.6 Hz, 1H, H4), 6.11 (br s, 2H, NH_2), 4.14 (s, 5H, C_5H_5), 4.12 – 4.05 (m, 3H), 4.02 – 3.95 (m, 2H), 1.61 (d, *J* = 6.9 Hz, 3H, CH_3). **^{13}C NMR** (101 MHz, $CDCl_3$) δ 144.3 (C5), 142.5 (C3), 132.8 (C1), 131.9 (C6), 121.6 (C2), 118.7 (C4), 90.5 (C_5H_4), 68.7 (C_5H_5), 68.1 (C_5H_4), 67.8 (C_5H_4), 67.7 (C_5H_4), 65.8 (C_5H_4), 44.7 (CH), 20.8 (CH_3). **IR** (cm^{-1}): 3485, 3369, 3092, 2972, 2926, 1621, 1551, 1503, 1467, 1402, 1351, 1334, 1274, 1247, 1166, 1121, 1105, 1077, 1023, 1000, 898, 821. **MS** (ESI): m/z = 383.0 ($[M+H]^+$).

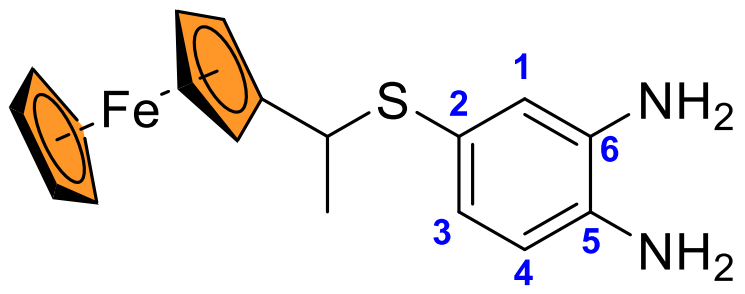

4f

Compound **4f** was synthesized following the general procedure **B**, using **3f** (305 mg, 0.79 mmol), Pd/C (4 mg, 0.04 mmol) in EtOH (10 mL), followed by the addition of hydrazine monohydrate (2.4 mL, 7.9 mmol). The resulting dark orange oil was not further purified (300 mg, 99% yield).

^{1}H NMR (400 MHz, $CDCl_3$) δ 6.75 (dd, *J* = 7.9, 2.0 Hz, 1H, H3), 6.71 (d, *J* = 1.9 Hz, 1H, H1), 6.58 (d, *J* = 7.9 Hz, 1H, H4), 4.13 (m, 6H, C_5H_5 and C_5H_4), 4.11 – 4.07 (m, 2H, C_5H_4), 4.04 – 3.98 (m, 1H, C_5H_4), 3.94 (q, *J* = 6.9 Hz, 1H, CH), 3.61 – 3.15 (br s, 4H, 2 x NH_2), 1.57 (d, *J* = 6.9 Hz, 3H, CH_3). **^{13}C NMR** (101 MHz, $CDCl_3$) δ 135.2, 134.5, 127.4 (C3), 124.1 (C2), 123.4 (C4), 116.5 (C1), 91.2 (C_5H_4), 68.6 (C_5H_5), 68.2 (C_5H_4), 67.6 (C_5H_4), 67.4 (C_5H_4), 66.0 (C_5H_4), 44.7 (CH), 21.0 (CH_3). **IR** (cm^{-1}): 3337, 3090, 2970, 2924, 2865, 1617, 1578, 1500, 1444, 1418, 1368, 1280, 1241, 1180, 1152, 1104, 1023, 1000, 906, 816. **MS** (ESI): m/z = 352.1 (M^+).

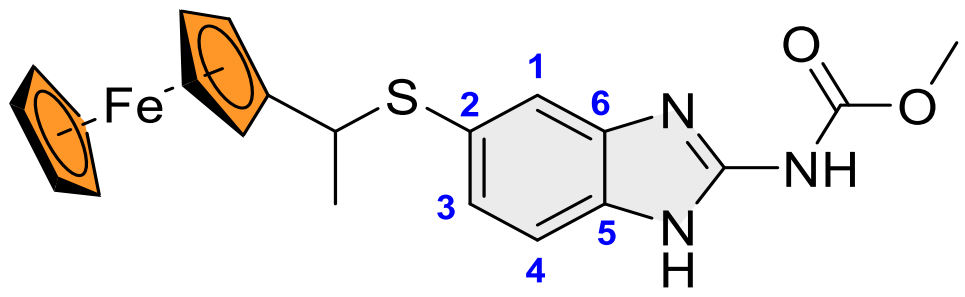

5f

Compound **5f** was prepared following the general procedure **C**, using **4f** (305 mg, 0.85 mmol), 1,3-bis(methoxycarbonyl)-2-methyl-2-thiopseudourea (178 mg, 0.85 mmol) and acetic acid (0.05 mL, 0.94 mmol) in EtOH/H_2O (5 mL : 5 mL). The crude residue was purified by column chromatography using a 20 g silica colum and a gradient of MeOH in DCM (0 to 5%). Eluting at 5% MeOH, the product was isolated as a light orange solid (367 mg, 97% yield).

^{1}H NMR (400 MHz, $(CD_3)_2SO$) δ 11.63 (br s, 2H, 2 x NH), 7.40 (d, *J* = 1.8 Hz, 1H, H1), 7.33 (d, *J* = 8.2 Hz, 1H, H4), 7.08 (dd, *J* = 8.2, 1.7 Hz, 1H, H3), 4.19 – 4.05 (m, 9H, C_5H_5 and C_5H_4 and CH), 4.01 – 3.96 (m, 1H, C_5H_4), 3.75 (s, 3H, CH_3), 1.54 (d, *J* = 6.9 Hz, 3H, CH_3). **^{13}C NMR** (101 MHz, $(CD_3)_2SO$) δ 154.8 (CO), 147.9, 127.2 (C3), 125.5, 118.9 (C1), 113.5 (C4), 90.6 (C_5H_4), 68.5 (C_5H_5), 67.8 (C_5H_4), 67.3 (C_5H_4), 67.1 (C_5H_4), 65.6 (C_5H_4), 52.5 (CH_3), 44.1 (CH), 20.7 (CH_3). **IR** (cm^{-1}): 3322, 3091, 2953, 2678, 1707, 1637, 1589, 1523, 1459, 1354, 1327, 1272, 1225, 1197, 1121, 1104, 1022, 1000, 958, 923, 863, 809. **HRMS** (APCI): Found, m/z = 434.0820 ($[M+H]^+$). Calculated for $C_{21}H_{21}FeN_3O_2S$, 433.0751. **Elemental analysis**: Calculated for $C_{21}H_{21}FeN_3O_2S$ = C, 57.94; H, 4.86; N, 9.65. Found = C, 57.78; H, 4.79; N, 9.81.

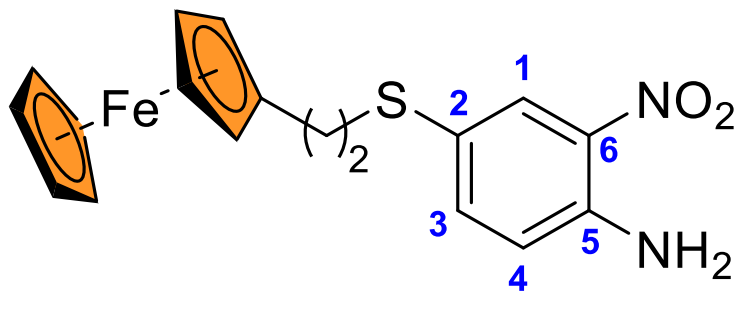

3g

Compound **3g** was prepared following the general procedure **A**, using in the first step 2-nitro-4-thiocyanatoaniline (100 mg, 0.5 mmol) and 2.5 mL of 1 M KOH in EtOH solution. The red residue obtained (86 mg) was taken to the second step with K_2CO_3 (345 mg, 2.5 mmol) and **2g** (150 mg, 0.5 mmol) in DMF (3 mL). Purification was performed by flash column chromatography using a 20 g silica column and a gradient of EtOAc in cyclohexane (0 to 50 %). Eluting at 15% EtOAc in cyclohexane, the product was isolated as a red oil (190 mg, 98% yield).

^{1}H NMR (400 MHz, $CDCl_3$) δ 8.17 (d, *J* = 2.2 Hz, 1H, H1), 7.39 (dd, *J* = 8.7, 2.2 Hz, 1H, H3), 6.77 (d, *J* = 8.7 Hz, 1H, H4), 6.13 (br s, 2H, NH_2), 4.14 – 4.03 (m, 9H, C_5H_5 and C_5H_4), 2.97 (t, *J* = 7.6 Hz, 2H, CH_2), 2.62 (t, *J* = 8.0 Hz, 2H, CH_2). **^{13}C NMR** (101 MHz, $CDCl_3$) δ 144.1 (C5), 139.5 (C3), 132.5 (C6), 128.8 (C1), 123.8 (C2), 119.9 (C4), 87.0 (C_5H_4), 69.0 (C_5H_5), 68.5 (C_5H_4), 67.9 (C_5H_4), 36.8 (CH_2), 30.2 (CH_2). **IR** (cm^{-1}): 3485, 3368, 3090, 2924, 1623, 1586, 1551, 1503, 1468, 1402, 1352, 1333, 1275, 1247, 1168, 1121, 1104, 1078, 1040, 1000, 898, 820. **MS** (ESI): m/z = 382.00 ($[M]^+$).

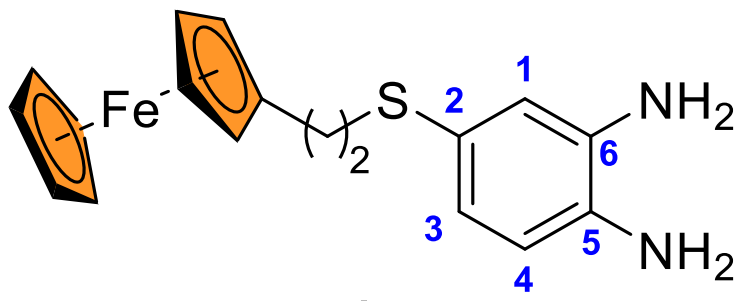

4g

Compound **4g** was prepared following the general procedure **B**, using **3g** (190 mg, 0.5 mmol), Pd/C (3 mg, 0.02 mmol) in EtOH (7.5 mL), followed by the addition of hydrazine monohydrate (5.2 mmol, 1.6 mL). The resulting dark orange oil was not further purified (170 mg, 96 % yield).

^{1}H NMR (400 MHz, $CDCl_3$) δ 6.84 – 6.75 (m, 2H, H1 and H3), 6.64 (d, *J* = 8.5 Hz, 1H, H4), 4.09 – 4.02 (m, 9H, C_5H_4 and C_5H_5), 3.43 (br s, 4H), 2.93 (t, *J* = 7.8 Hz, 2H, CH_2), 2.58 (t, *J* = 7.8 Hz, 2H, CH_2). **^{13}C NMR** (101 MHz, $CDCl_3$) δ 135.3 (C6), 134.3 (C5), 125.6 (C2), 124.1 (C3), 120.3 (C4), 117.2 (C1), 87.4 (C_5H_4), 68.6 (C_5H_5), 68.2 (C_5H_4), 67.4 (C_5H_4), 36.7 (CH_2), 30.0 (CH_2). **IR** (cm^{-1}): 3335, 3089, 2957, 2922, 1618, 1579, 1501, 1420, 1279, 1242, 1103, 1039, 1022, 1000, 914, 810. **MS** (ESI): m/z = 353.0 ($[M+H]^+$).

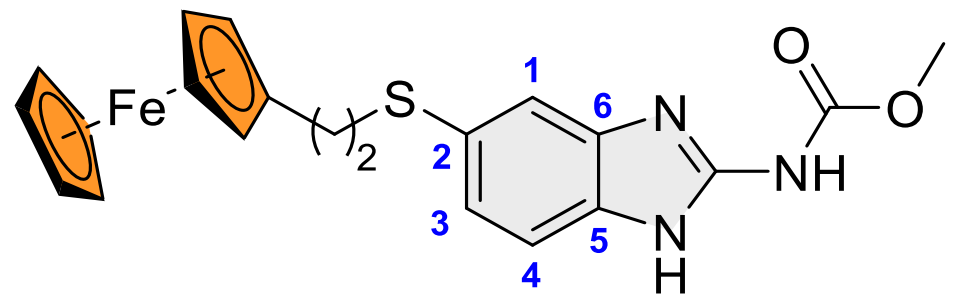

5g

Compound **5g** was prepared following the general procedure **C**, using **4g** (170 mg, 0.5 mmol), 1,3-bis(methoxycarbonyl)-2-methyl-2-thiopseudourea (103 mg, 0.5 mmol) and acetic acid (0.033 mL, 0.55 mmol) in EtOH/H_2O (5 mL : 5 mL). The crude residue was purified by flash column chromatography using a 20 g silica column and a gradient of MeOH in DCM (0 to 5%). Eluting at 5% MeOH, the product was isolated as a yellow solid (204 mg, 94% yield).

^{1}H NMR (400 MHz, $(CD_3)_2SO$) δ 11.55 (br s, 2H, 2 x NH), 7.47 (d, *J* = 1.8 Hz, 1H, H1), 7.36 (d, *J* = 8.2 Hz, 1H, H4), 7.15 (dd, *J* = 8.3, 1.8 Hz, 1H, H3), 4.14 (t, *J* = 1.8 Hz, 2H, C_5H_4), 4.07 (s, 5H, C_5H_5), 4.05 (t, *J* = 1.8 Hz, 2H, C_5H_4), 3.75 (s, 3H, CH_3), 3.04 (t, *J* = 8.0 Hz, 2H, CH_2), 2.57 – 2.51 (m, 2H, CH_2). **^{13}C NMR** (101 MHz, $(CD_3)_2SO$) δ 154.8 (CO), 147.8, 126.6, 124.2 (C4), 115.9 (C1), 114.3 (C3), 86.9 (C_5H_4), 68.3 (C_5H_5), 67.8 (C_5H_4), 67.0 (C_5H_4), 52.5 (CH_3), 35.4 (CH_2), 29.2 (CH_2). **IR** (cm^{-1}): 3324, 3088, 2948, 2672, 1710, 1637, 1592, 1523, 1459, 1356, 1329, 1270, 1225, 1196, 1104, 1001, 923, 864, 807. **HRMS** (APCI): Found, m/z = 434.0820 ($[M+H]^+$). Calculated for $C_{21}H_{21}FeN_3O_2S$, 433.0751. **Elemental analysis**: Calculated for $C_{21}H_{21}FeN_3O_2S$ = C, 57.94; H, 4.86; N, 9.65. Found = C, 57.74; H, 4.65; N, 9.72.

1.5. NMR spectra for characterization

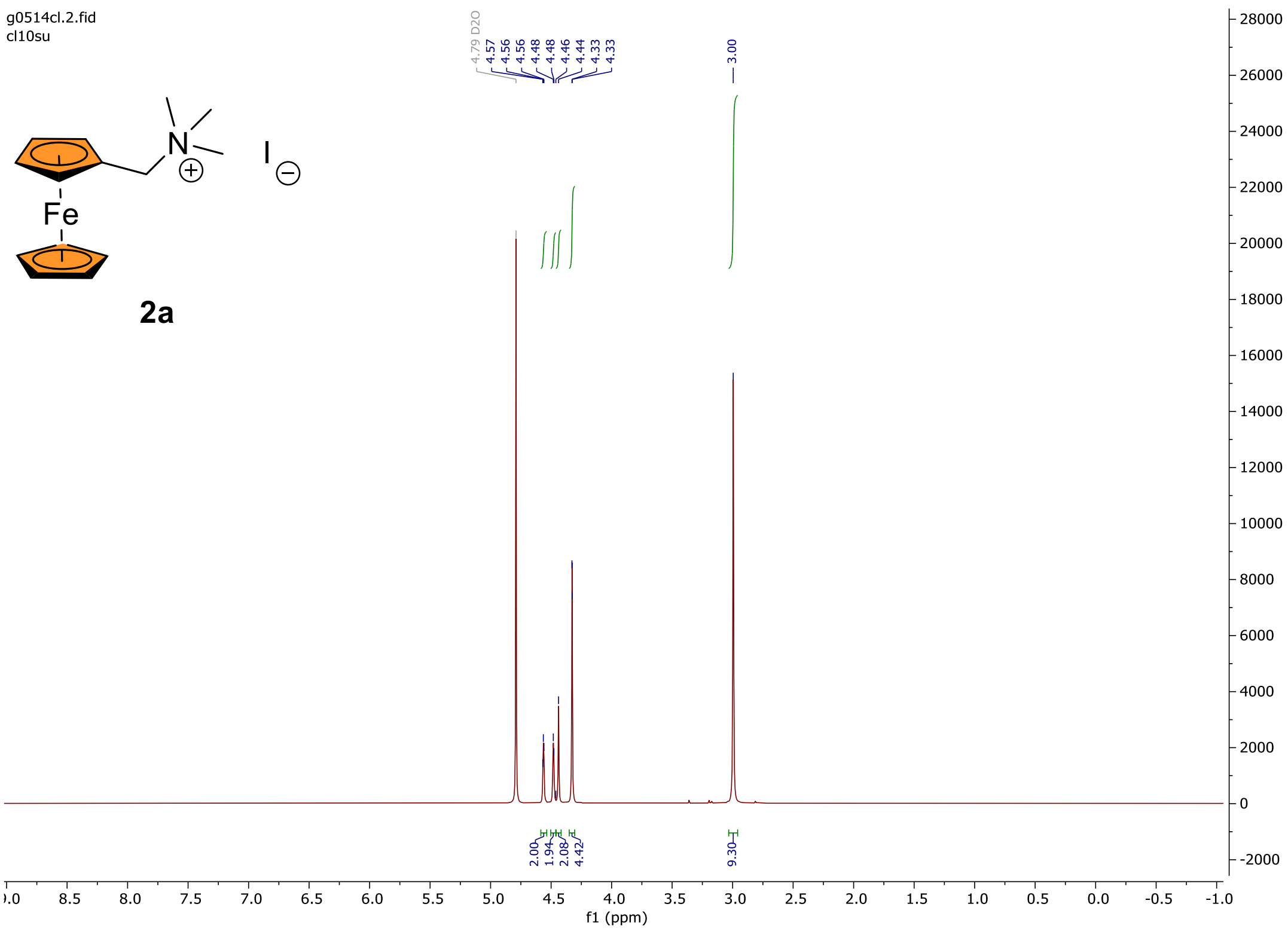

Figure S1. ^{1}H NMR spectrum of **2a** in D_2O.

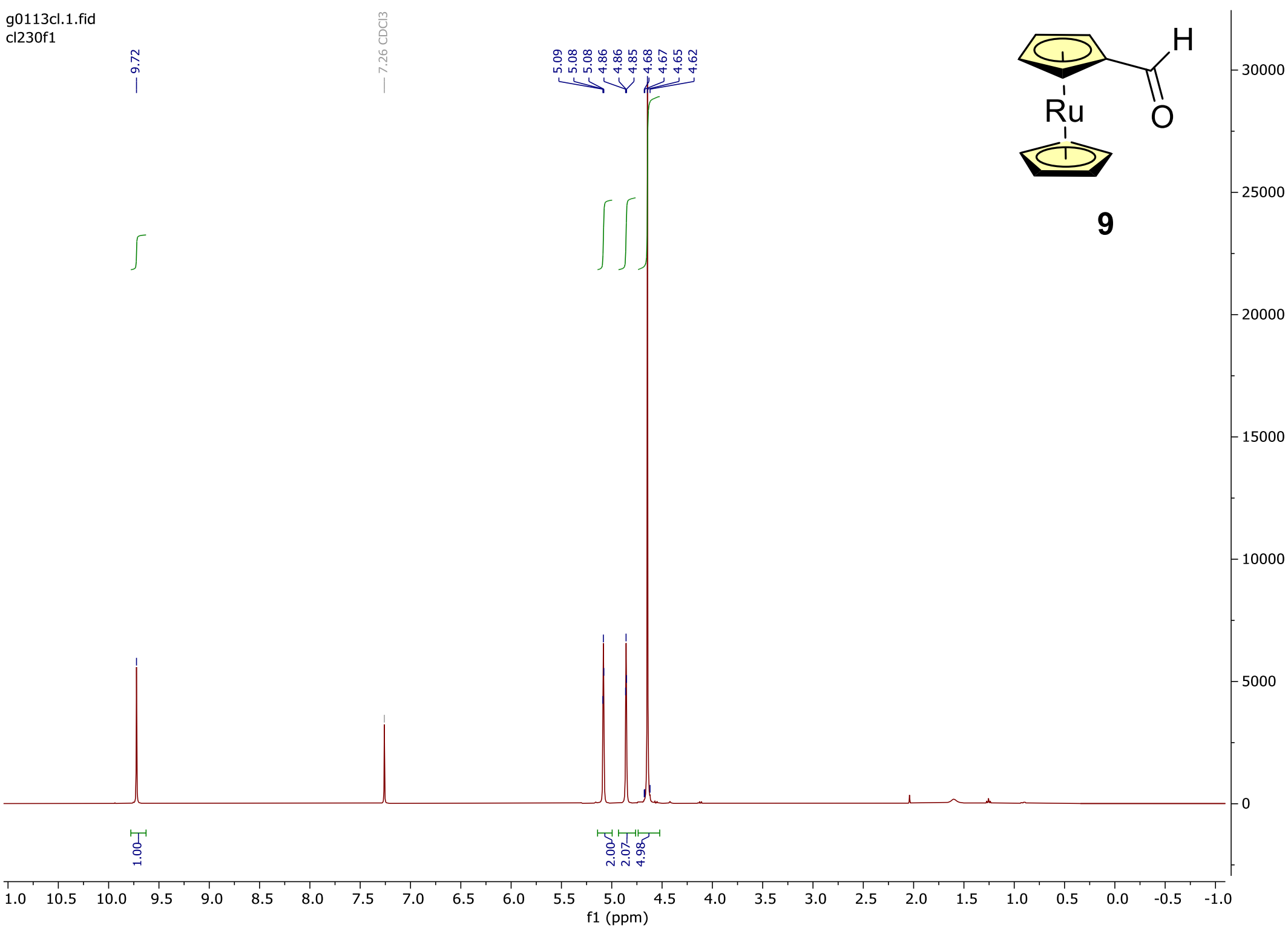

Figure S2. ^{1}H NMR spectrum of **9** in $CDCl_3$.

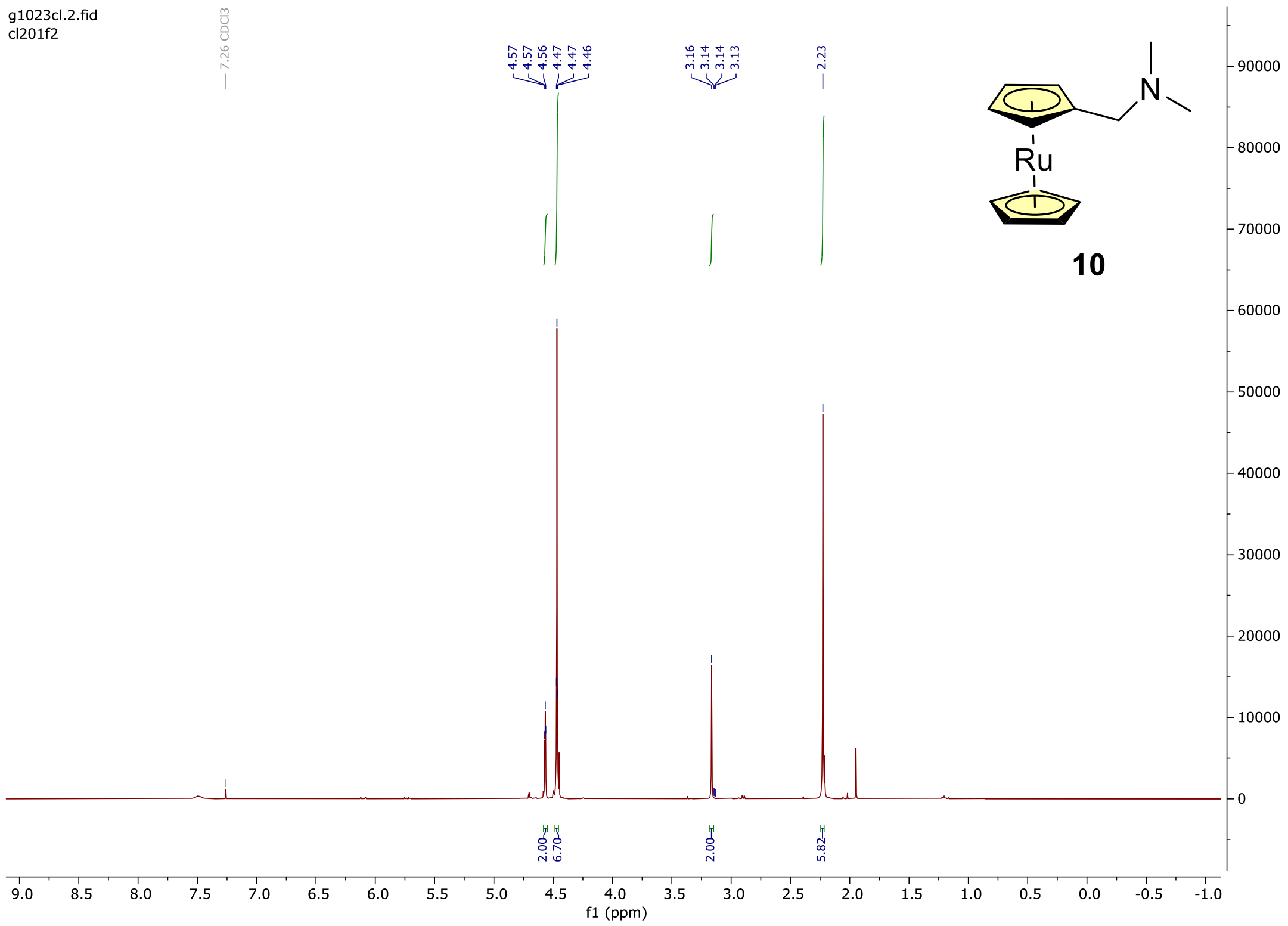

Figure S3. ^{1}H NMR spectrum of **10** in $CDCl_3$.

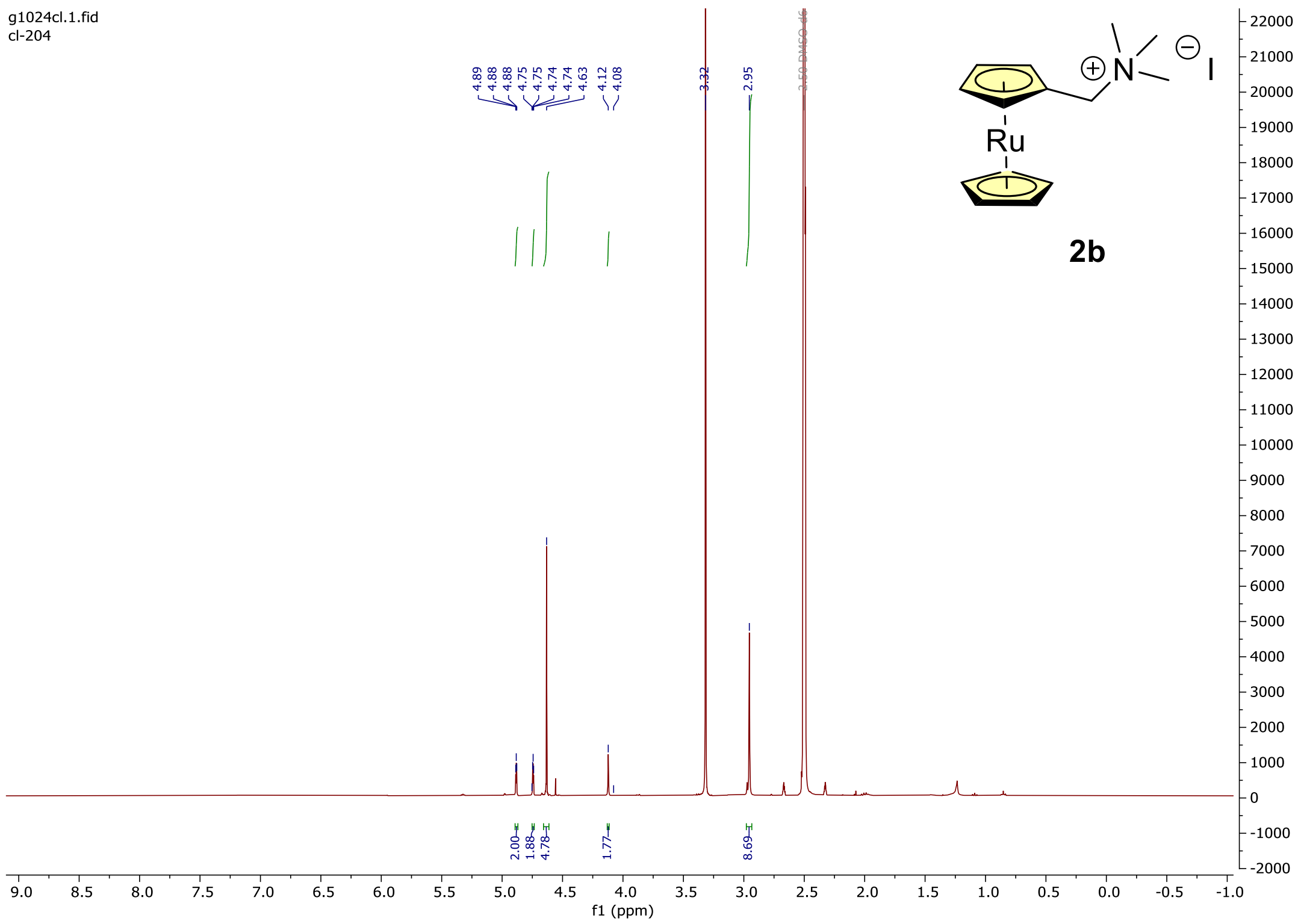

Figure S4. ^{1}H NMR spectrum of **2b** in $(CD_3)_2SO$.

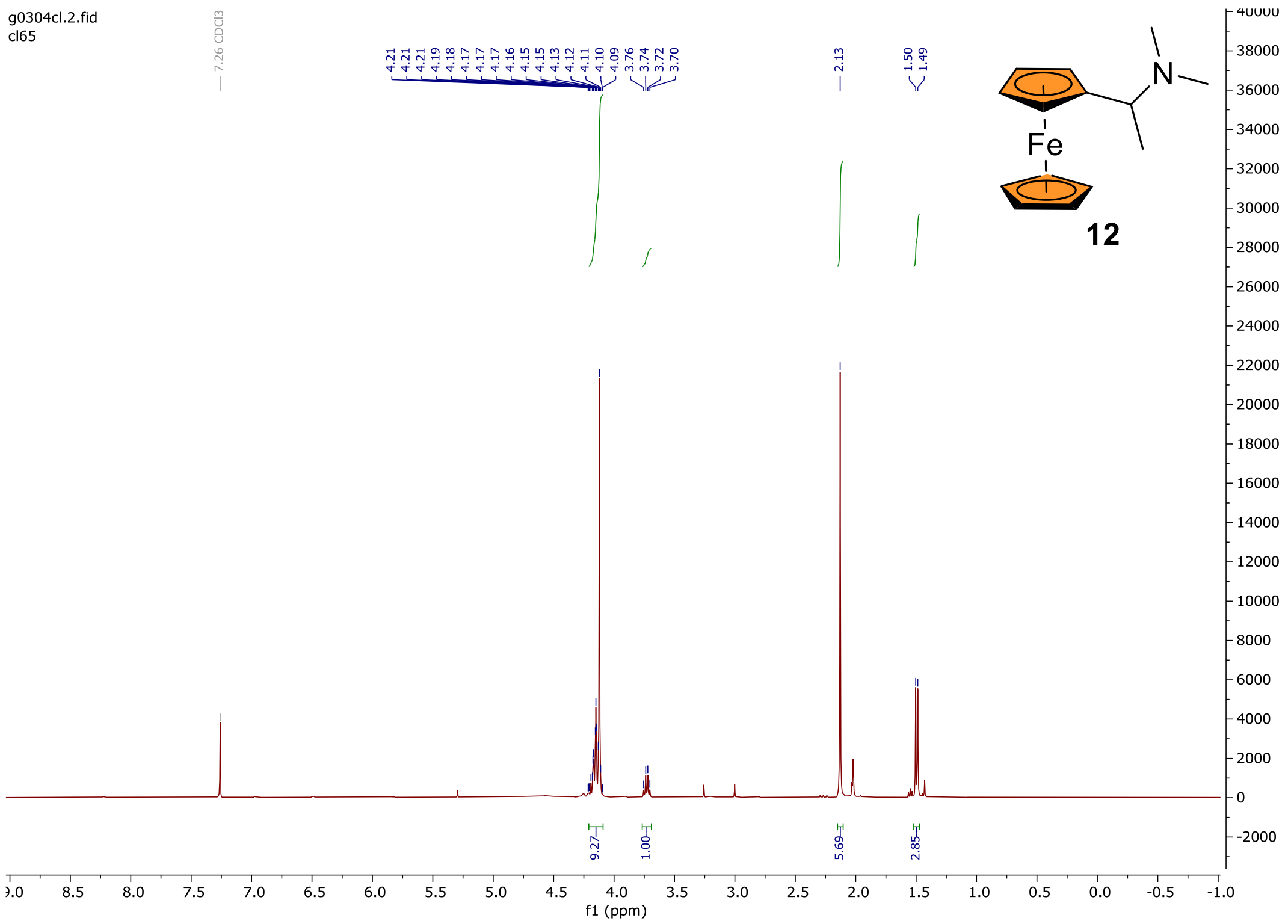

Figure S5. ^{1}H NMR spectrum of **12** in $CDCl_3$.

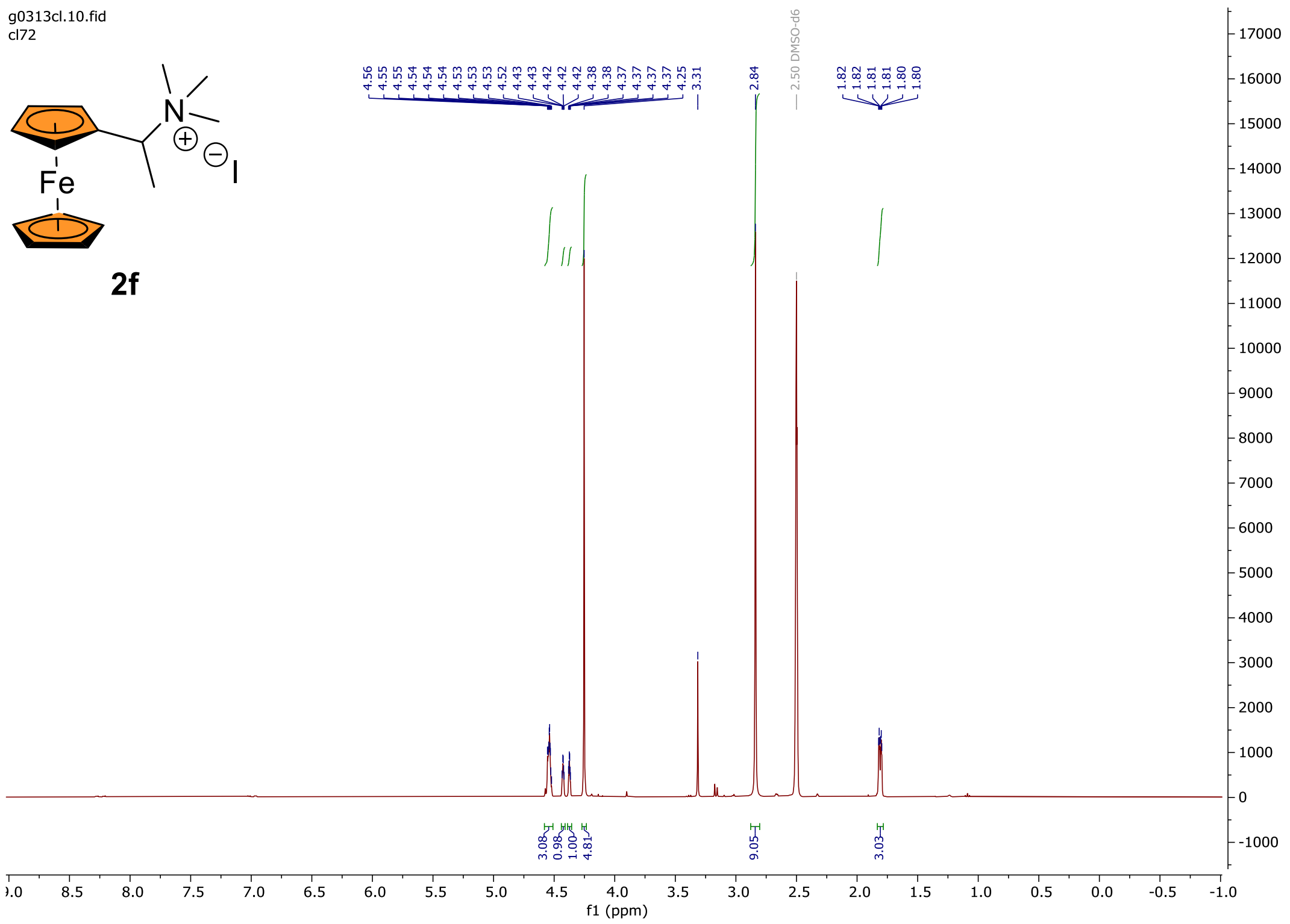

Figure S6. ^{1}H NMR spectrum of **2f** in $(CD_3)_2SO$.

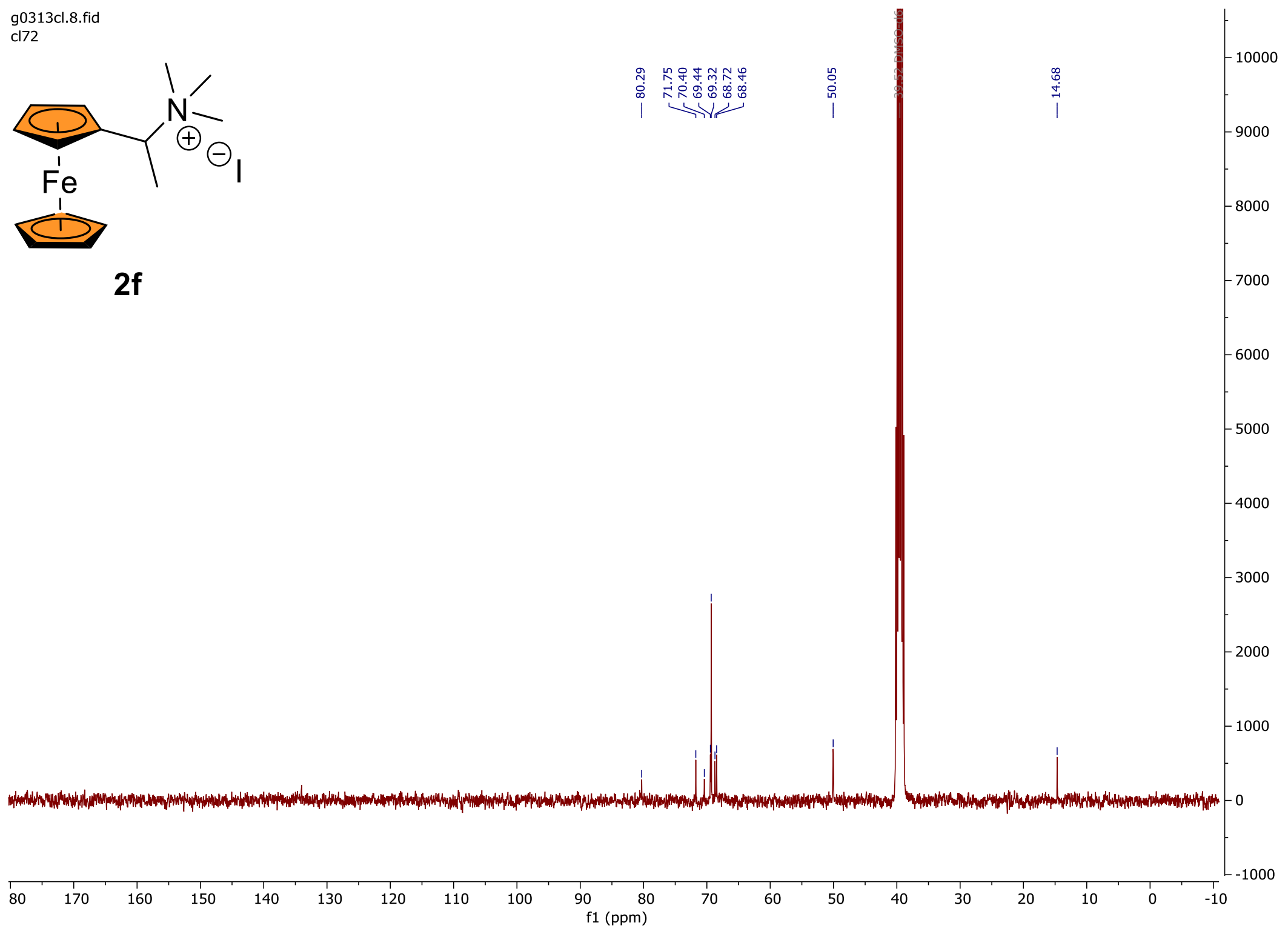

Figure S7. ^{13}C NMR spectrum of **2f** in $(CD_3)_2SO$.

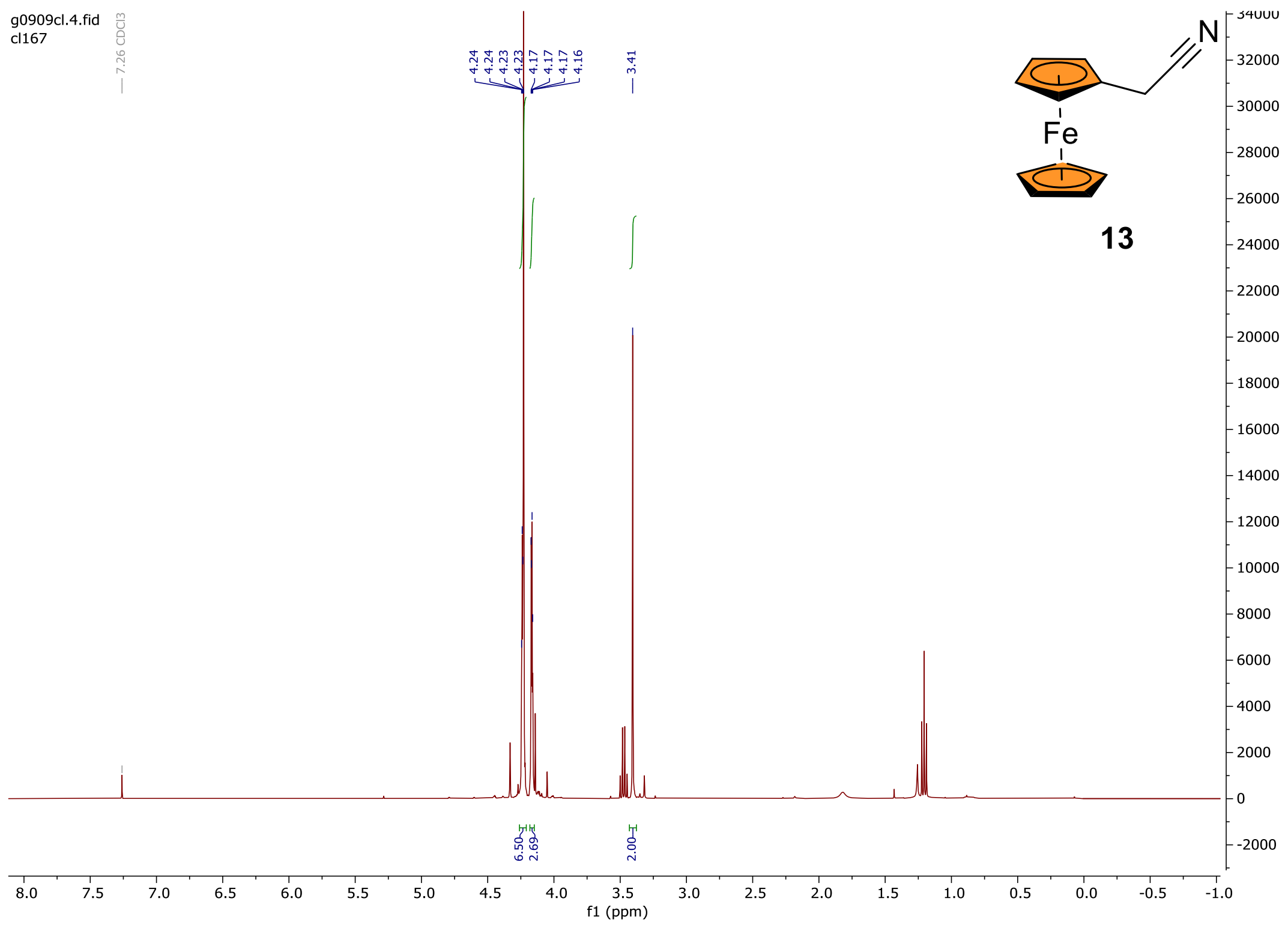

Figure S8. ^{1}H NMR spectrum of **13** in $CDCl_3$.

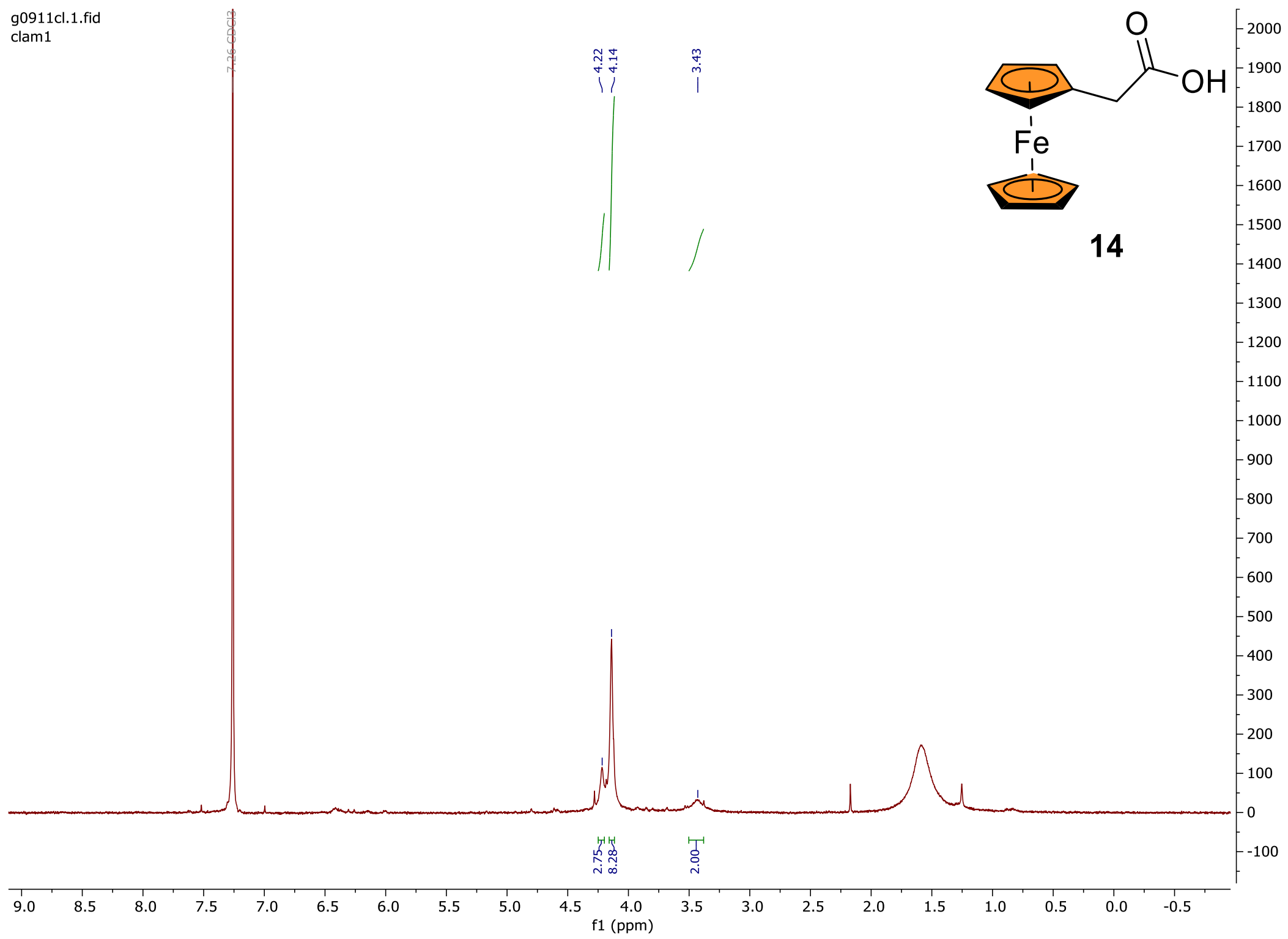

Figure S9. ^{1}H NMR spectrum of **14** in $CDCl_3$.

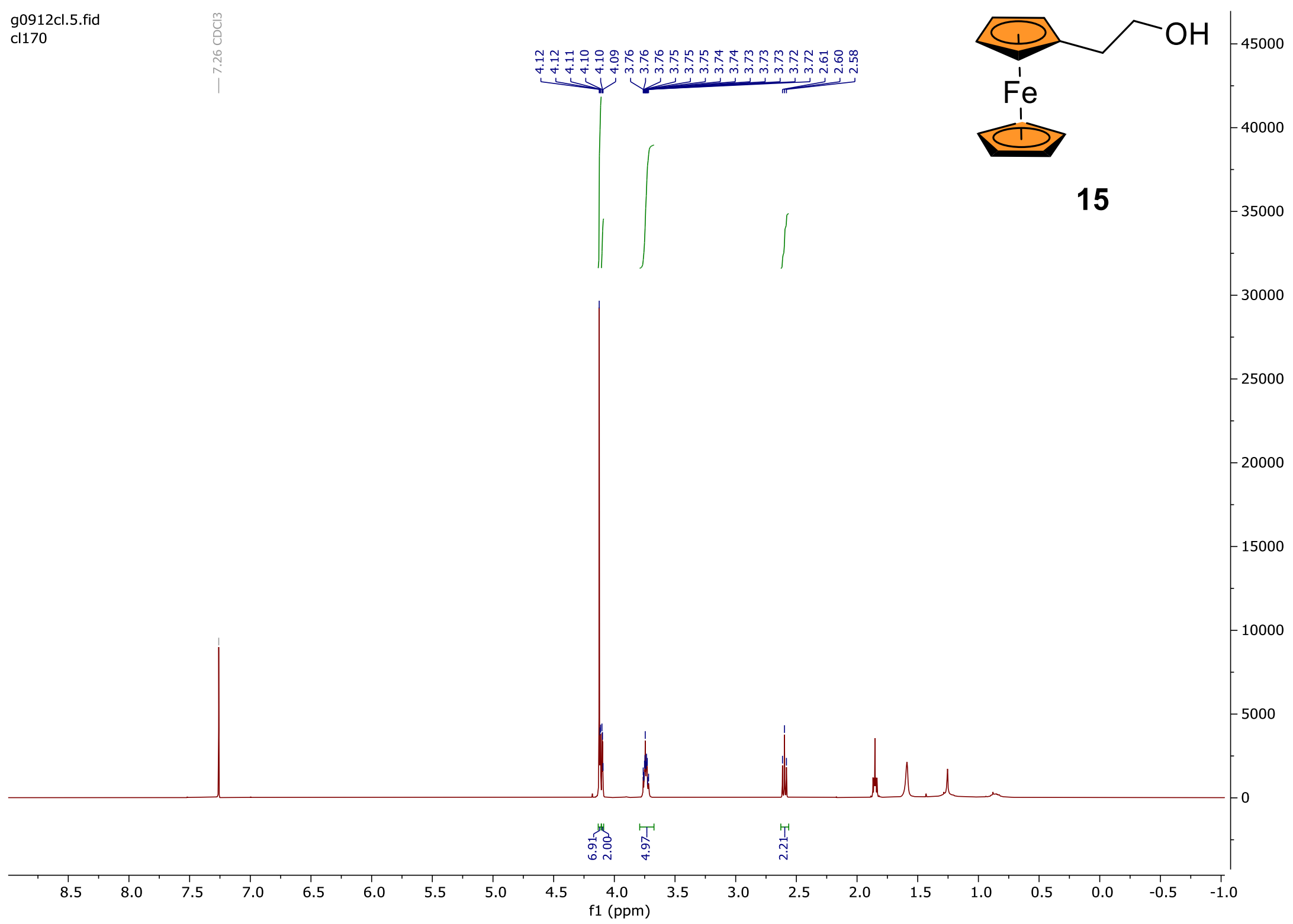

Figure S10. ^{1}H NMR spectrum of **15** in $CDCl_3$.

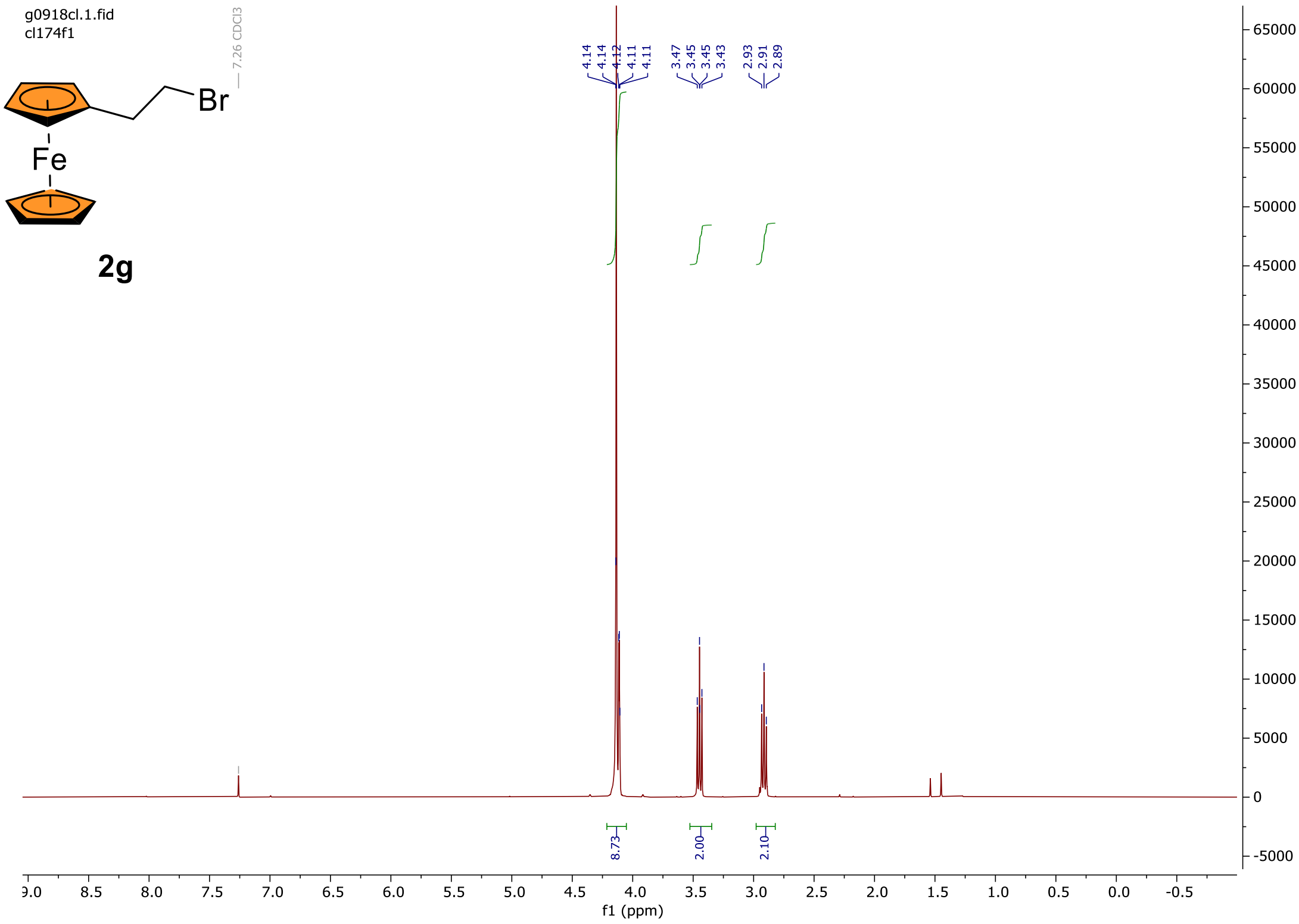

Figure S11. ^{1}H NMR spectrum of **2g** in $CDCl_3$.

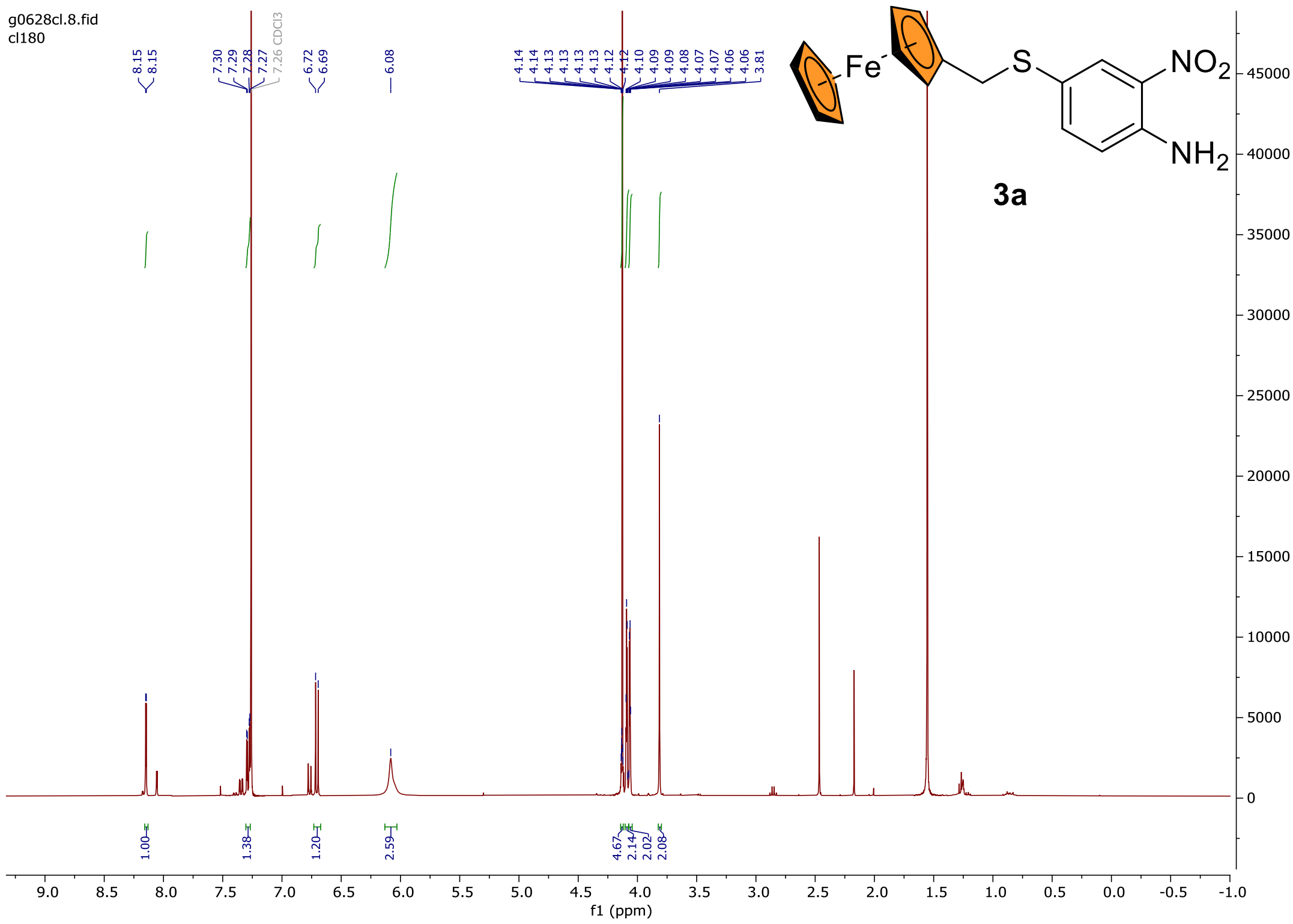

Figure S12. ^{1}H NMR spectrum of **3a** in $CDCl_3$.

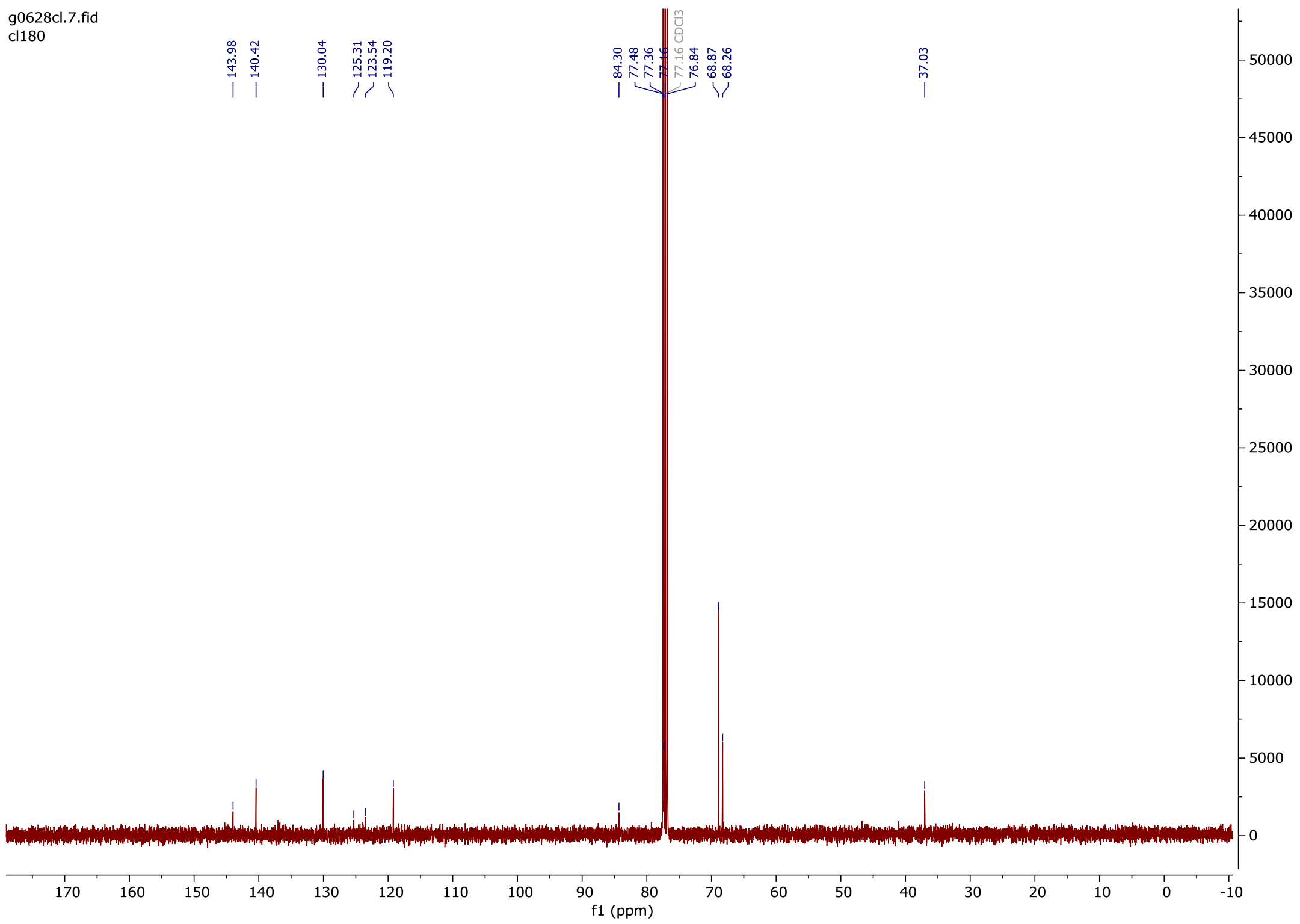

Figure S13. ^{13}C NMR spectrum of **3a** in $CDCl_3$.

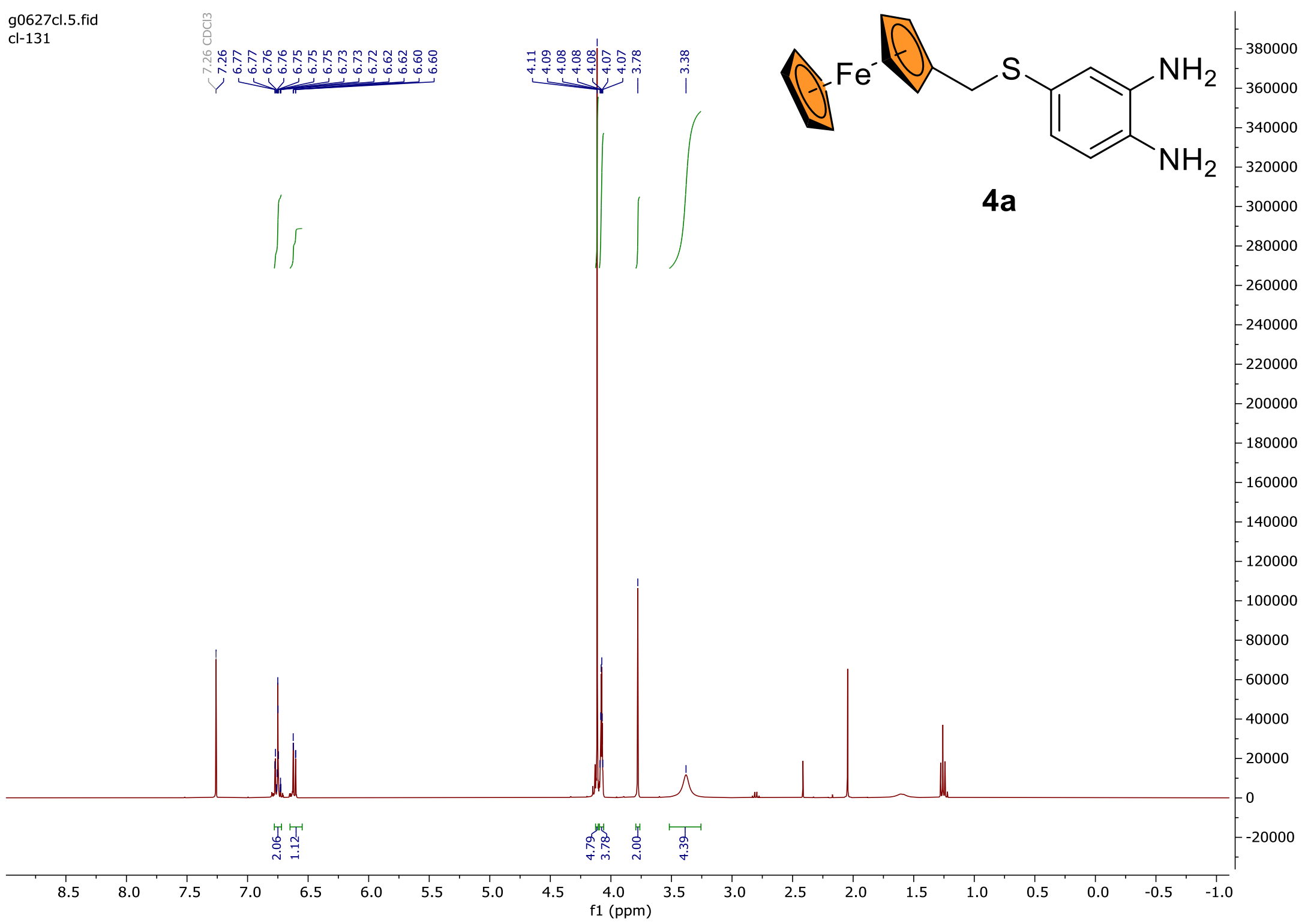

Figure S14. ^{1}H NMR spectrum of **4a** in $CDCl_3$.

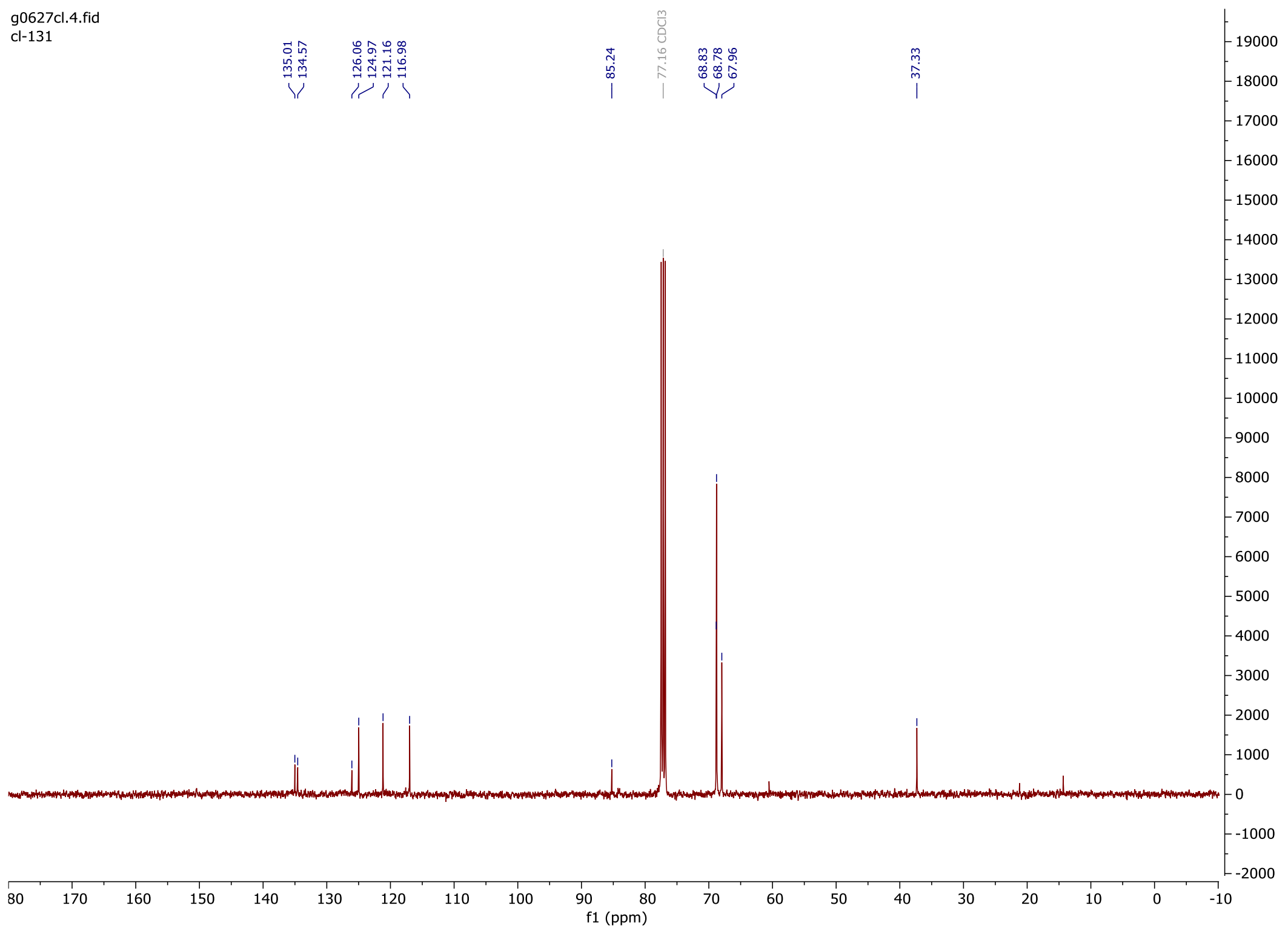

Figure S15. ^{13}C NMR spectrum of **4a** in $CDCl_3$.

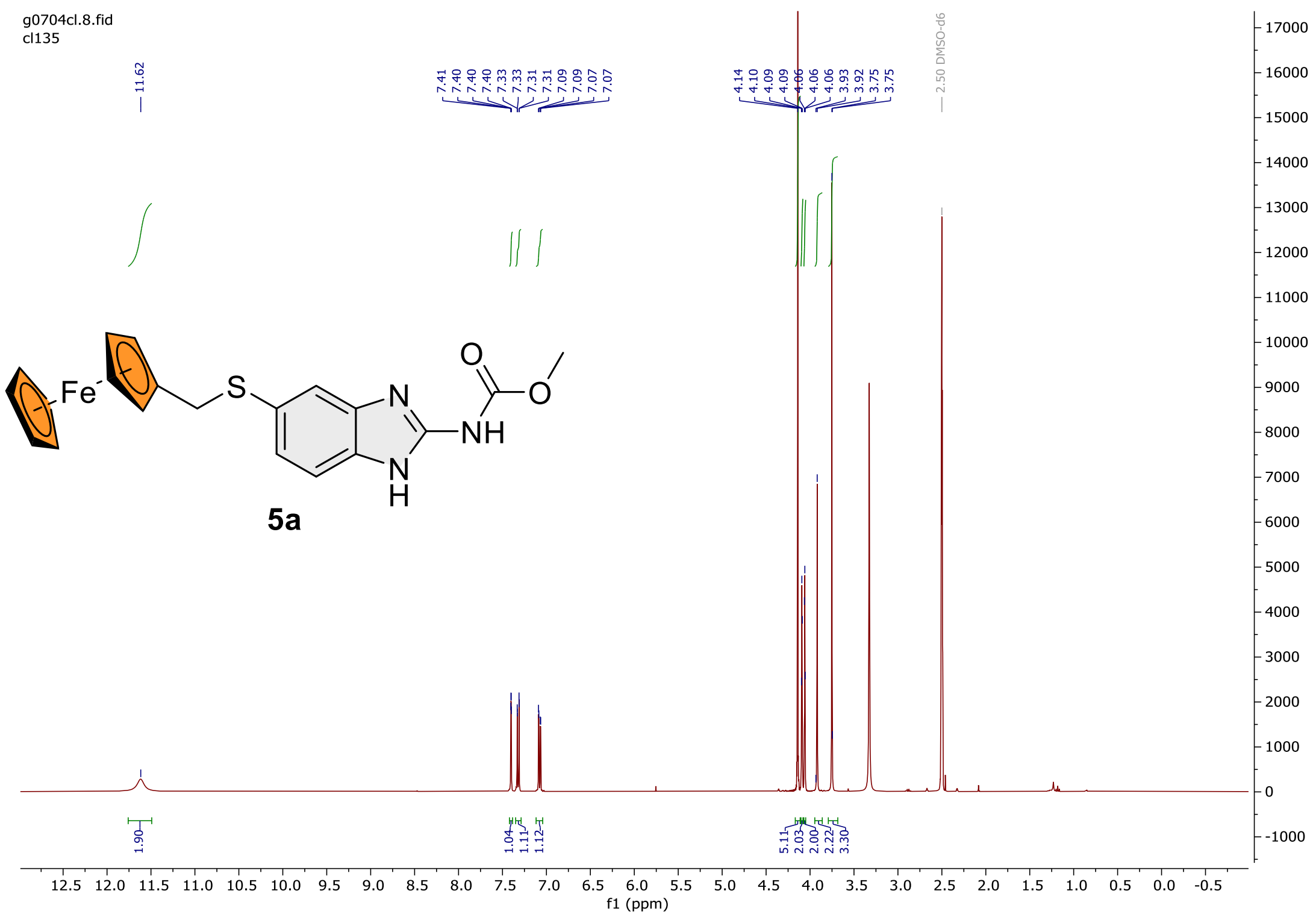

Figure S16. ^{1}H NMR spectrum of **5a** in $(CD_3)_2SO$.

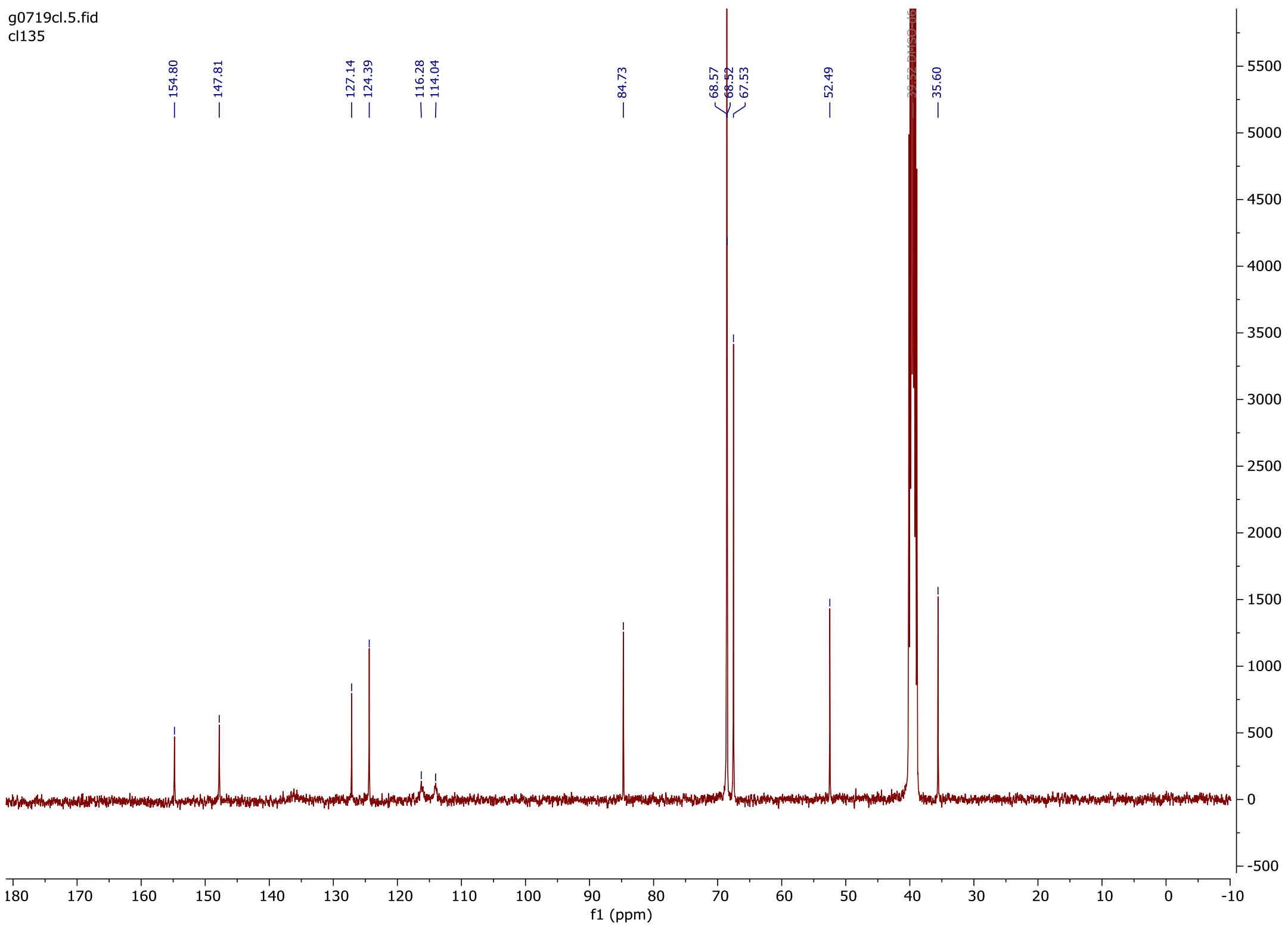

Figure S17. ^{13}C NMR spectrum of **5a** in $(CD_3)_2SO$.

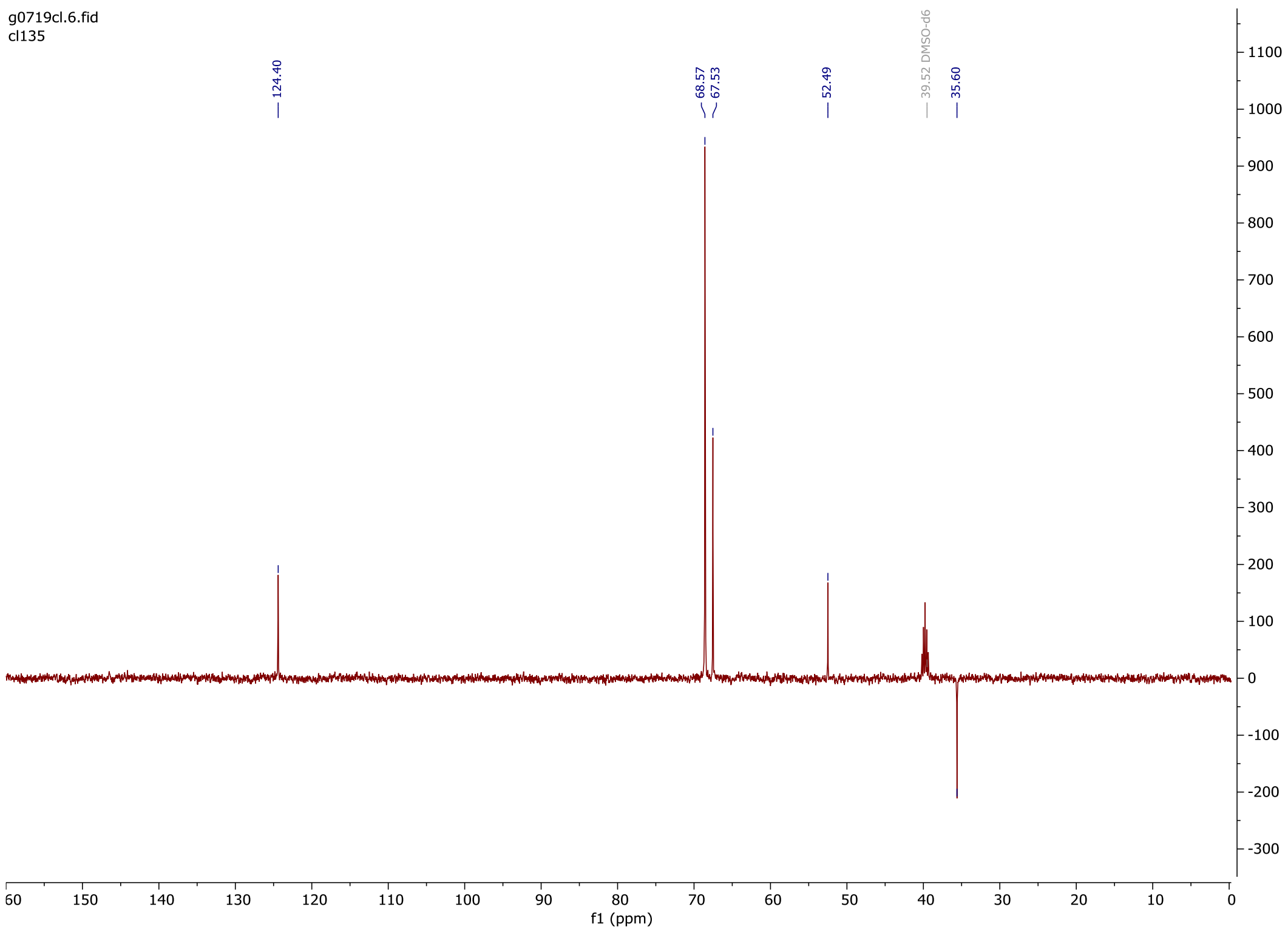

Figure S18. DEPT 135 NMR spectrum of **5a** in $(CD_3)_2SO$.

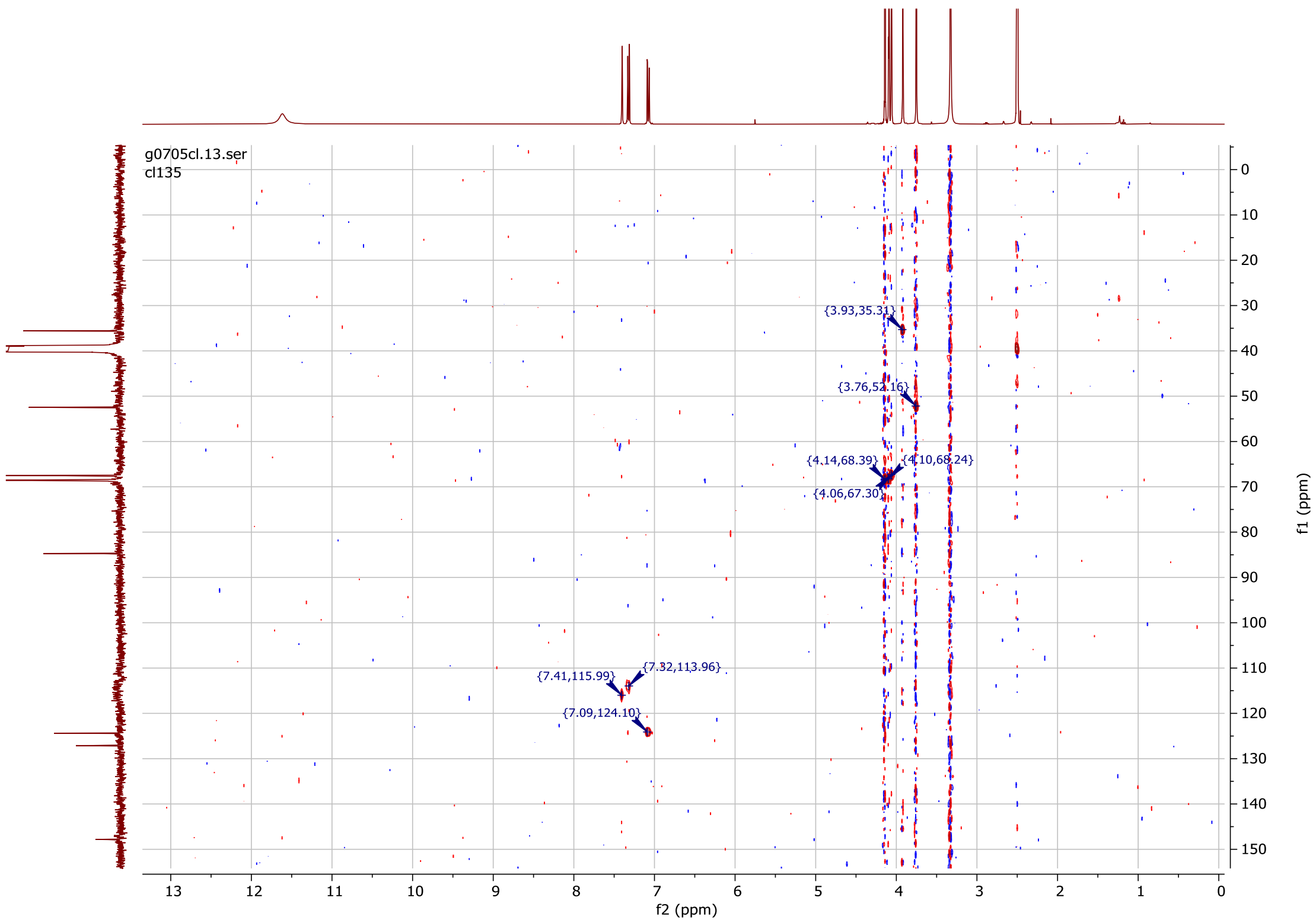

Figure S19. HSQC NMR spectrum of **5a** in $(CD_3)_2SO$.

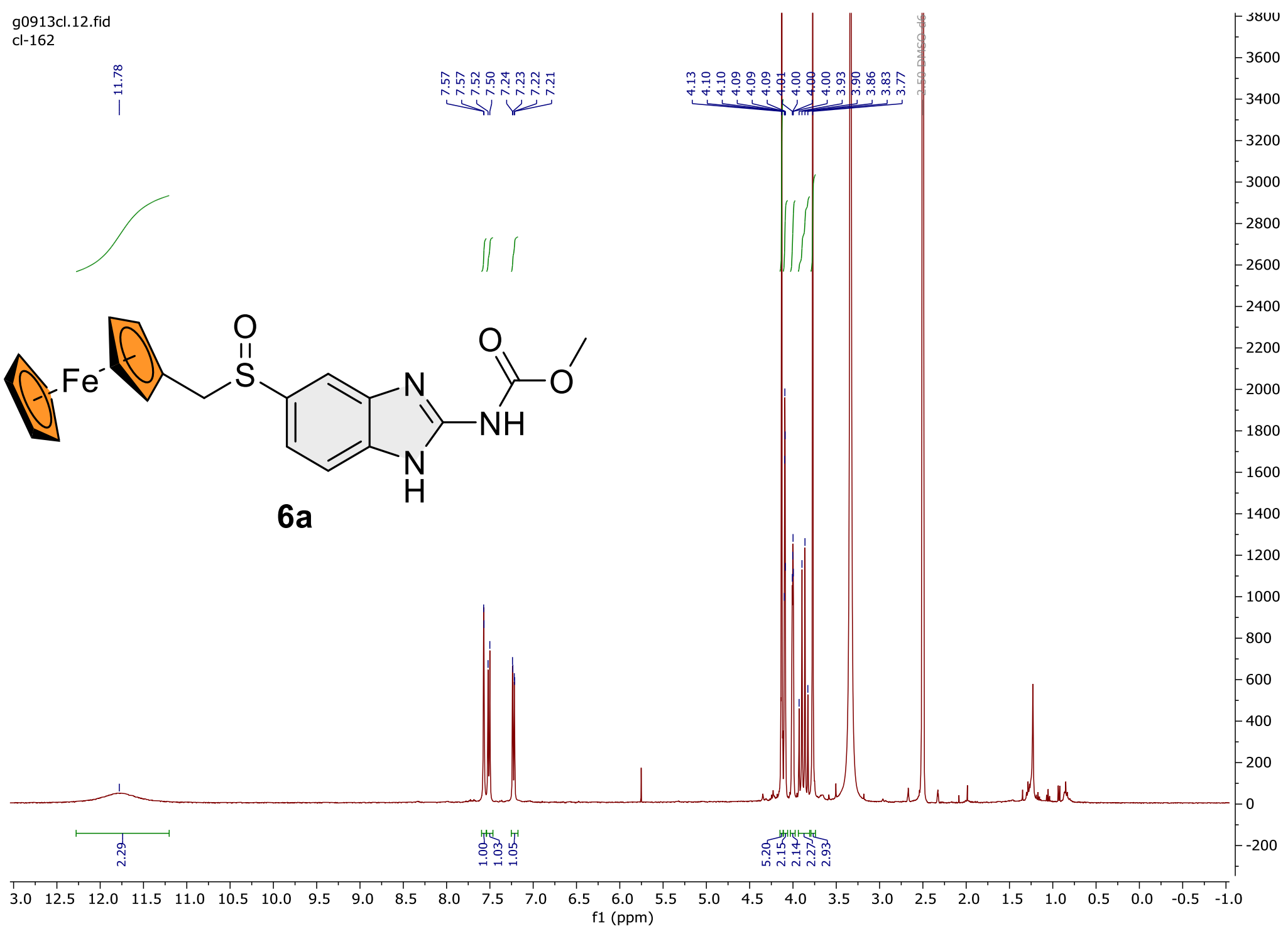

Figure S20. ^{1}H NMR spectrum of **6a** in $(CD_3)_2SO$.

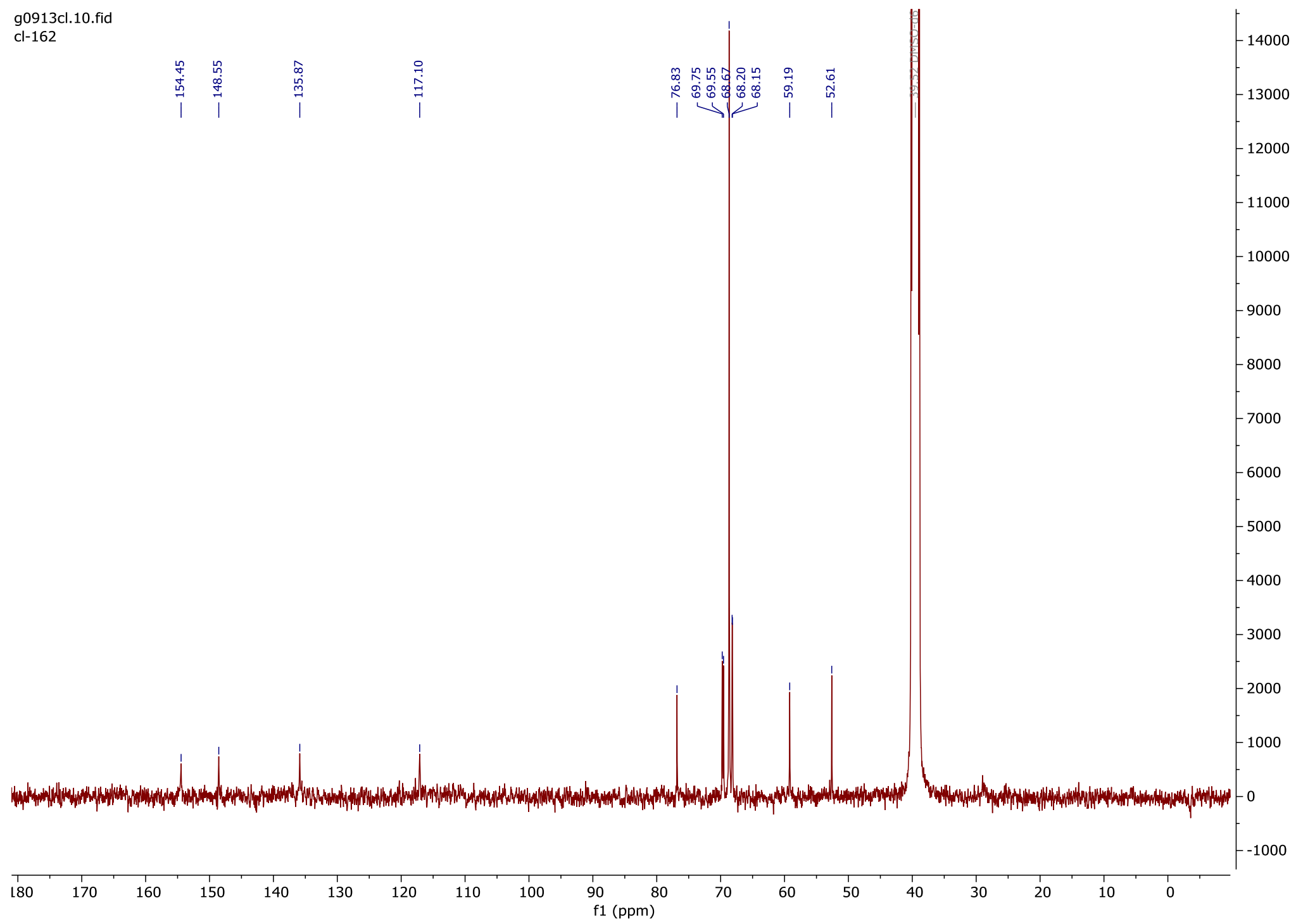

Figure S21. ^{13}C NMR spectrum of **6a** in $(CD_3)_2SO$.

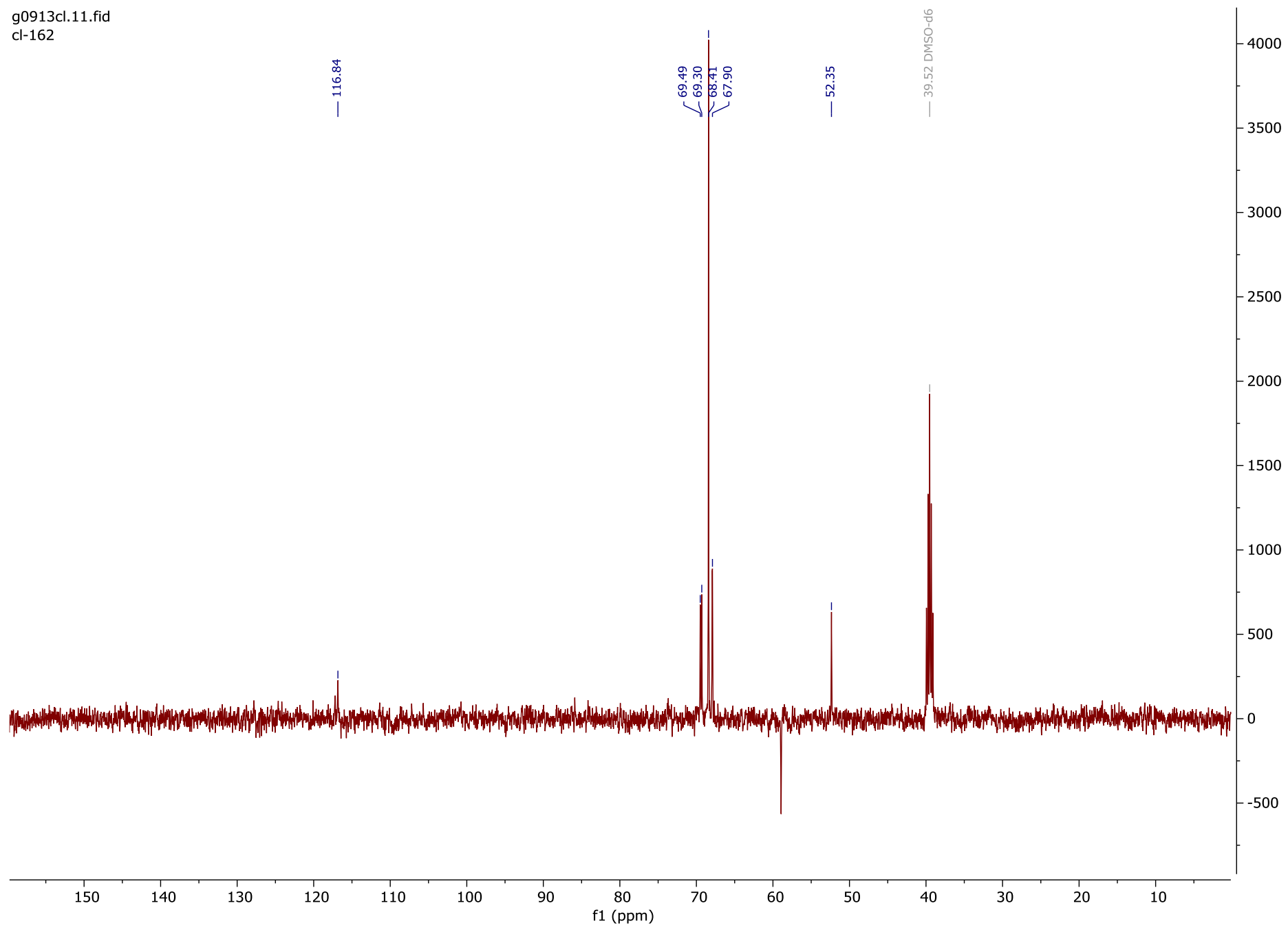

Figure S22. DEPT 135 NMR spectrum of **6a** in $(CD_3)_2SO$.

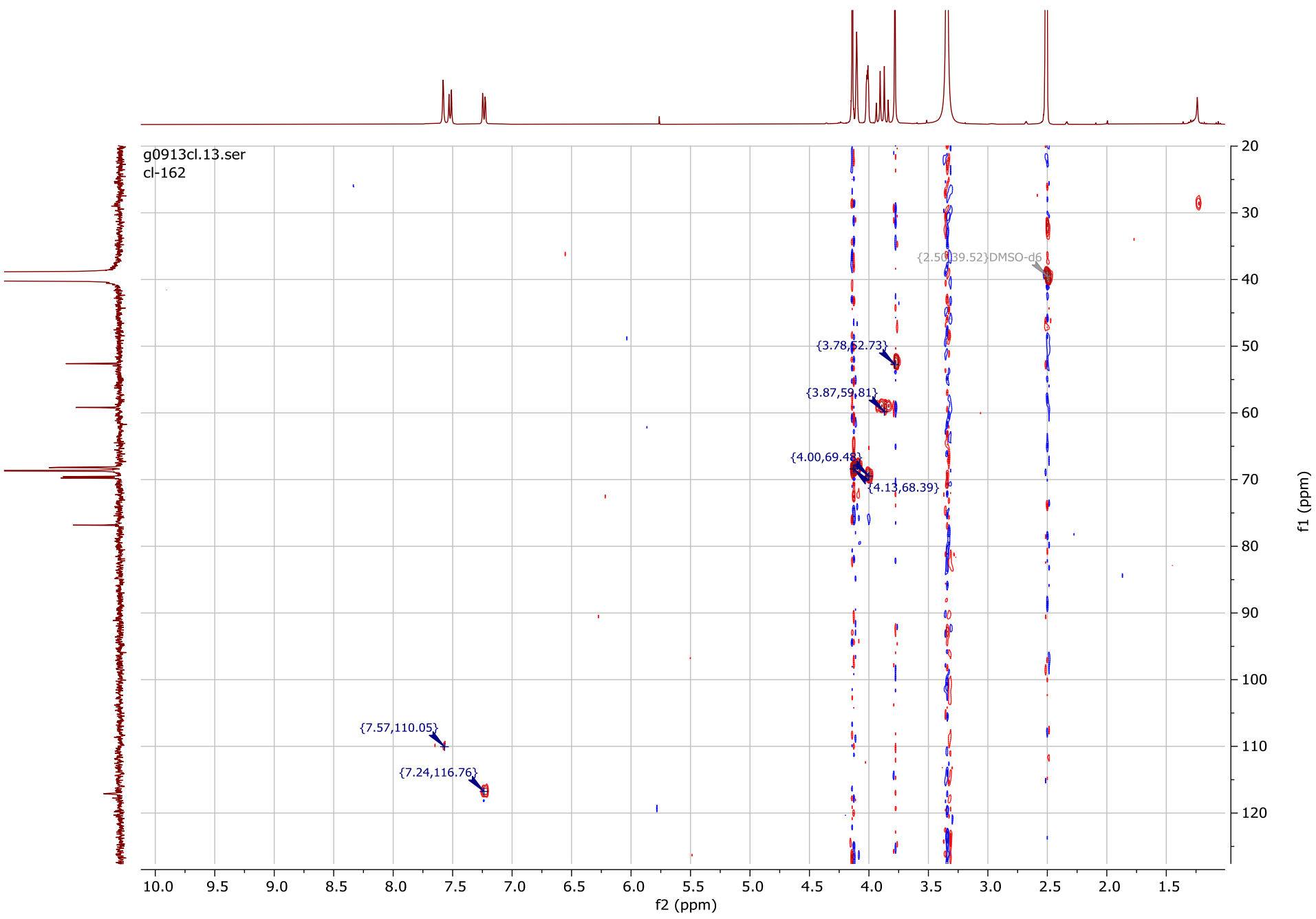

Figure S23. HSQC NMR spectrum of **6a** in $(CD_3)_2SO$.

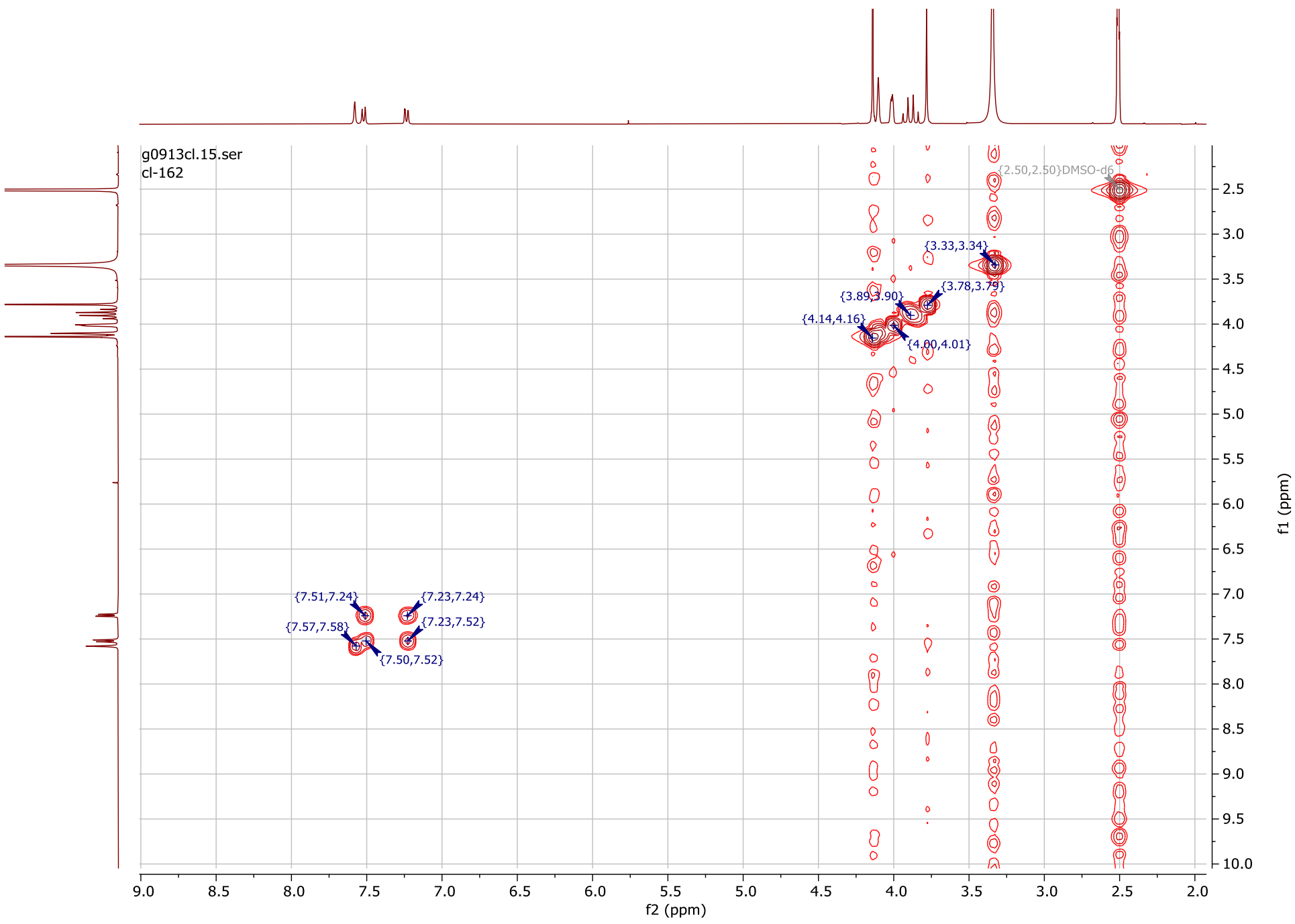

Figure S24. COSY NMR spectrum of **6a** in $(CD_3)_2SO$.

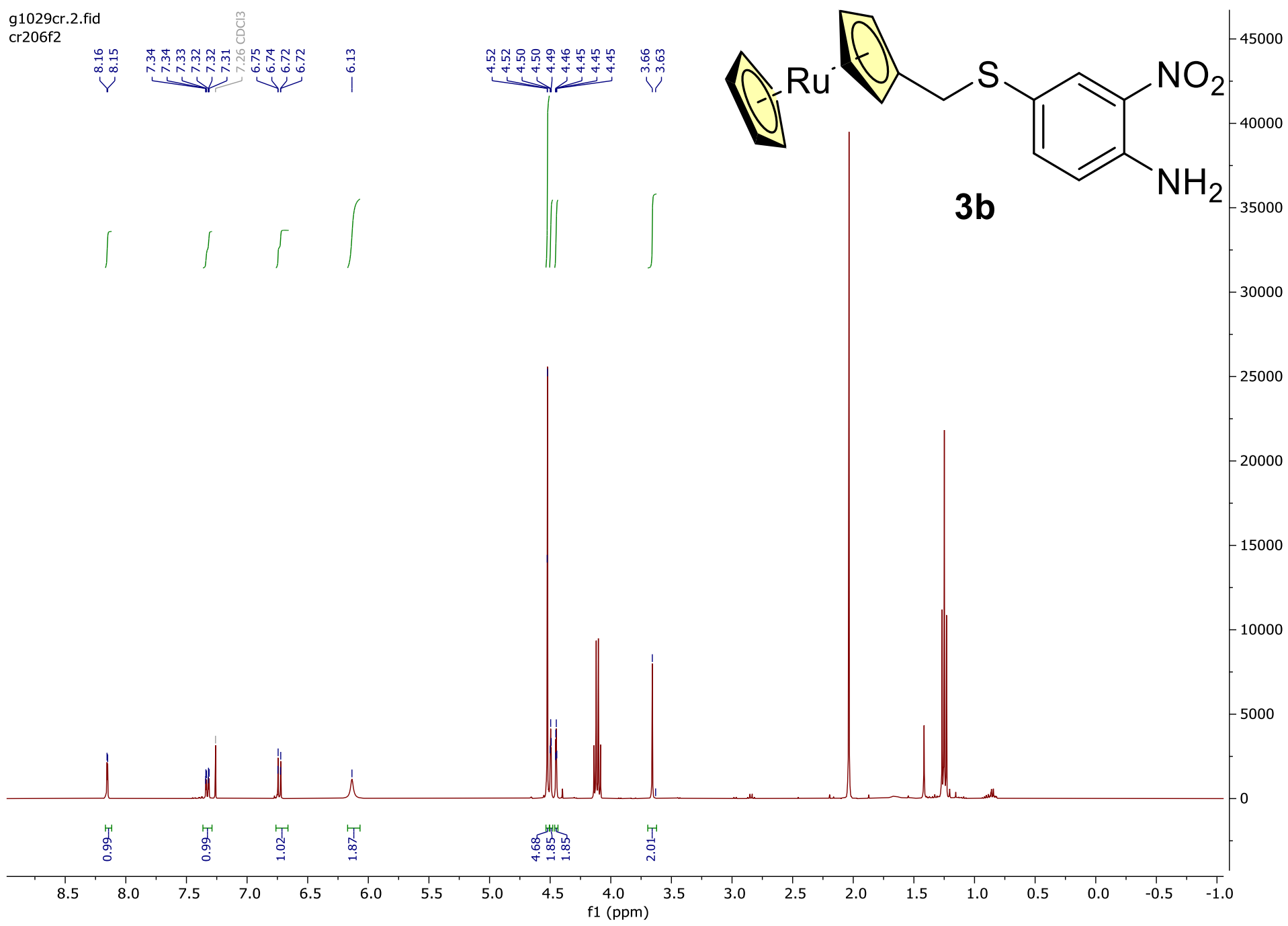

Figure S25. ^{1}H NMR spectrum of **3b** in $CDCl_3$.

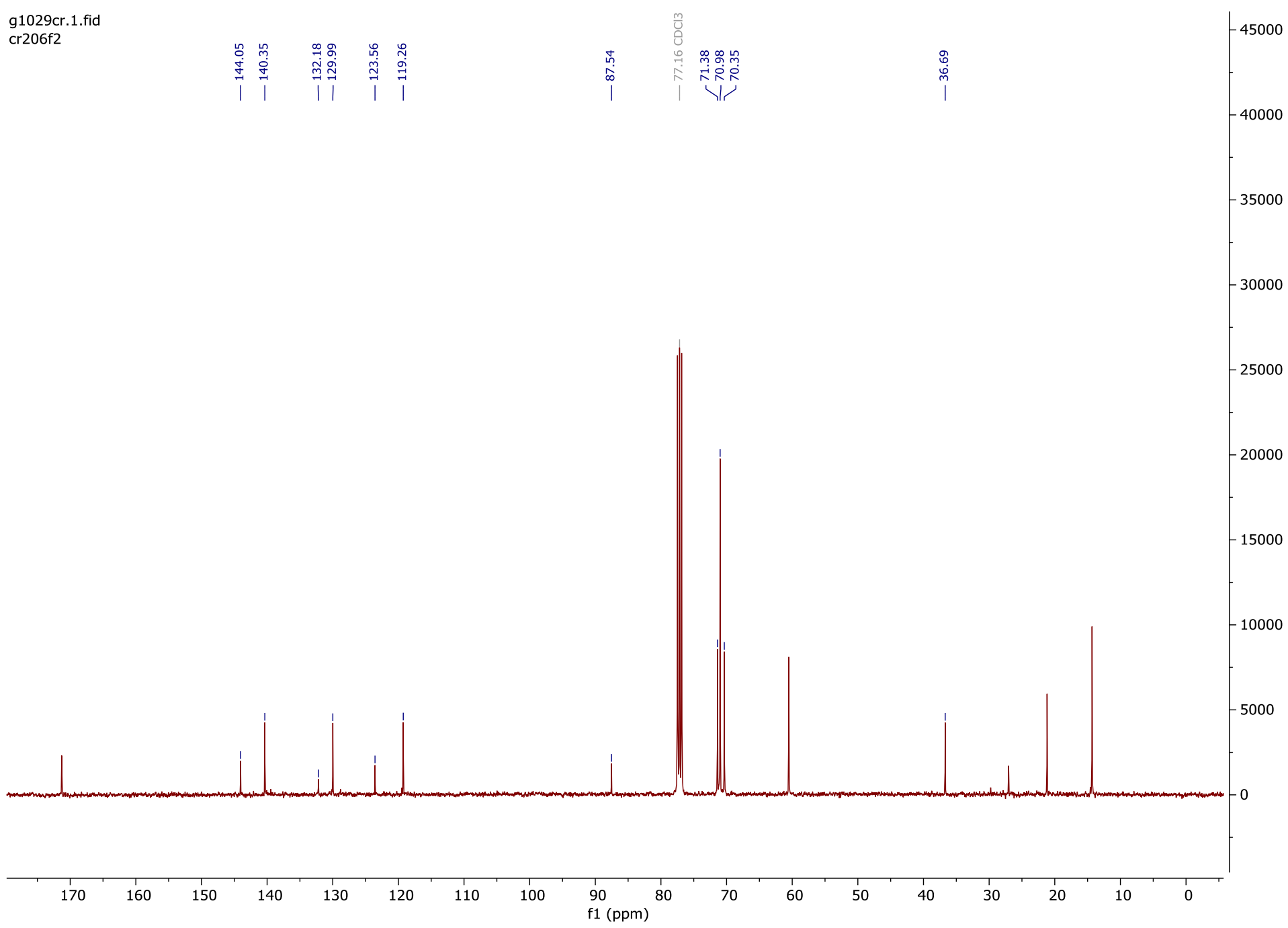

Figure S26. ^{13}C NMR spectrum of **3b** in $CDCl_3$.

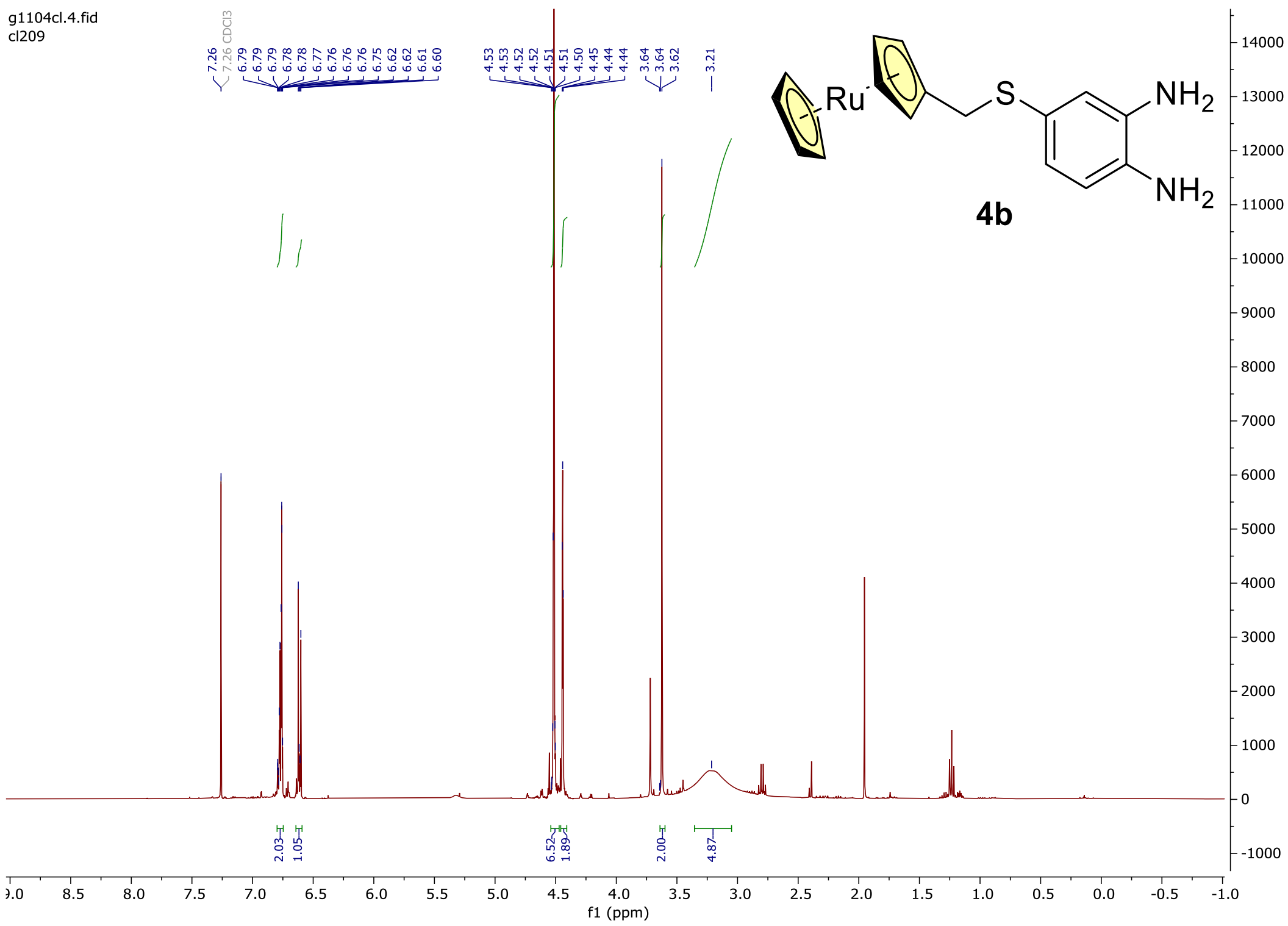

Figure S27. ^{1}H NMR spectrum of **4b** in $CDCl_3$.

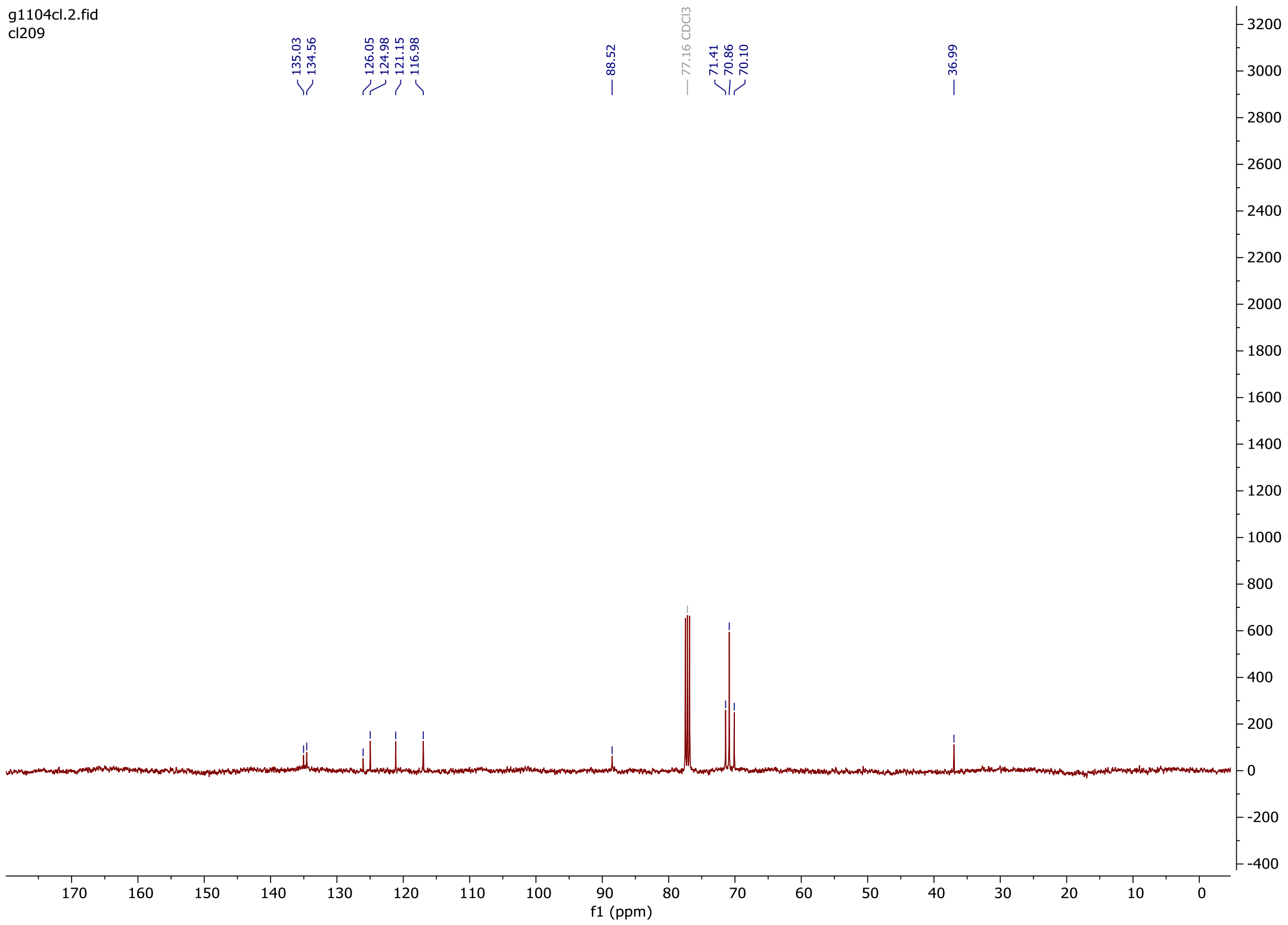

Figure S28. ^{13}C NMR spectrum of **4b** in $CDCl_3$.

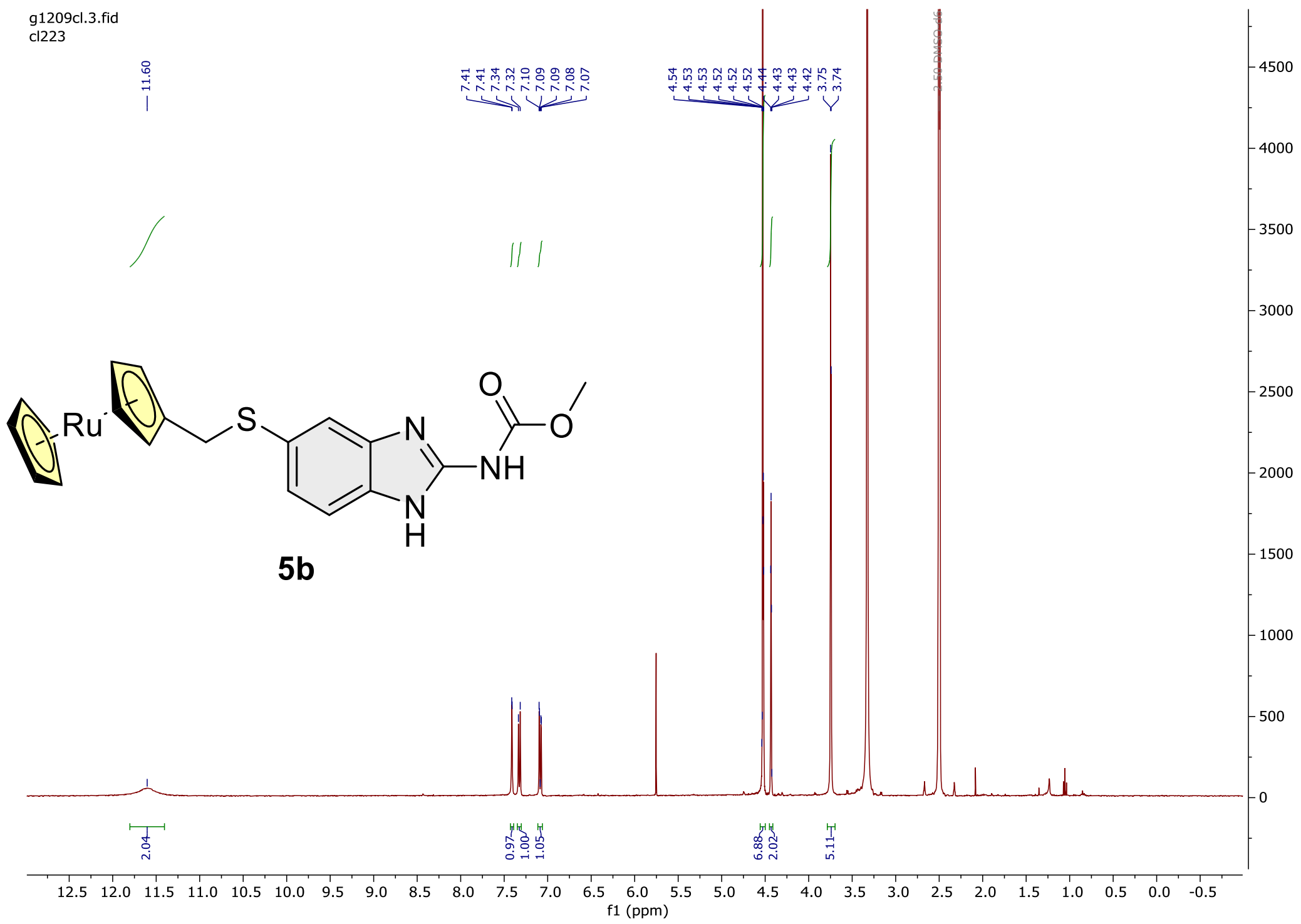

Figure S29. ^{1}H NMR spectrum of **5b** in $(CD_3)_2SO$.

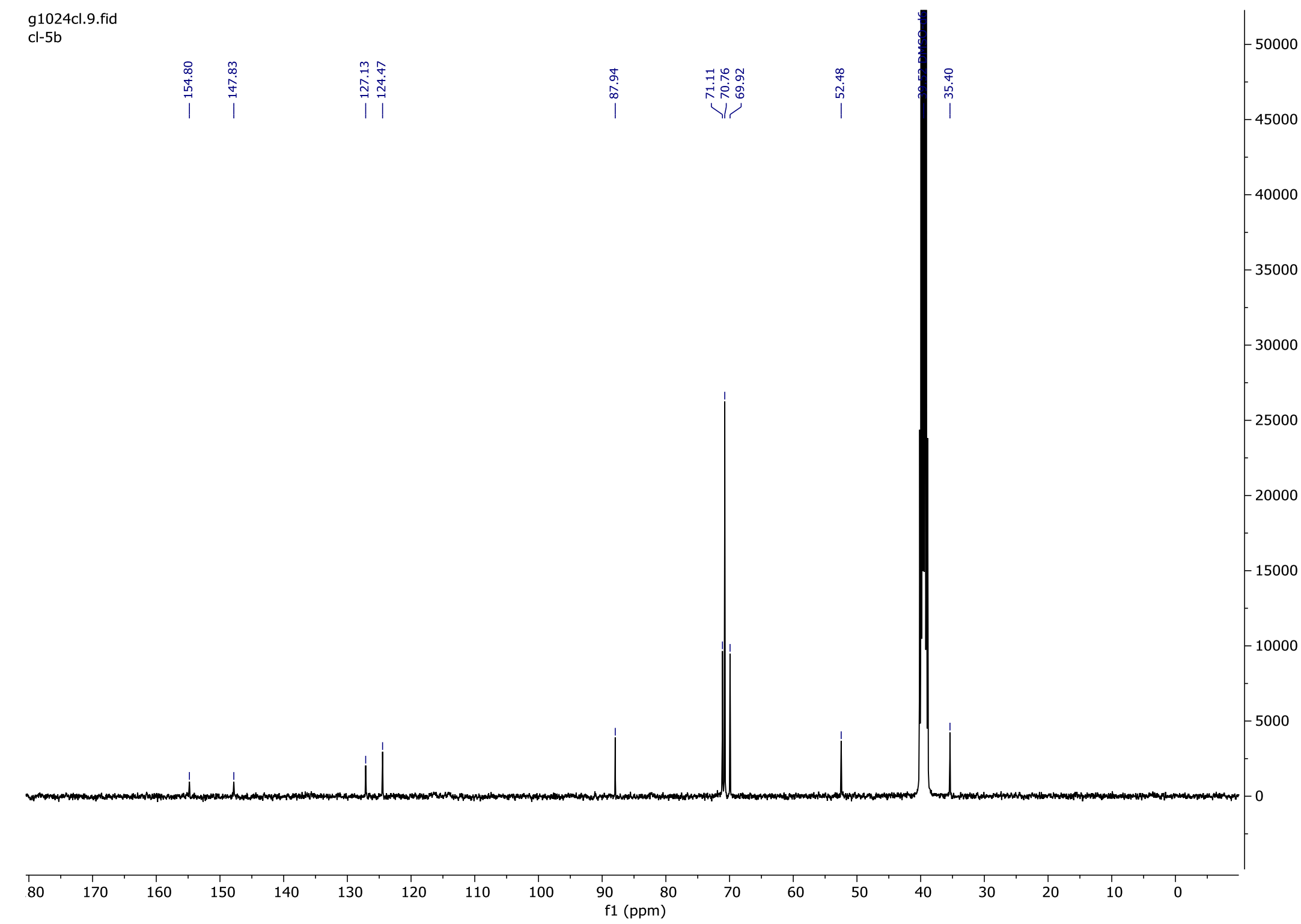

Figure S30. ^{13}C NMR spectrum of **5b** in $(CD_3)_2SO$.

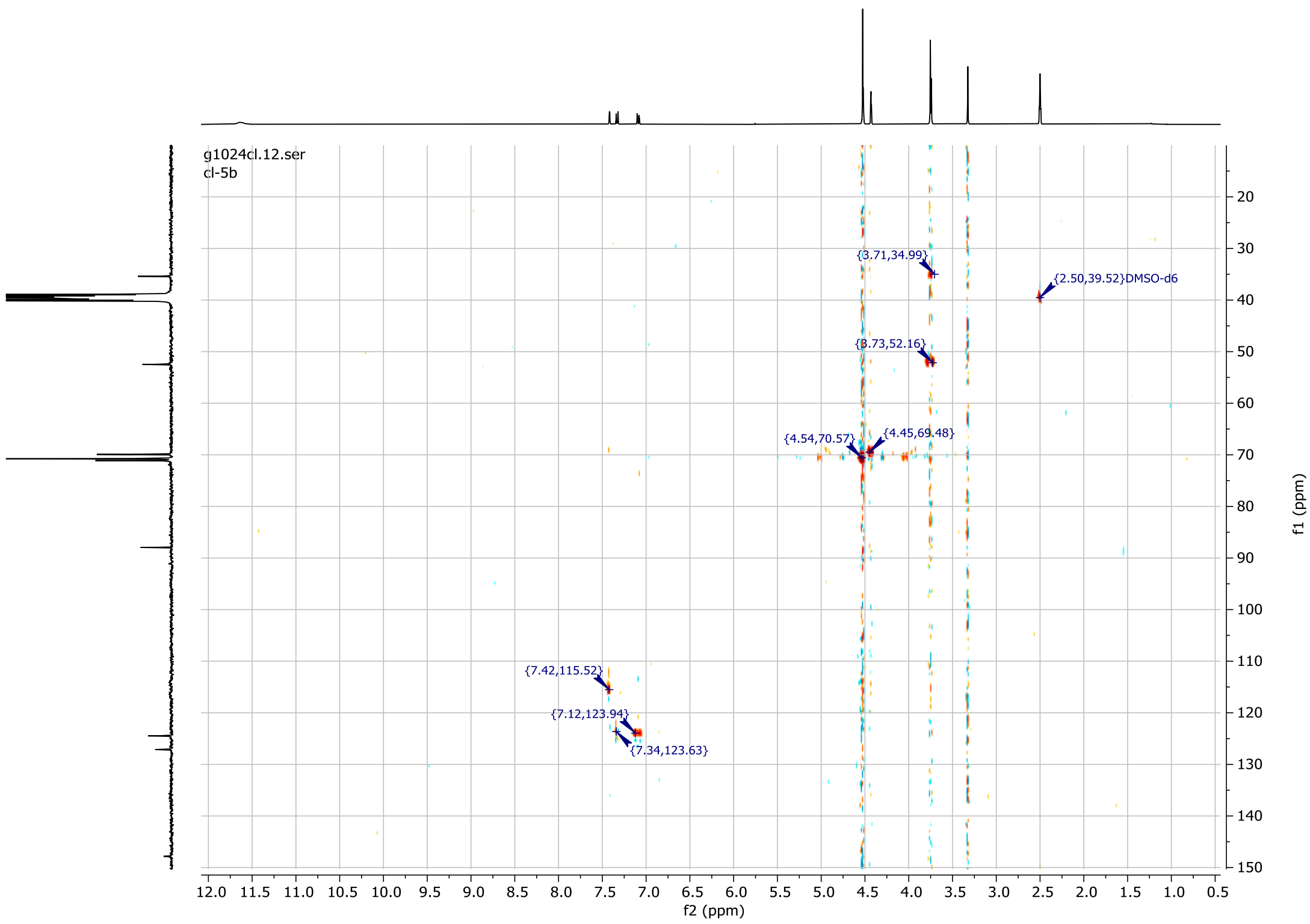

Figure S31. HSQC NMR spectrum of **5b** in $(CD_3)_2SO$.

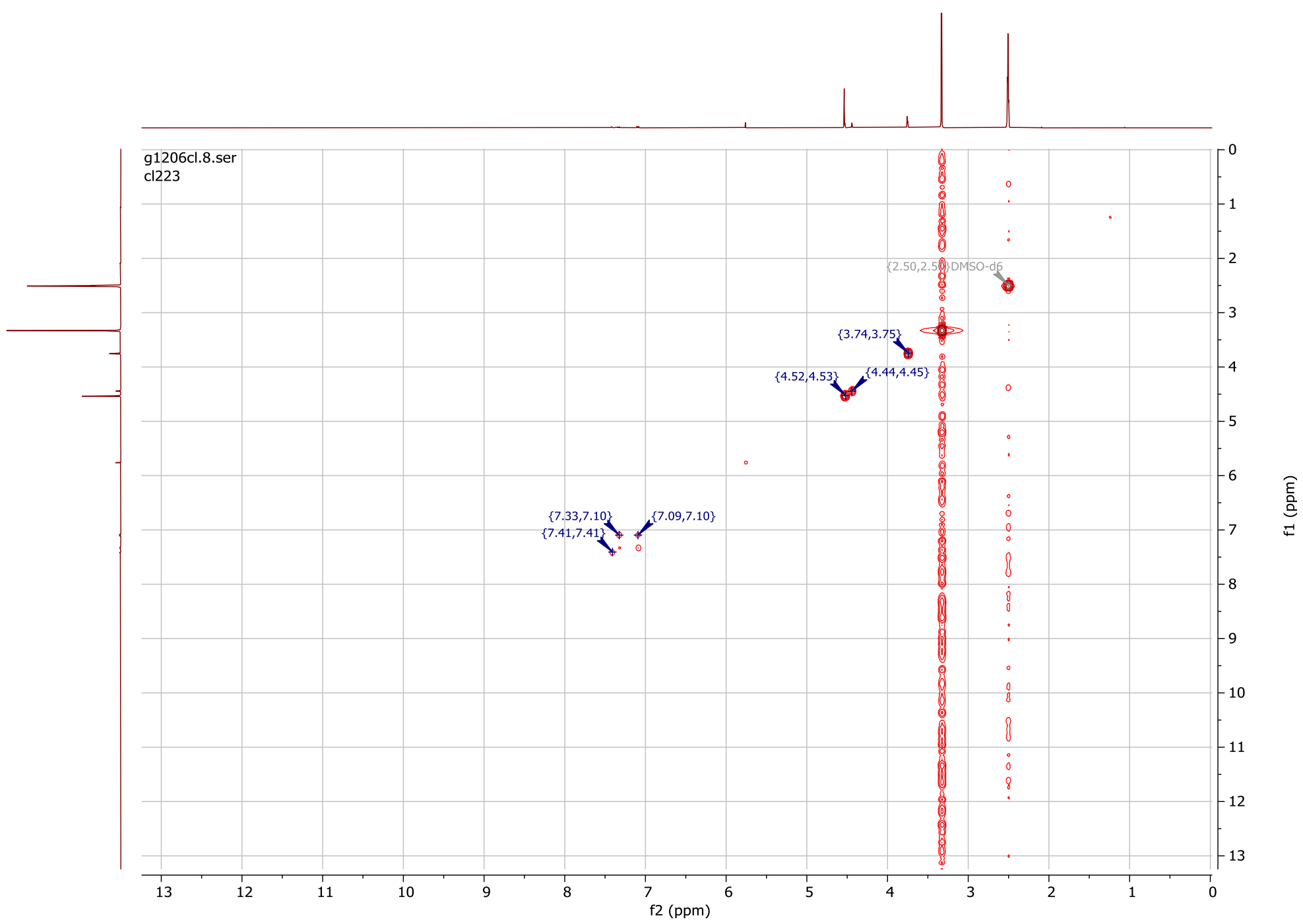

Figure S32. COSY NMR spectrum of **5b** in $(CD_3)_2SO$.

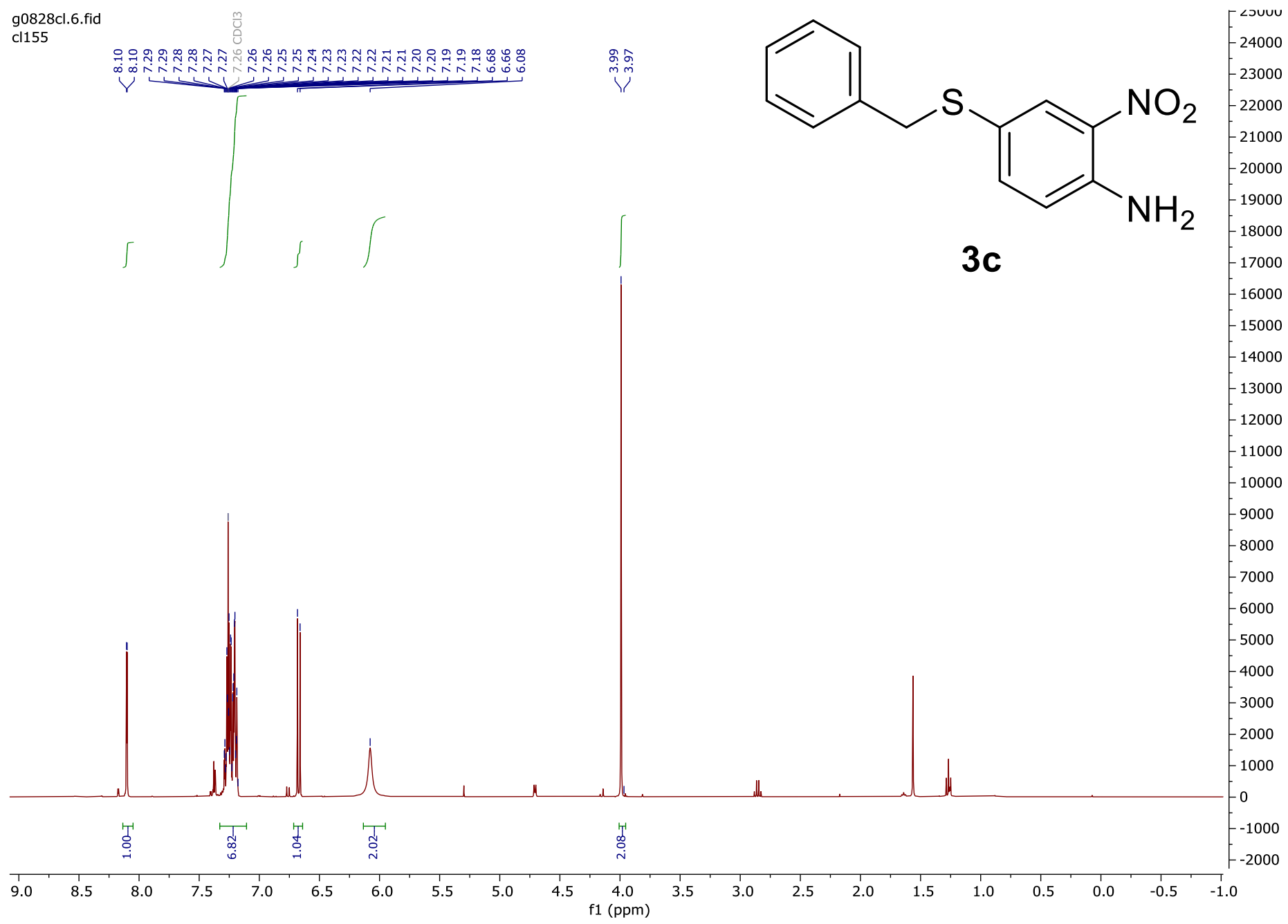

Figure S33. ^{1}H NMR spectrum of **3c** in $CDCl_3$.

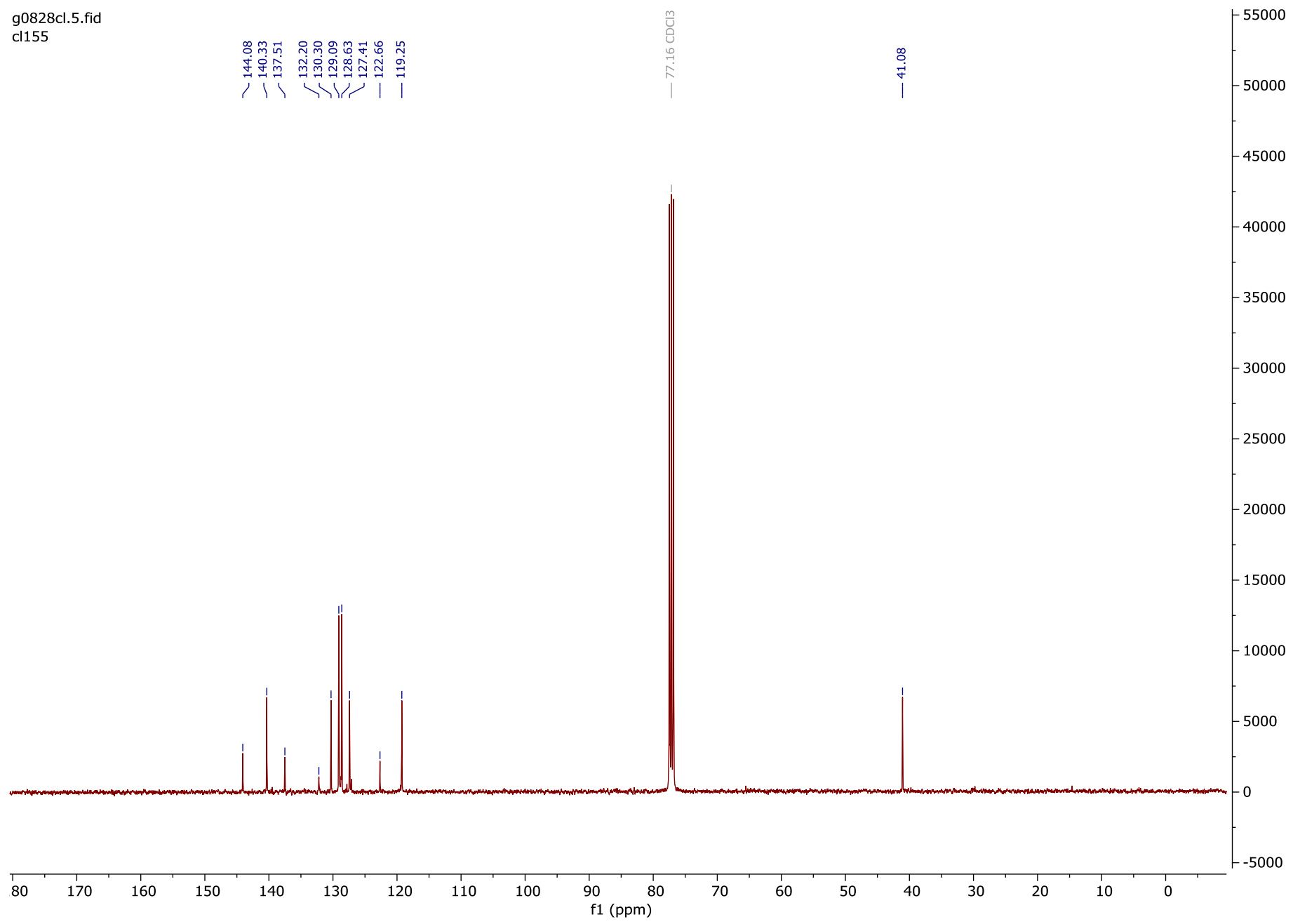

Figure S34. ^{13}C NMR spectrum of **3c** in $CDCl_3$.

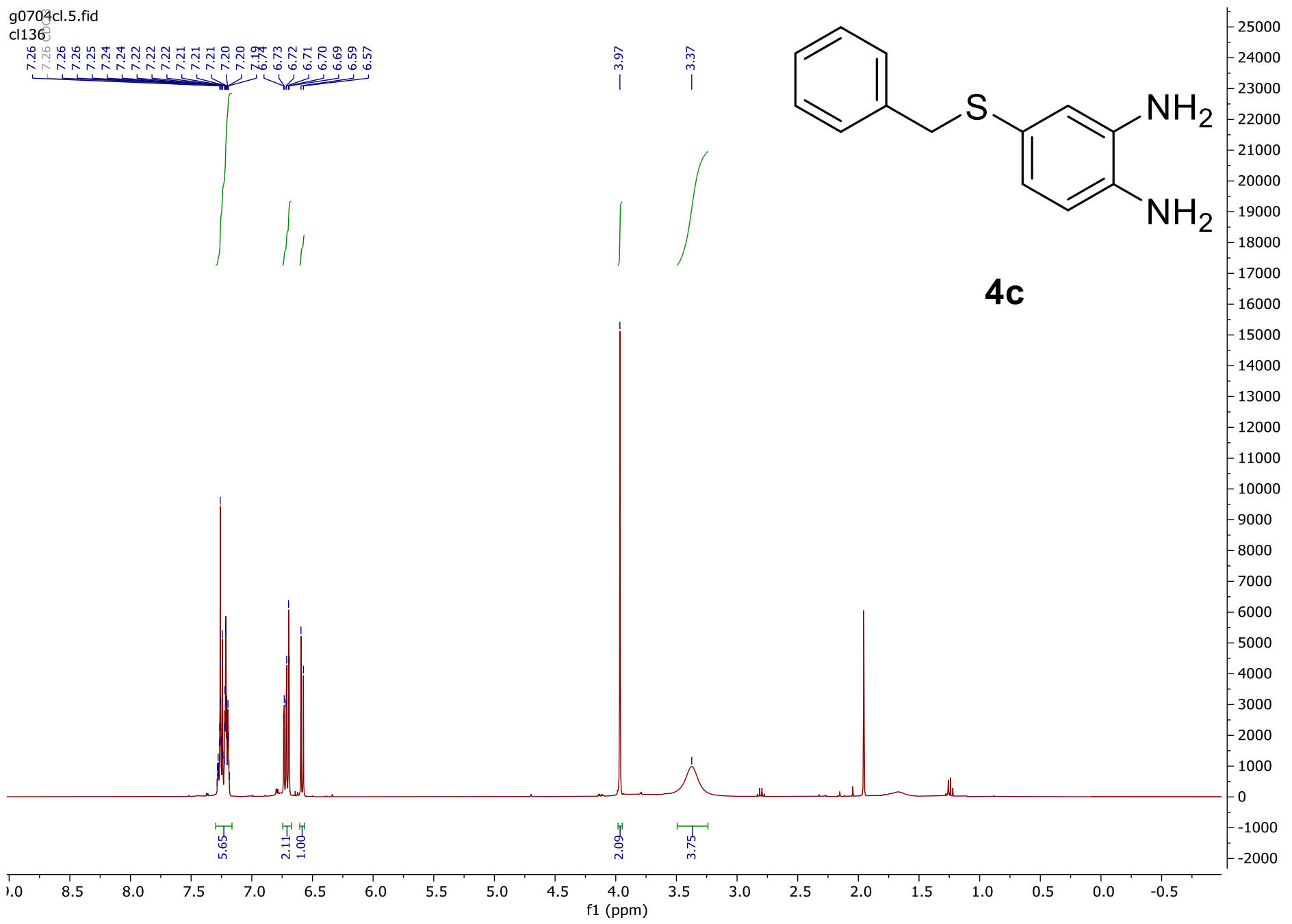

Figure S35. ^{1}H NMR spectrum of **4c** in $CDCl_3$.

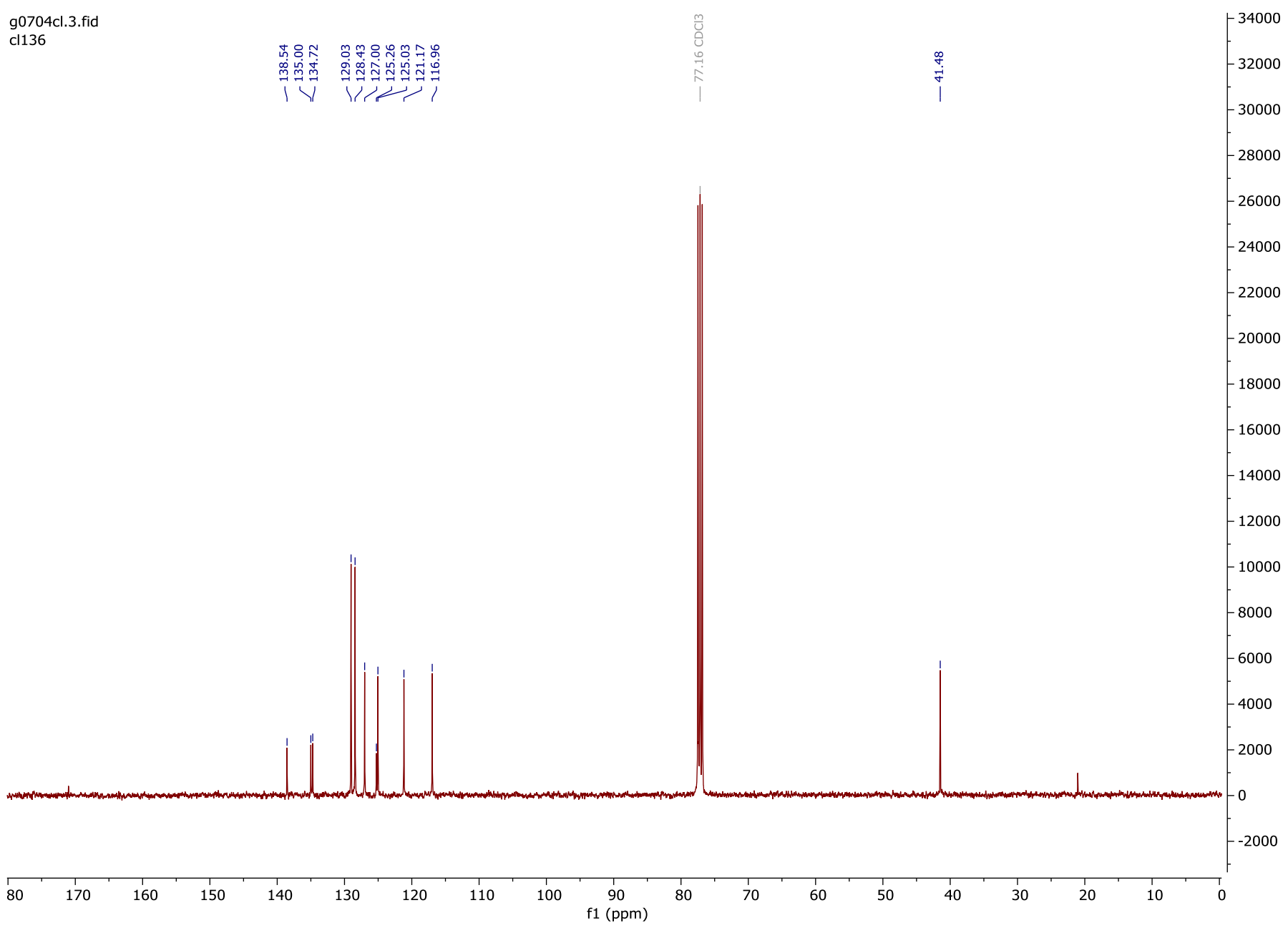

Figure S36. ^{13}C NMR spectrum of **4c** in $CDCl_3$.

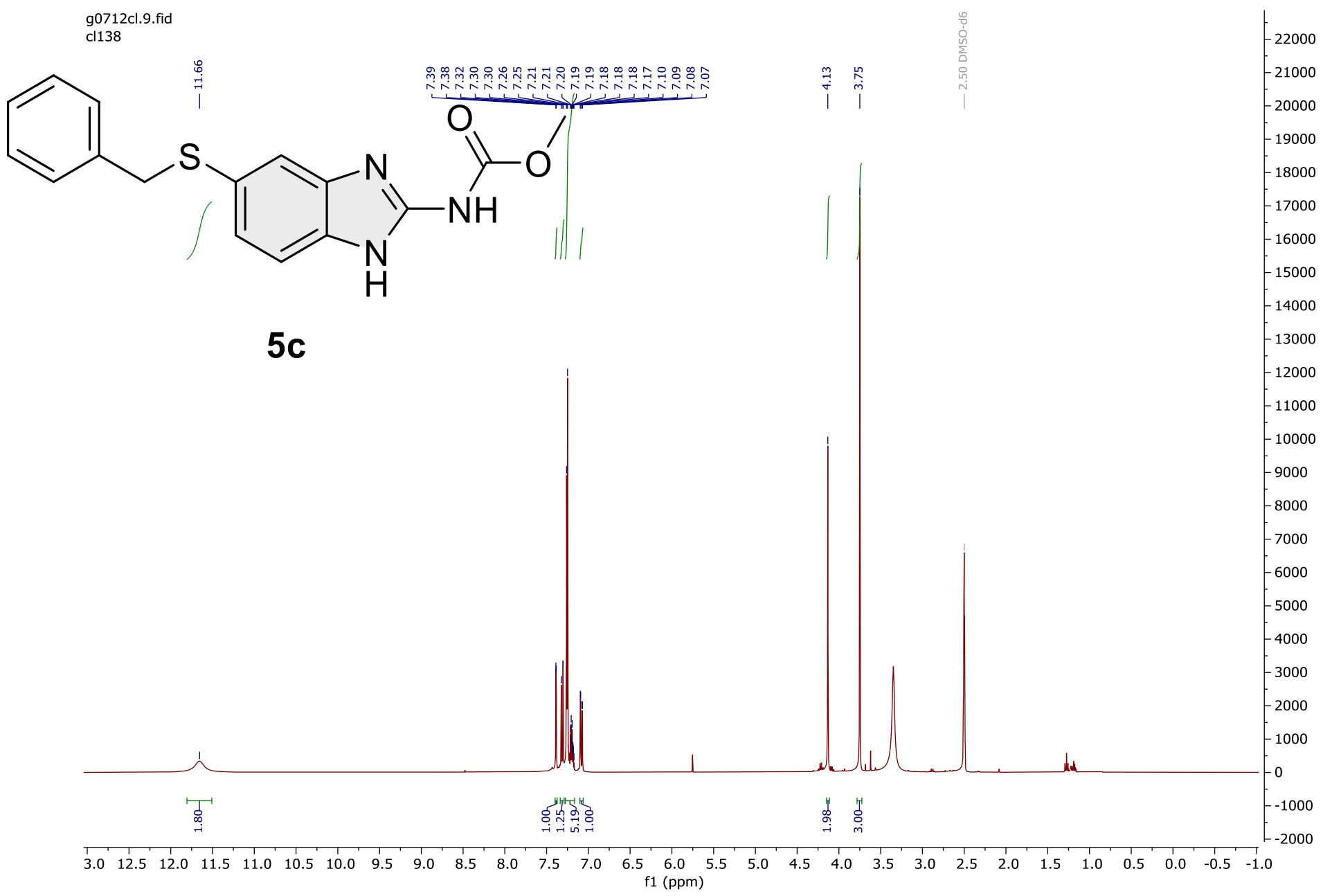

Figure S37. ^{1}H NMR spectrum of **5c** in $(CD_3)_2SO$.

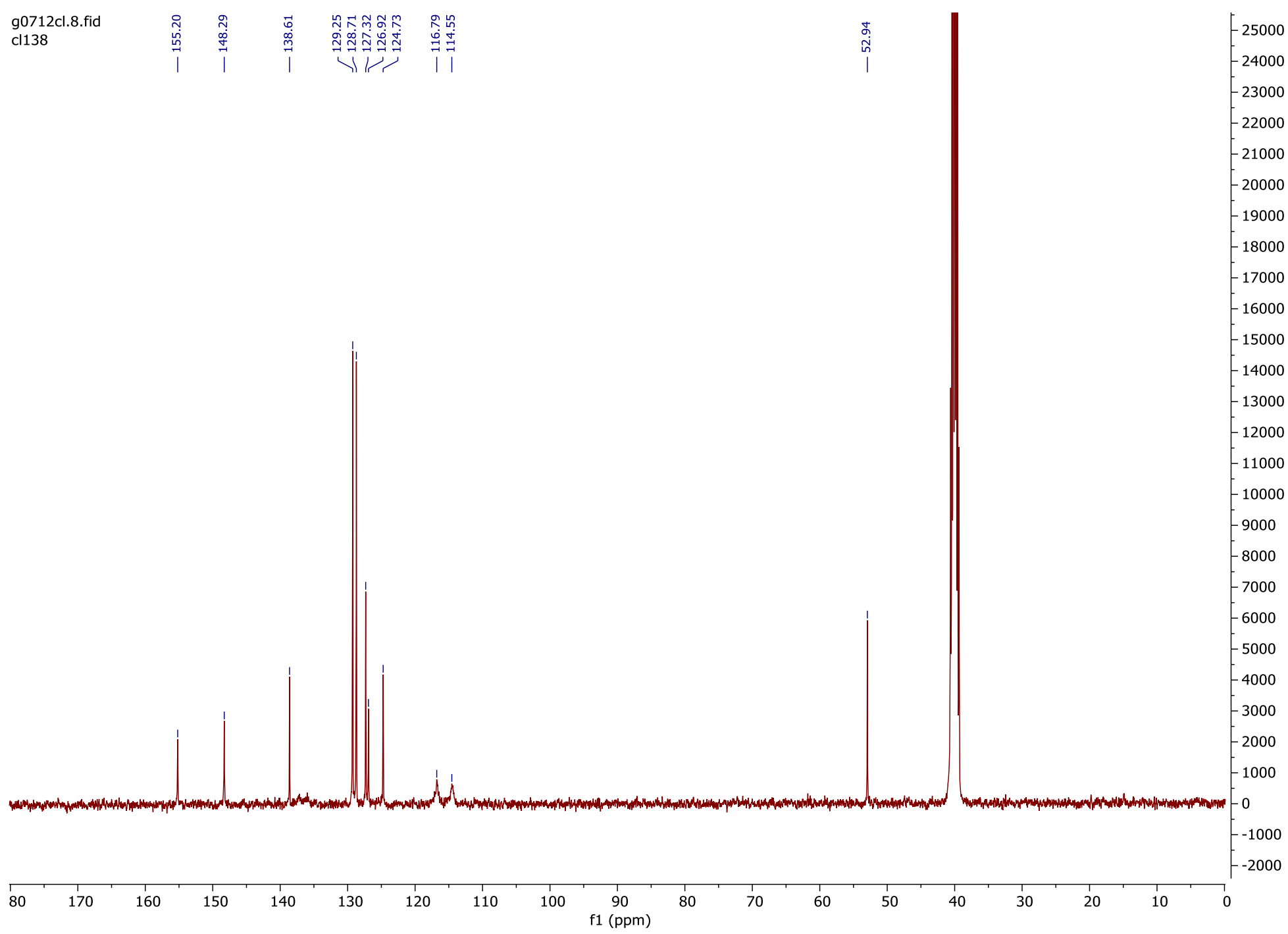

Figure S38. ^{13}C NMR spectrum of **5c** in $(CD_3)_2SO$.

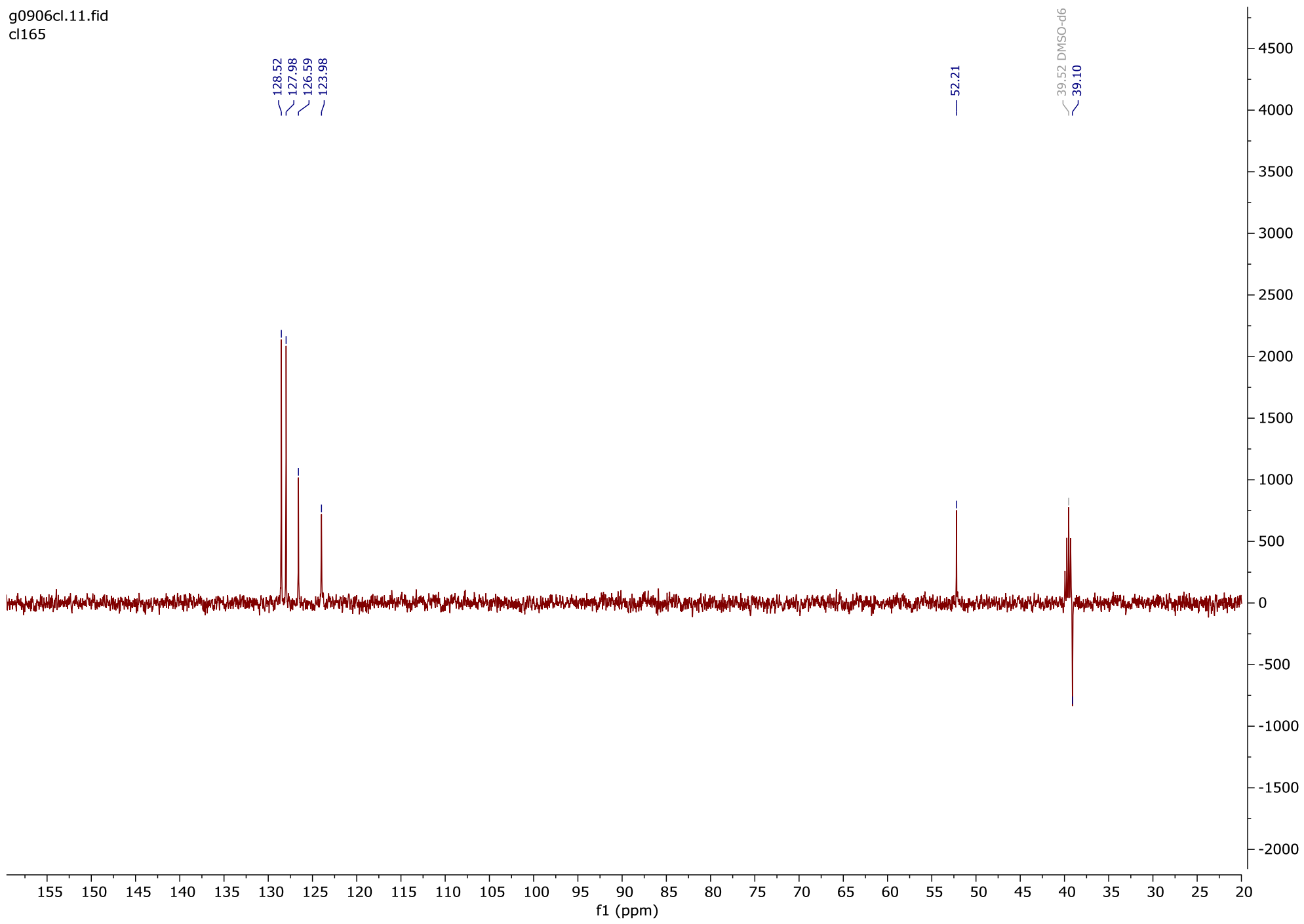

Figure S39. DEPT 135 NMR spectrum of **5c** in $(CD_3)_2SO$.

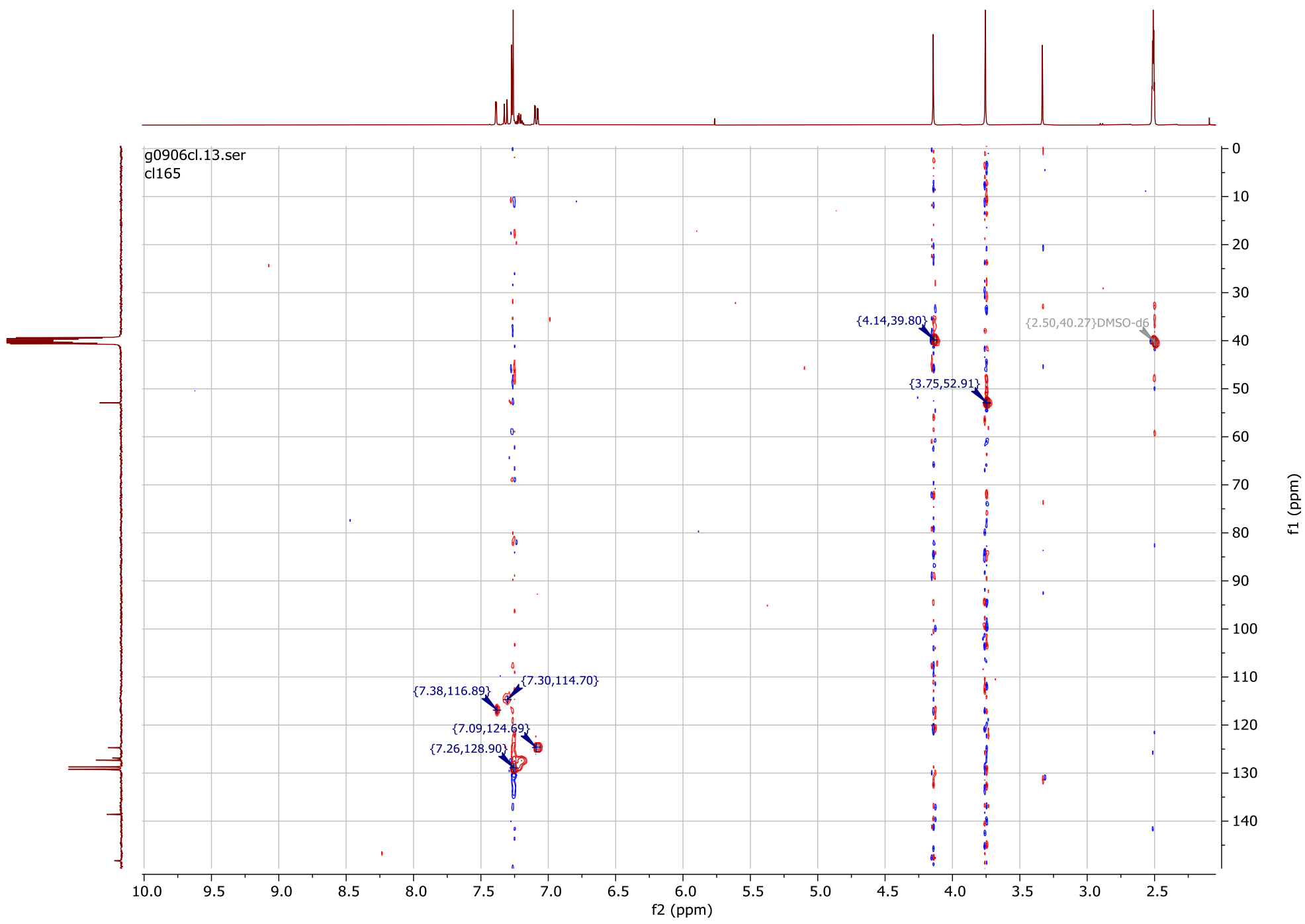

Figure S40. HSQC NMR spectrum of **5c** in $(CD_3)_2SO$.

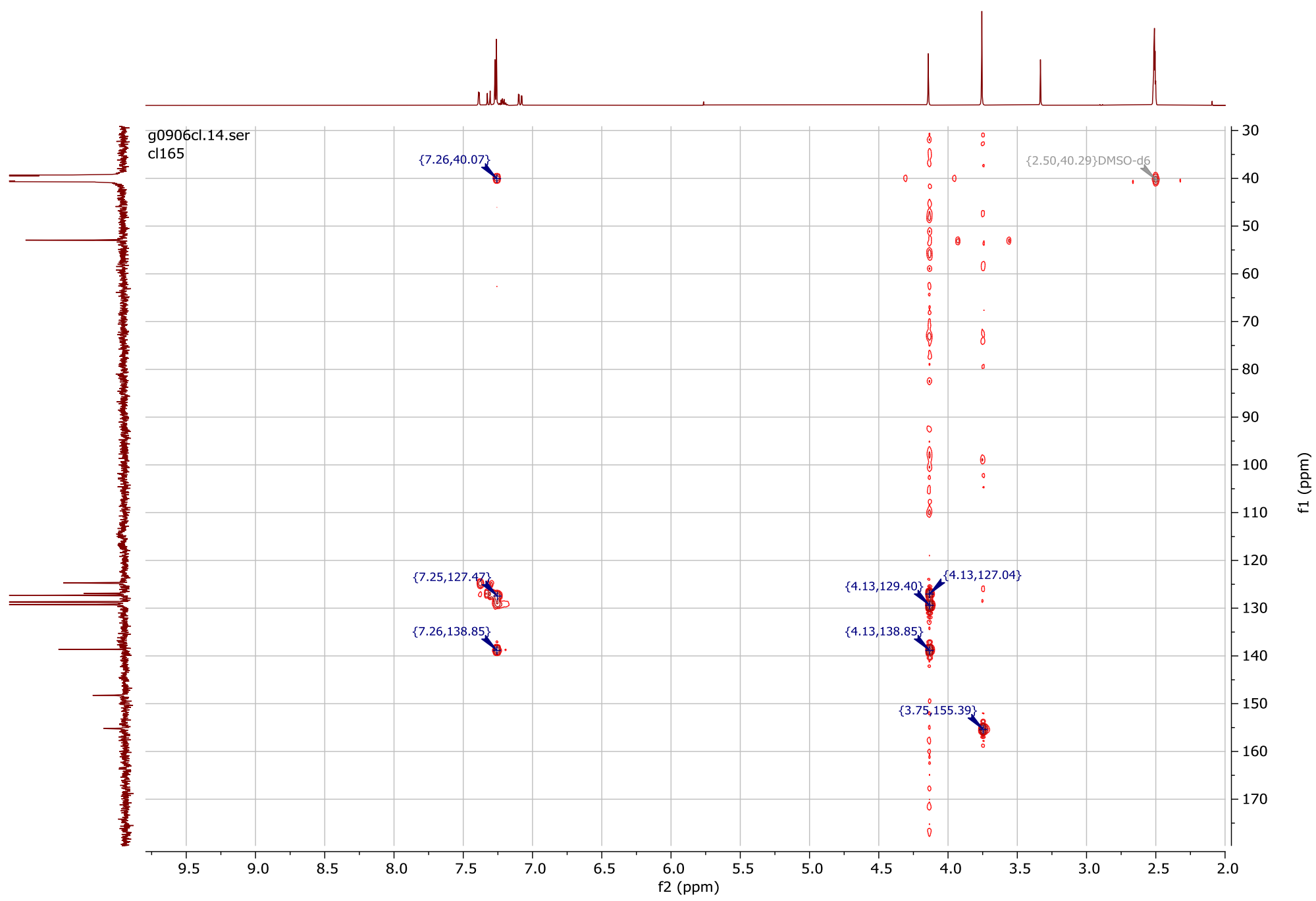

Figure S41. HMBC NMR spectrum of **5c** in $(CD_3)_2SO$.

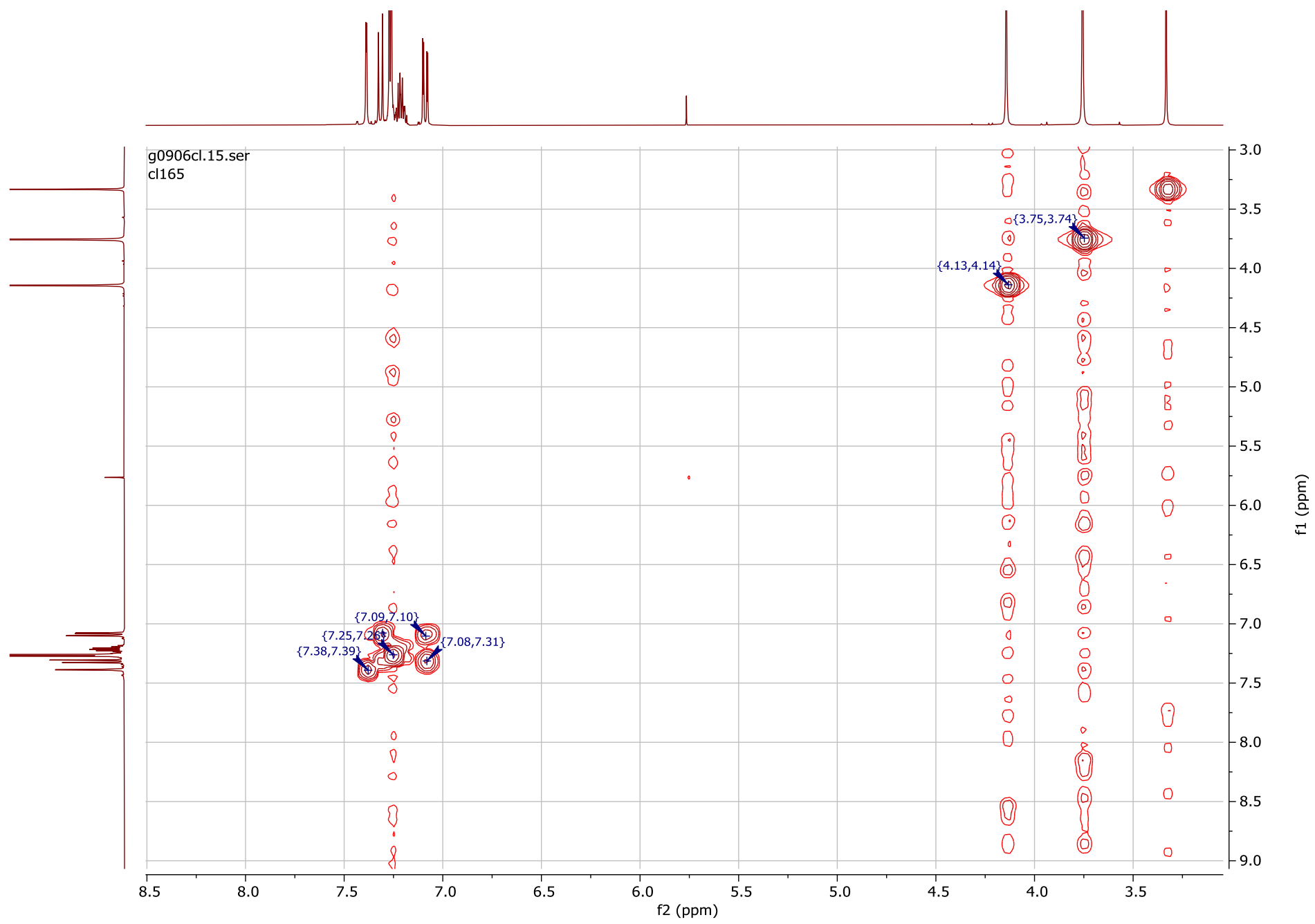

Figure S42. COSY NMR spectrum of **5c** in $(CD_3)_2SO$.

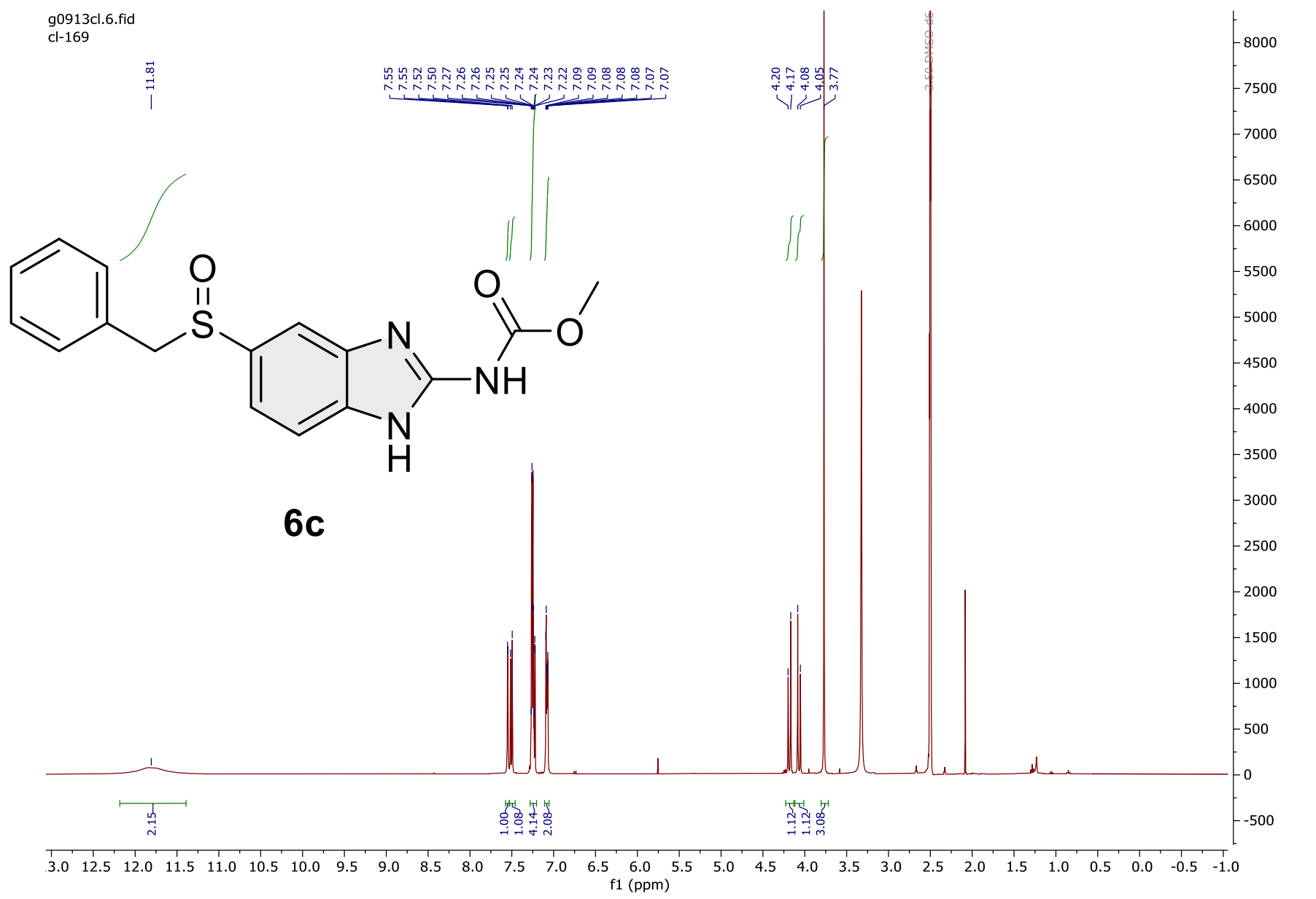

Figure S43. ^{1}H NMR spectrum of **6c** in $(CD_3)_2SO$.

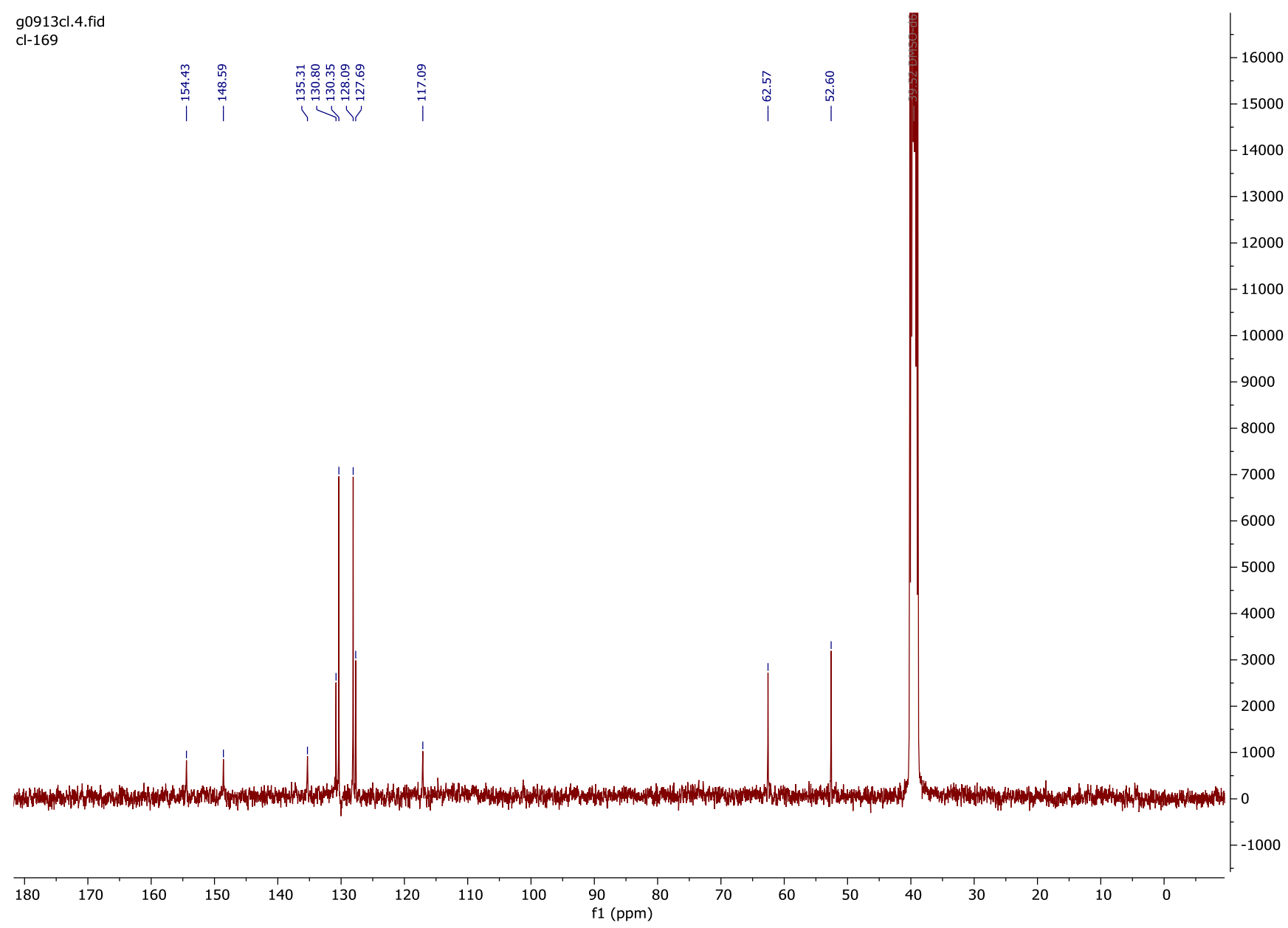

Figure S44. ^{13}C NMR spectrum of **6c** in $(CD_3)_2SO$.

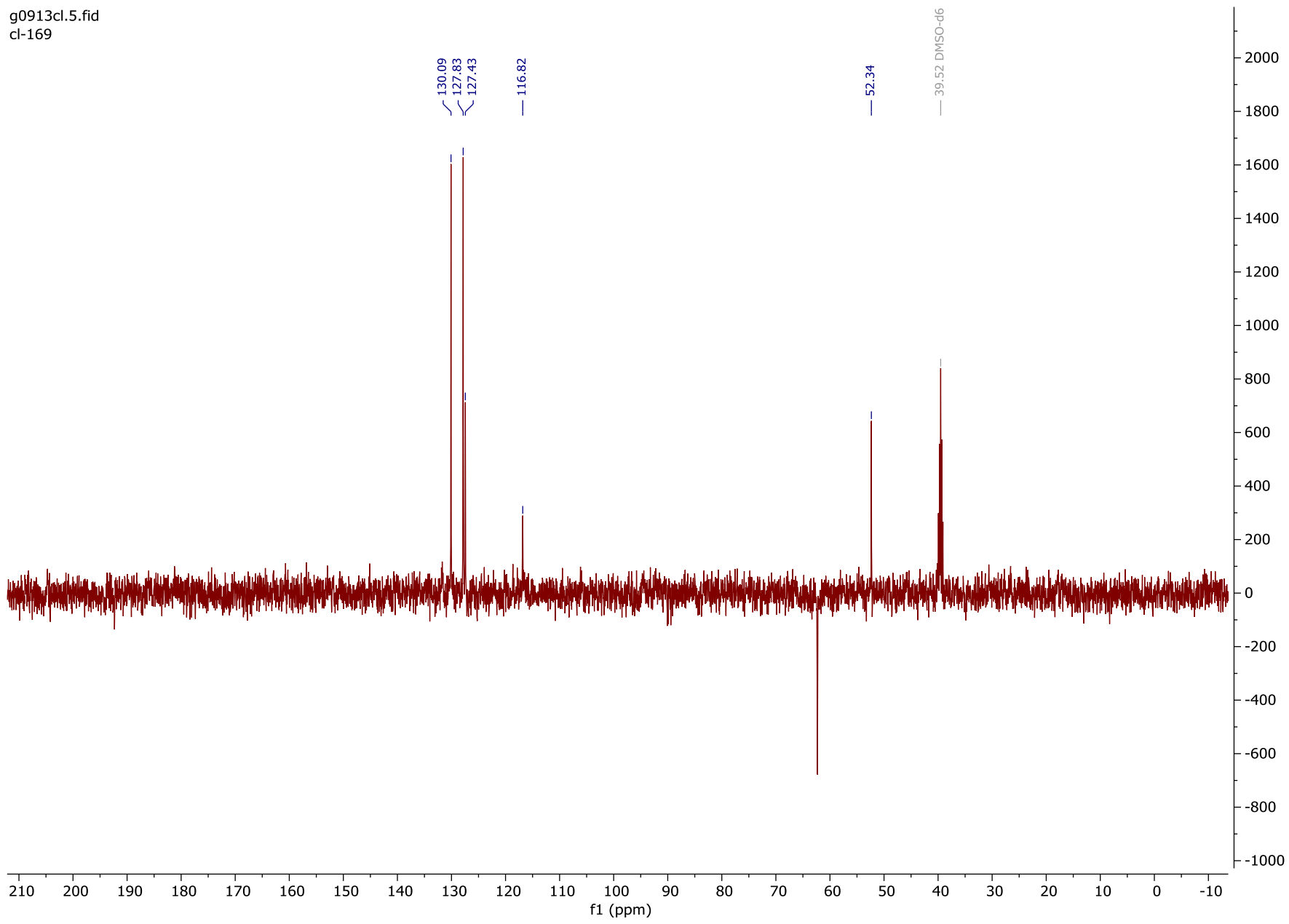

Figure S45. DEPT 135 NMR spectrum of **6c** in $(CD_3)_2SO$.

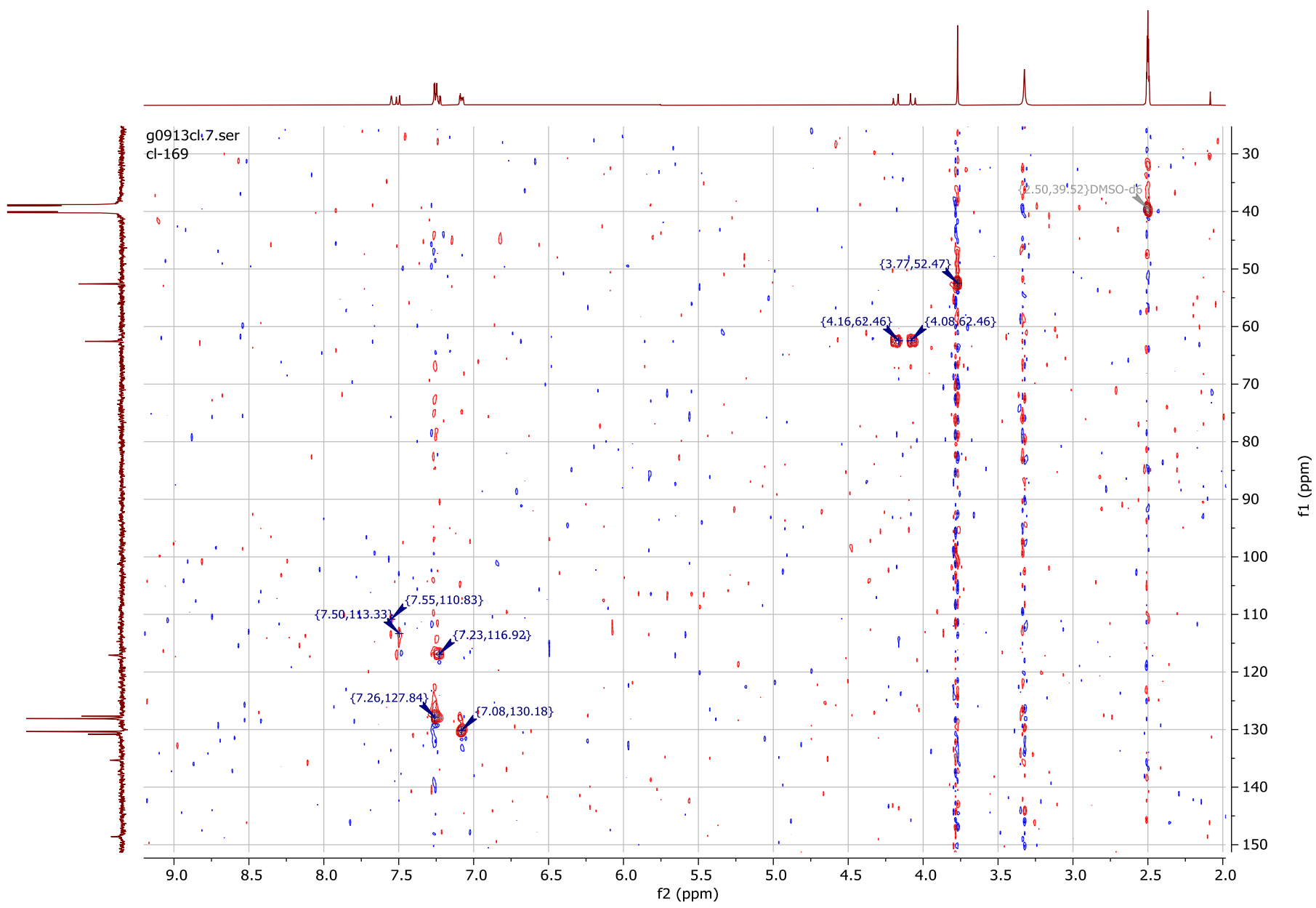

Figure S46. HSQC NMR spectrum of **6c** in $(CD_3)_2SO$.

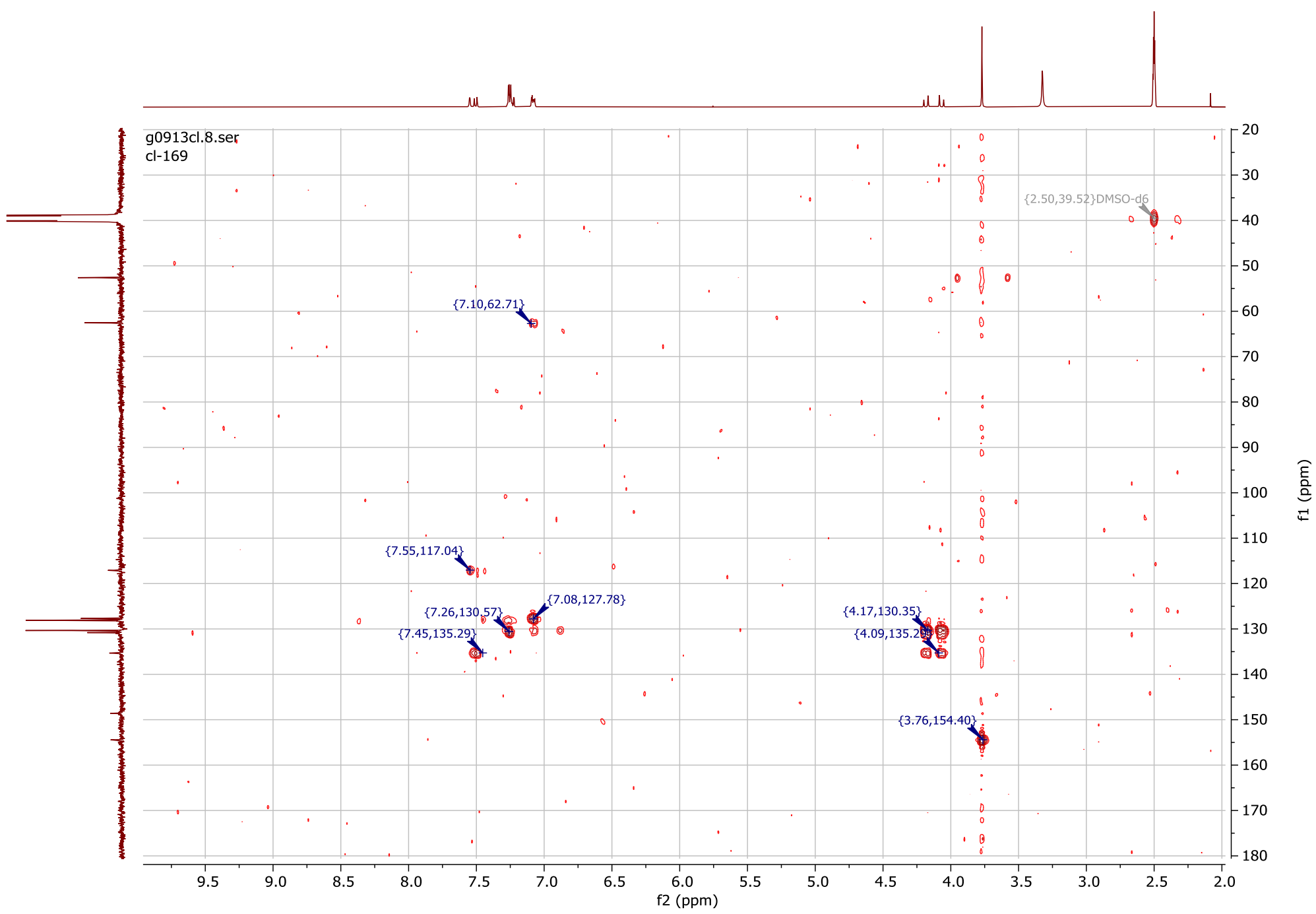

Figure S47. HMBC NMR spectrum of **6c** in $(CD_3)_2SO$.

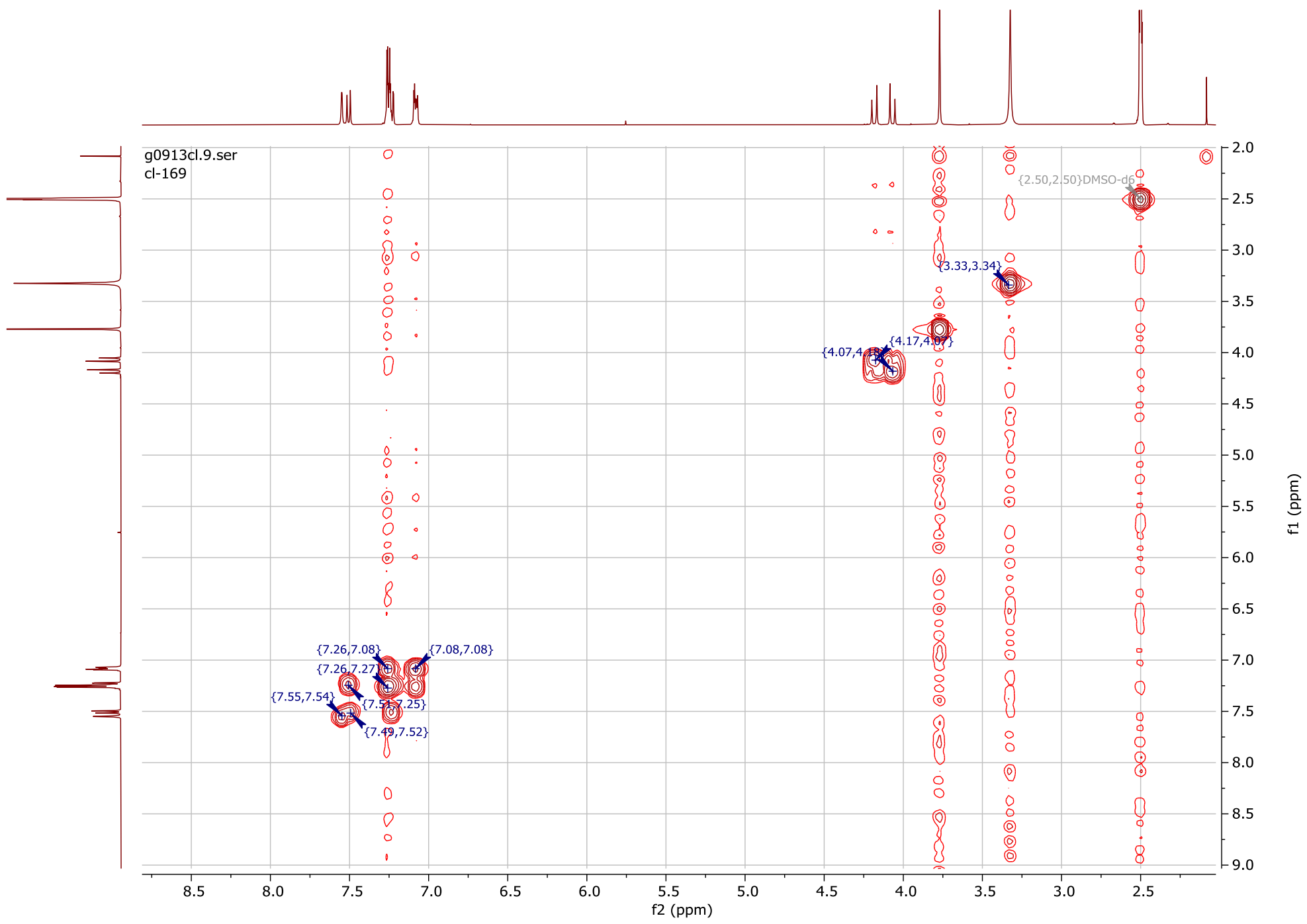

Figure S48. COSY NMR spectrum of **6c** in $(CD_3)_2SO$.

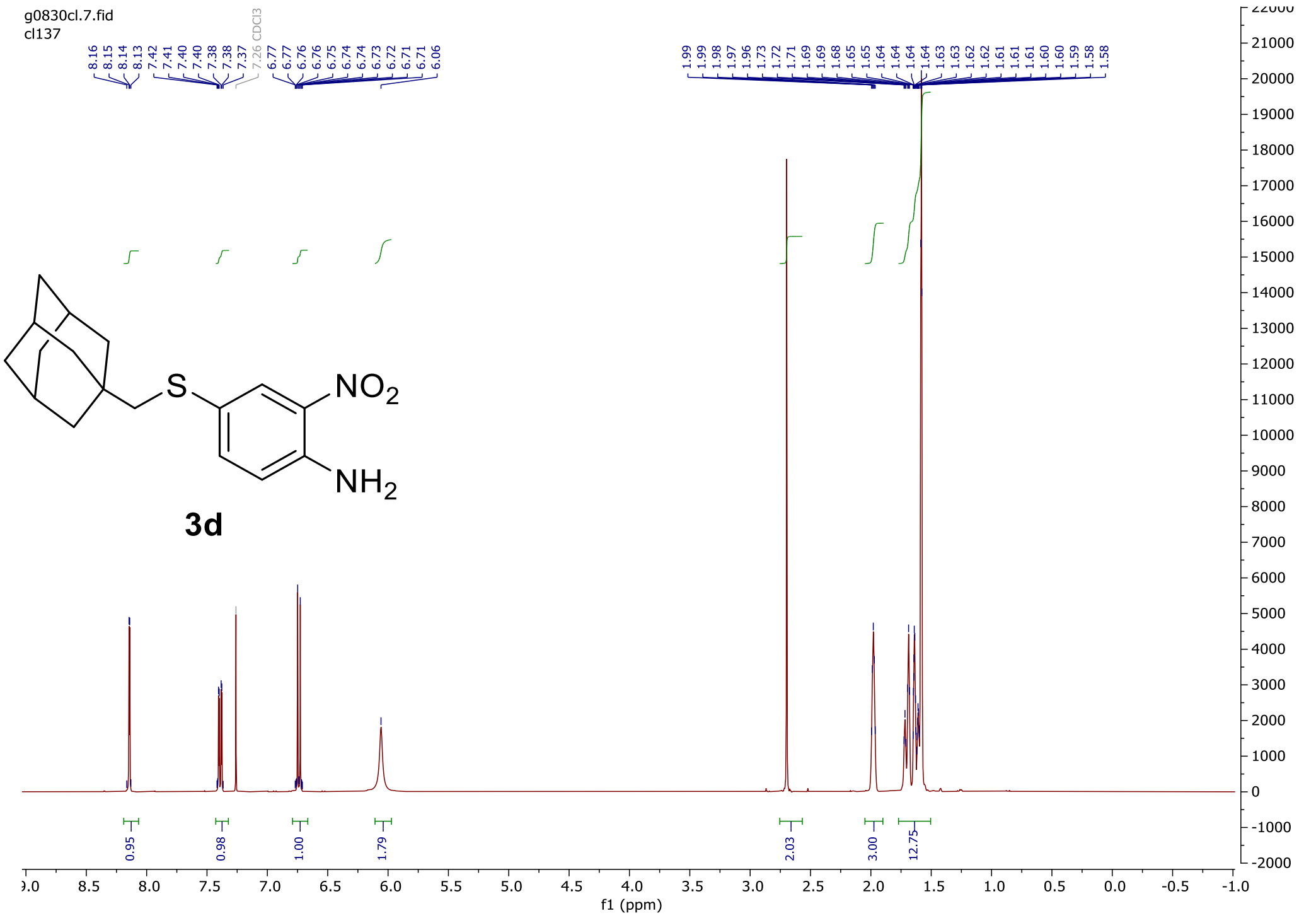

Figure S49. ^{1}H NMR spectrum of **3d** in $CDCl_3$.

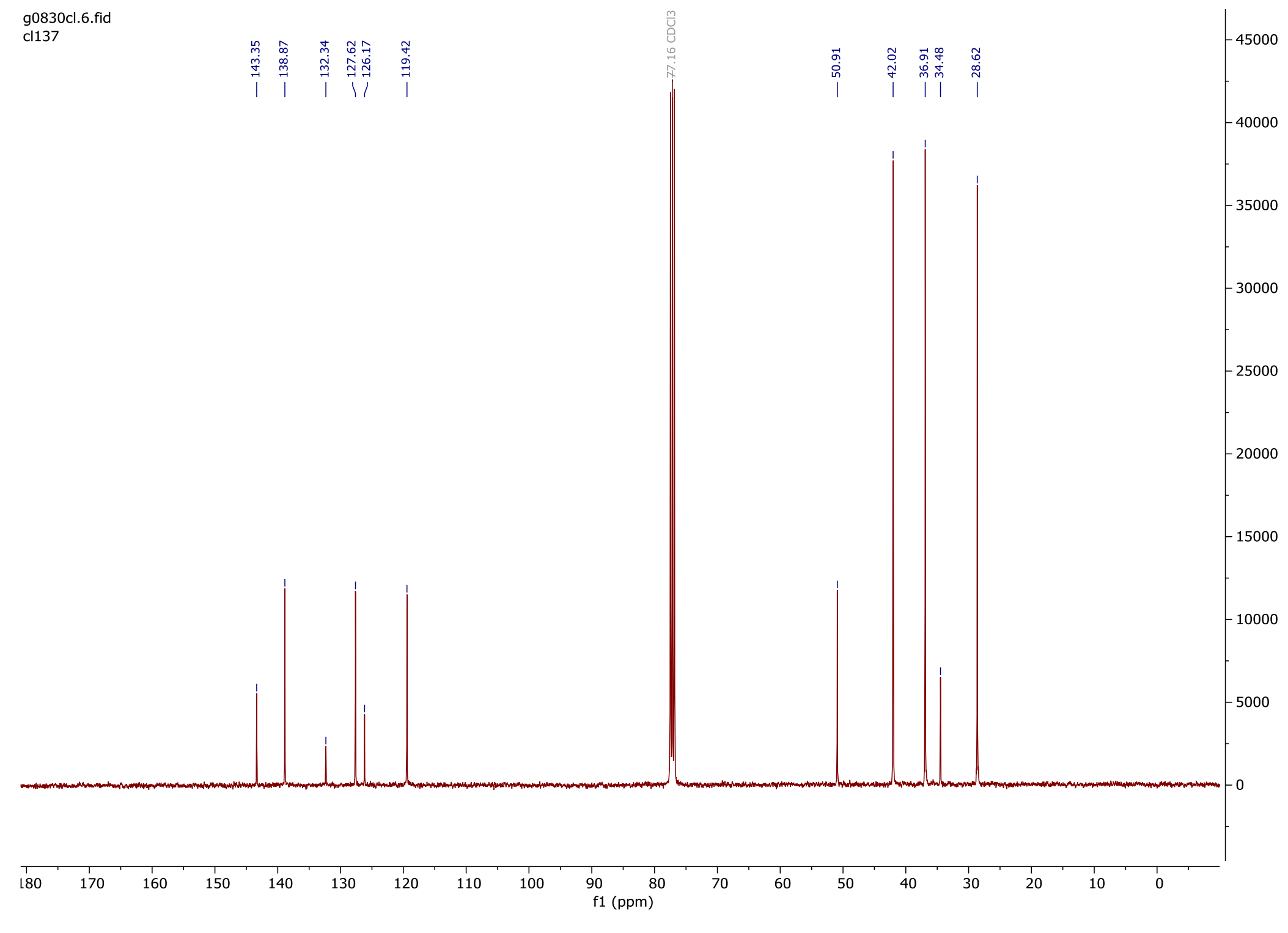

Figure S50. ^{13}C NMR spectrum of **3d** in $CDCl_3$.

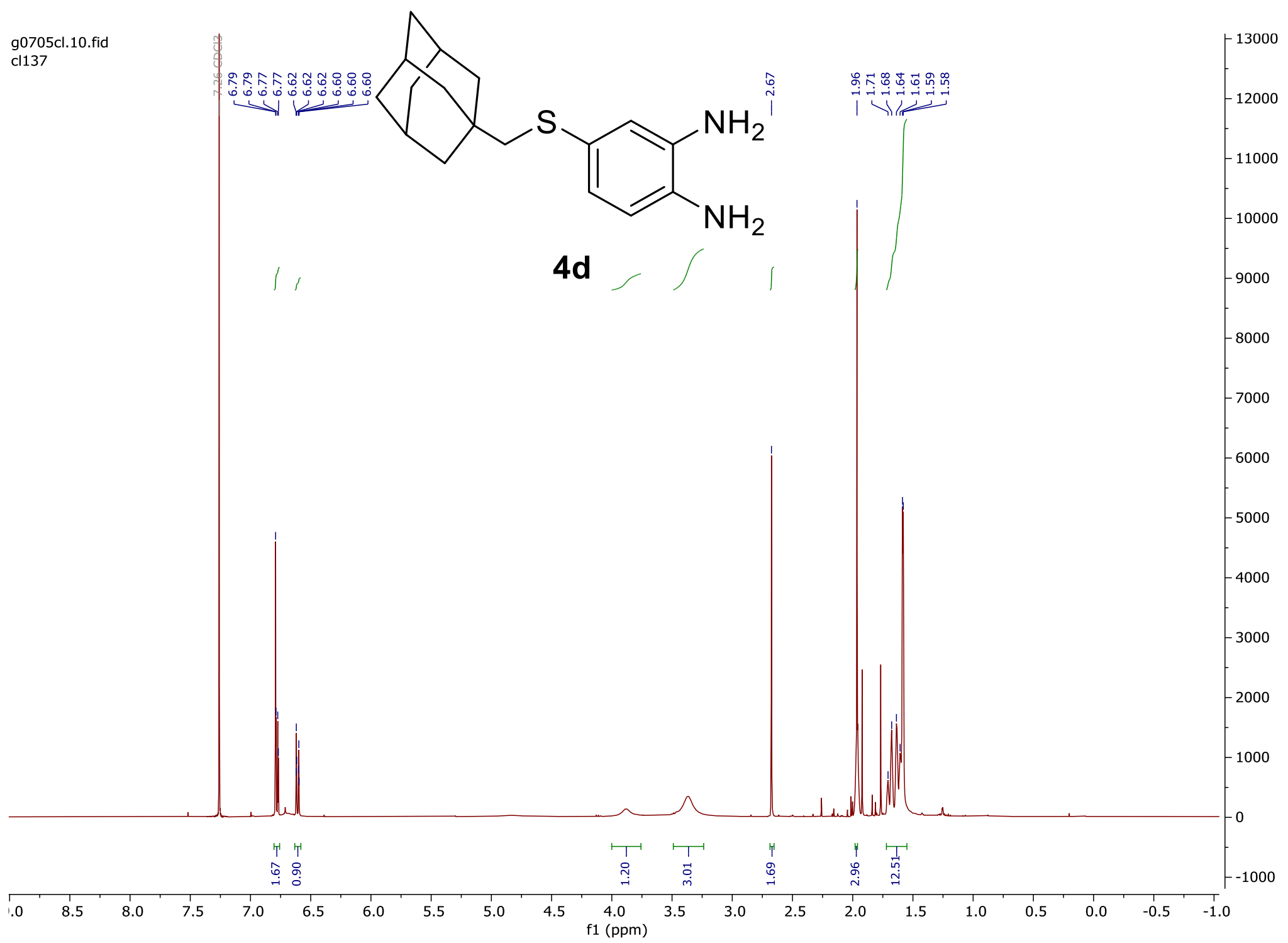

Figure S51. ^{1}H NMR spectrum of **4d** in $CDCl_3$.

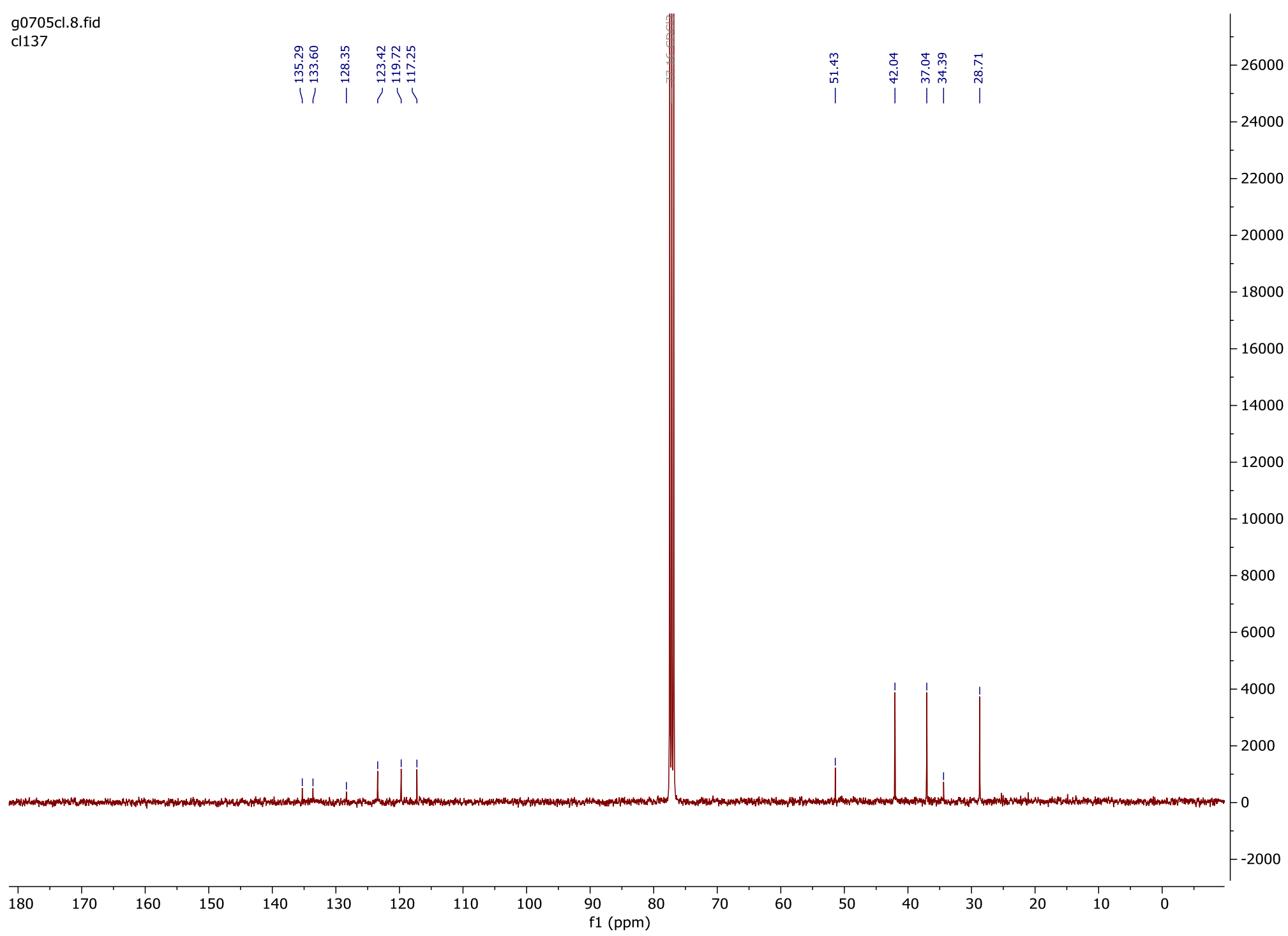

Figure S52. ^{13}C NMR spectrum of **4d** in $CDCl_3$.

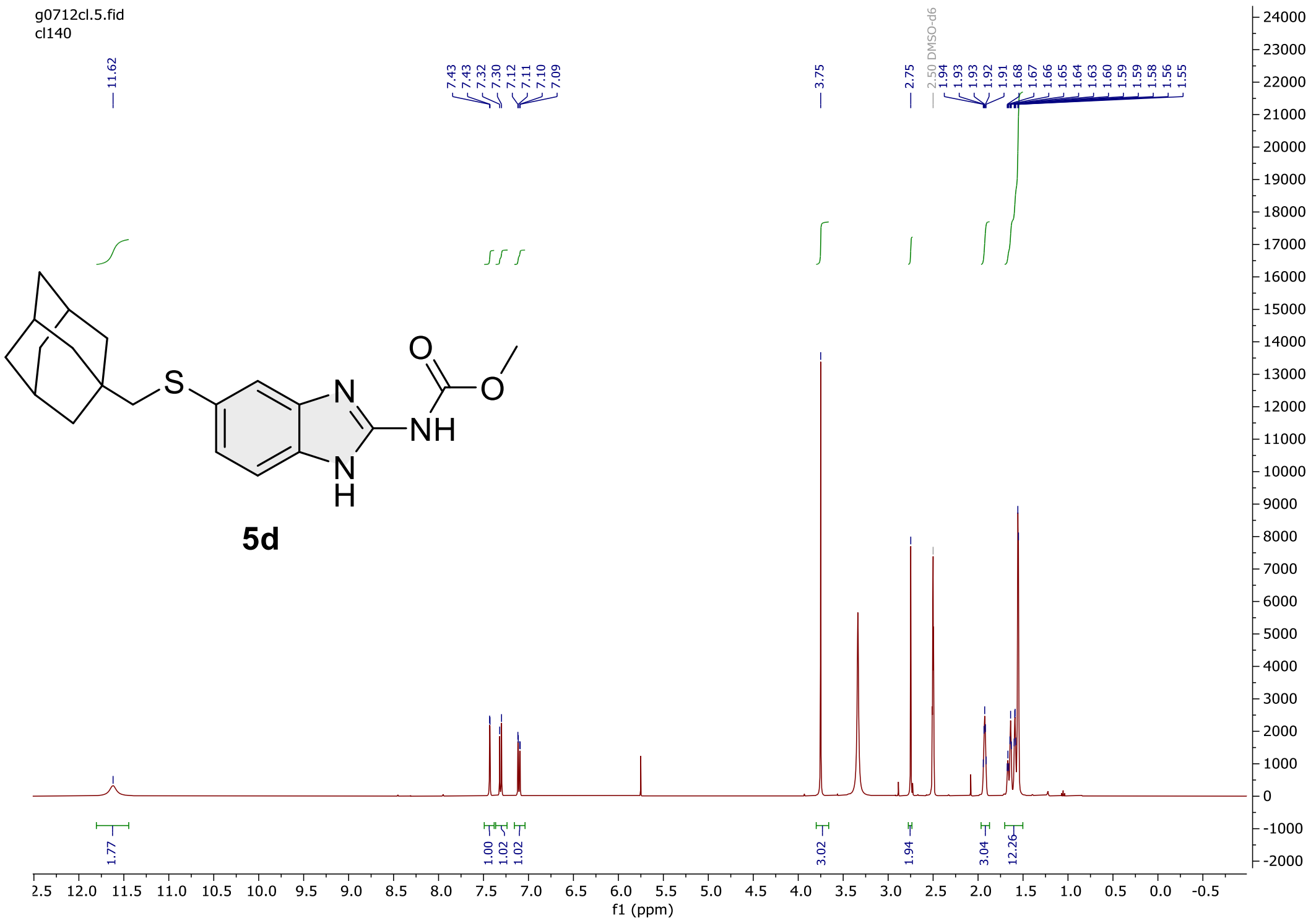

Figure S53. ^{1}H NMR spectrum of **5d** in $(CD_3)_2SO$.

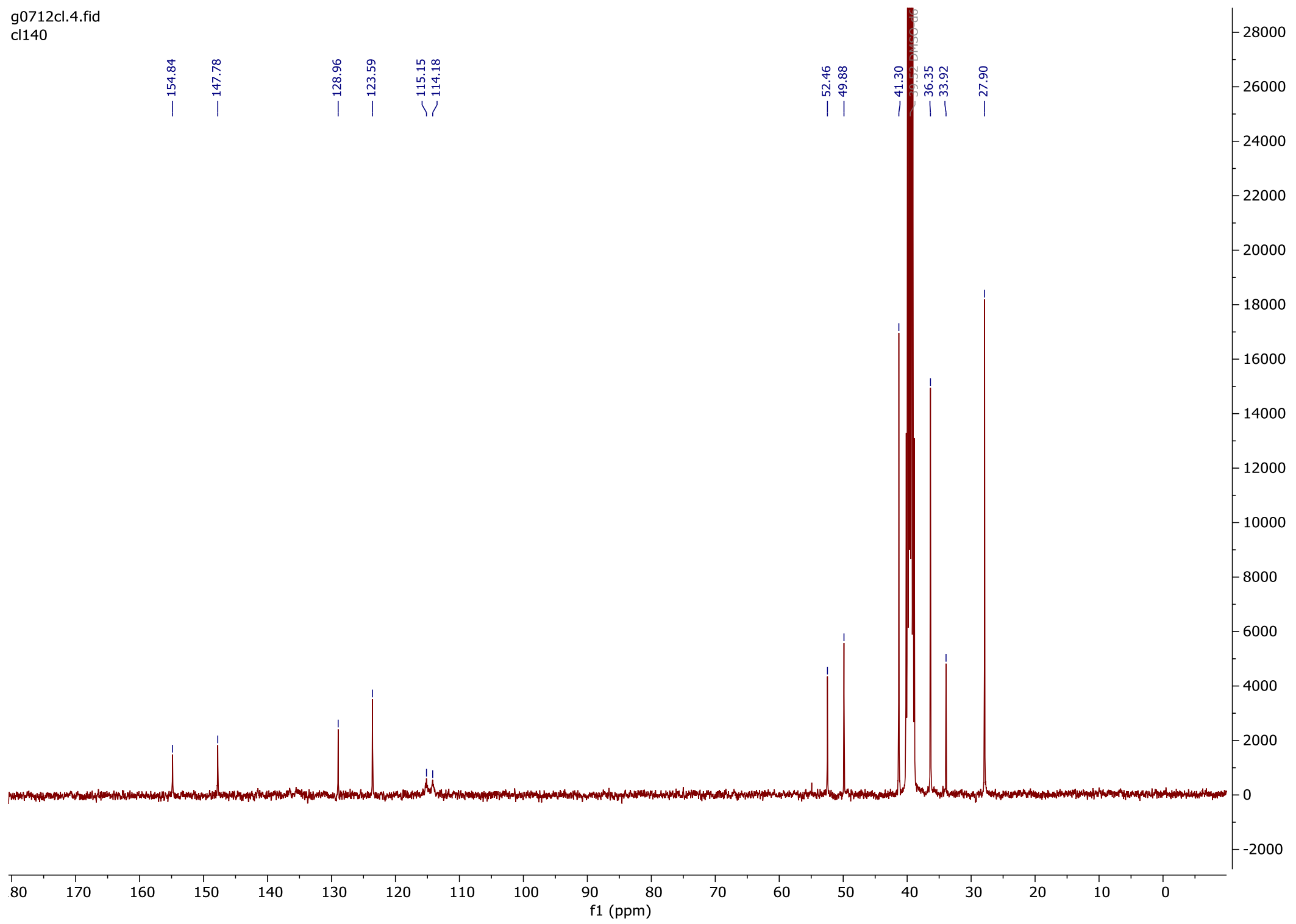

Figure S54. ^{13}C NMR spectrum of **5d** in $(CD_3)_2SO$.

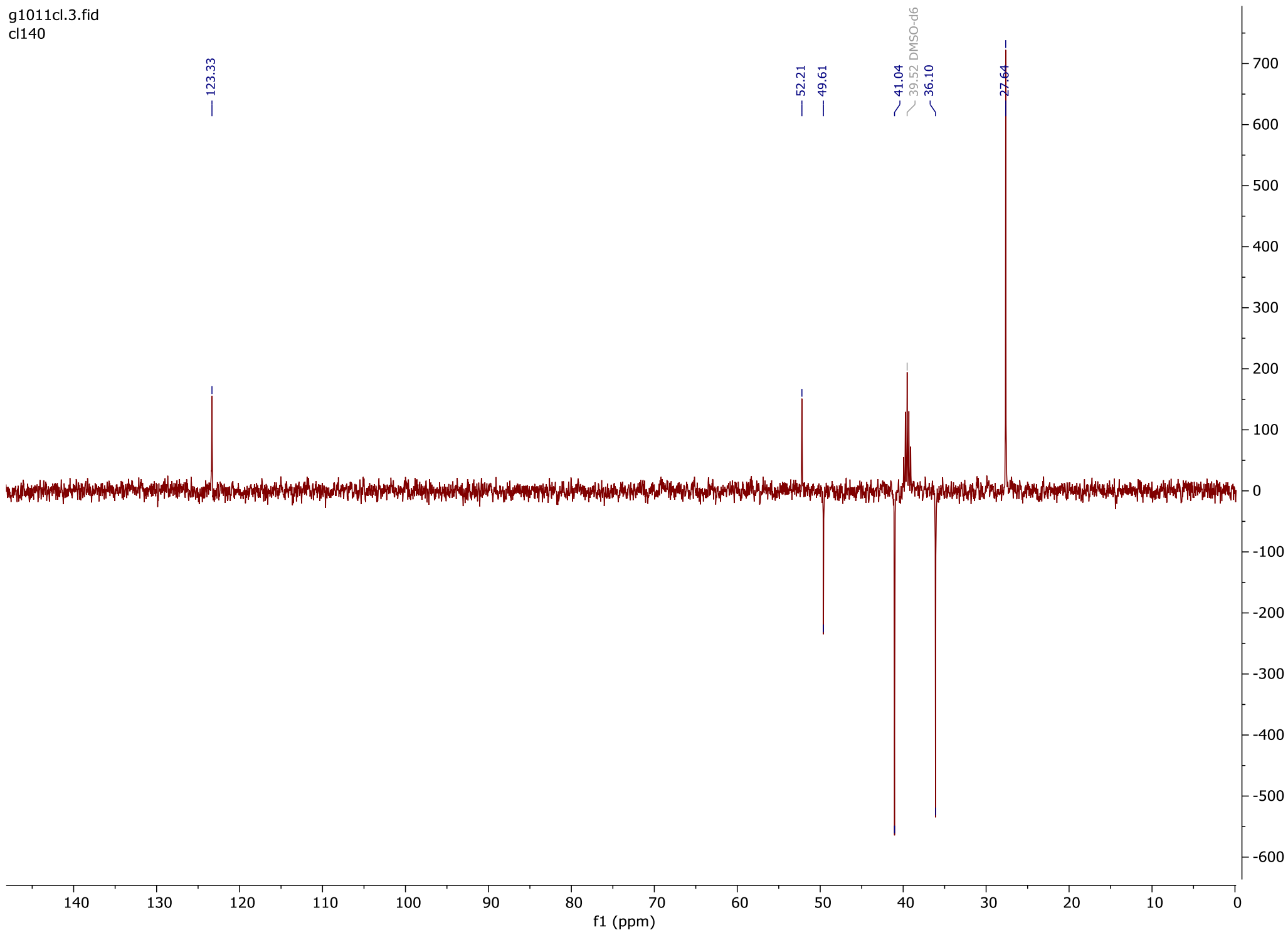

Figure S55. DEPT 135 NMR spectrum of **5d** in $(CD_3)_2SO$.

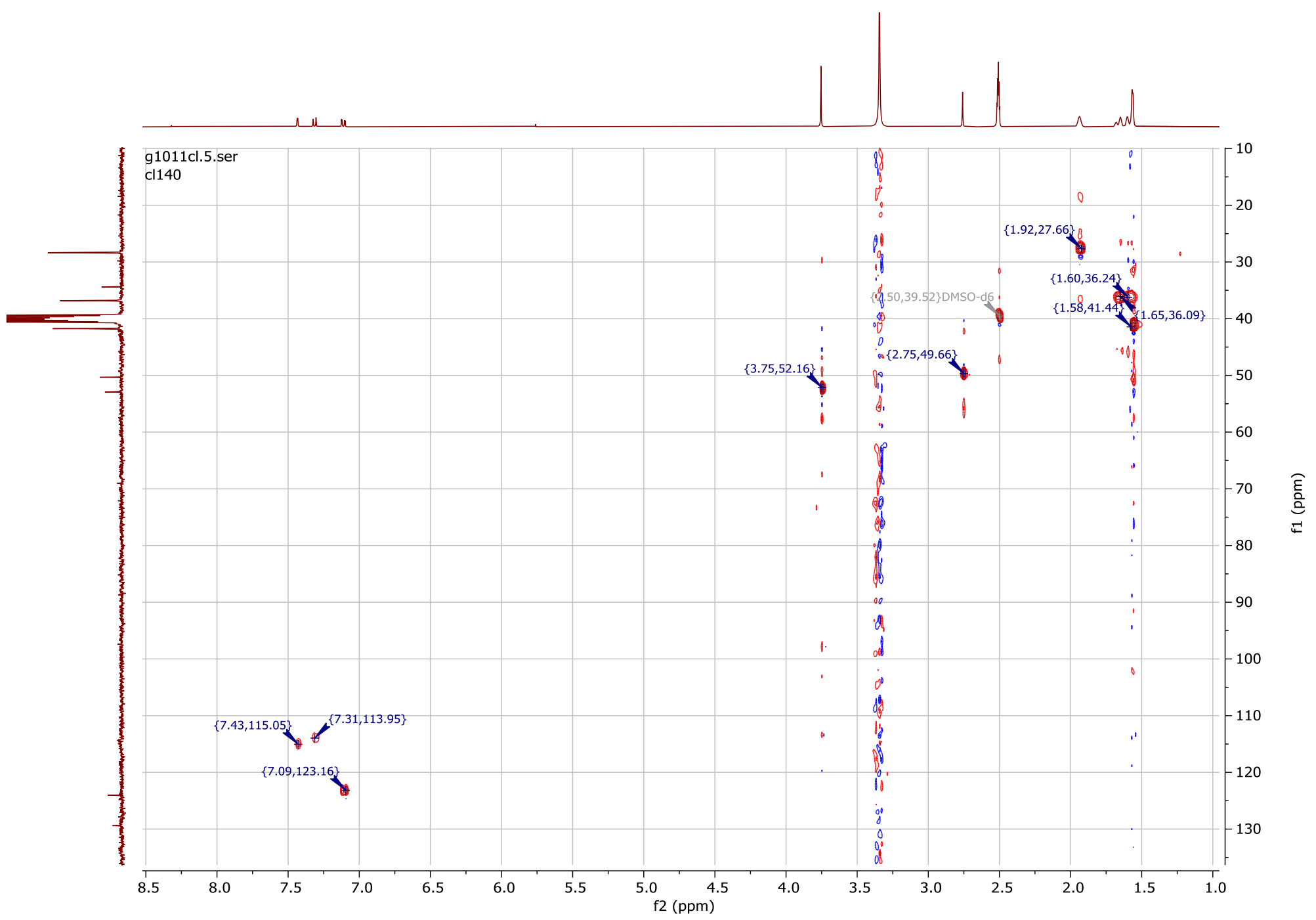

Figure S56. HSQC NMR spectrum of **5d** in $(CD_3)_2SO$.

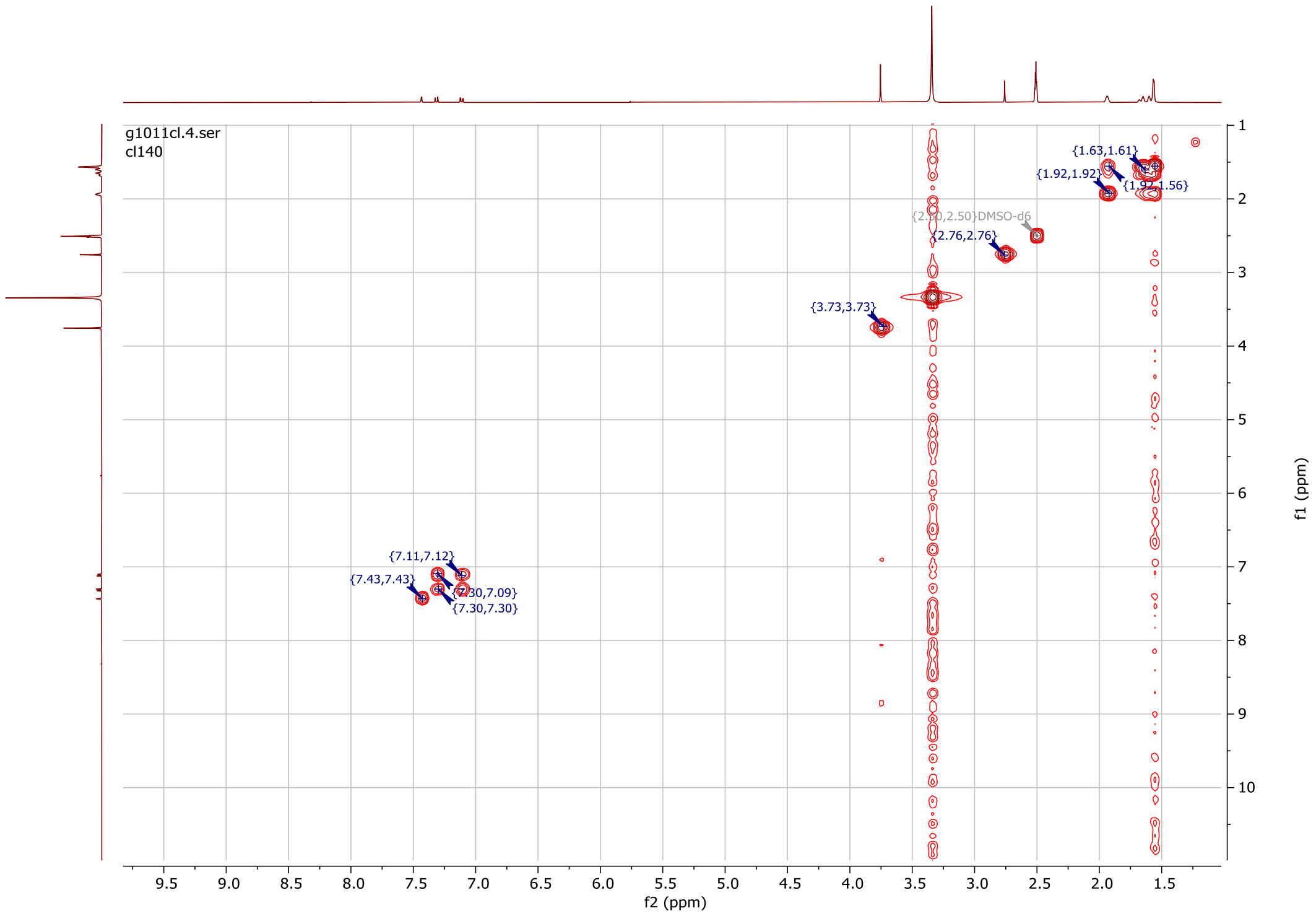

Figure S57. COSY NMR spectrum of **5d** in $(CD_3)_2SO$.

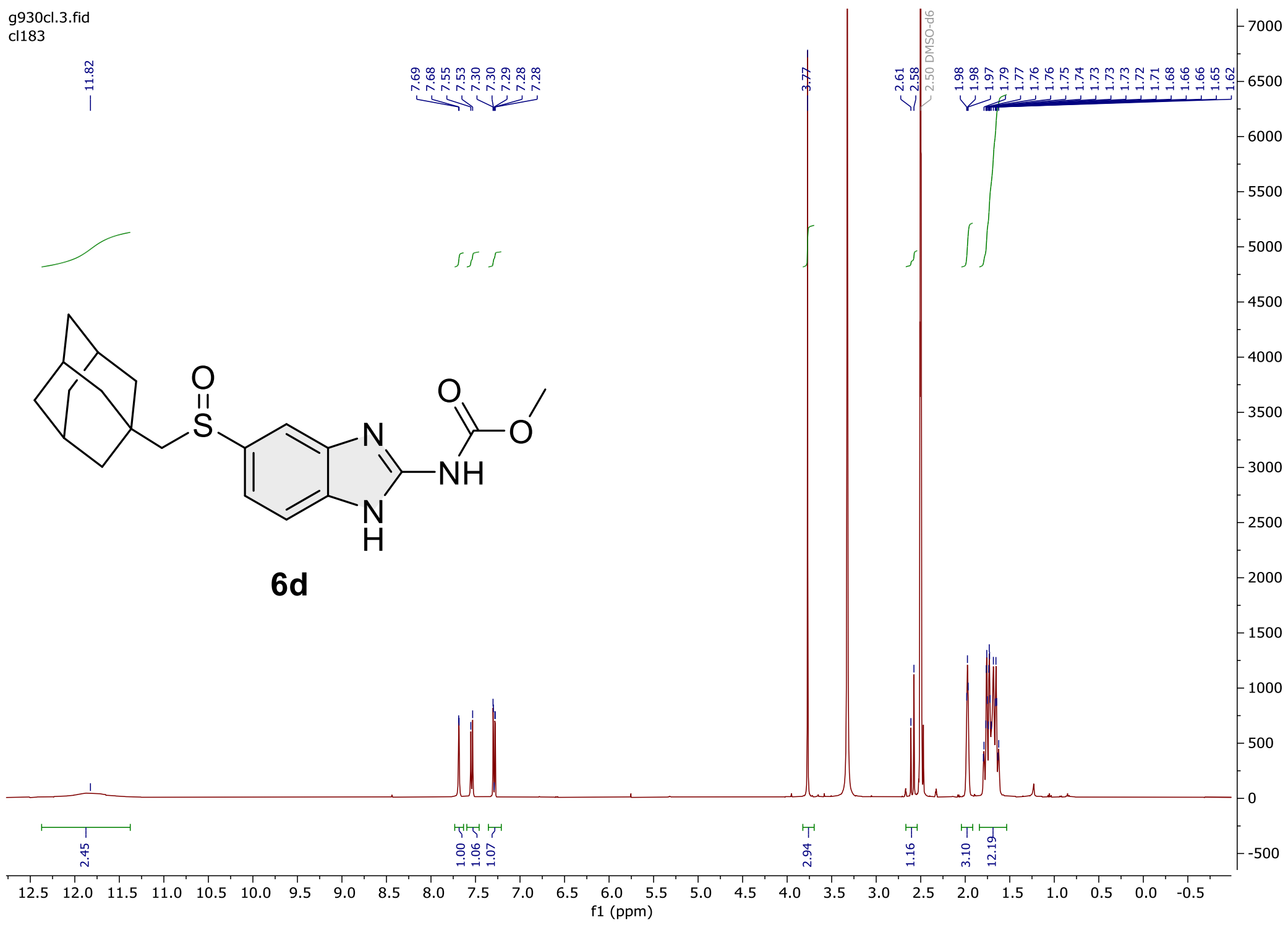

Figure S58. ^1H NMR spectrum of **6d** in $(CD_3)_2SO$.

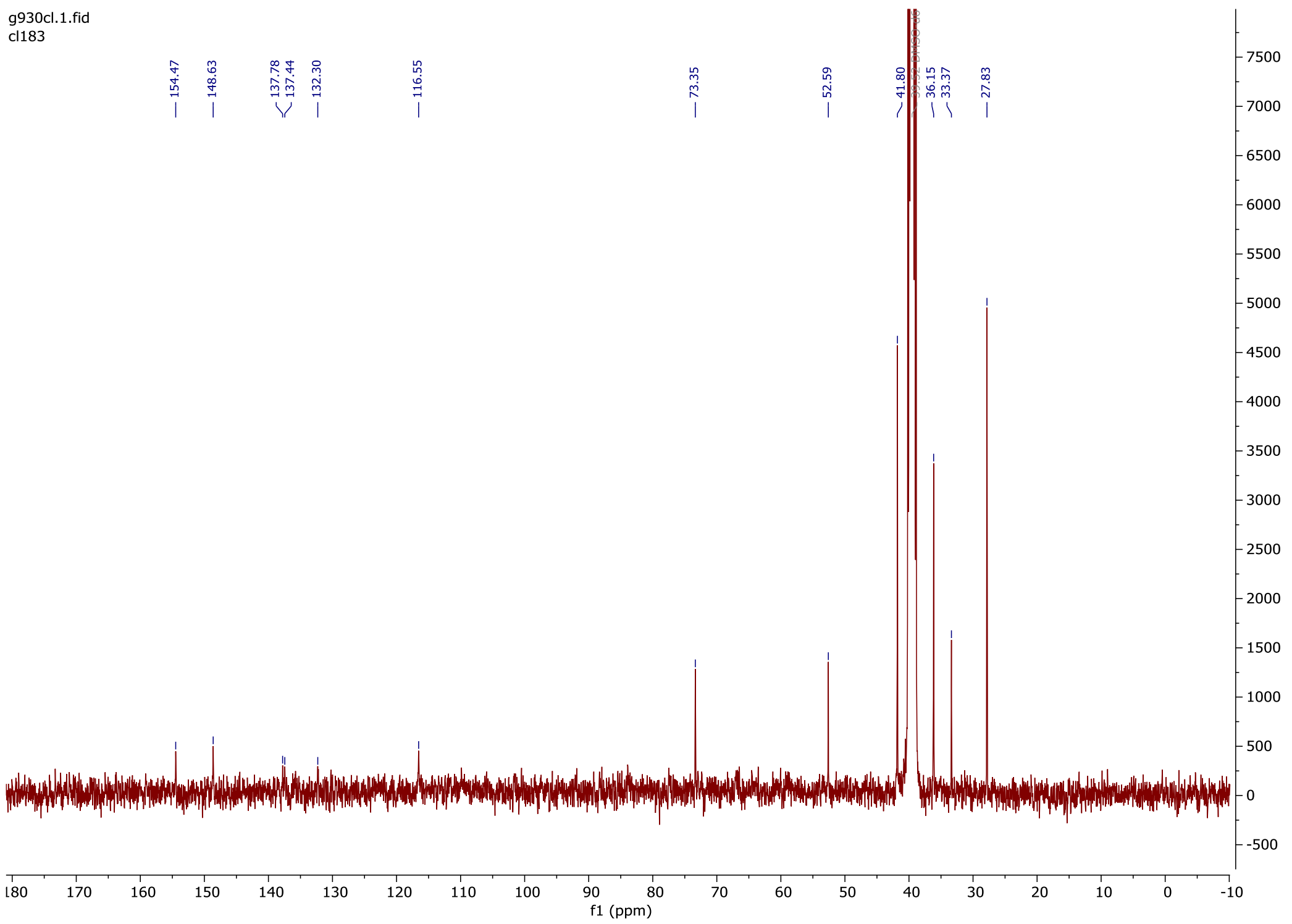

Figure S59. ^{13}C NMR spectrum of **6d** in $(CD_3)_2SO$.

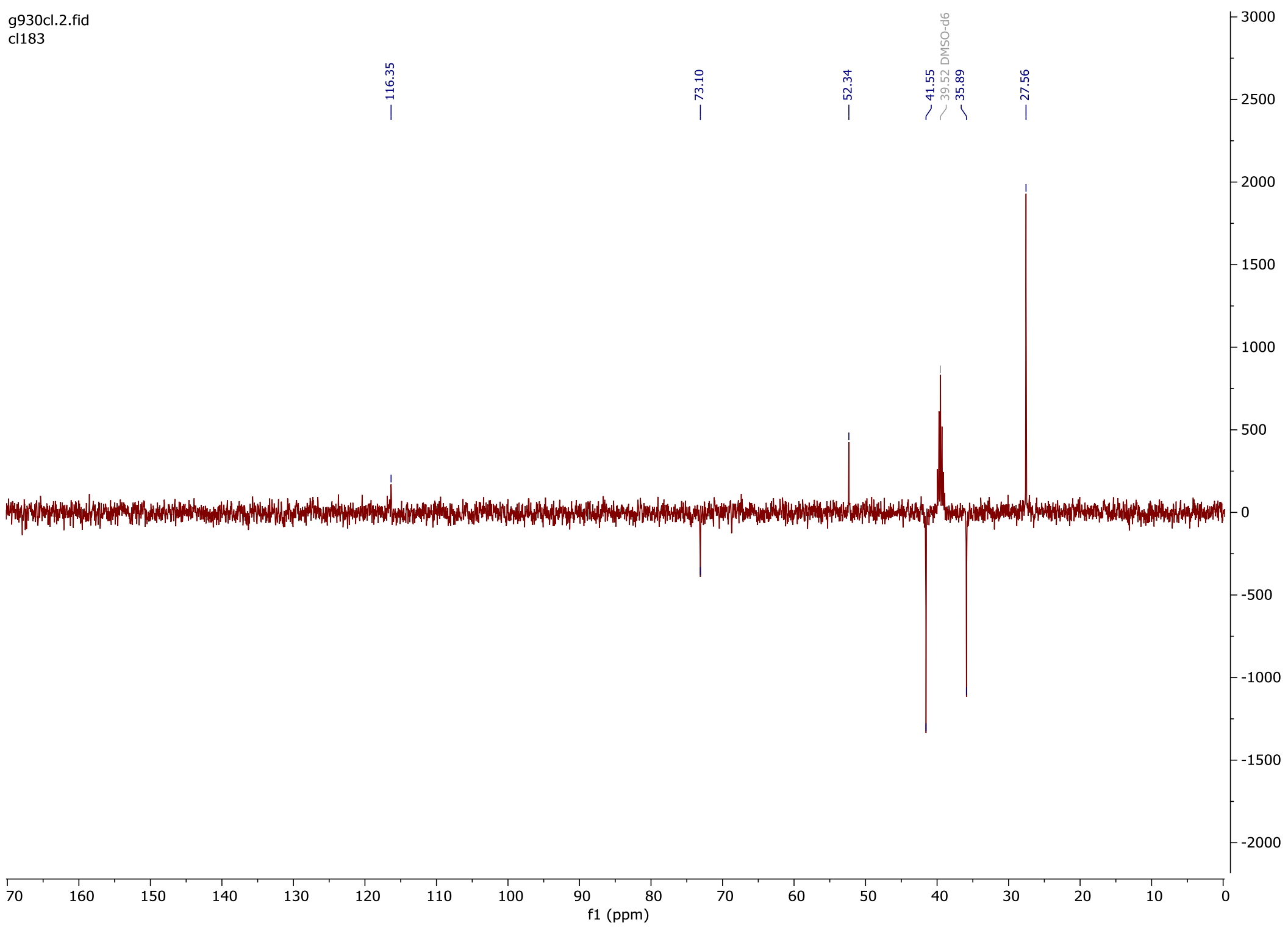

Figure S60. DEPT 135 NMR spectrum of **6d** in $(CD_3)_2SO$.

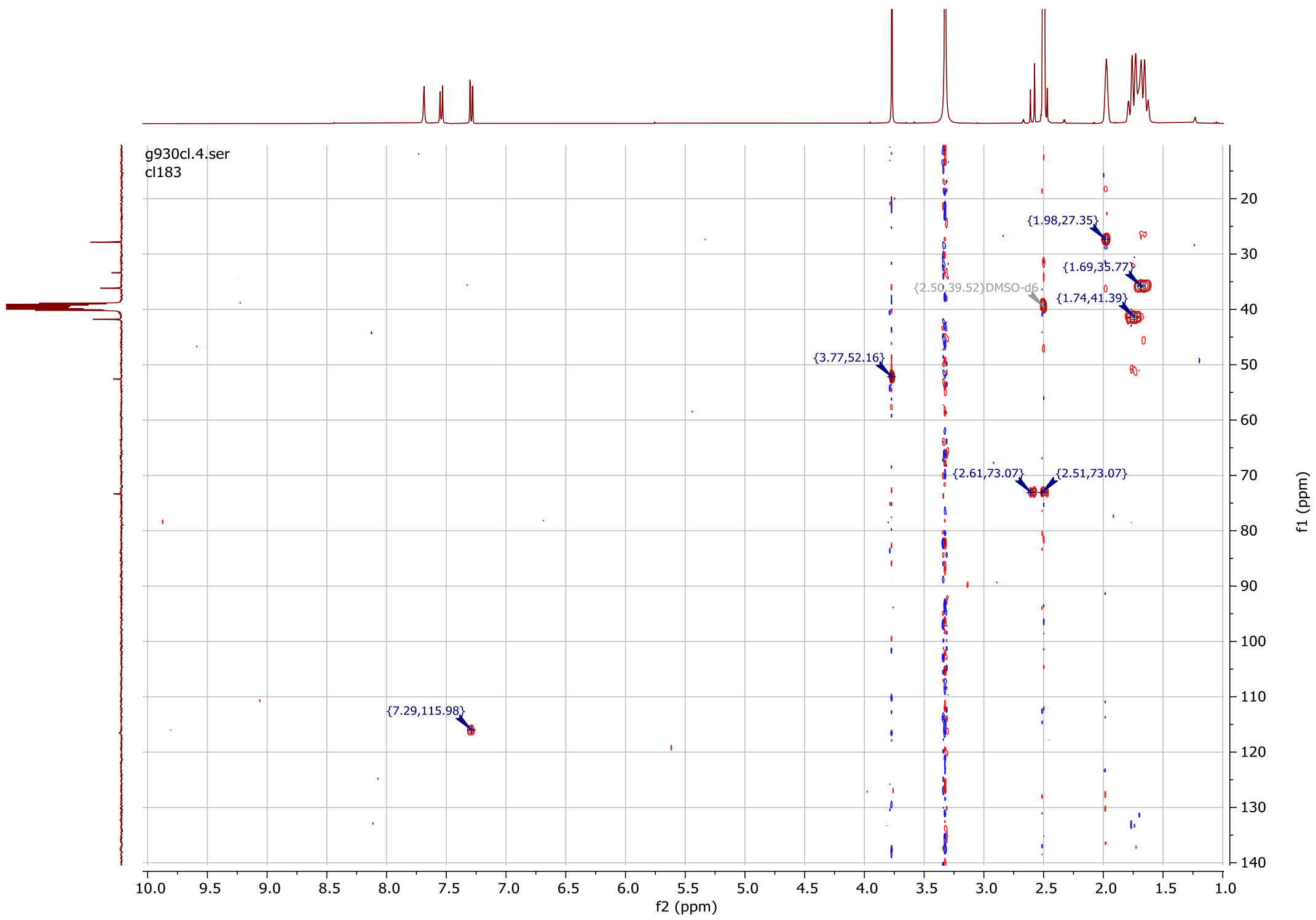

Figure S61. HSQC NMR spectrum of **6d** in $(CD_3)_2SO$.

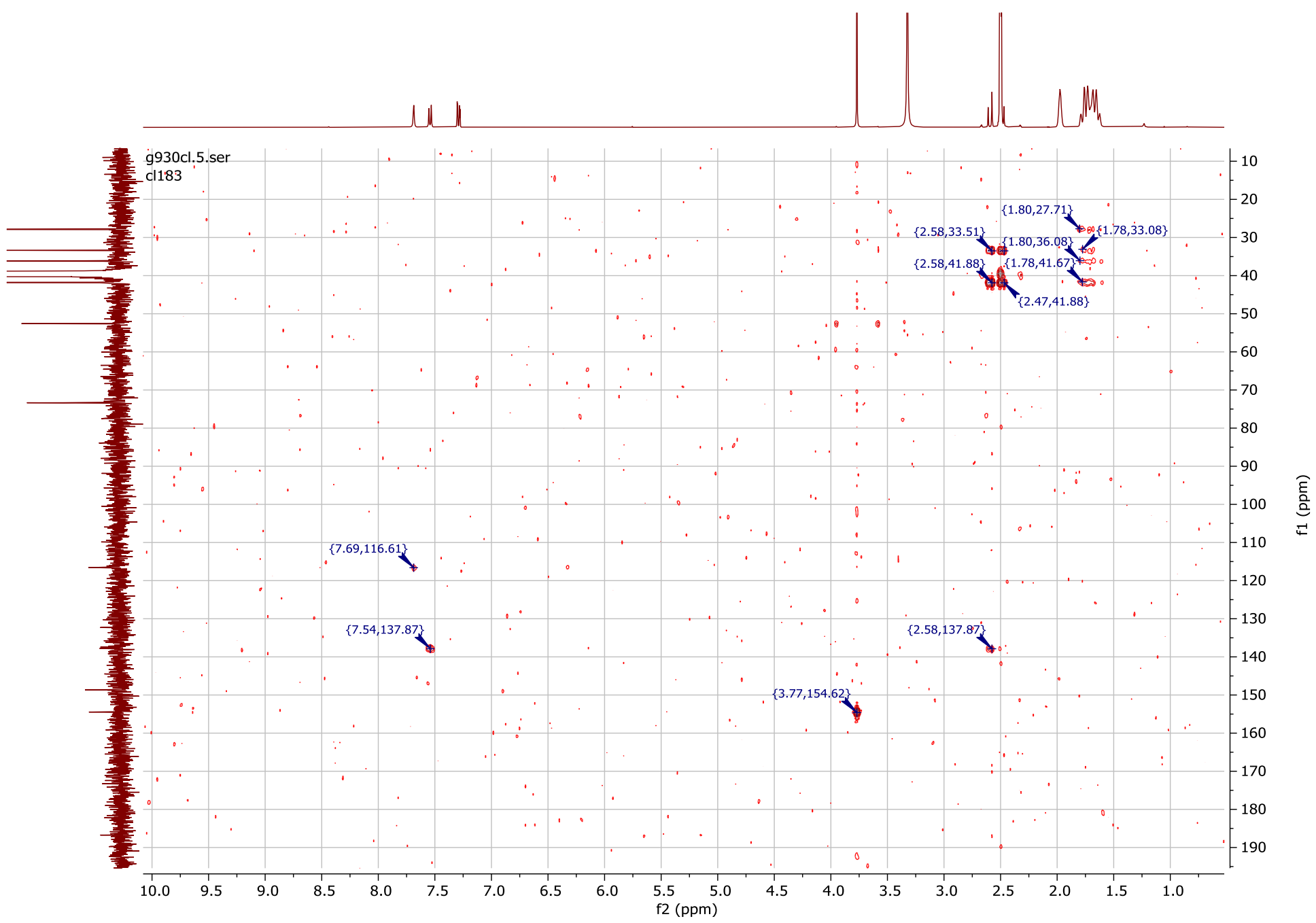

Figure S62. HMBC NMR spectrum of **6d** in $(CD_3)_2SO$.

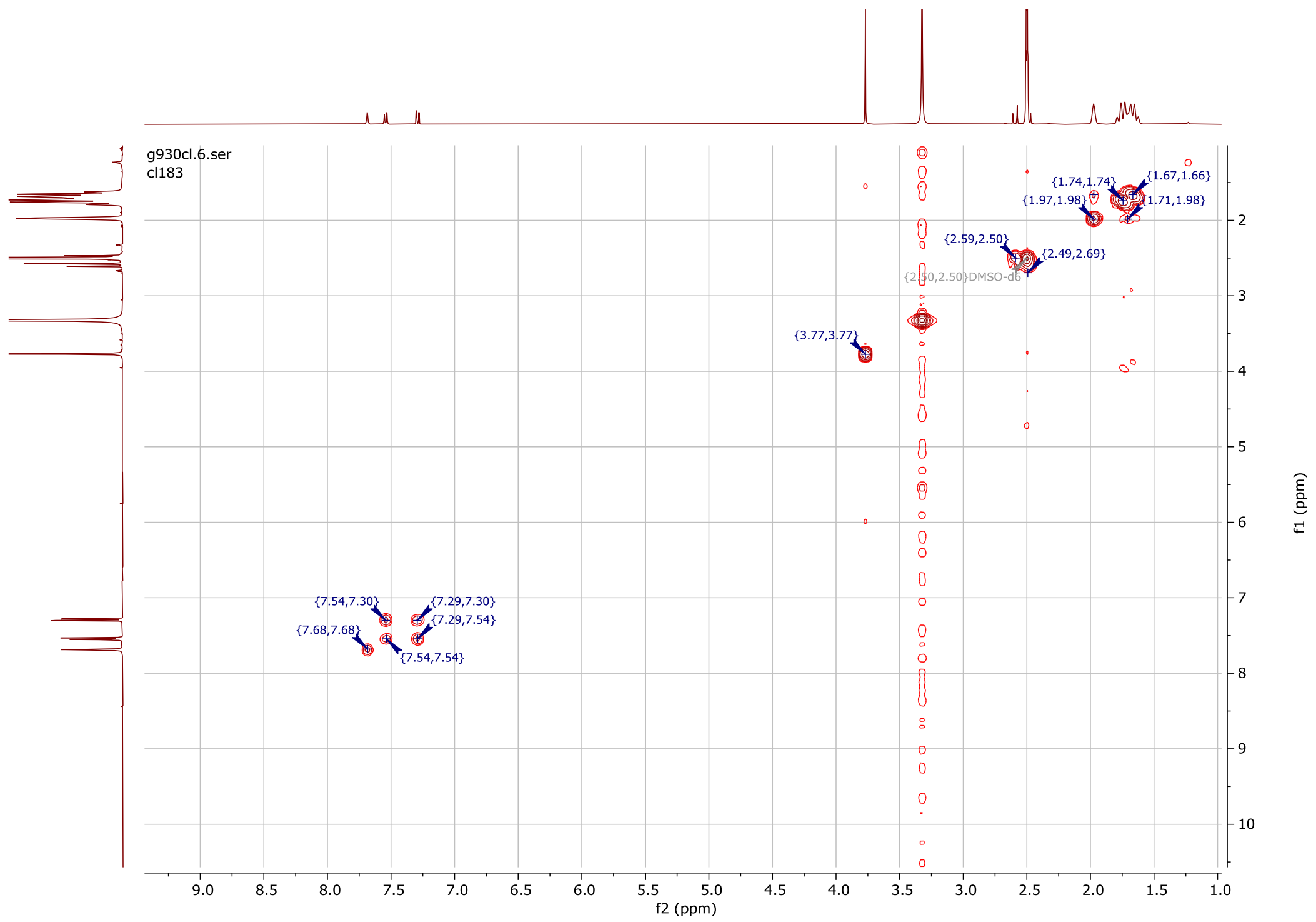

Figure S63. COSY NMR spectrum of **6d** in $(CD_3)_2SO$.

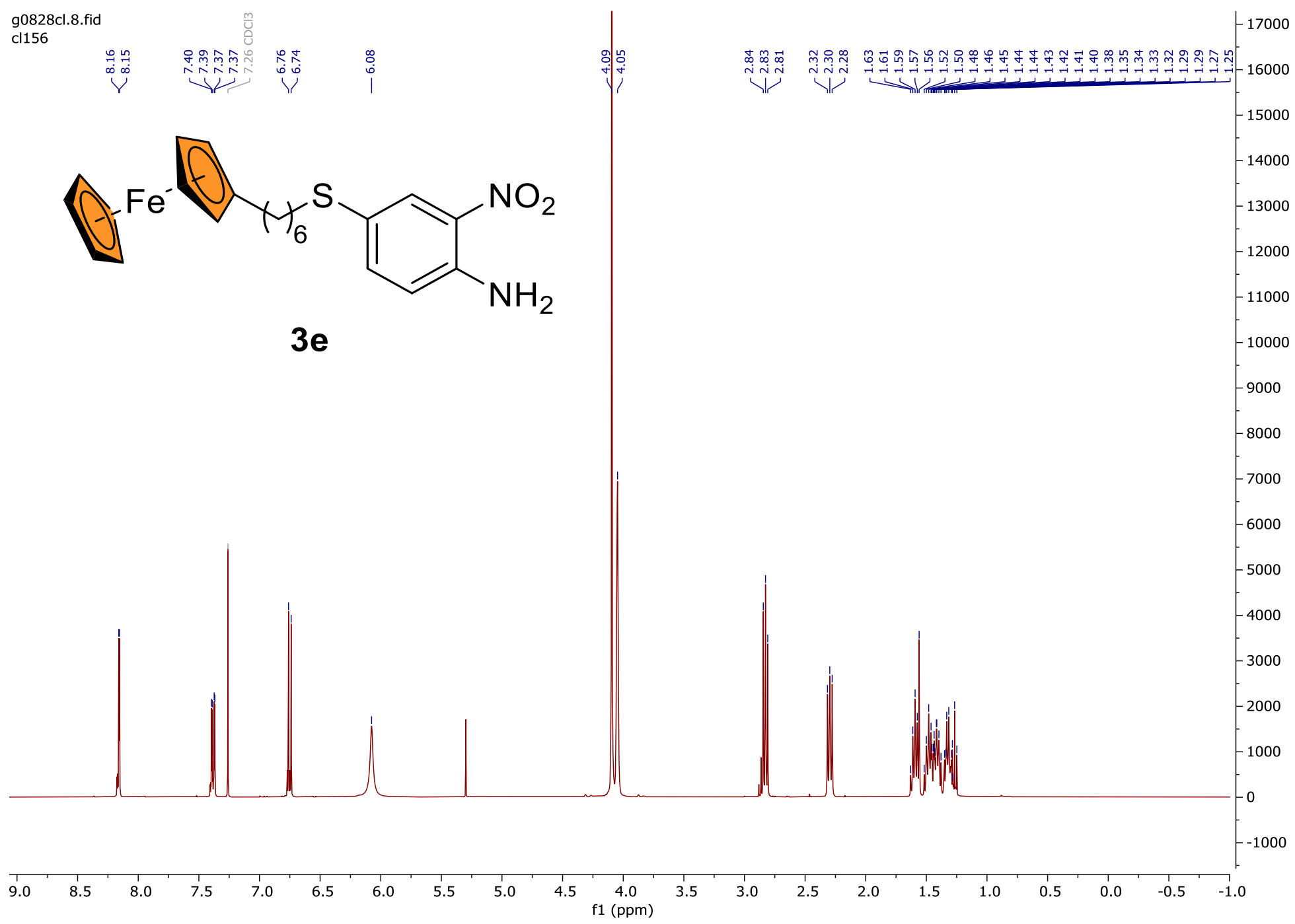

Figure S64. ^1H NMR spectrum of **3e** in $CDCl_3$.

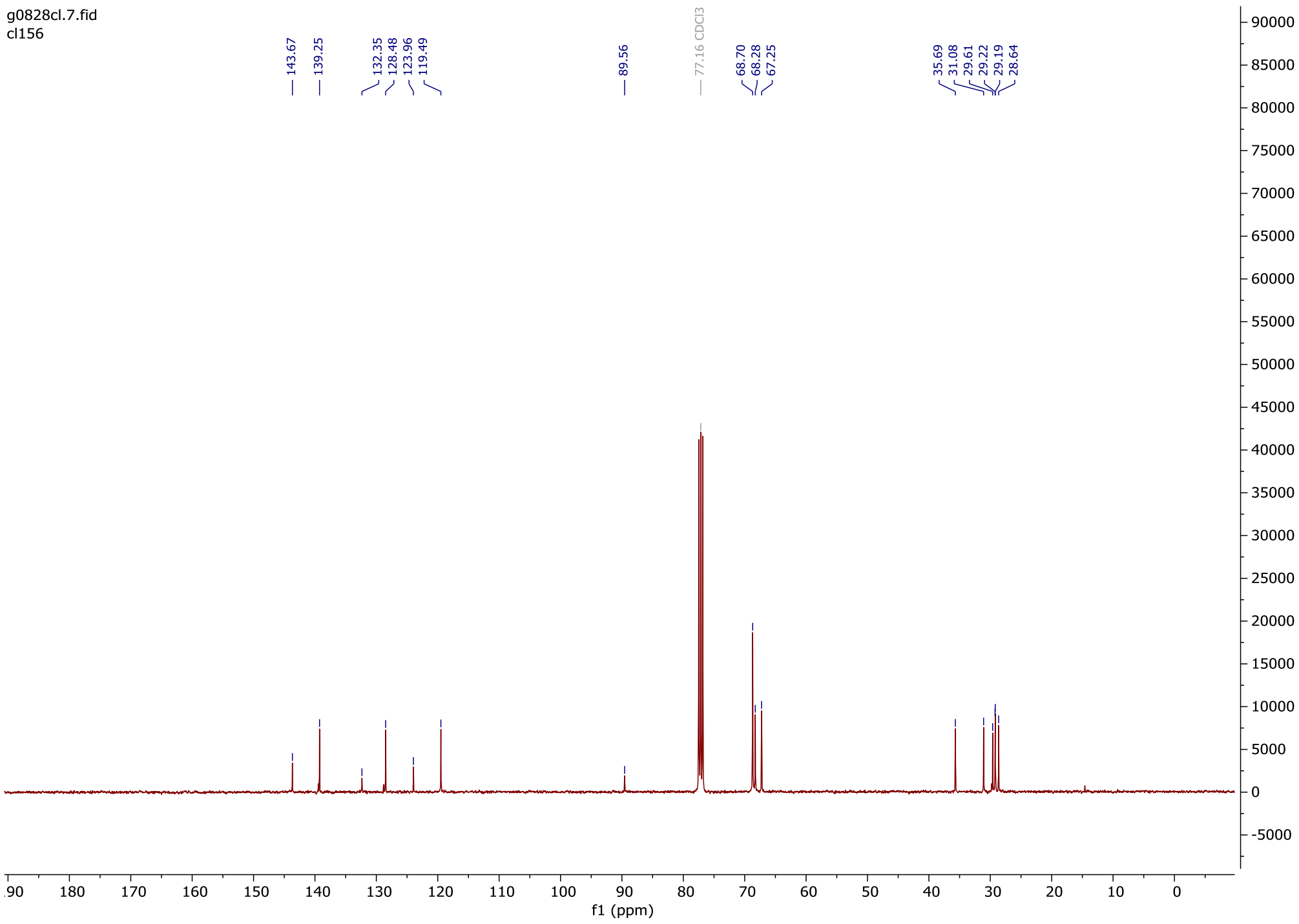

Figure S65. ^{13}C NMR spectrum of **3e** in $CDCl_3$.

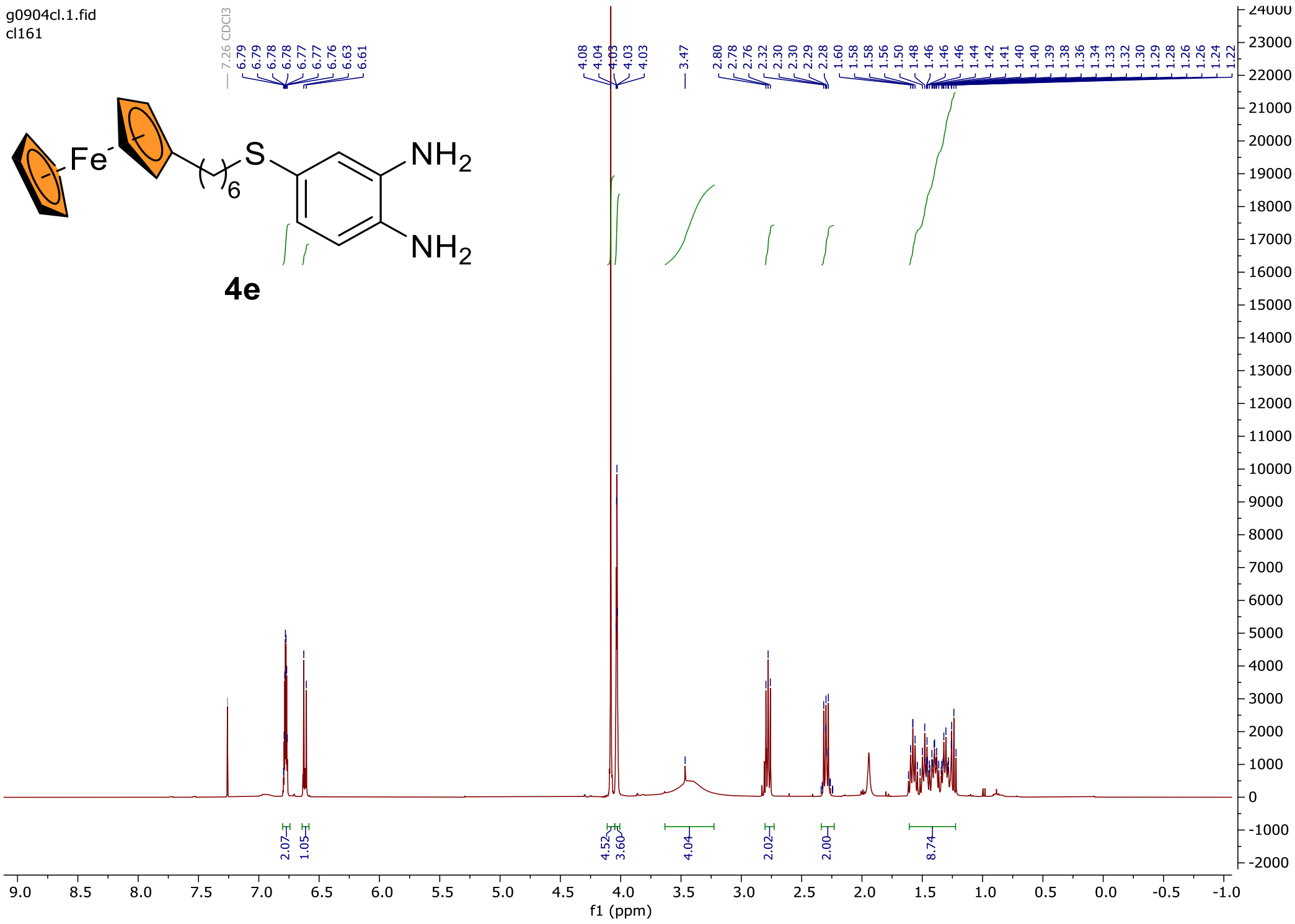

Figure S66. ^{1}H NMR spectrum of **4e** in $CDCl_3$.

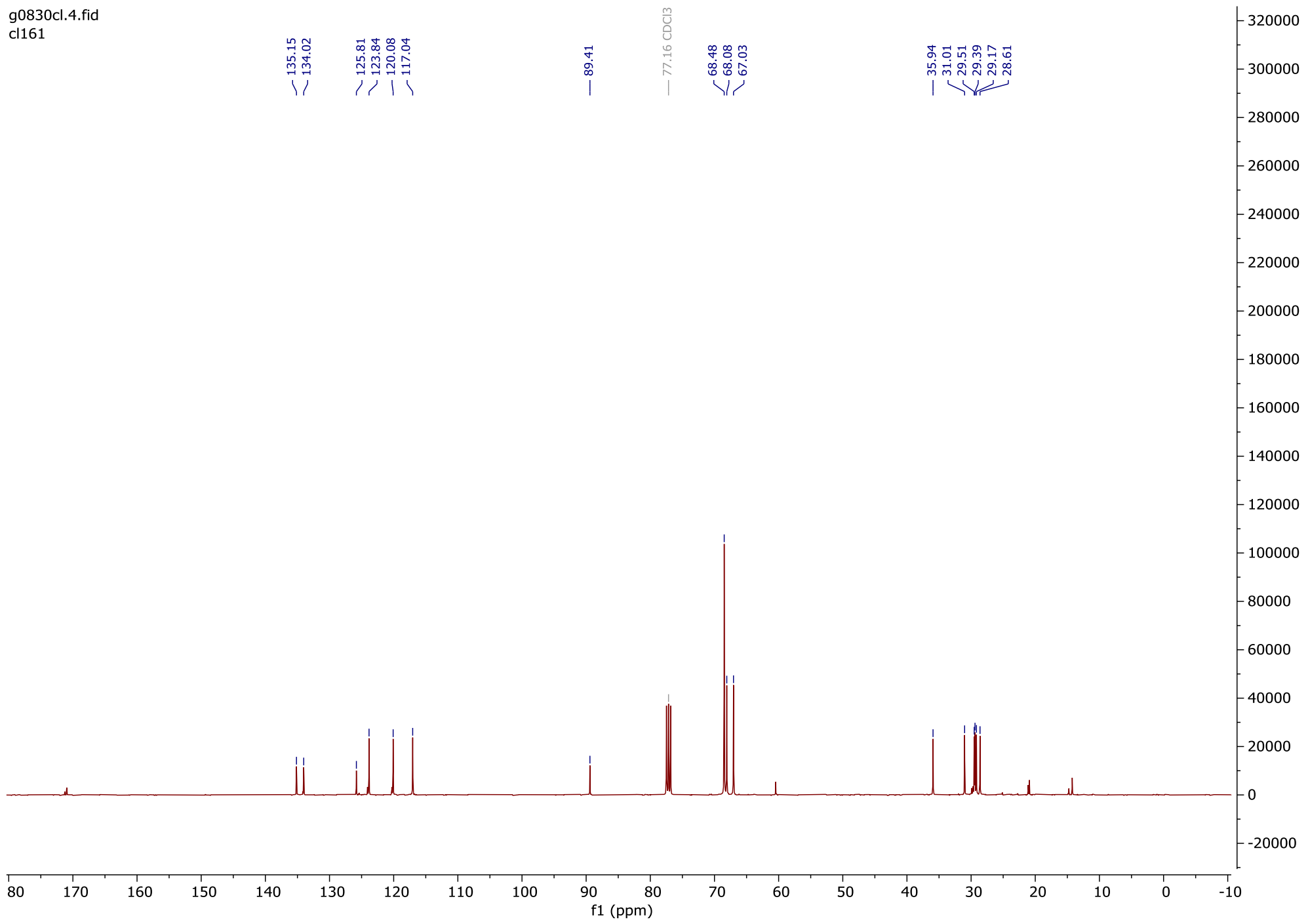

Figure S67. ^{13}C NMR spectrum of **4e** in $CDCl_3$.

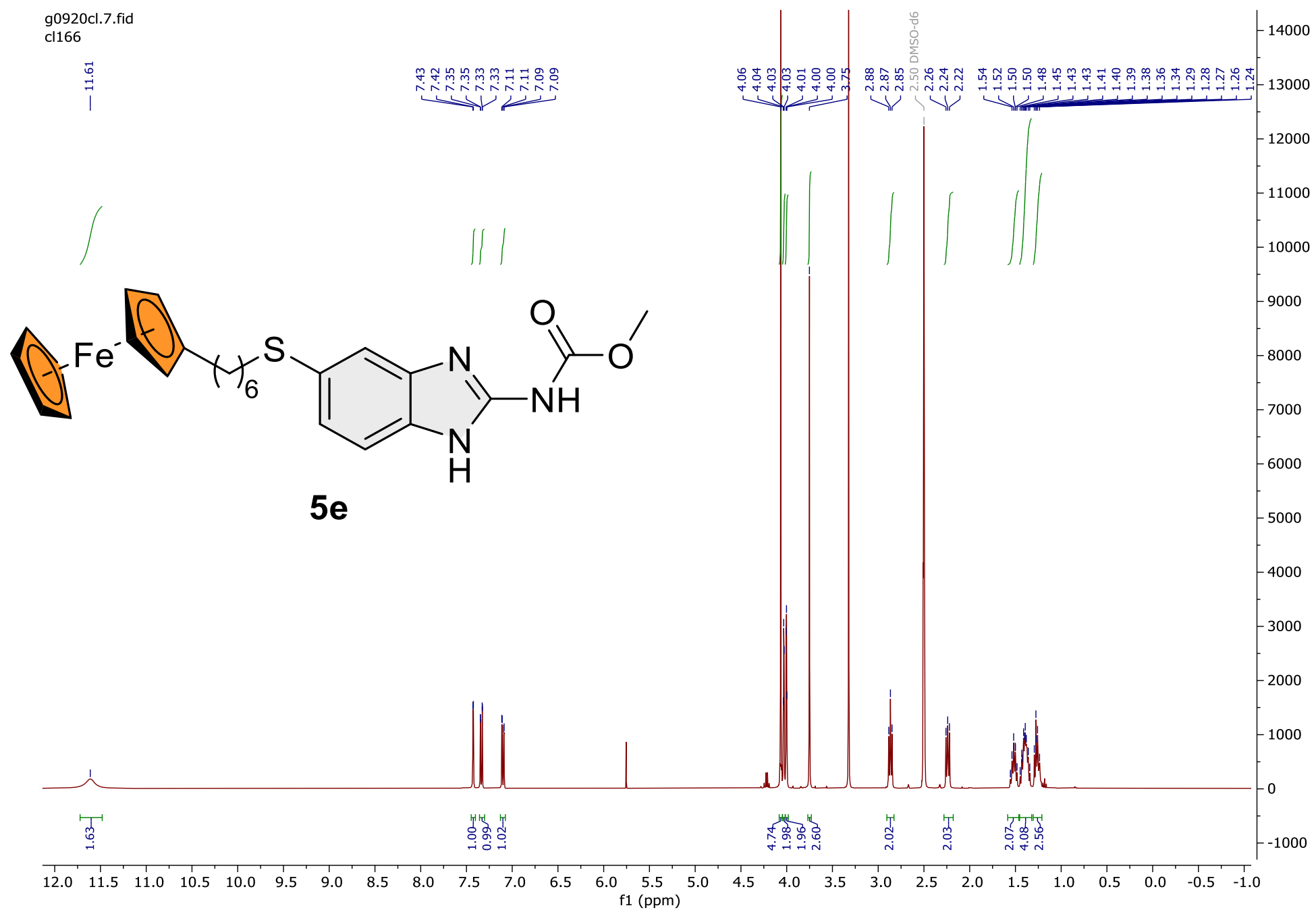

Figure S68. ^{1}H NMR spectrum of **5e** in $(CD_3)_2SO$.

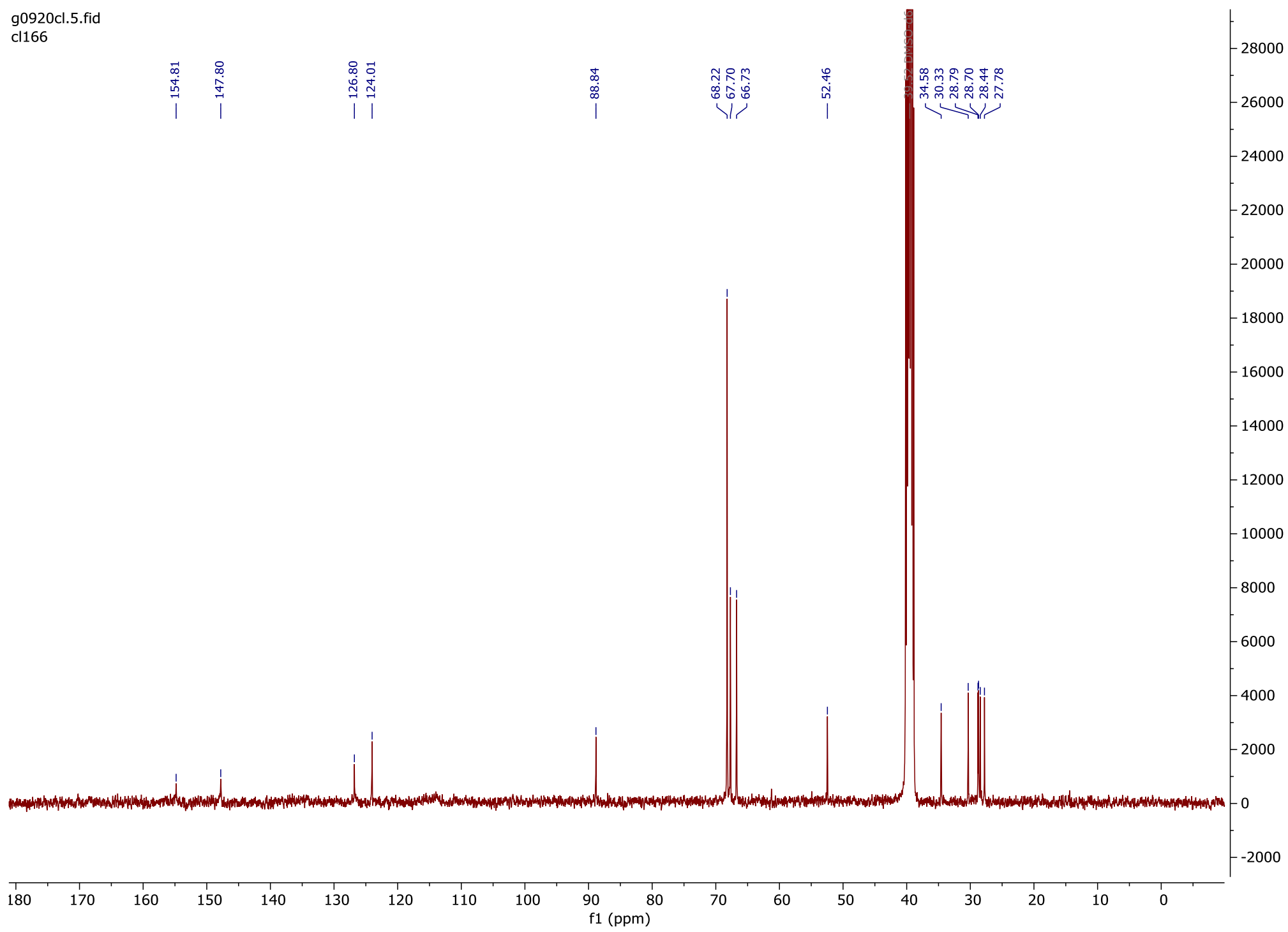

Figure S69. ^{13}C NMR spectrum of **5e** in $(CD_3)_2SO$.

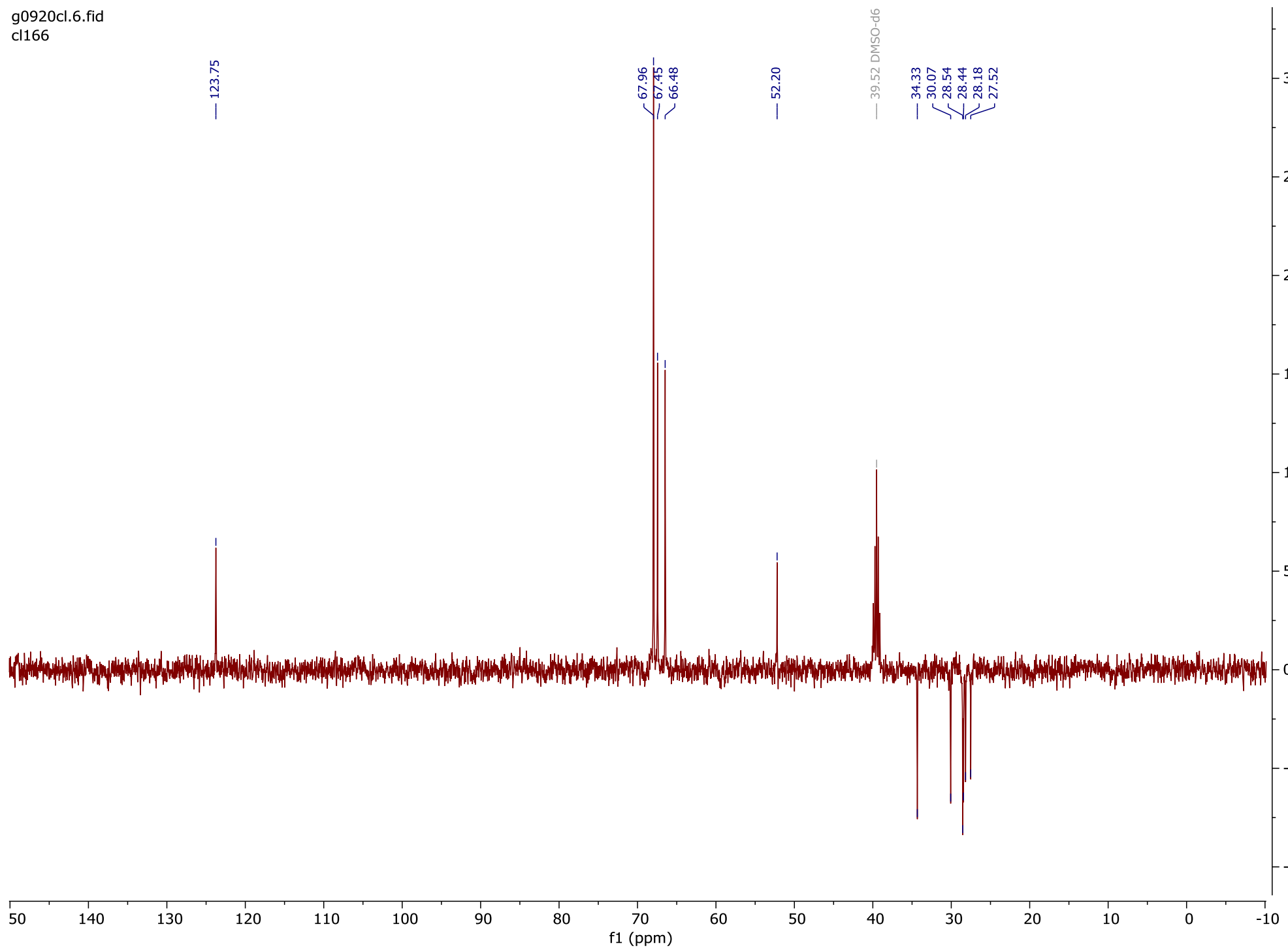

Figure S70. DEPT 135 NMR spectrum of **5e** in $(CD_3)_2SO$.

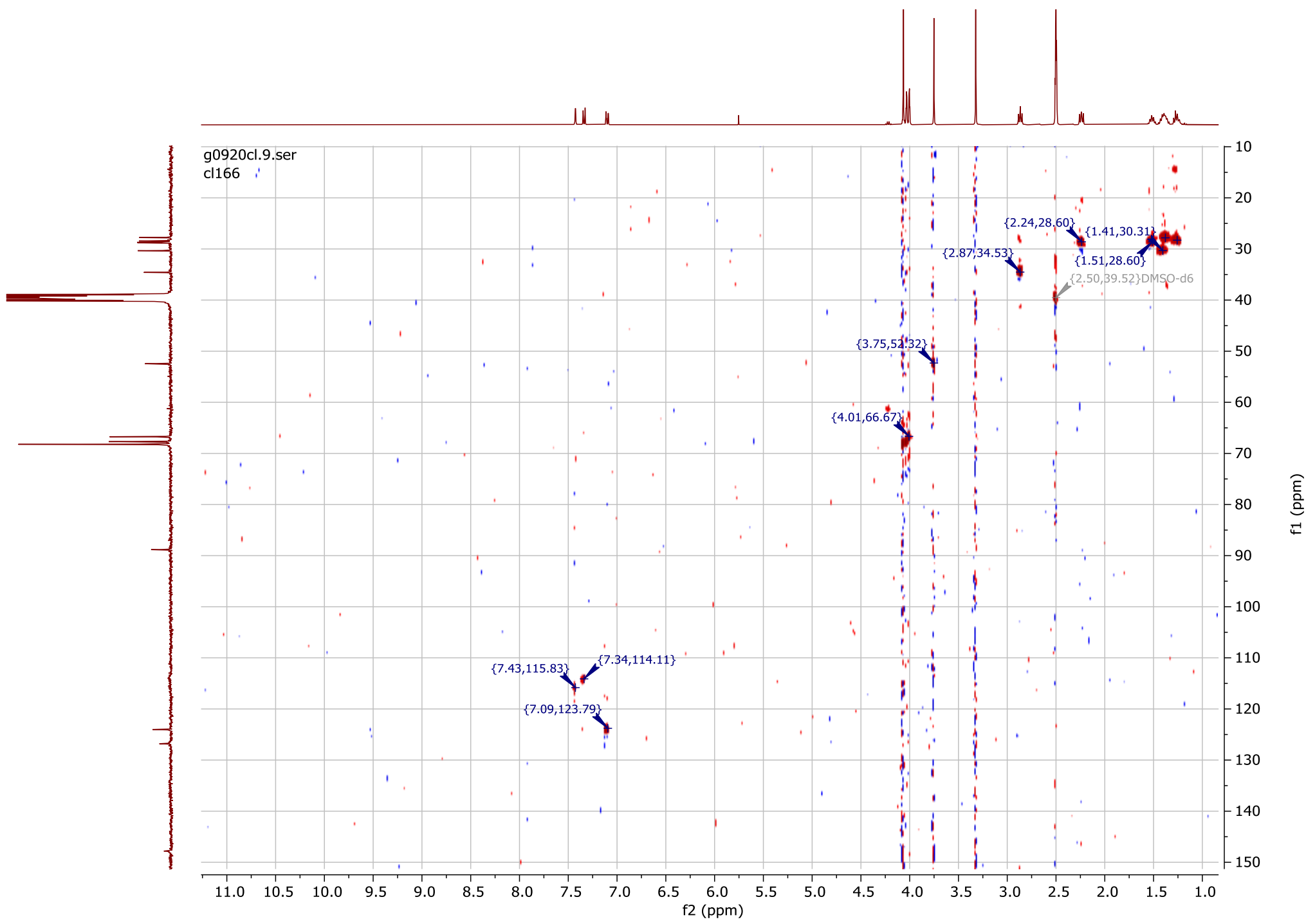

Figure S71. HSQC NMR spectrum of **5e** in $(CD_3)_2SO$.

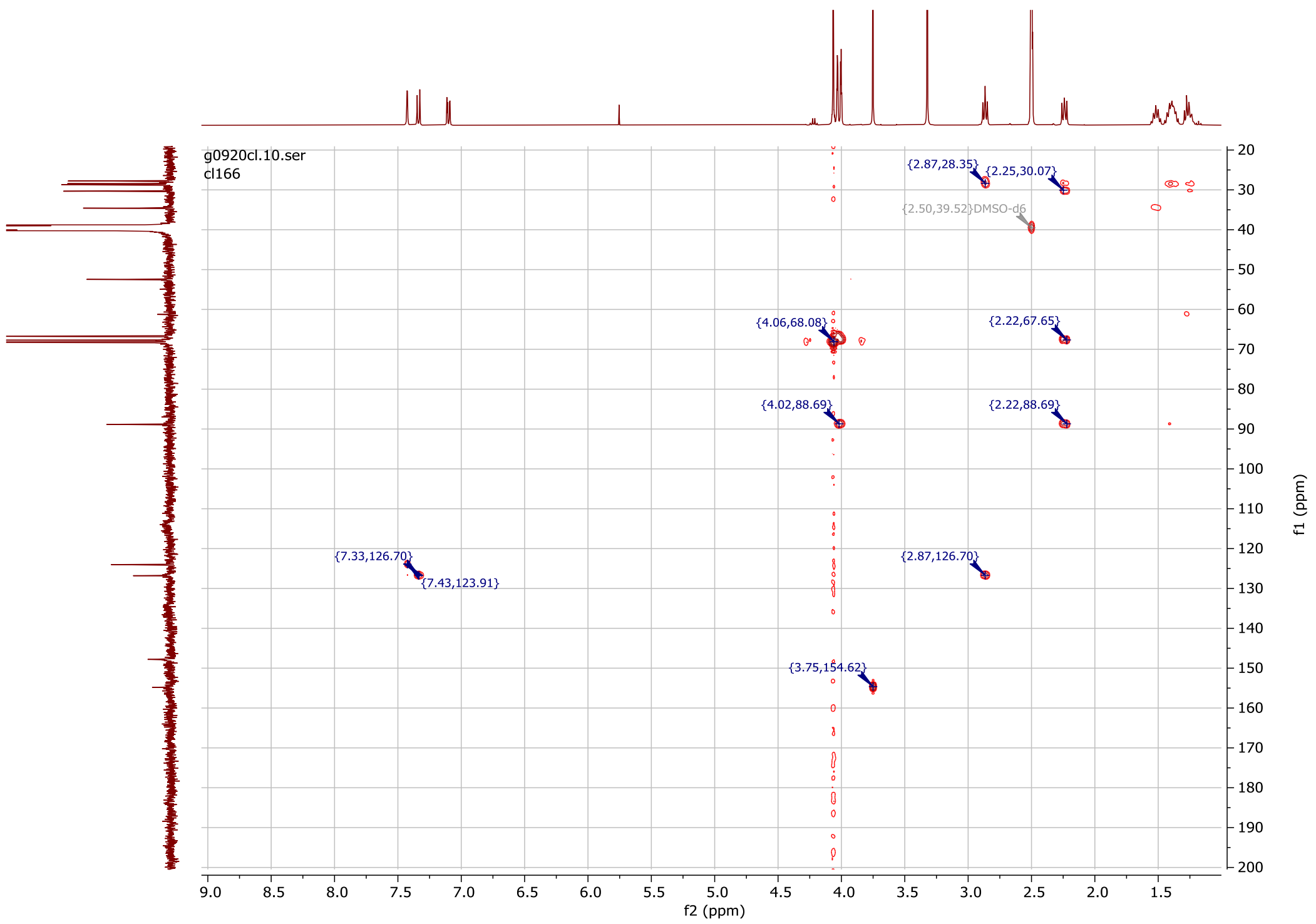

Figure S72. HMBC NMR spectrum of **5e** in $(CD_3)_2SO$.

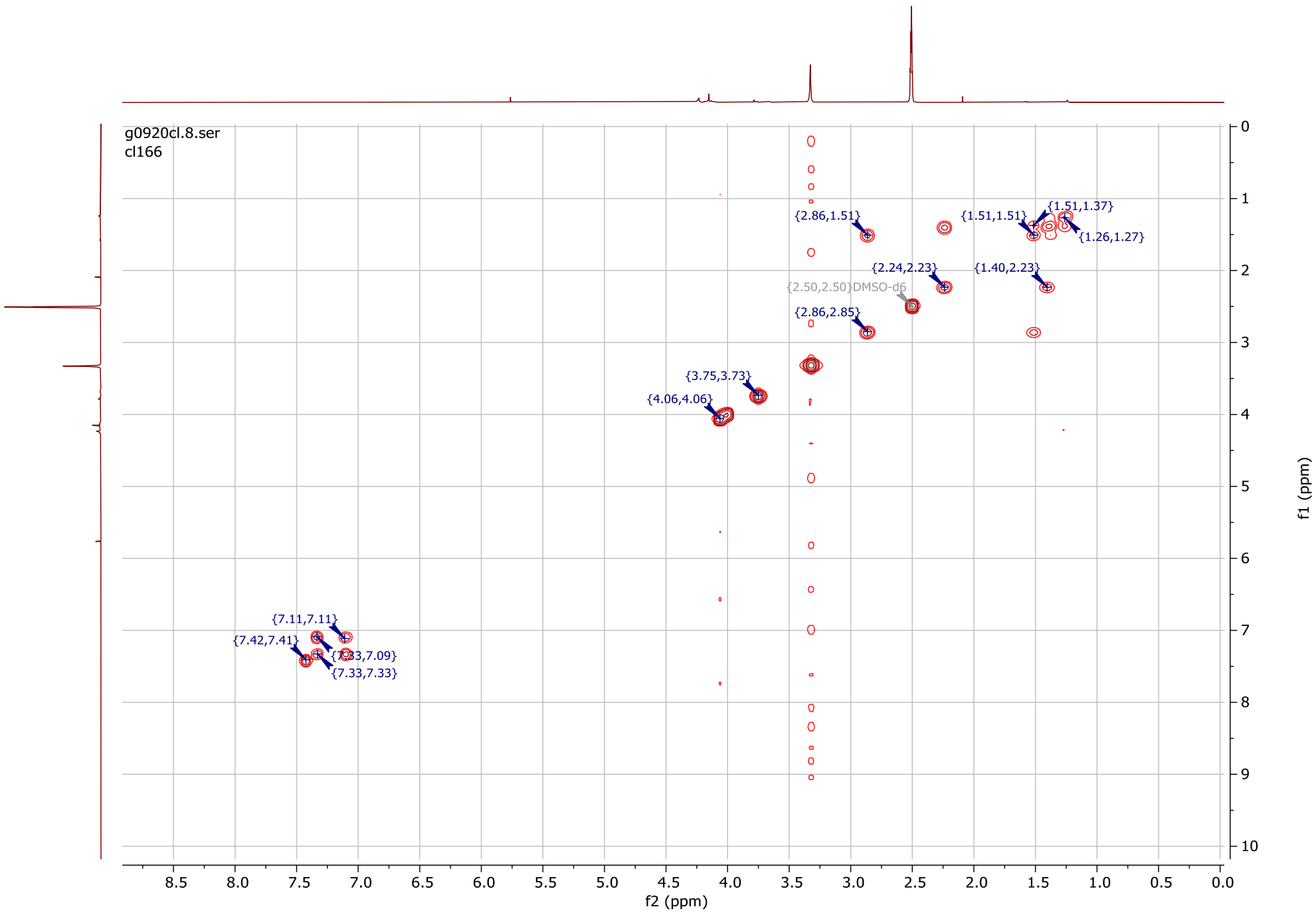

Figure S73. COSY NMR spectrum of **5e** in $(CD_3)_2SO$.

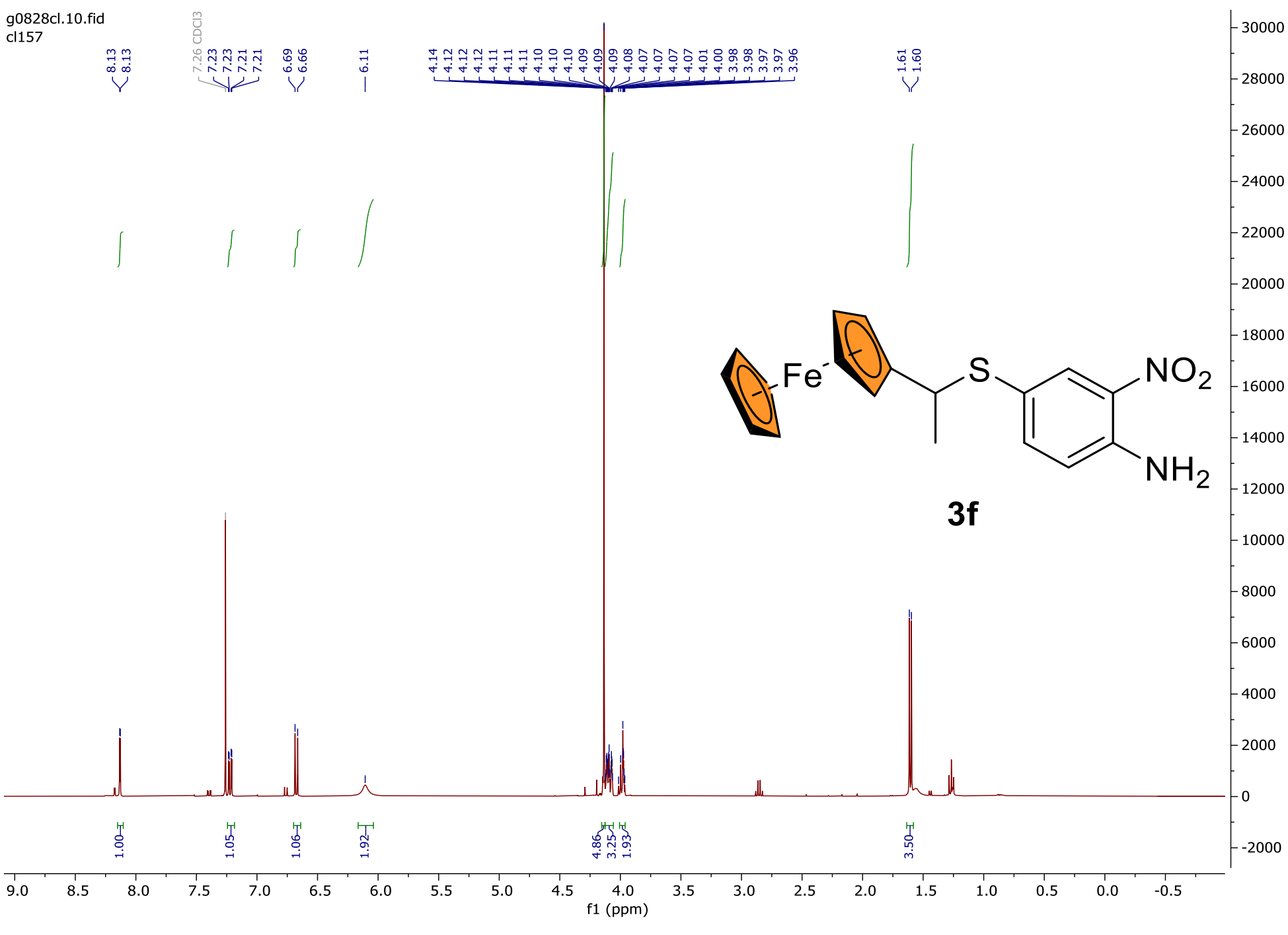

Figure S74. ^1H NMR spectrum of **3f** in $CDCl_3$.

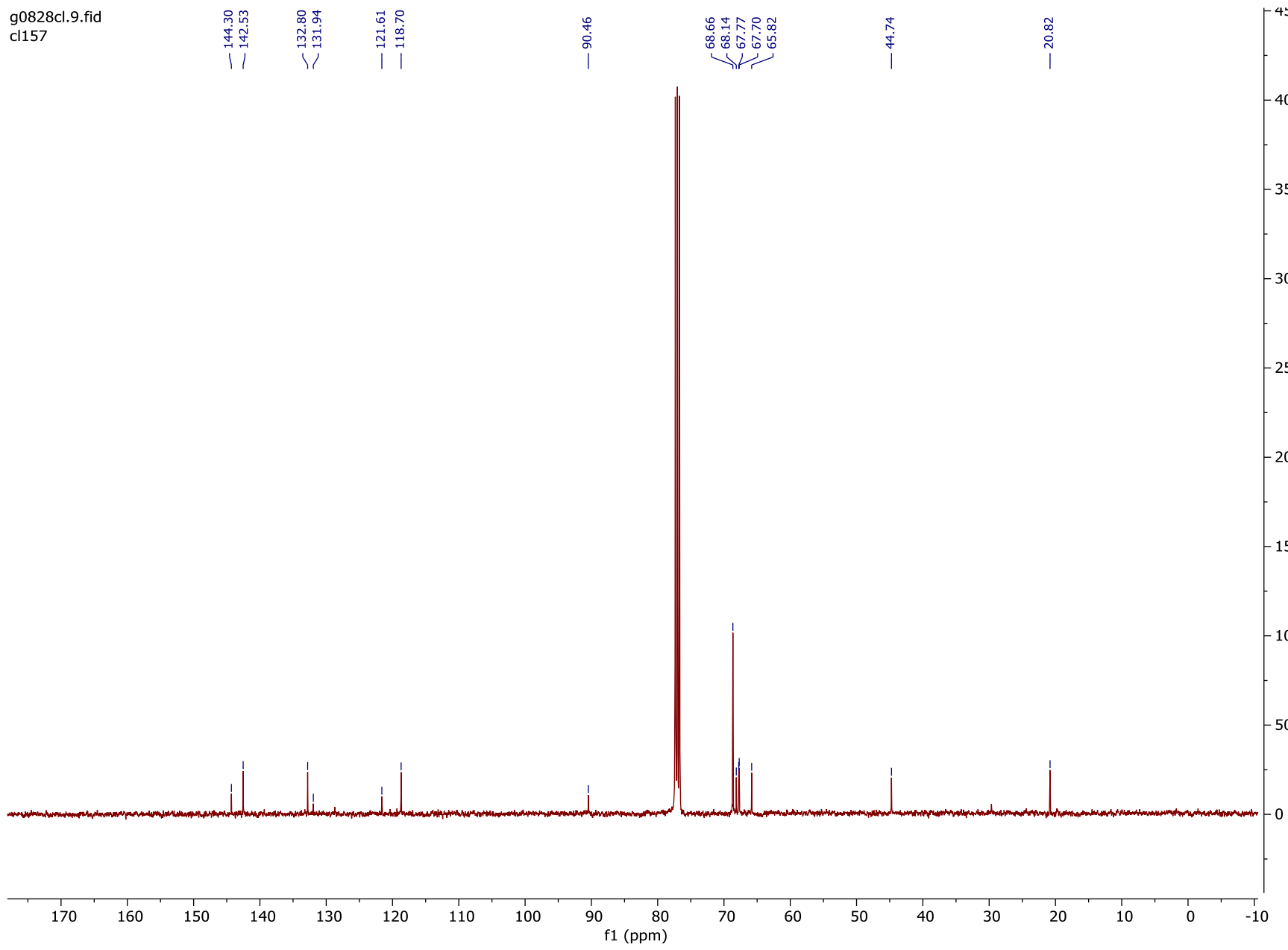

Figure S75. ^{13}C NMR spectrum of **3f** in $CDCl_3$.

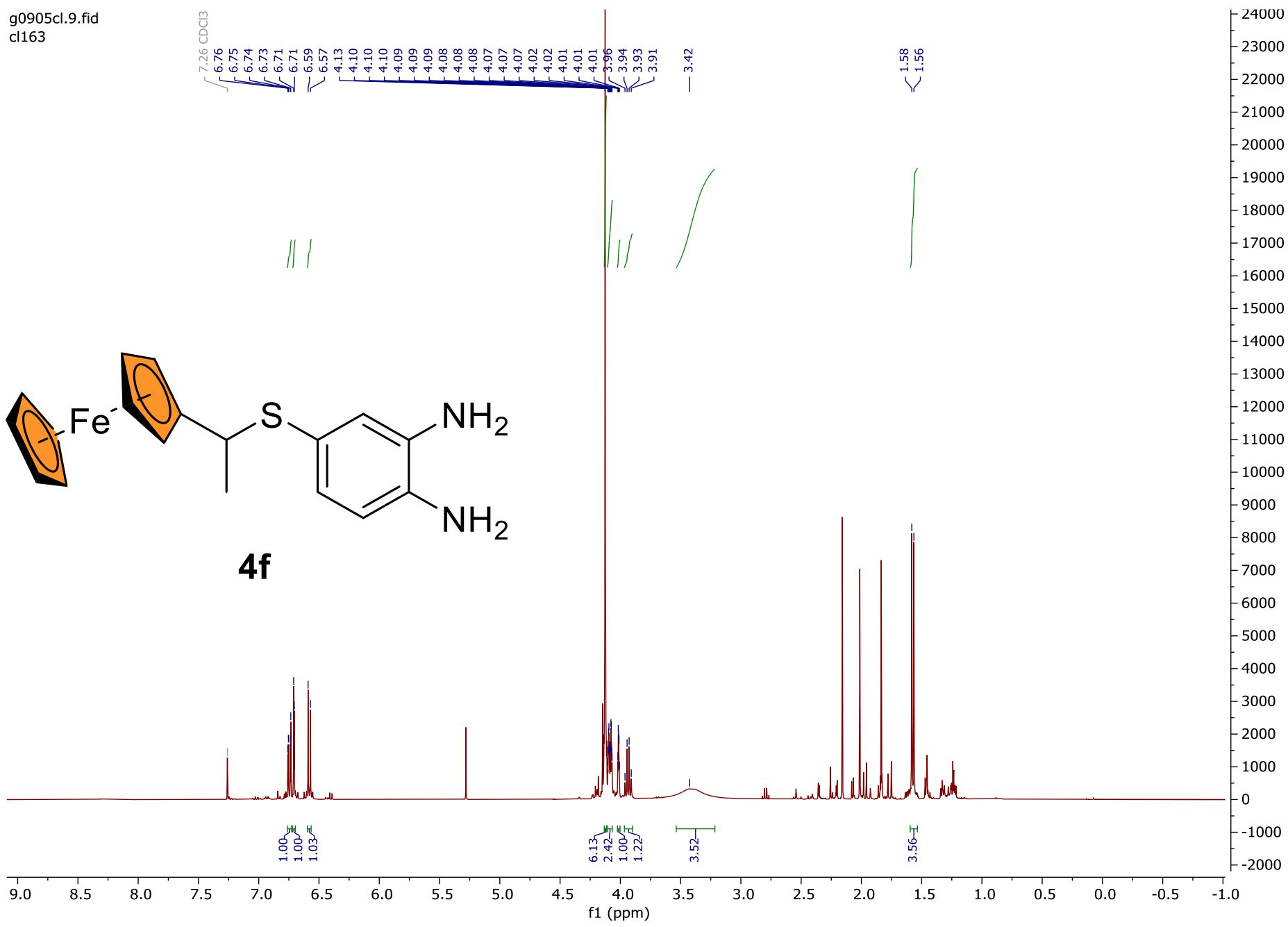

Figure S76. ^{1}H NMR spectrum of **4f** in $CDCl_3$.

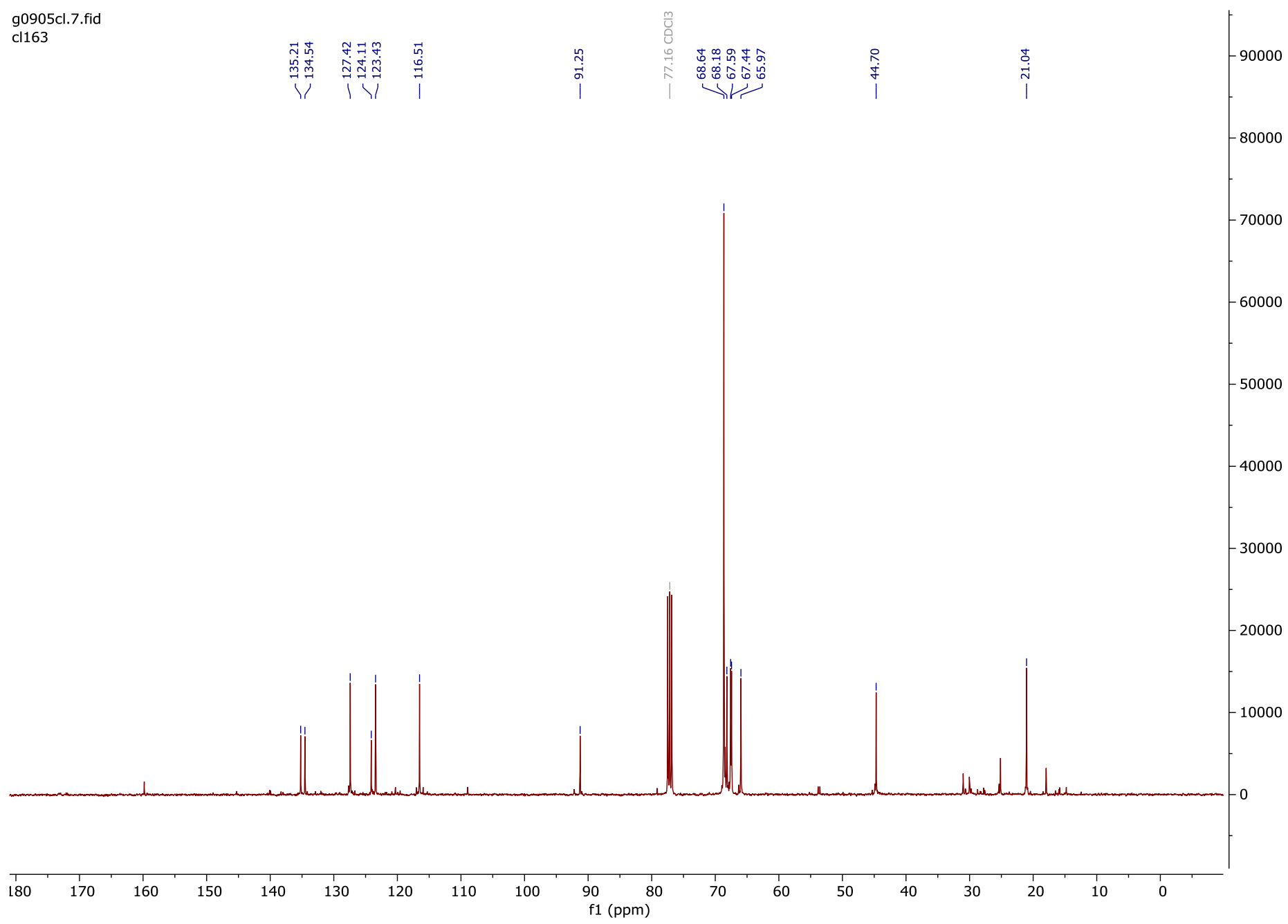

Figure S77. ^{13}C NMR spectrum of **4f** in $CDCl_3$.

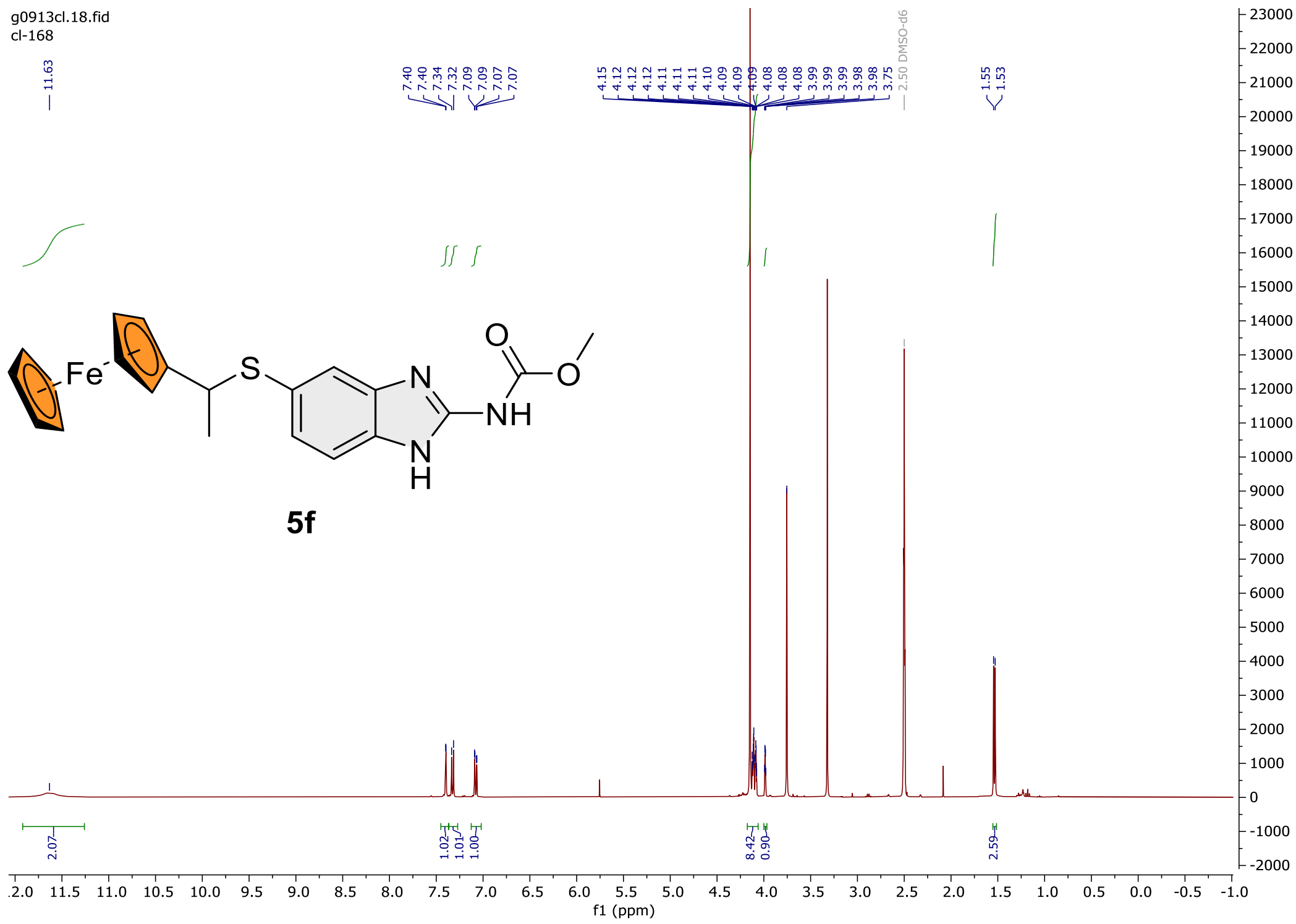

Figure S78. ^{1}H NMR spectrum of **5f** in $(CD_3)_2SO$.

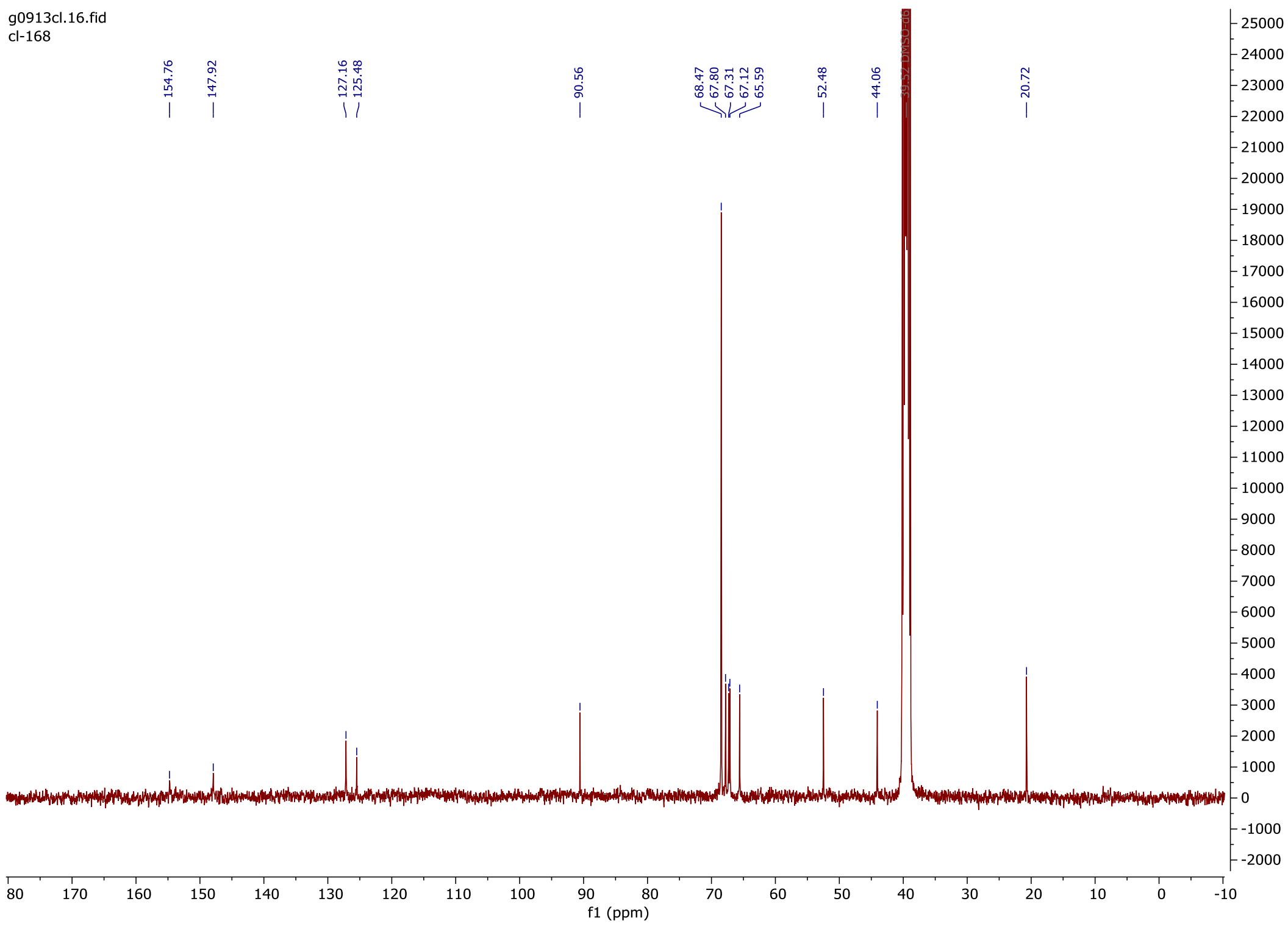

Figure S79. ^{13}C NMR spectrum of **5f** in $(CD_3)_2SO$.

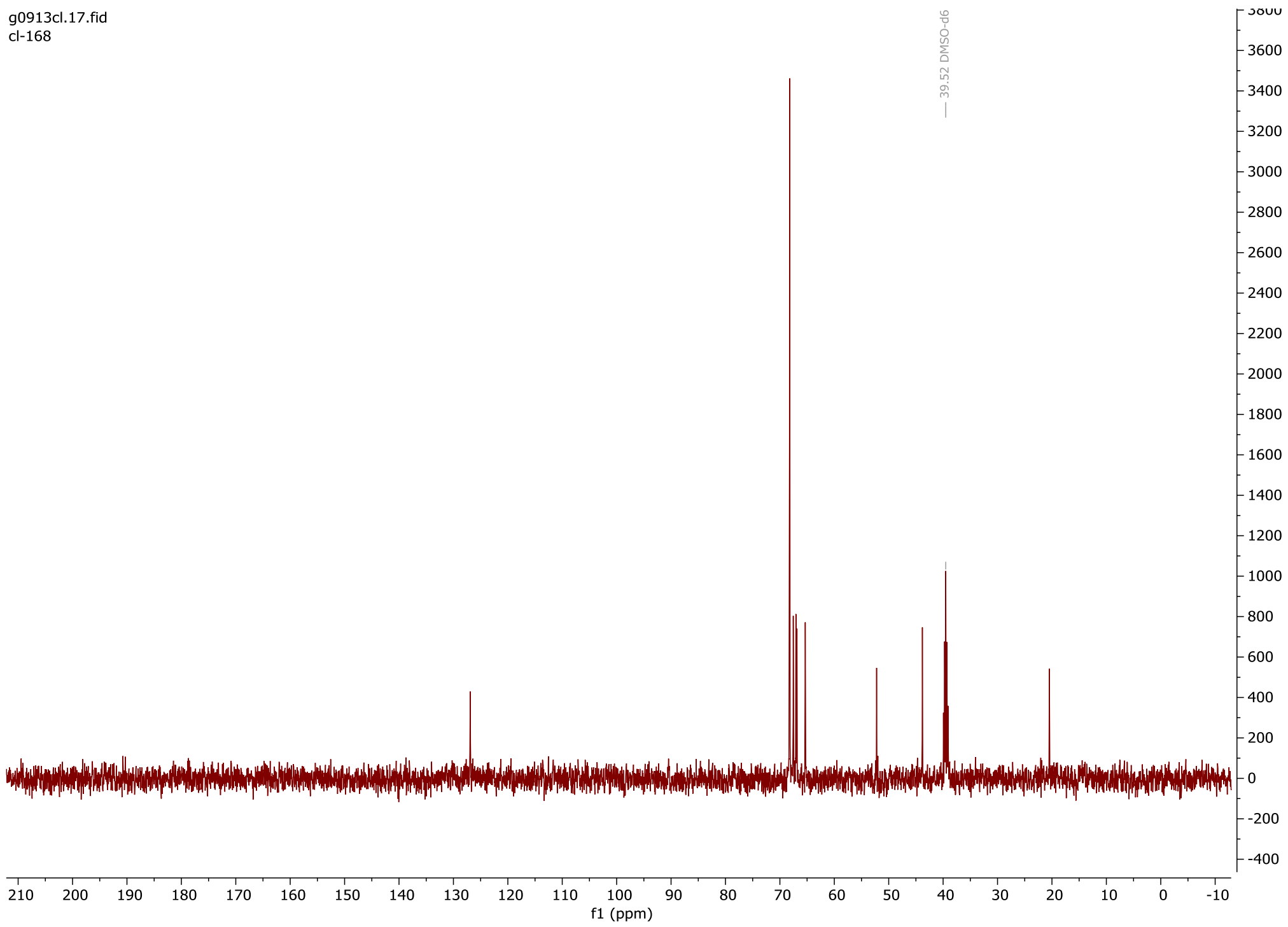

Figure S80. DEPT 135 NMR spectrum of **5f** in $(CD_3)_2SO$.

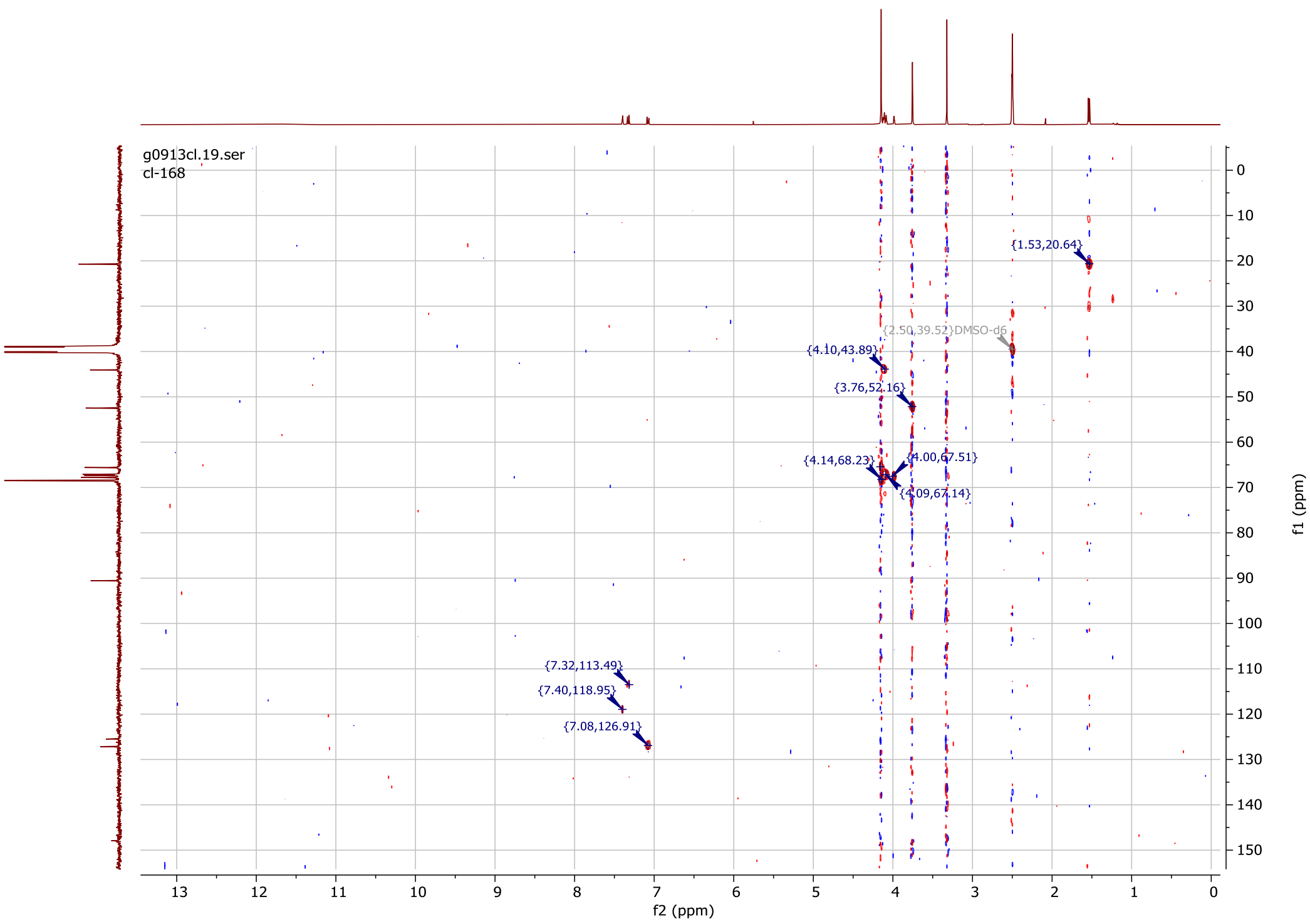

Figure S81. HSQC NMR spectrum of **5f** in $(CD_3)_2SO$.

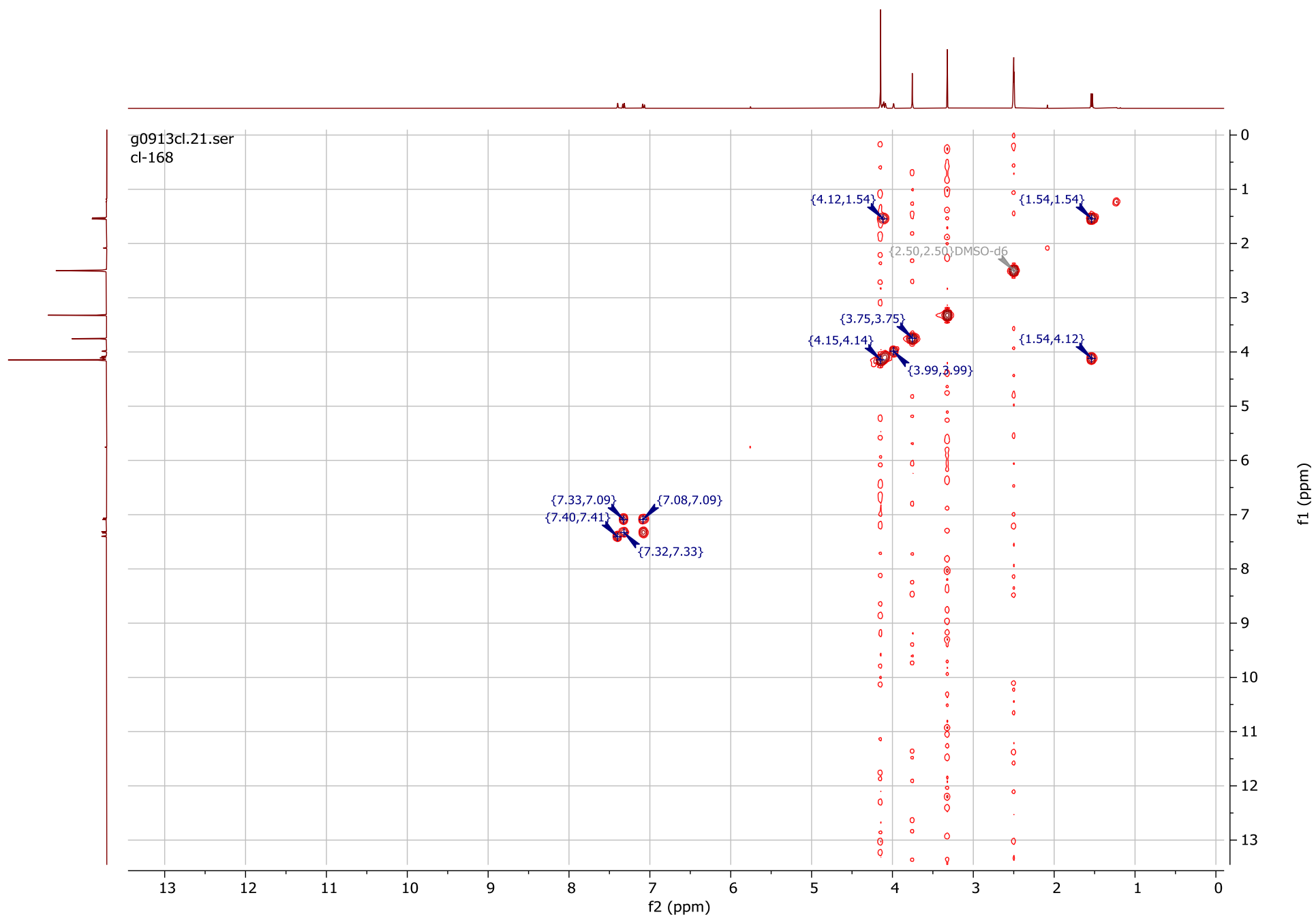

Figure S82. COSY NMR spectrum of **5f** in $(CD_3)_2SO$.

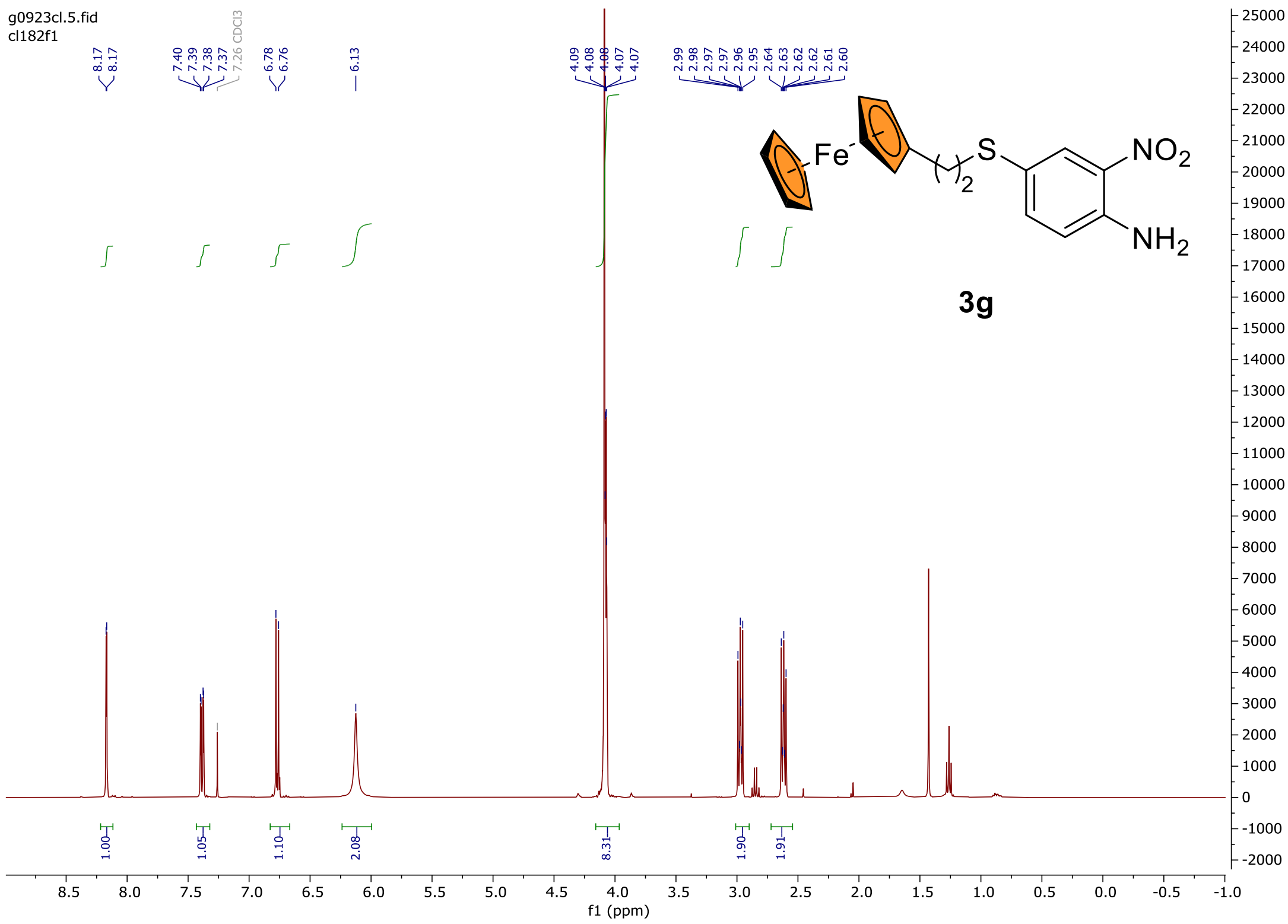

Figure S83. ^{1}H NMR spectrum of **3g** in $CDCl_3$.

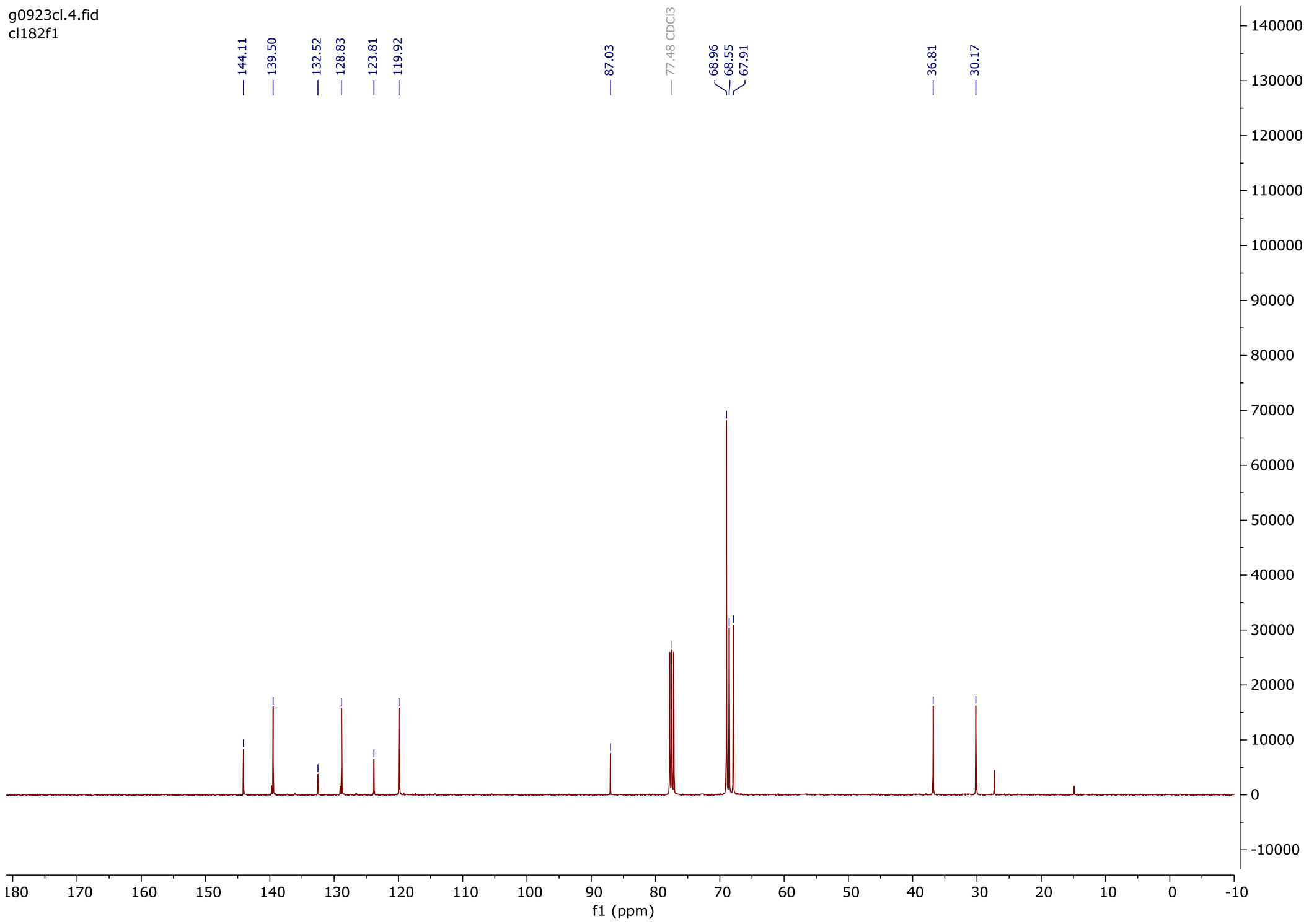

Figure S84. ^{13}C NMR spectrum of **3g** in $CDCl_3$.

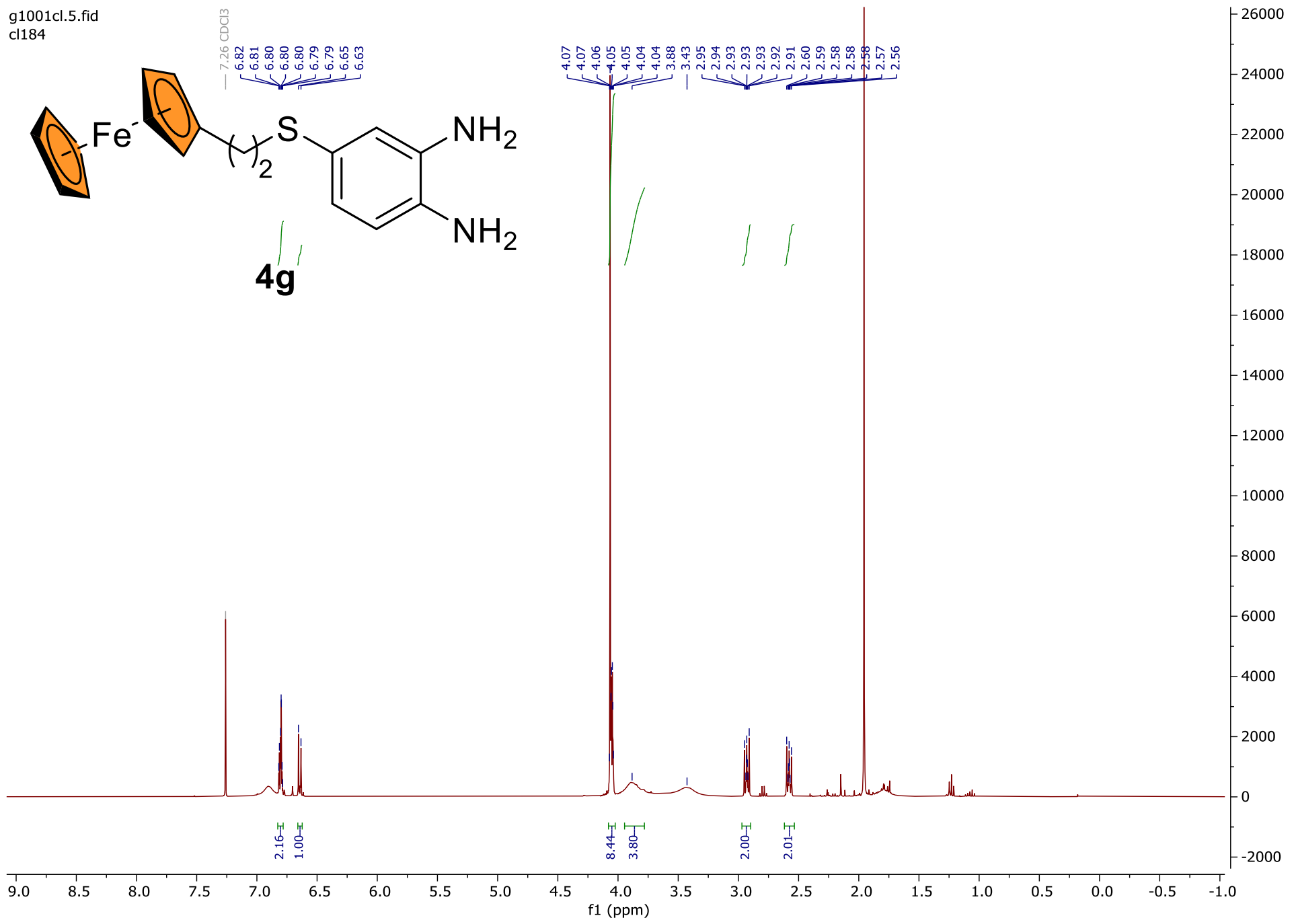

Figure S85. ^{1}H NMR spectrum of **4g** in $CDCl_3$.

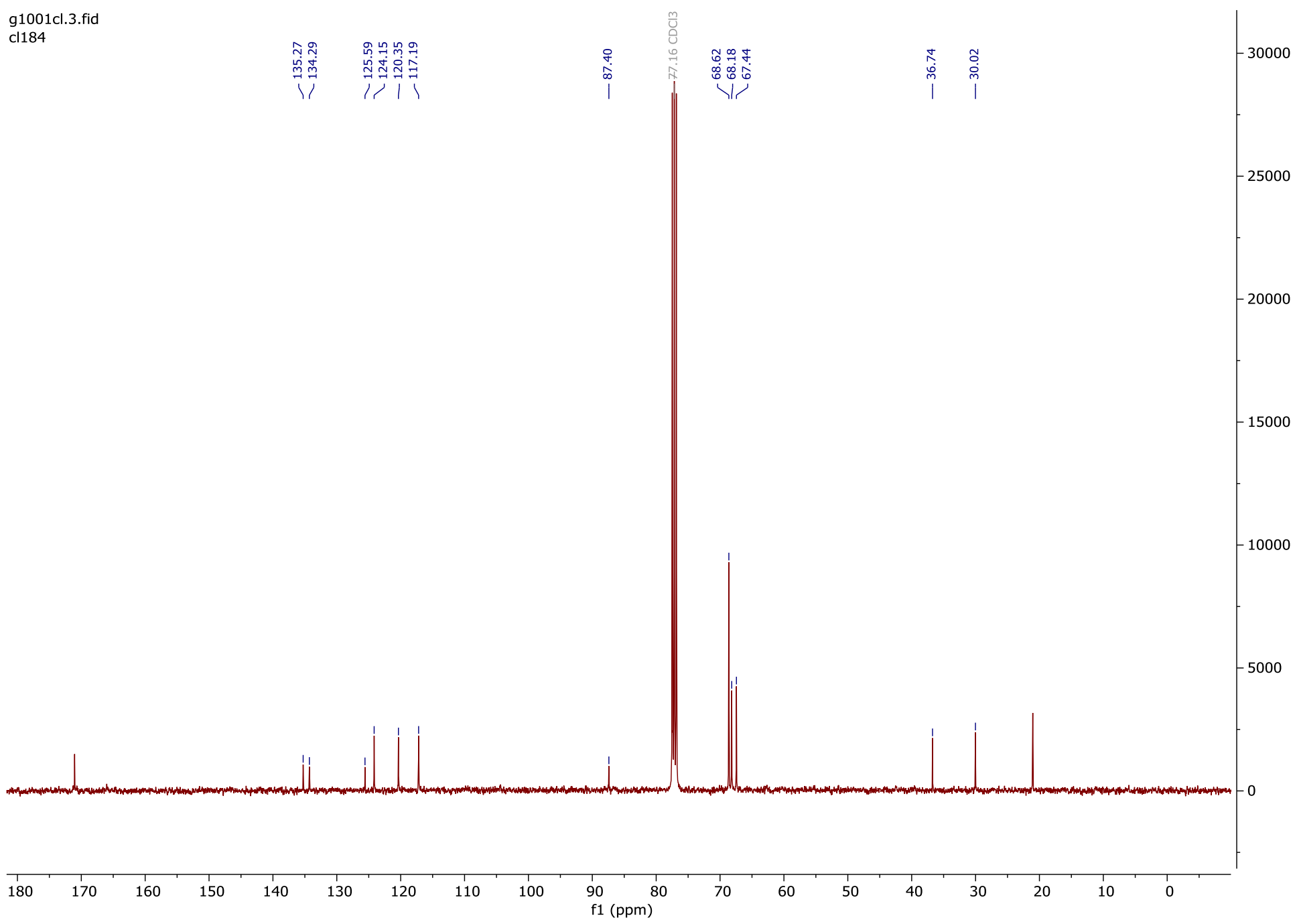

Figure S86. ^{13}C NMR spectrum of **4g** in $CDCl_3$.

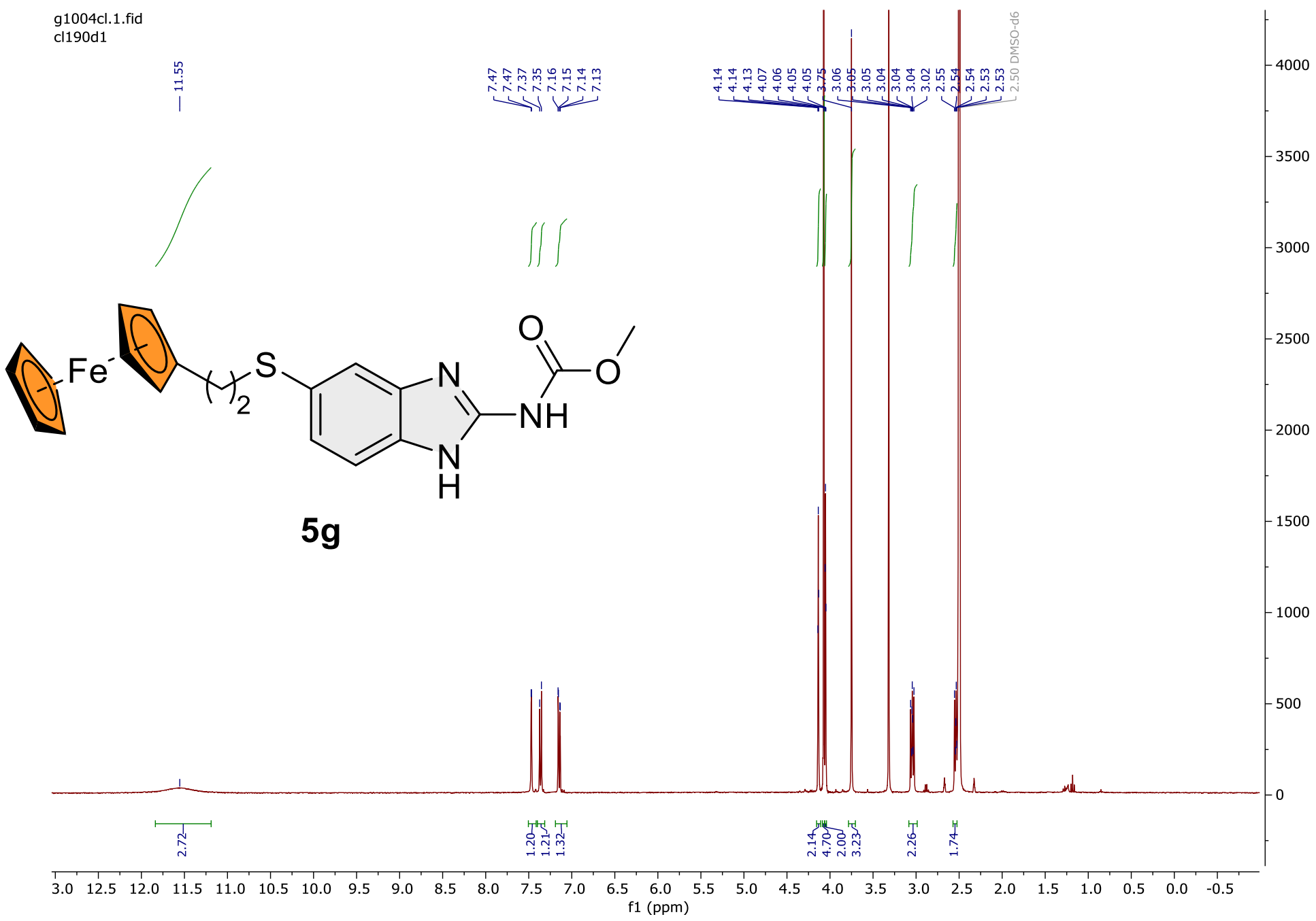

Figure S87. ^{1}H NMR spectrum of **5g** in $(CD_3)_2SO$.

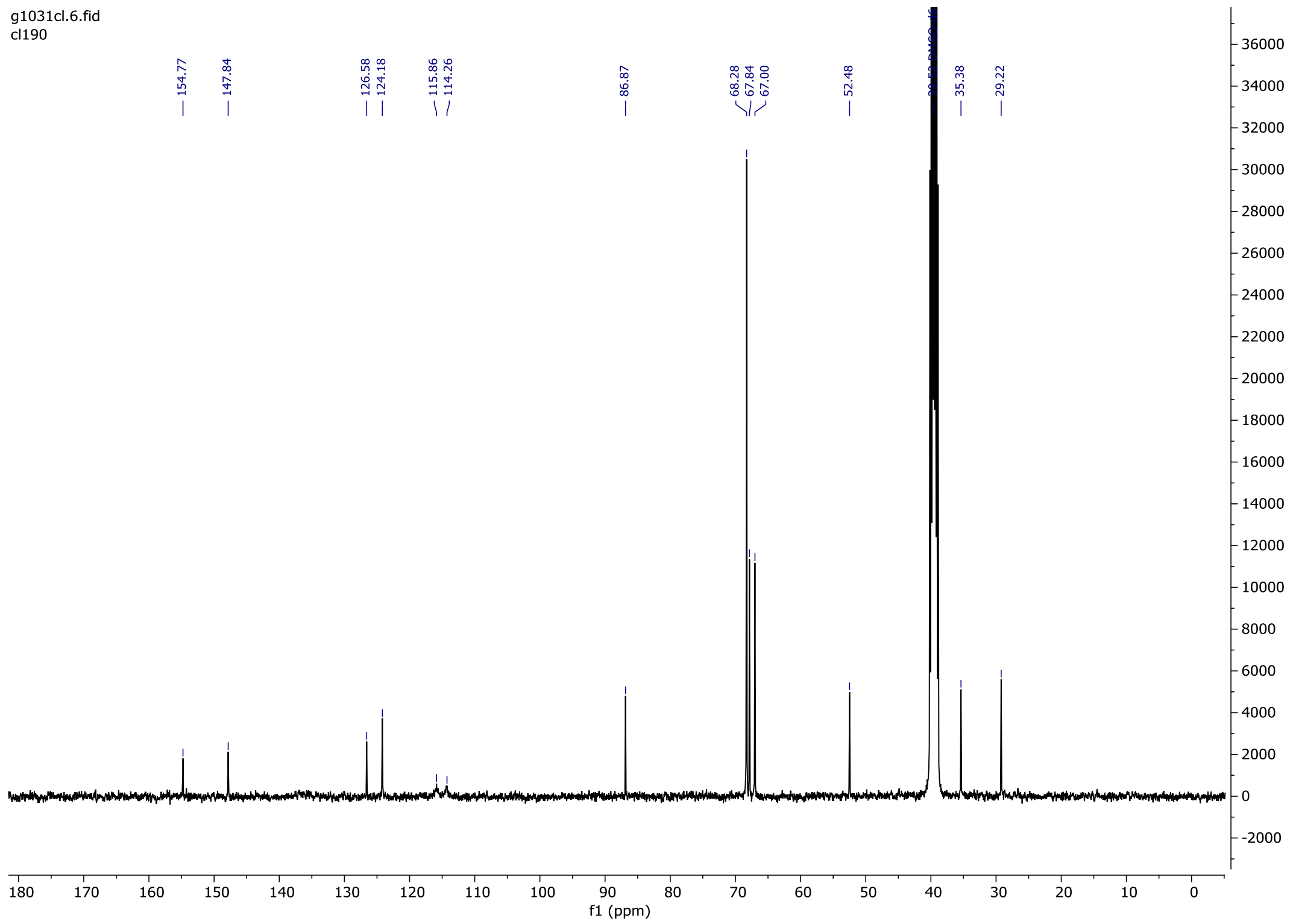

Figure S88. ^{13}C NMR spectrum of **5g** in $(CD_3)_2SO$.

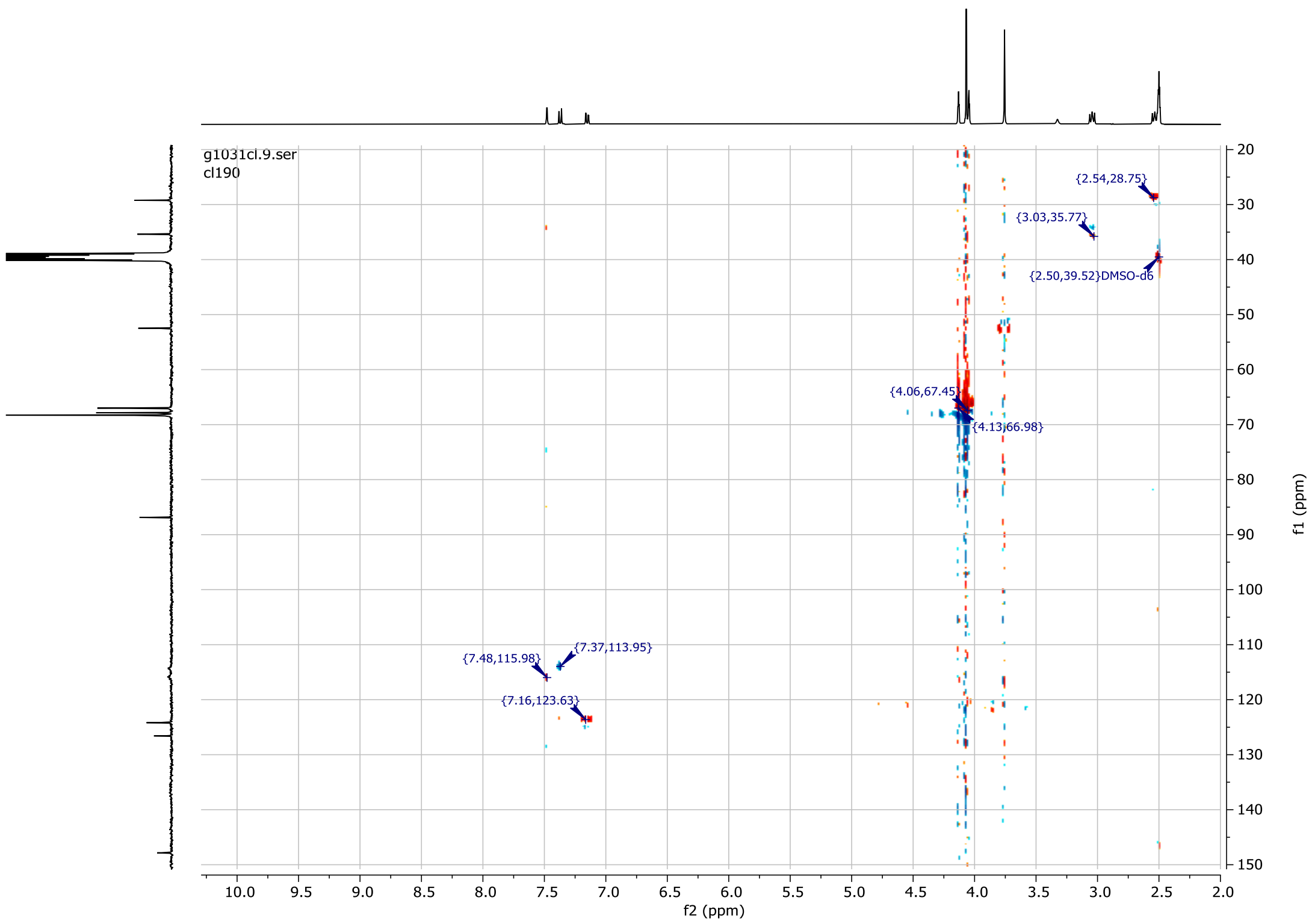

Figure S89. HSQC NMR spectrum of **5g** in $(CD_3)_2SO$.

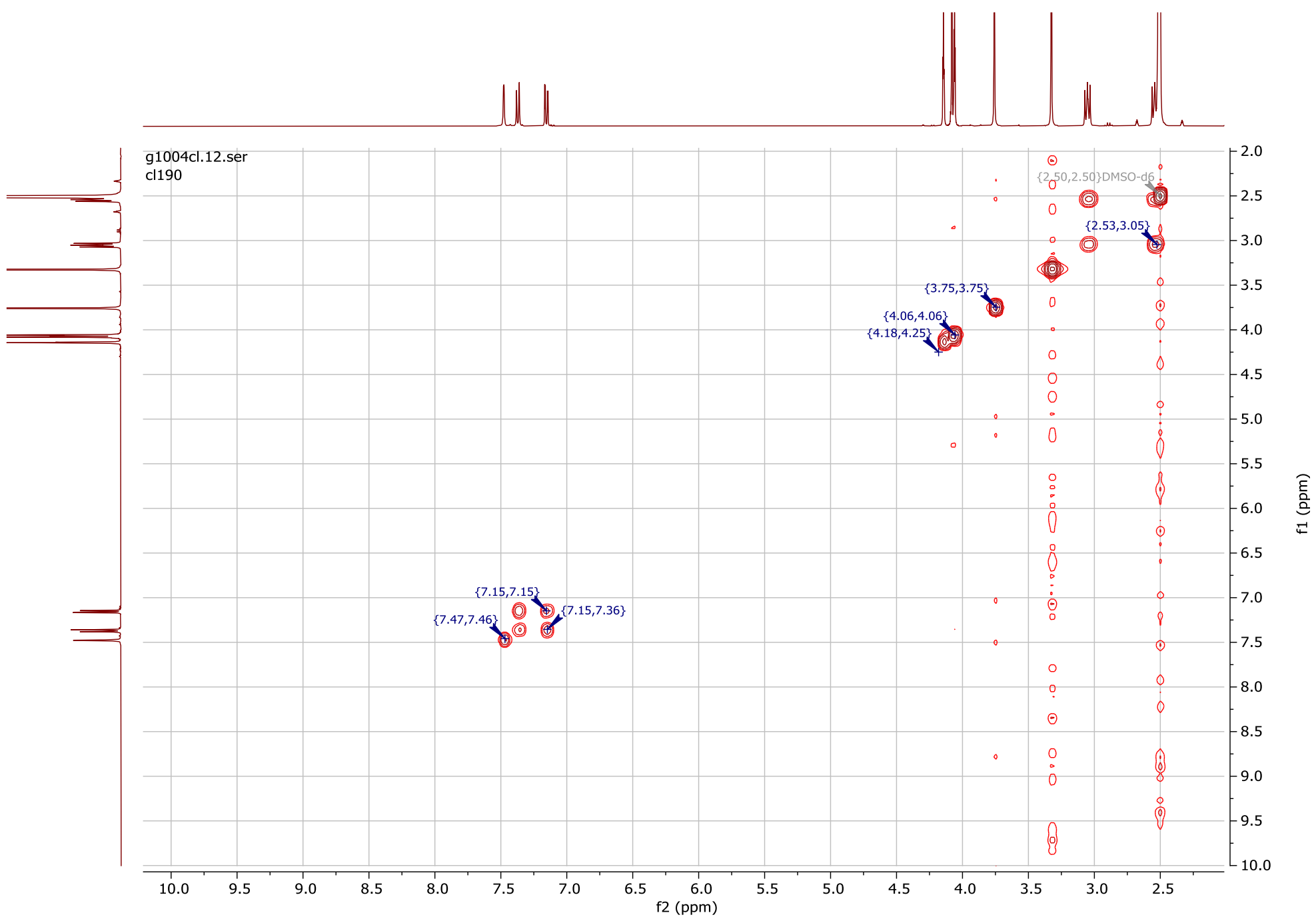

Figure S90. COSY NMR spectrum of **5g** in $(CD_3)_2SO$.

1.6. ^{1}H NMR spectra for stability in DMSO

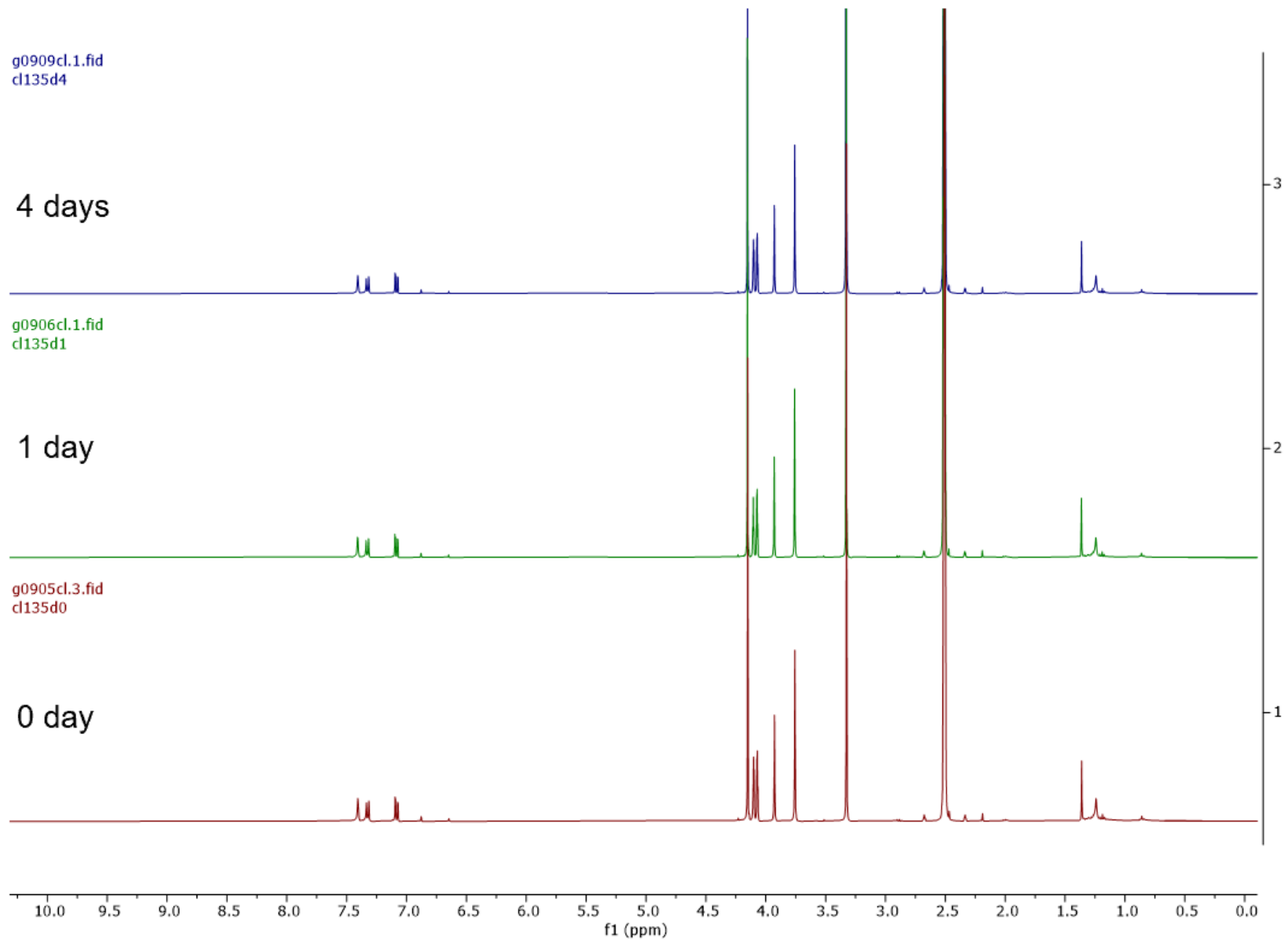

Figure S91. ^{1}H NMR spectra of **5a** for stability in $(CD_3)_2SO$ up to 4 days.

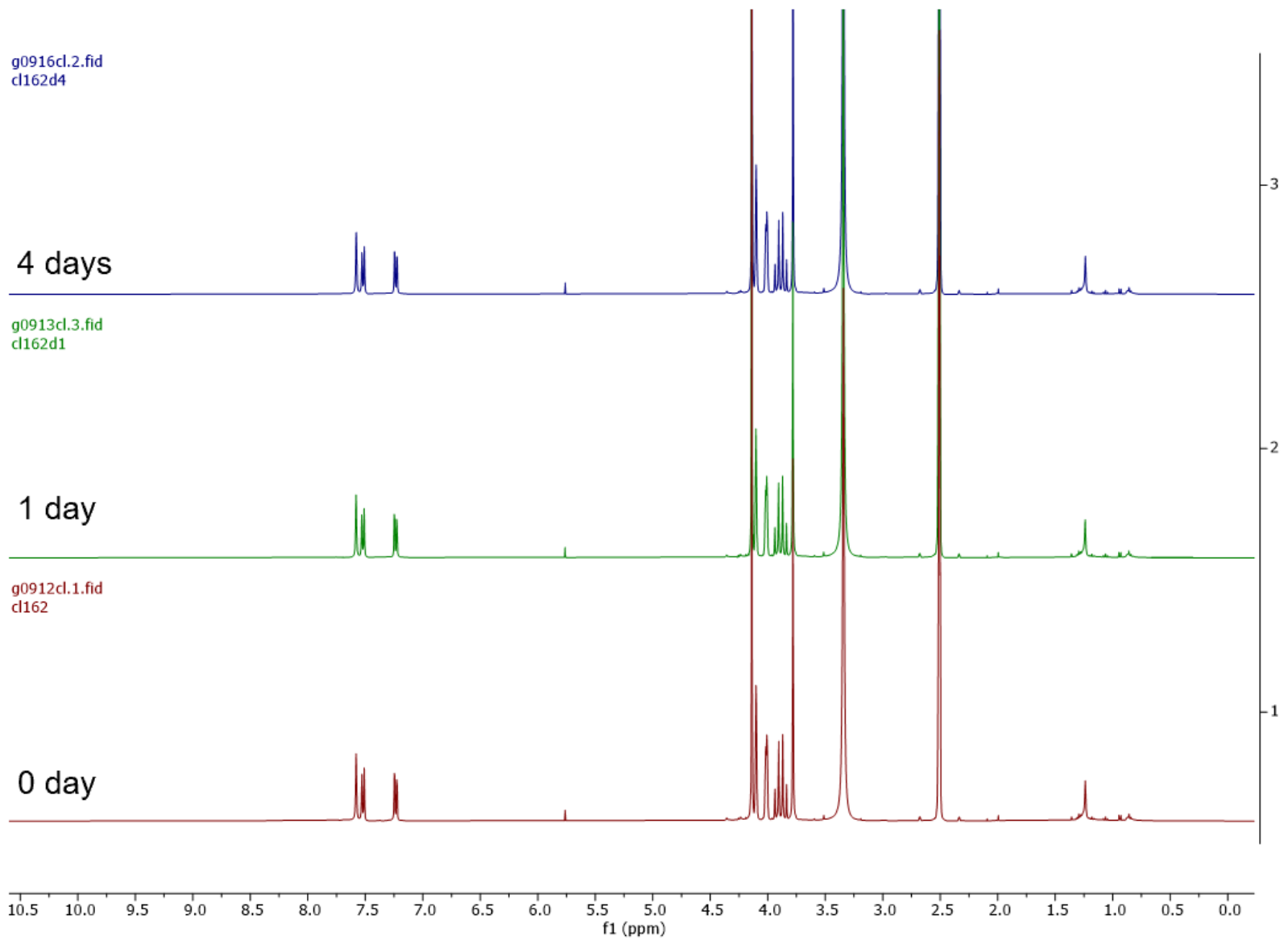

Figure S92. ^{1}H NMR spectra of **6a** for stability in $(CD_3)_2SO$ up to 4 days.

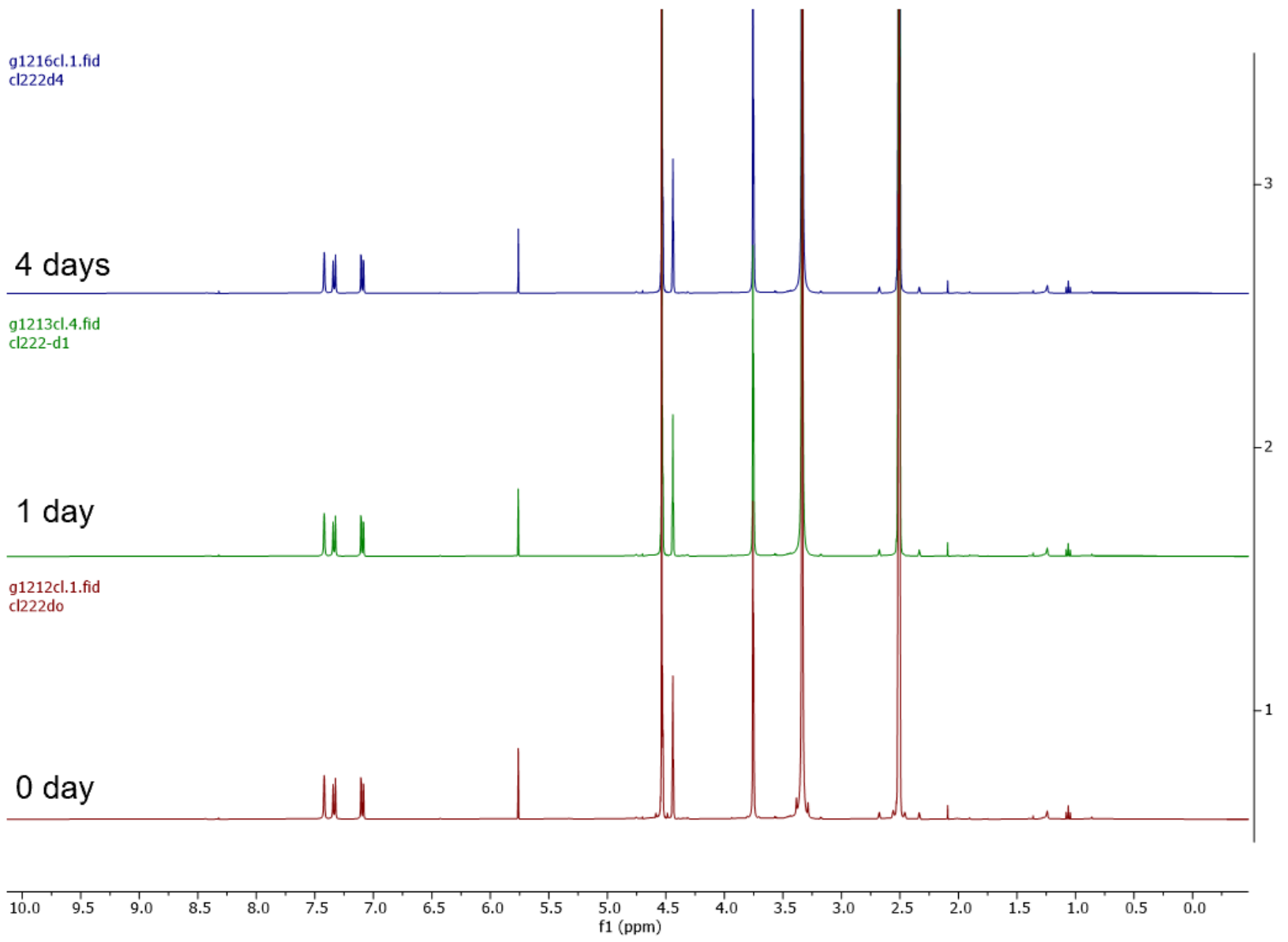

Figure S93. ^{1}H NMR spectra of **5b** for stability in $(CD_3)_2SO$ up to 4 days.

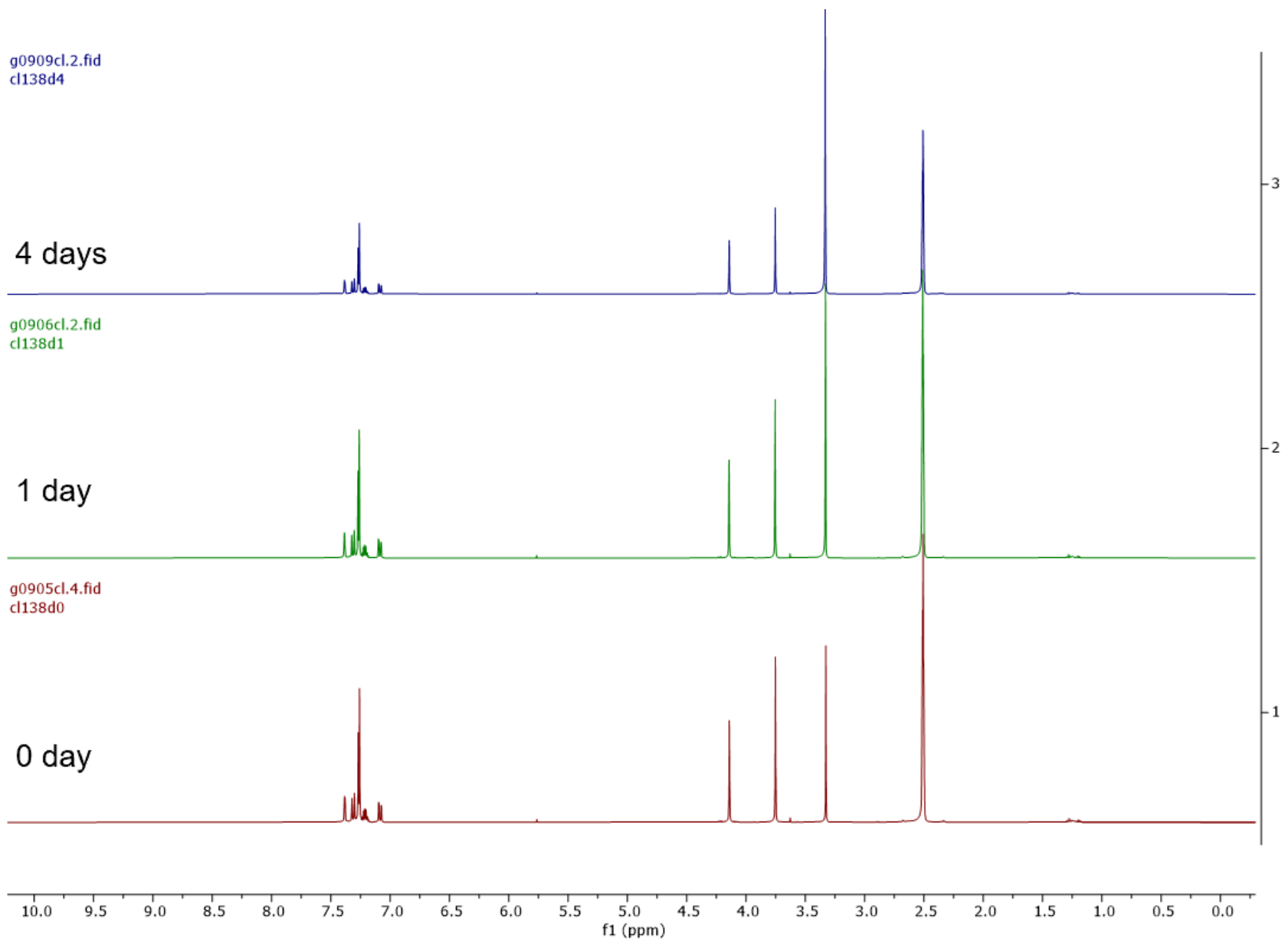

Figure S94. ^{1}H NMR spectra of **5c** for stability in $(CD_3)_2SO$ up to 4 days.

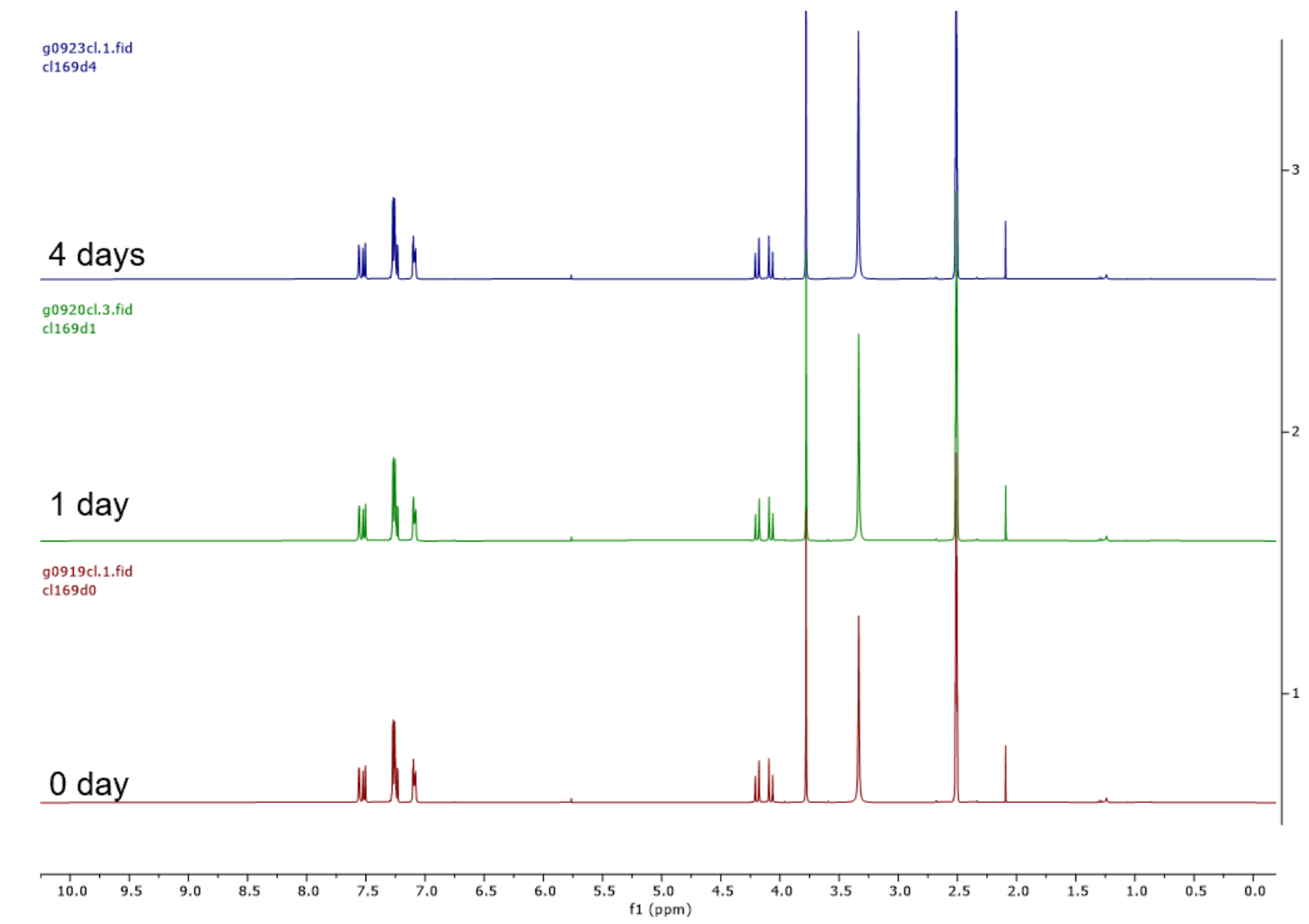

Figure S95. ^{1}H NMR spectra of **6c** for stability in $(CD_3)_2SO$ up to 4 days.

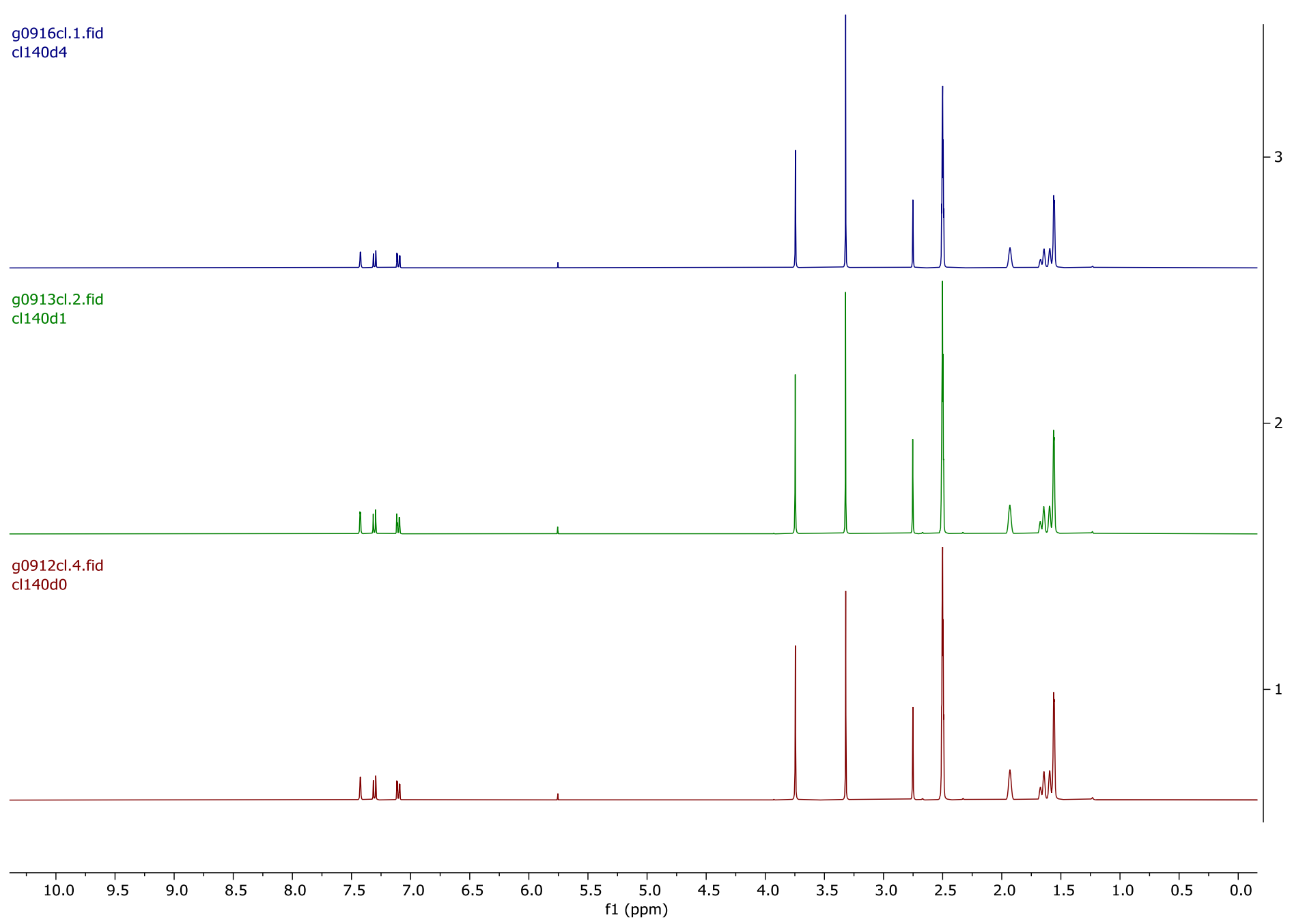

Figure S96. ^{1}H NMR spectra of **5d** for stability in $(CD_3)_2SO$ up to 4 days.

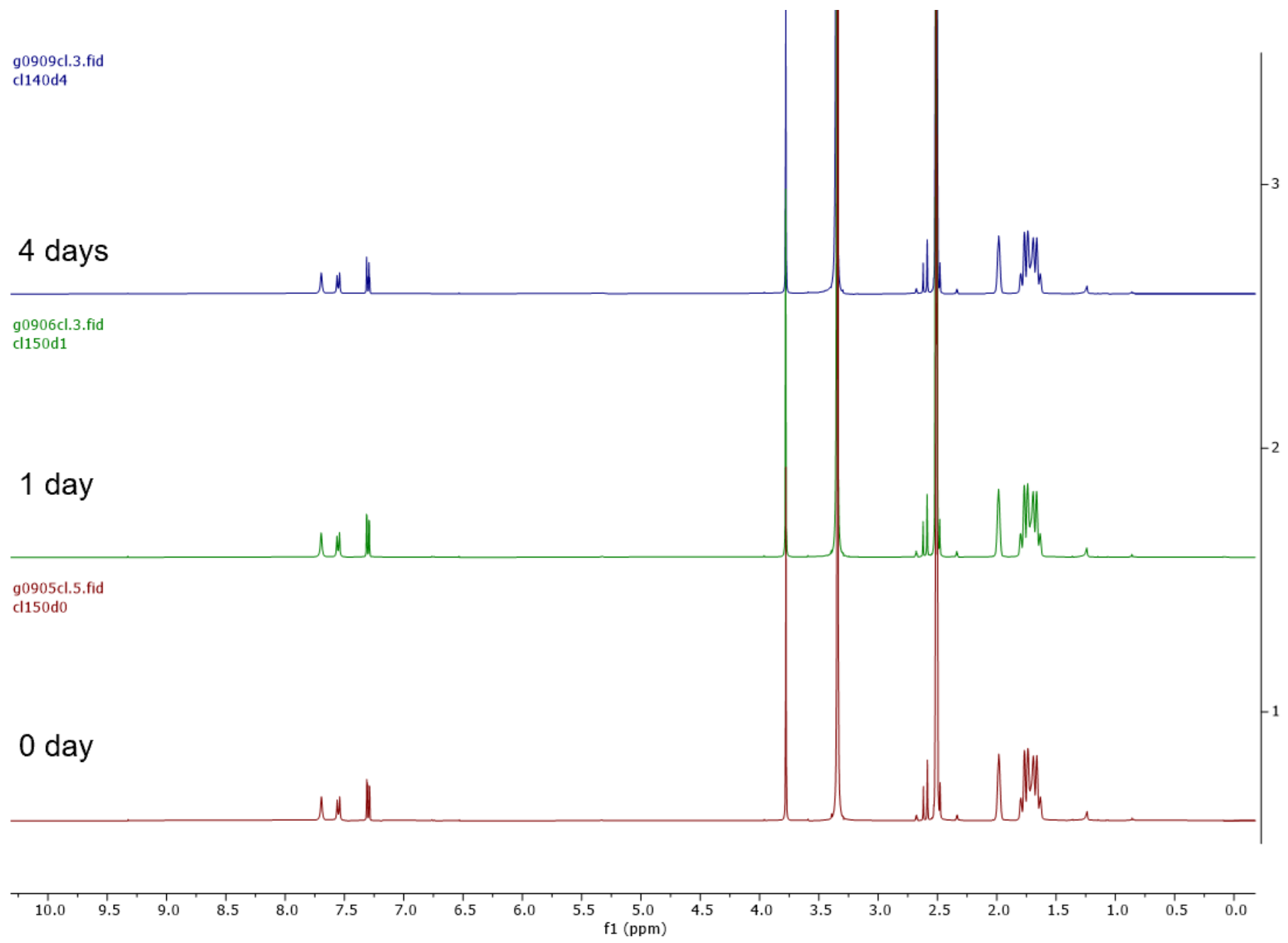

Figure S97. ^{1}H NMR spectra of **6d** for stability in $(CD_3)_2SO$ up to 4 days.

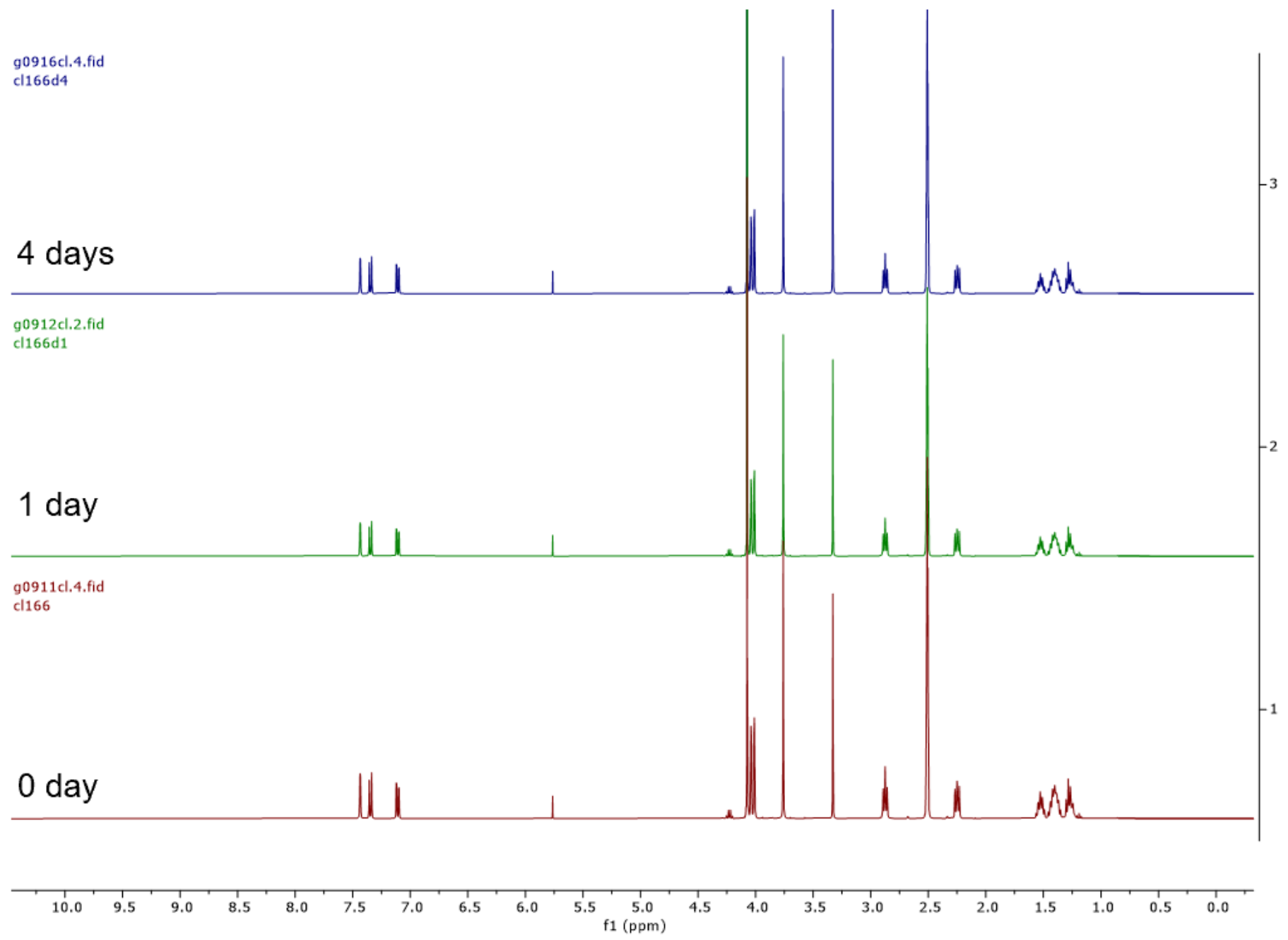

Figure S98. ^{1}H NMR spectra of **5e** for stability in $(CD_3)_2SO$ up to 4 days.

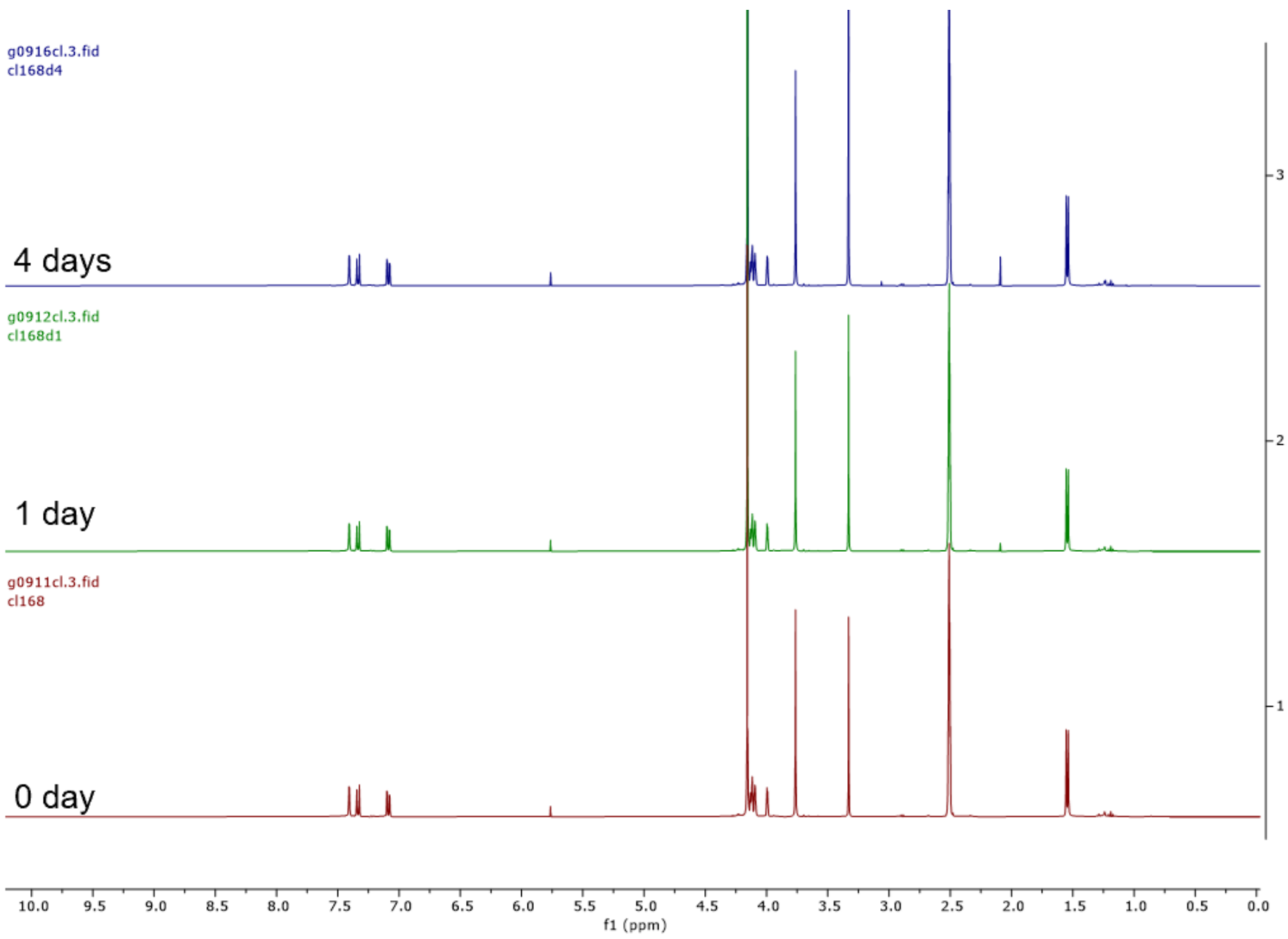

Figure S99. ^{1}H NMR spectra of **5f** for stability in $(CD_3)_2SO$ up to 4 days.

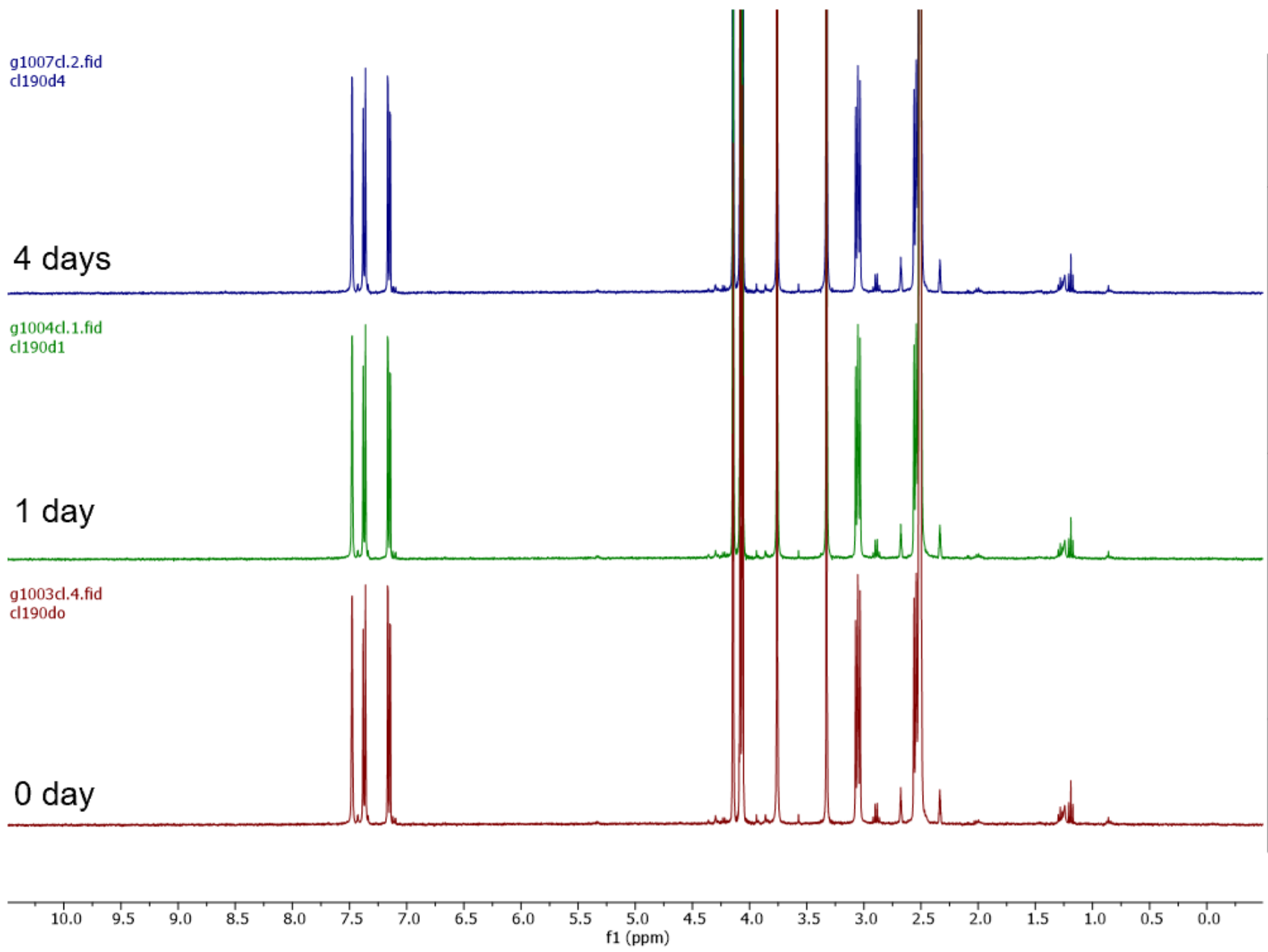

Figure S100. ^{1}H NMR spectra of **5g** for stability in $(CD_3)_2SO$ up to 4 days.

1.7. Infrared spectra

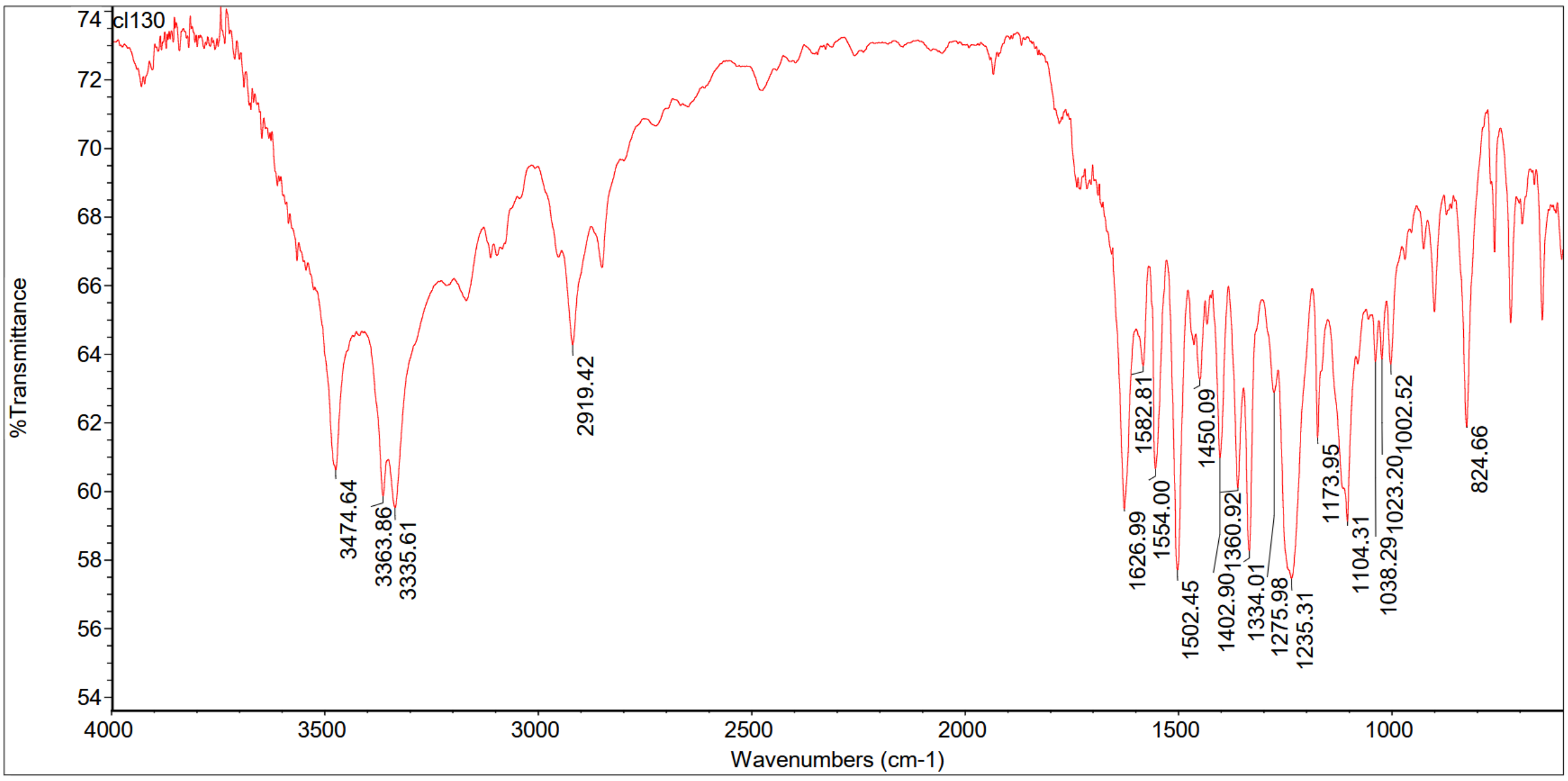

Figure S101. Infrared spectrum of compound **3a**.

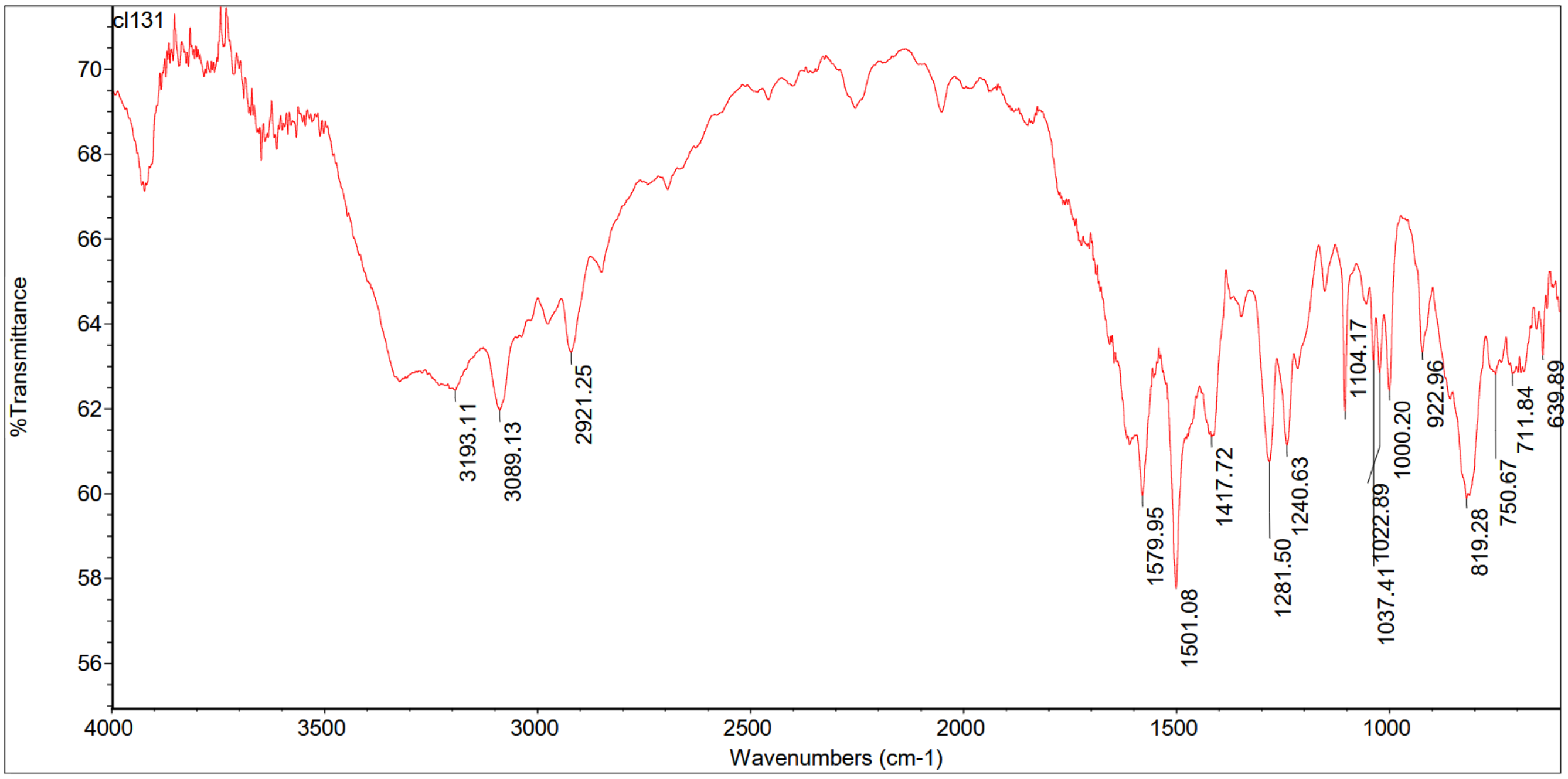

Figure S102. Infrared spectrum of compound **4a**.

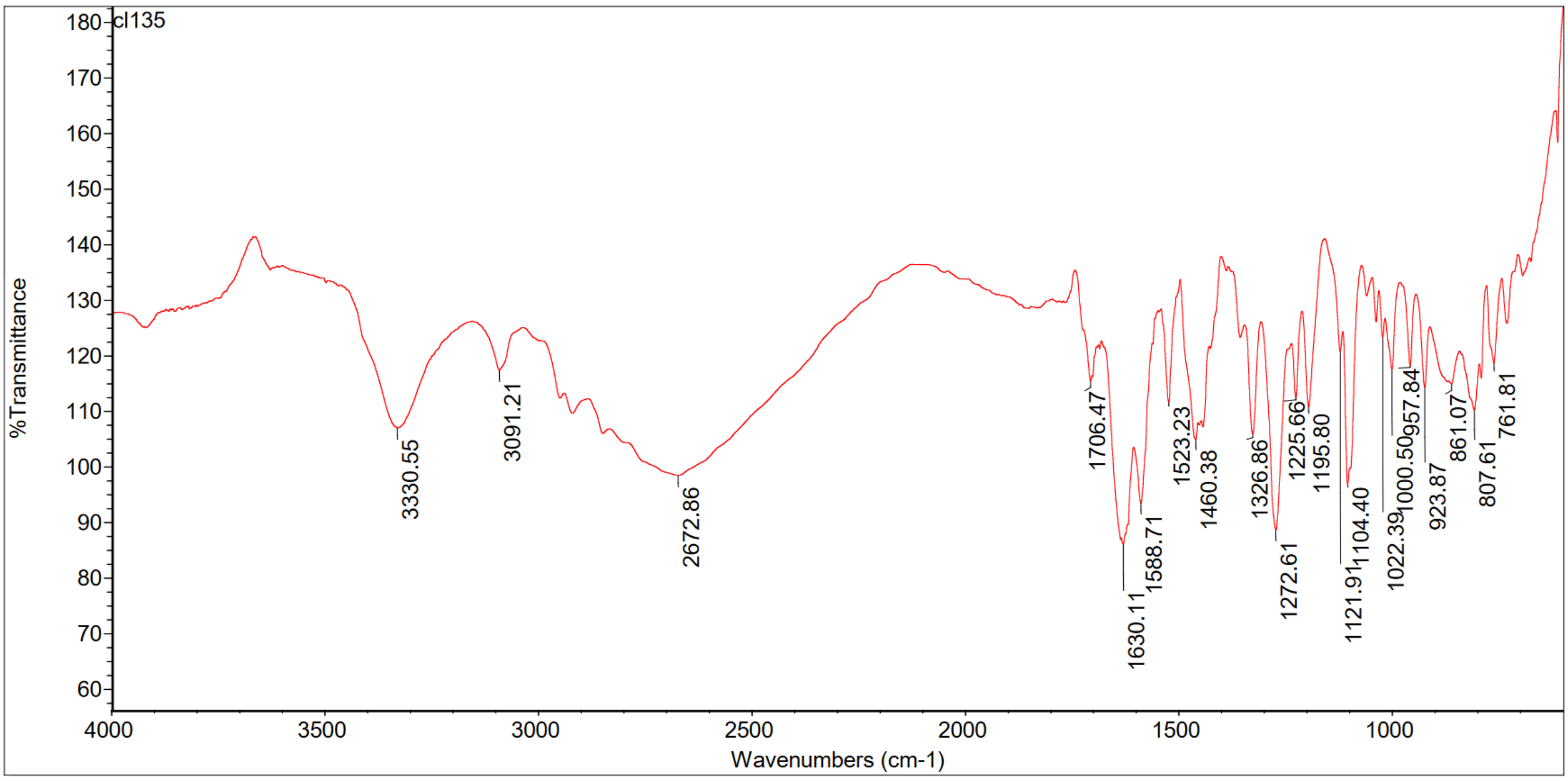

Figure S103. Infrared spectrum of **5a**.

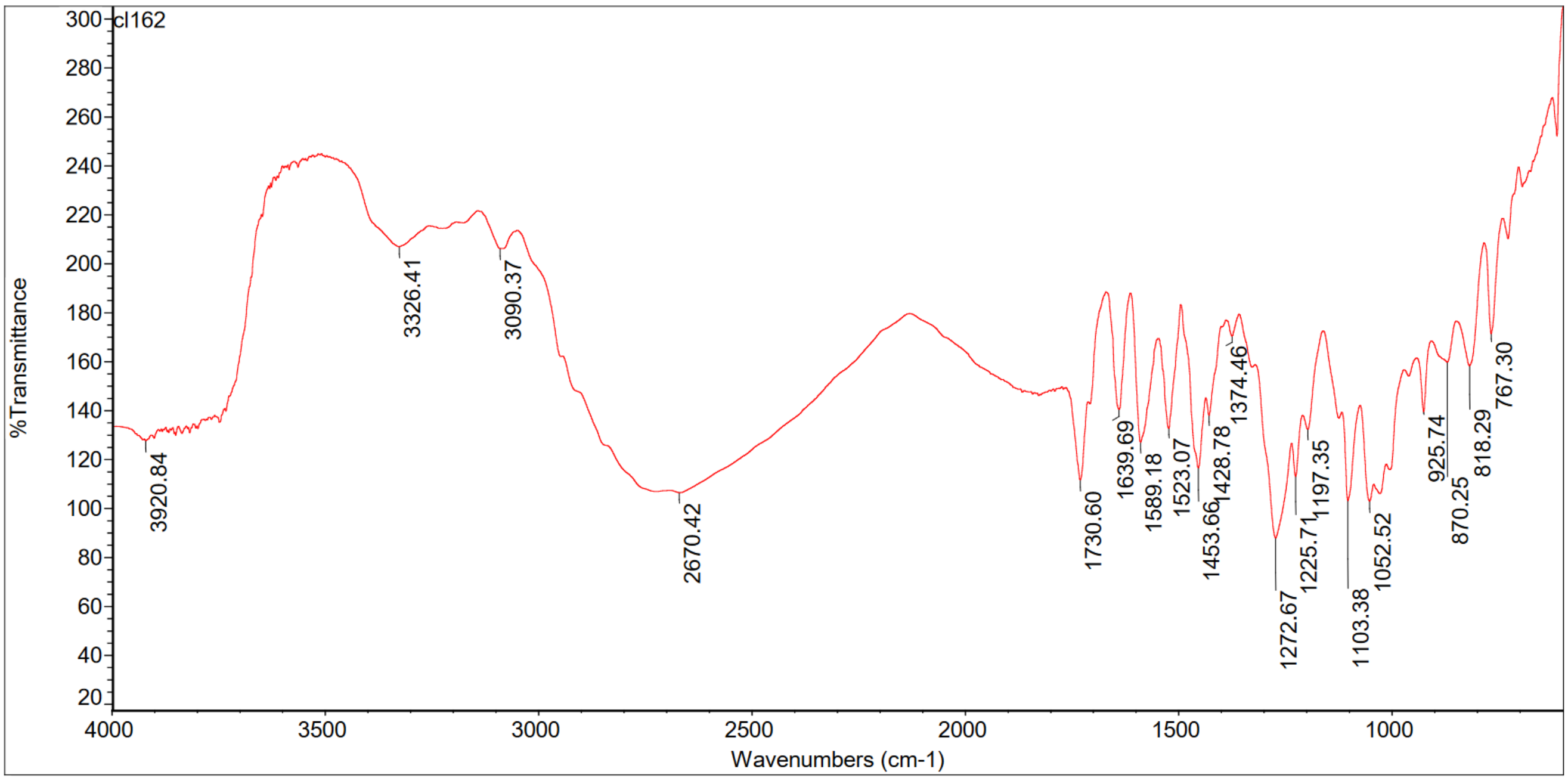

Figure S104. Infrared spectrum of **6a**.

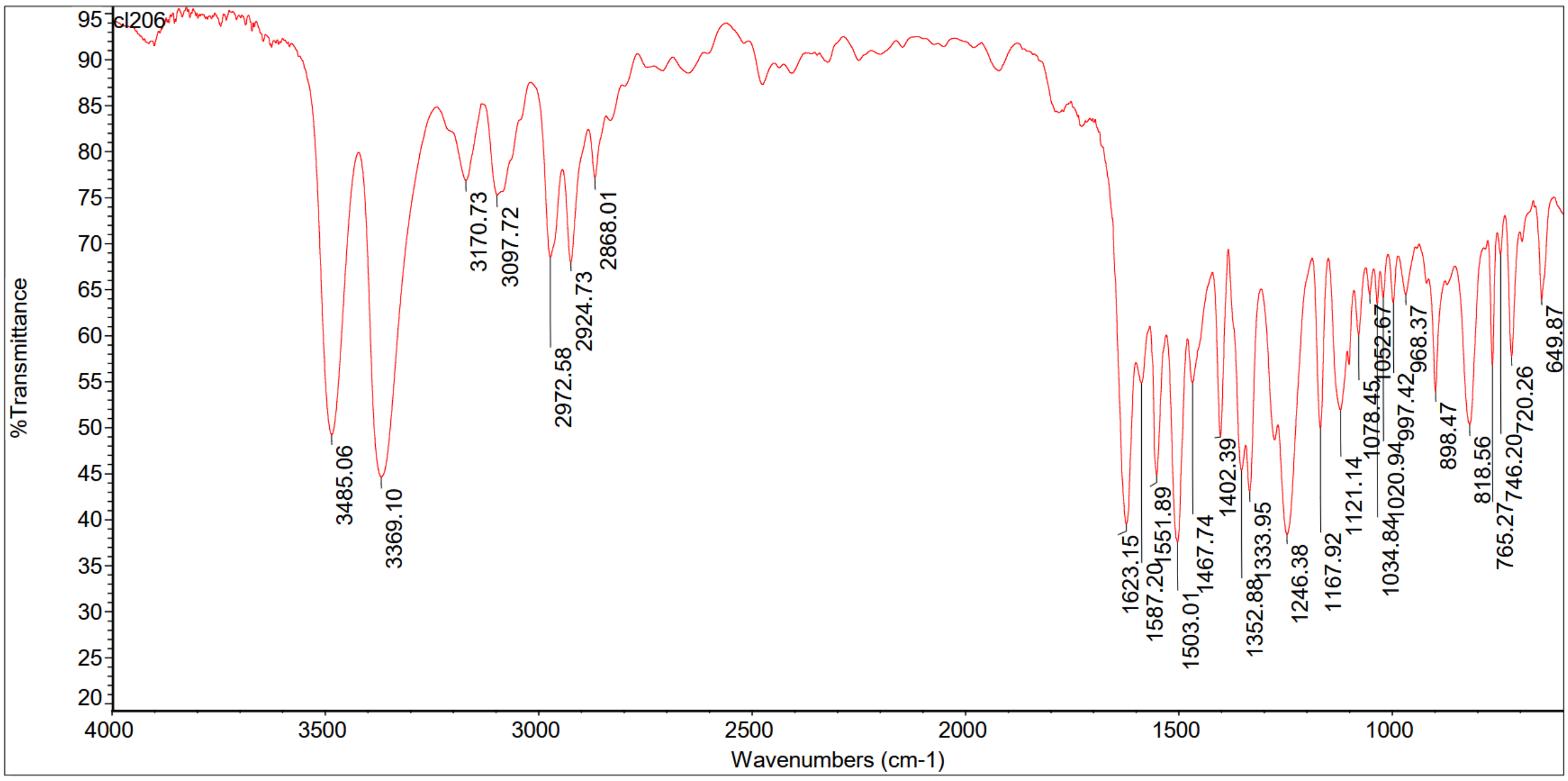

Figure S105. Infrared spectrum of **3b**.

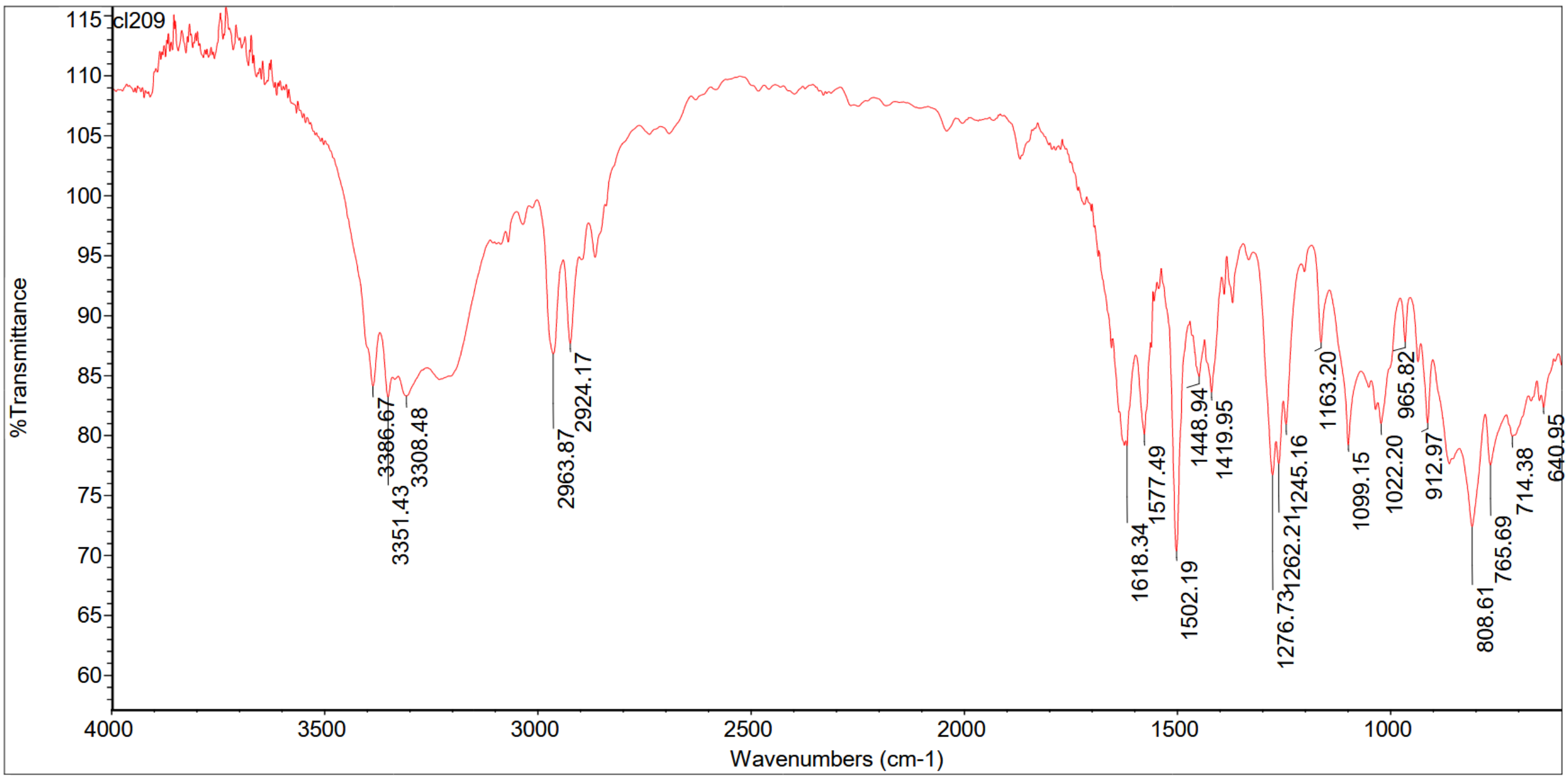

Figure S106. Infrared spectrum of **4b**.

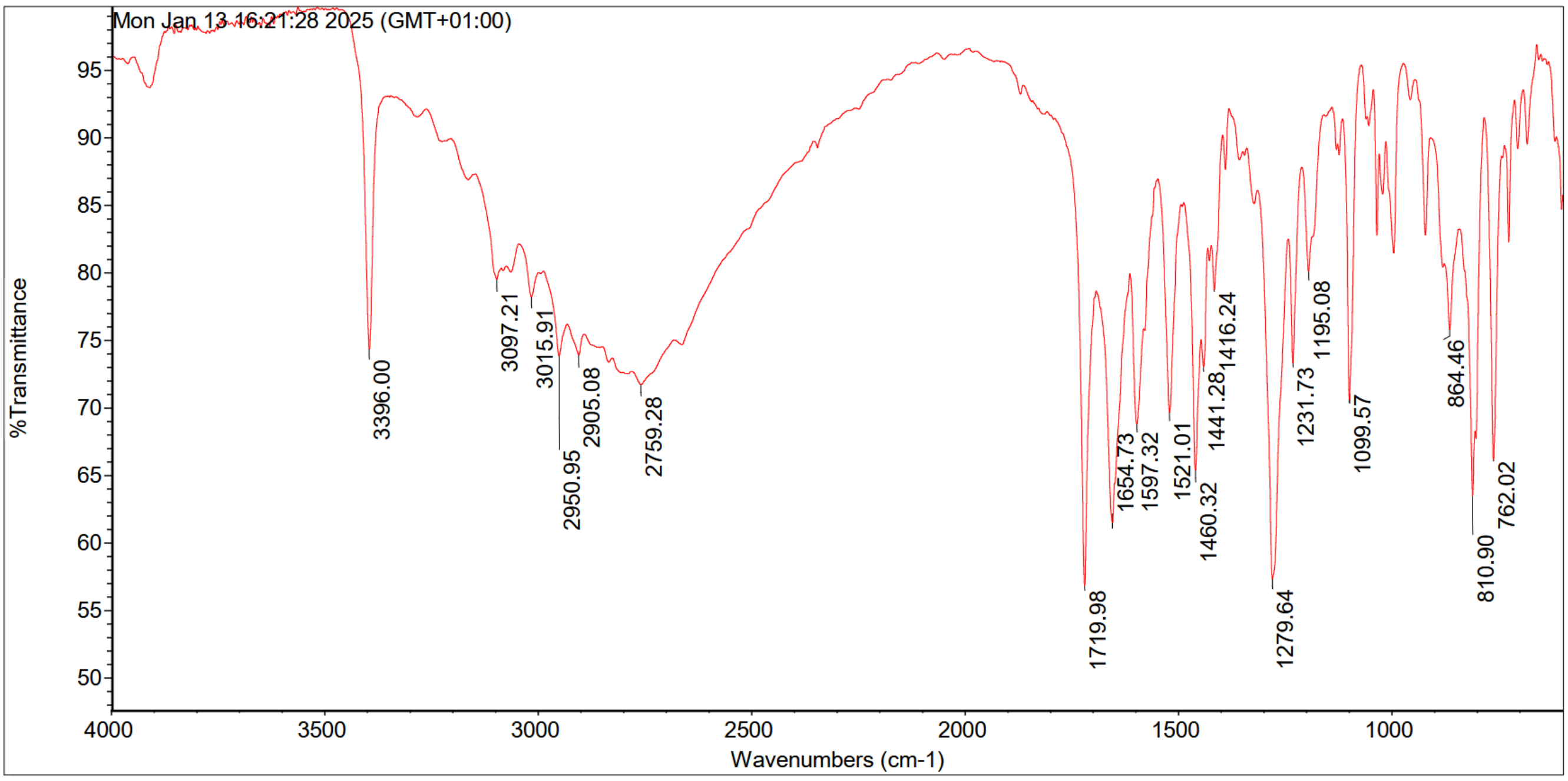

Figure S107. Infrared spectrum of **5b**.

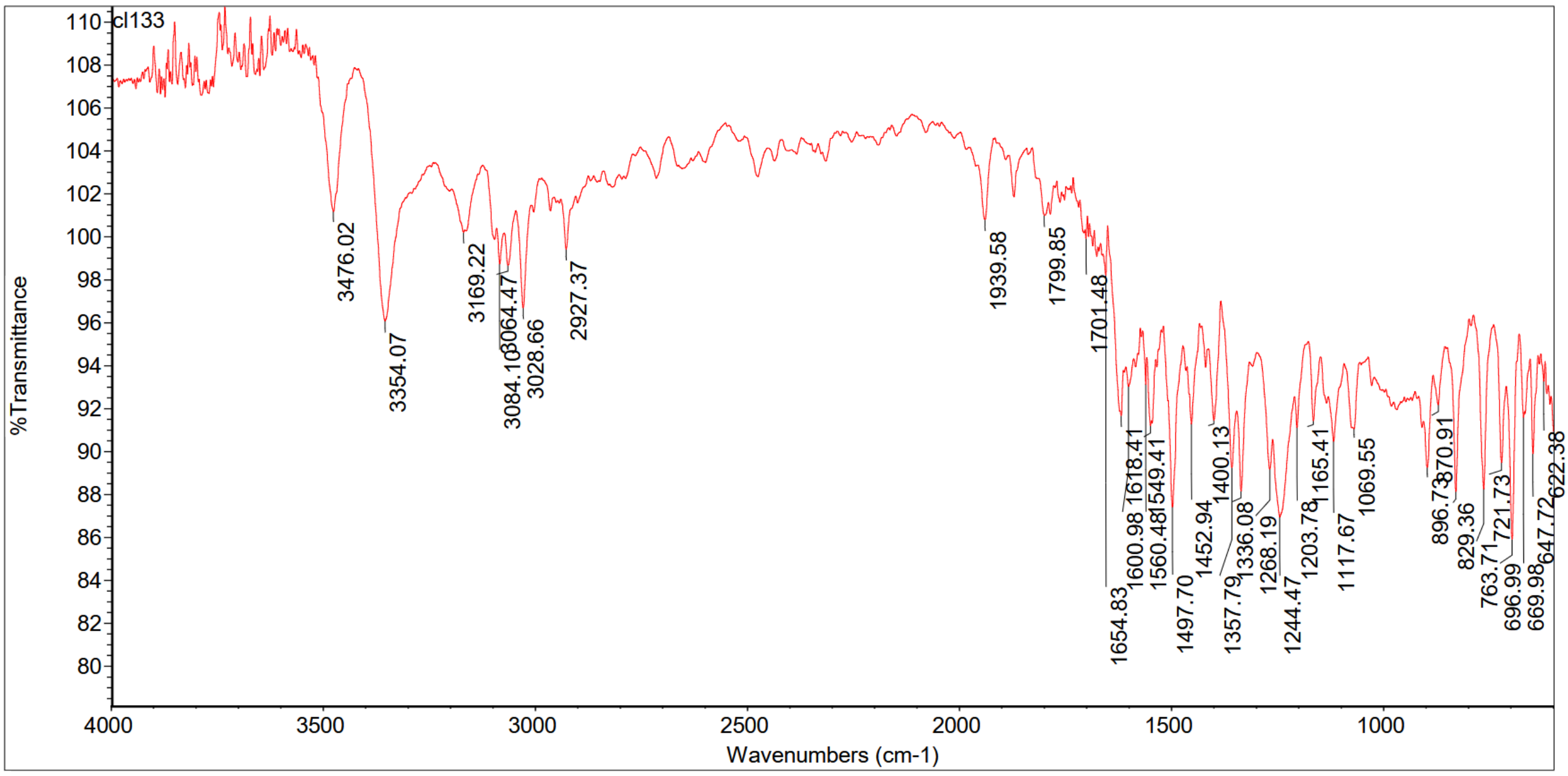

Figure S108. Infrared spectrum of **3c**.

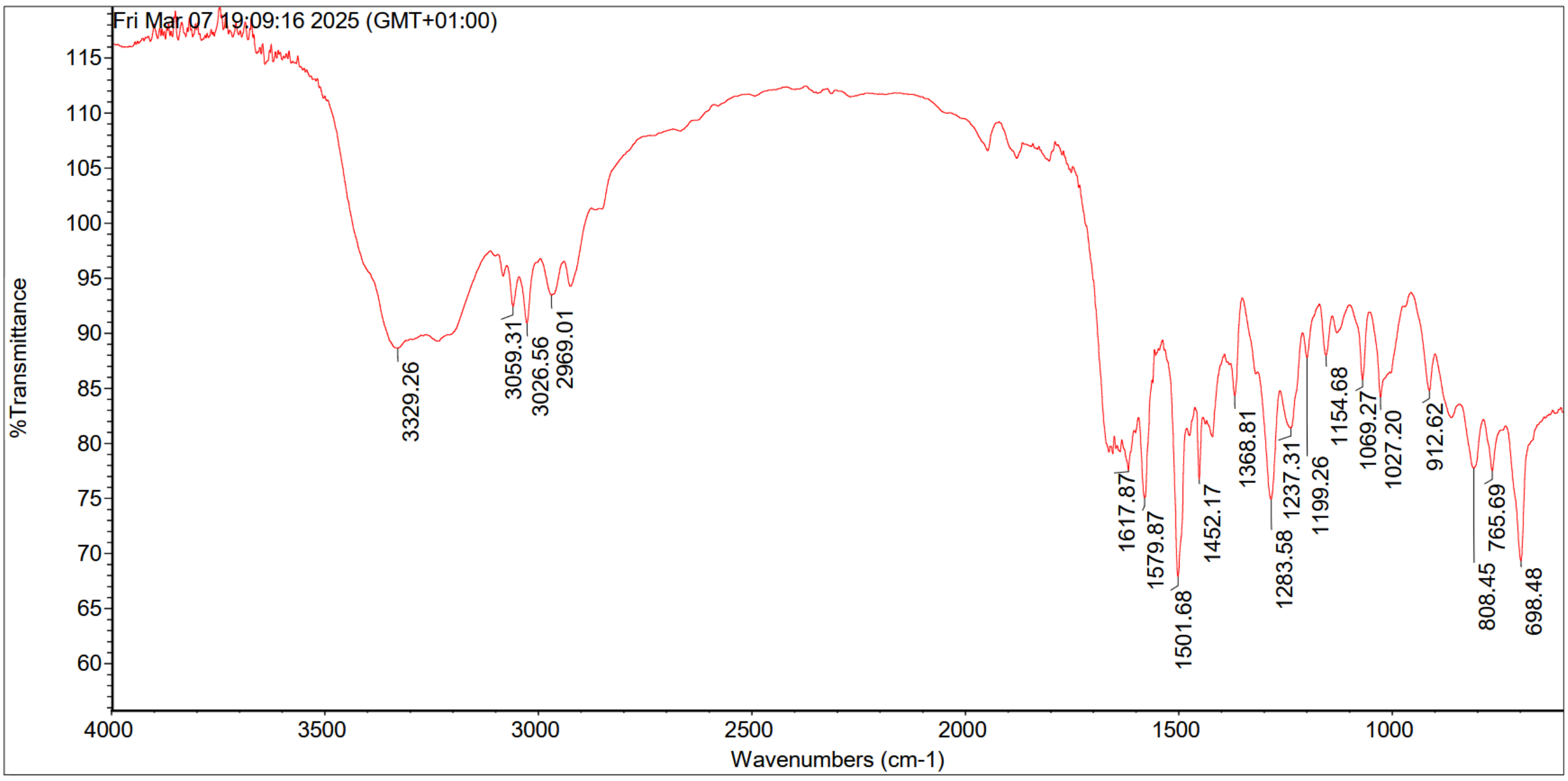

Figure S109. Infrared spectrum of **4c**.

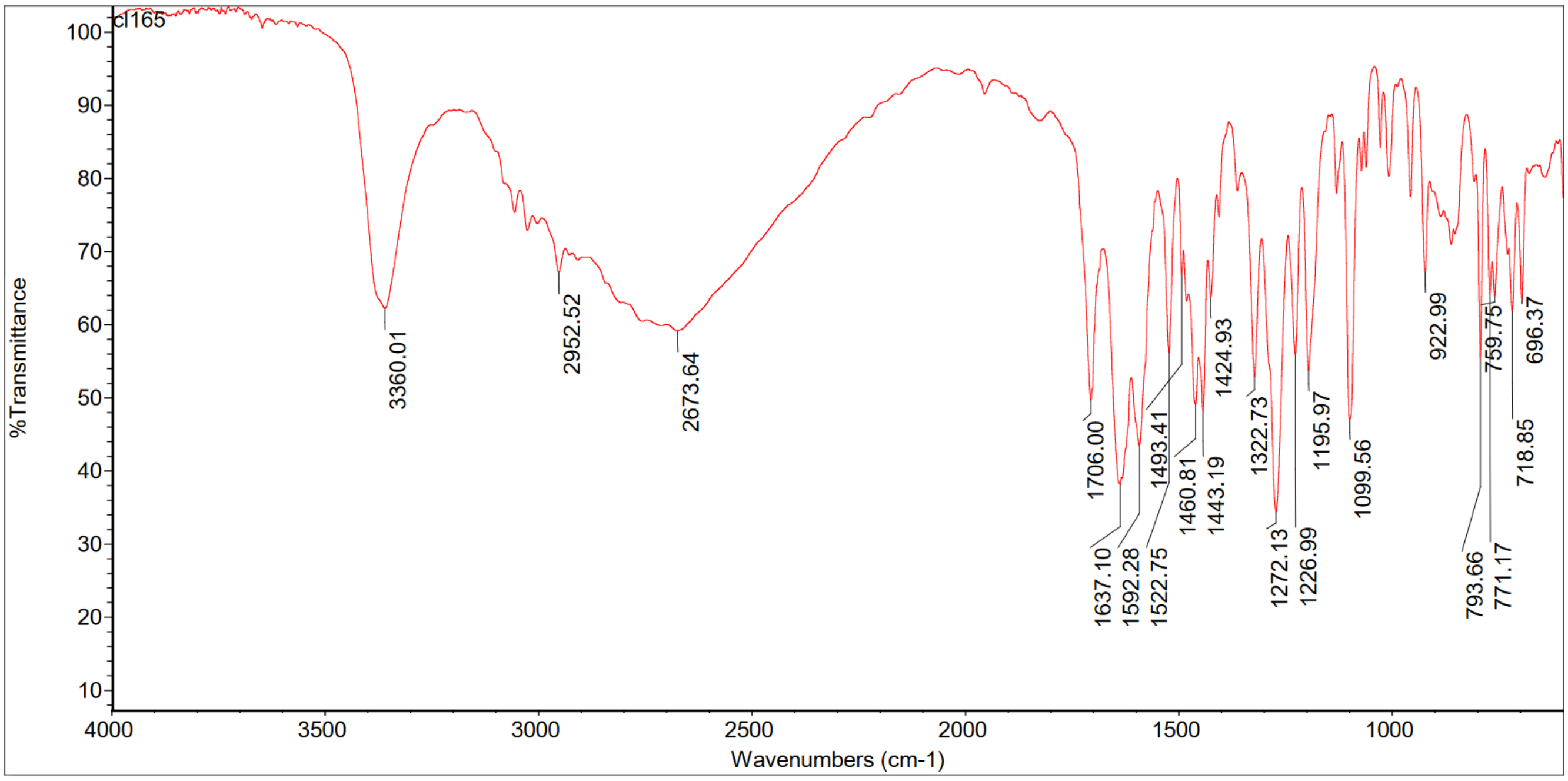

Figure S110. Infrared spectrum of **5c**.

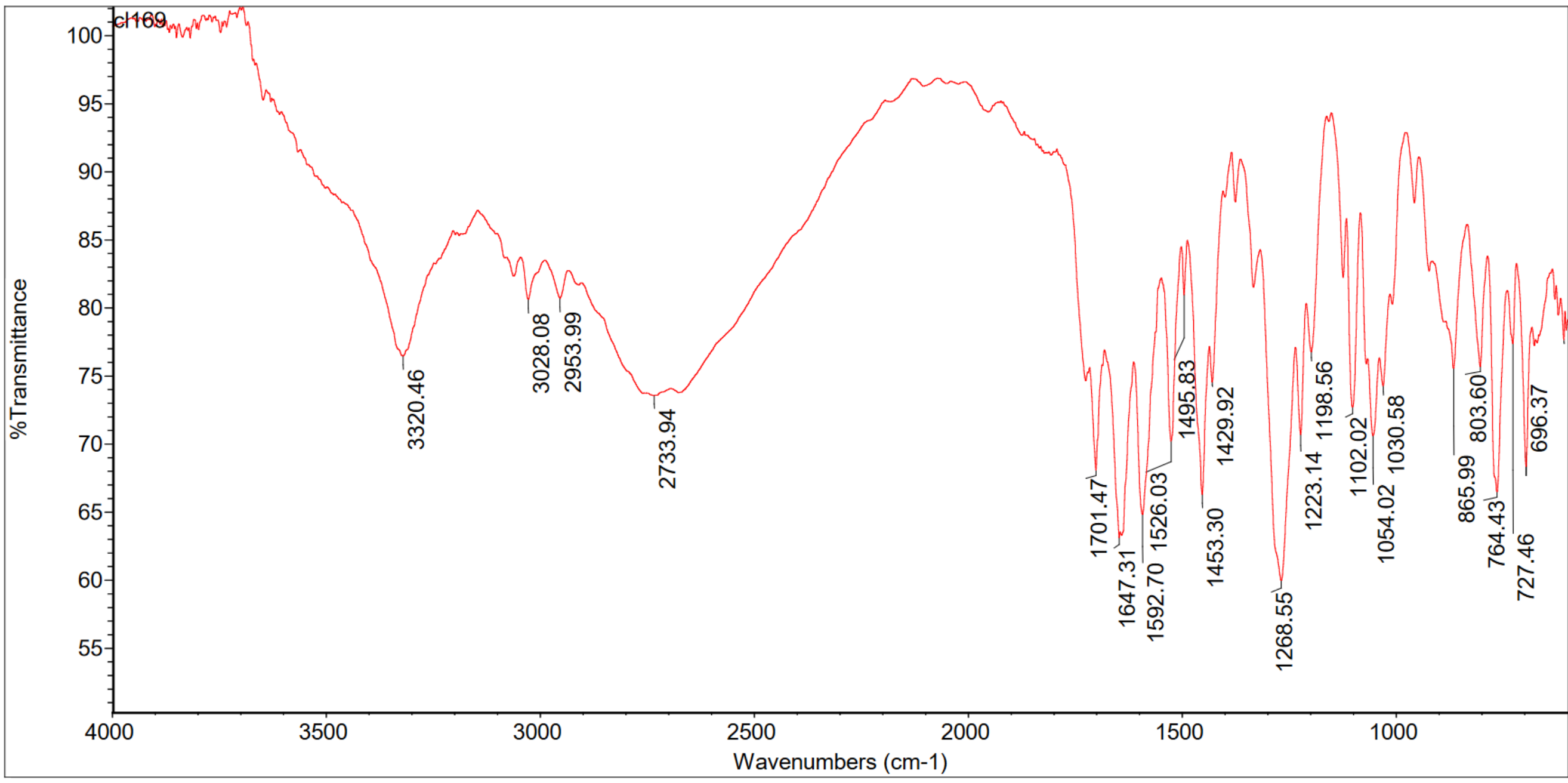

Figure S111. Infrared spectrum of **6c**

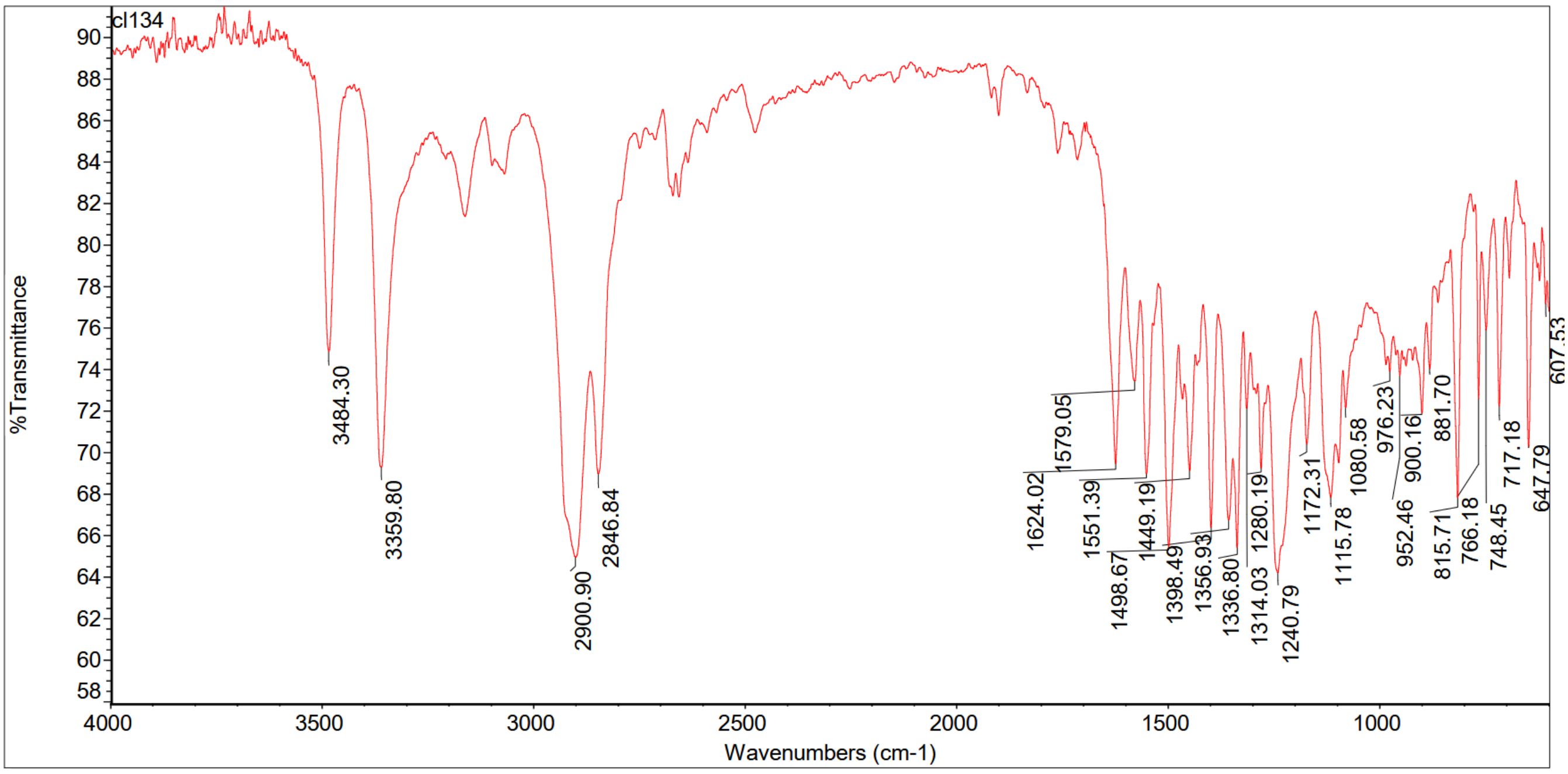

Figure S112. Infrared spectrum of **3d**.

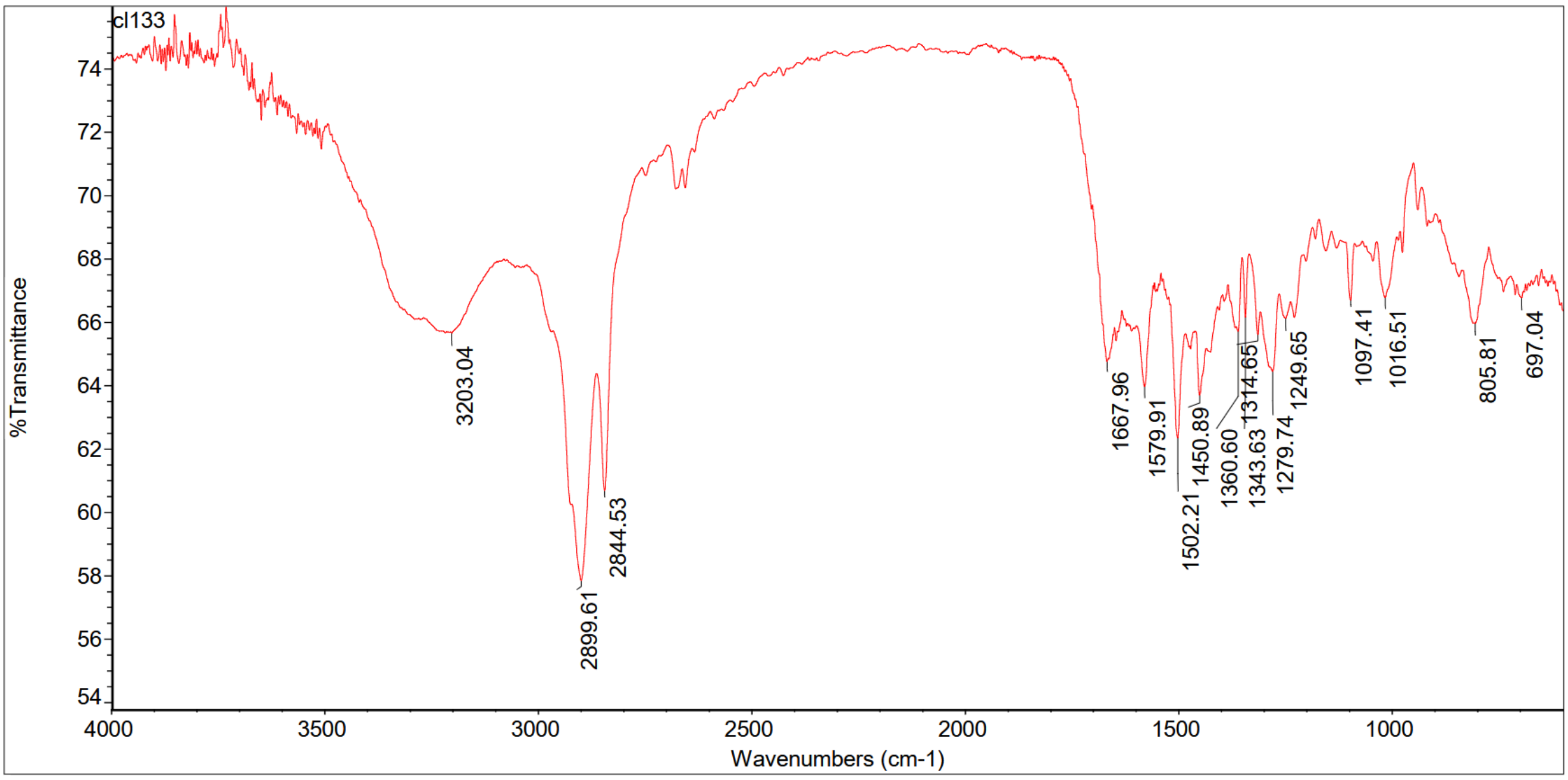

Figure S113. Infrared spectrum of **4d**.

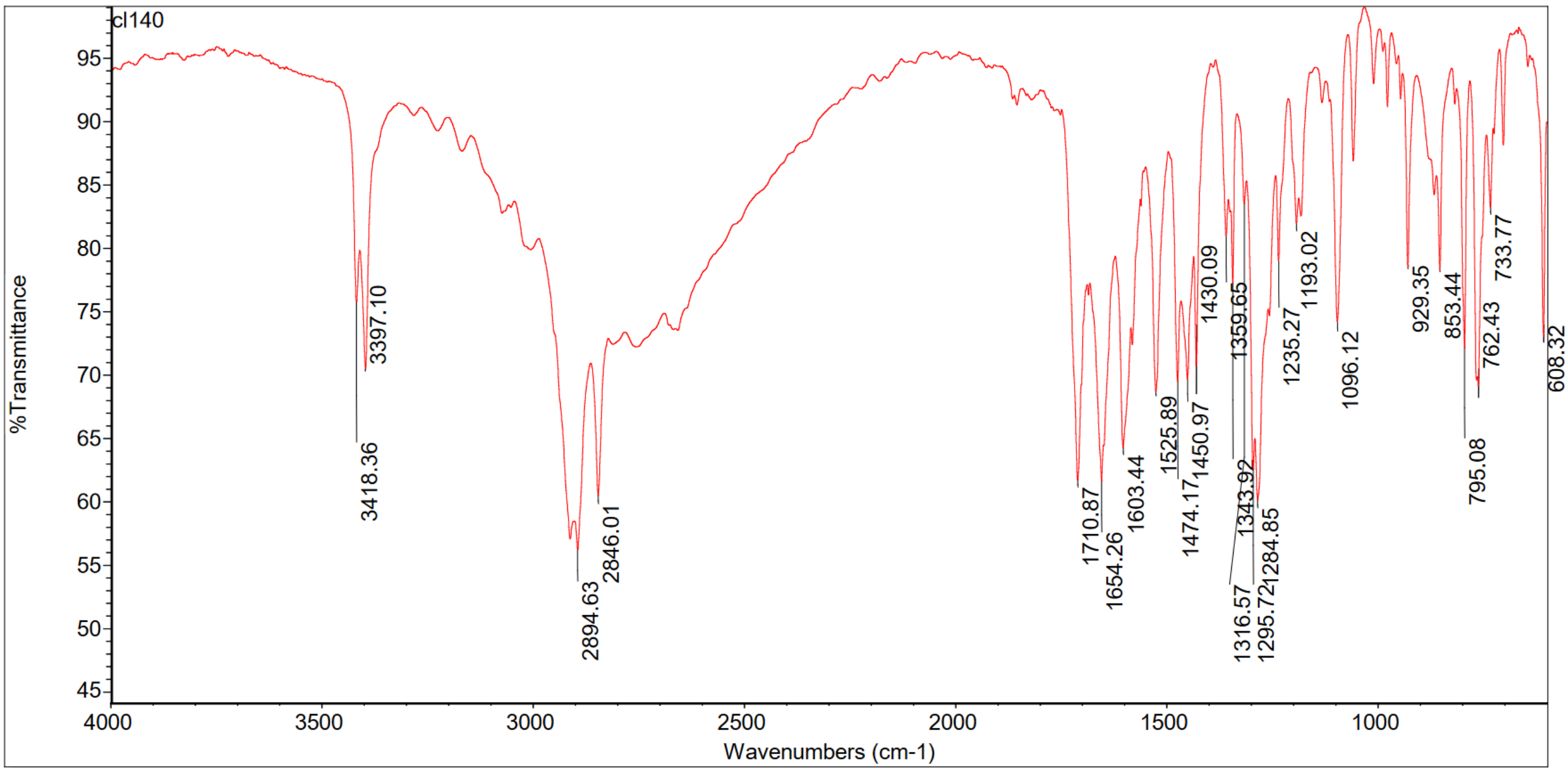

Figure S114. Infrared spectrum of **5d**.

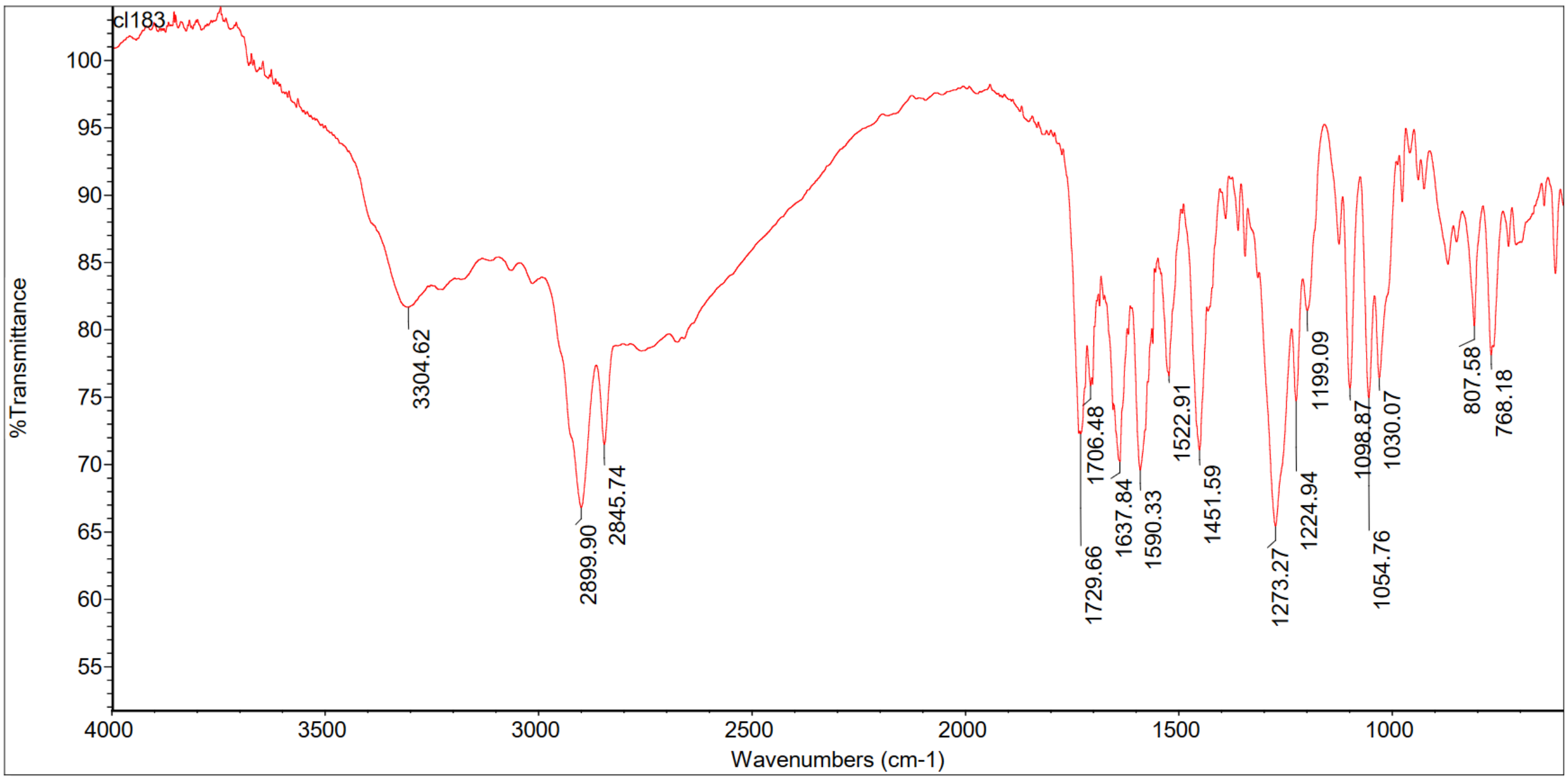

Figure S115. Infrared spectrum of **6d**.

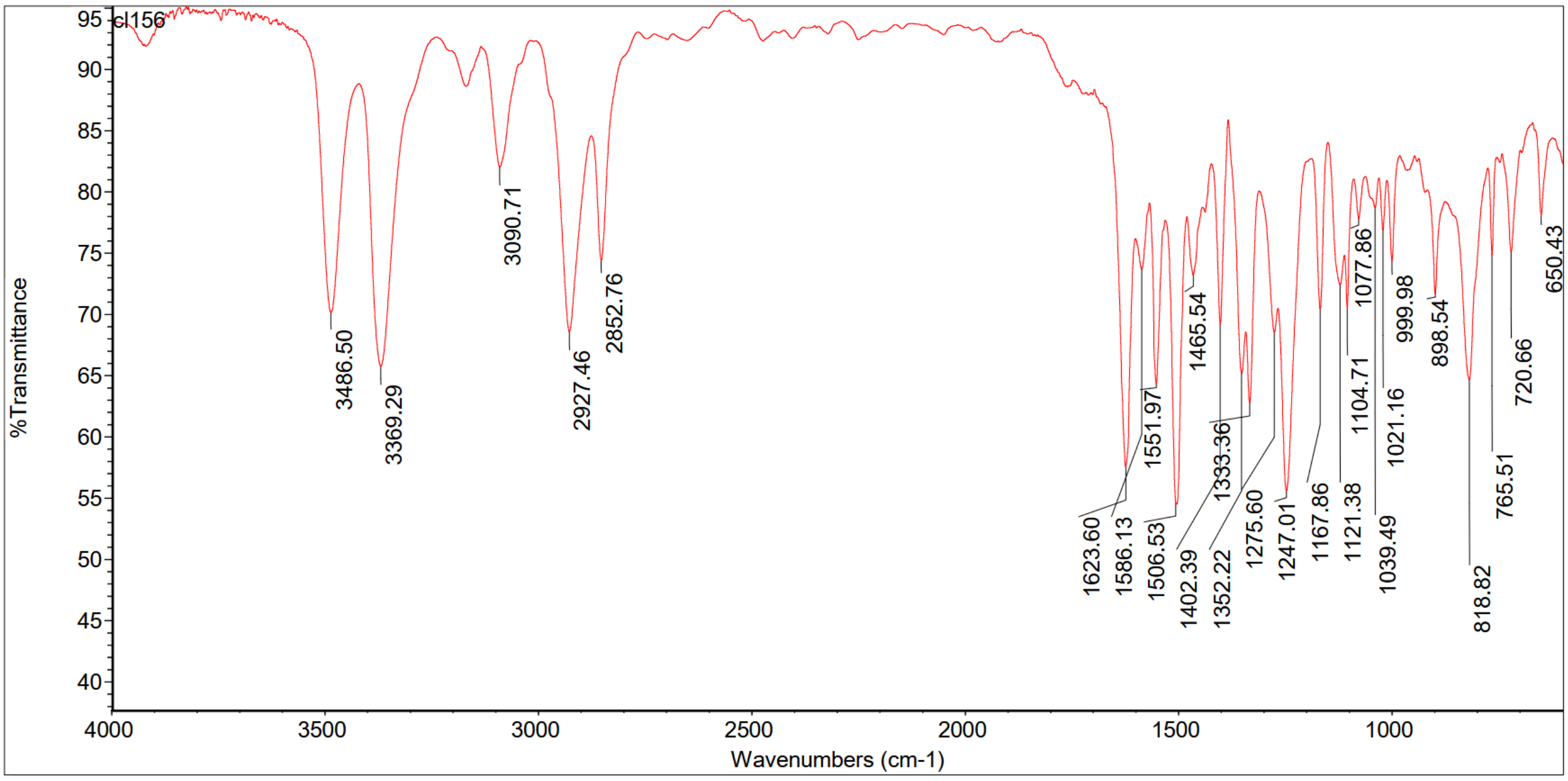

Figure S116. Infrared spectrum of **3e**.

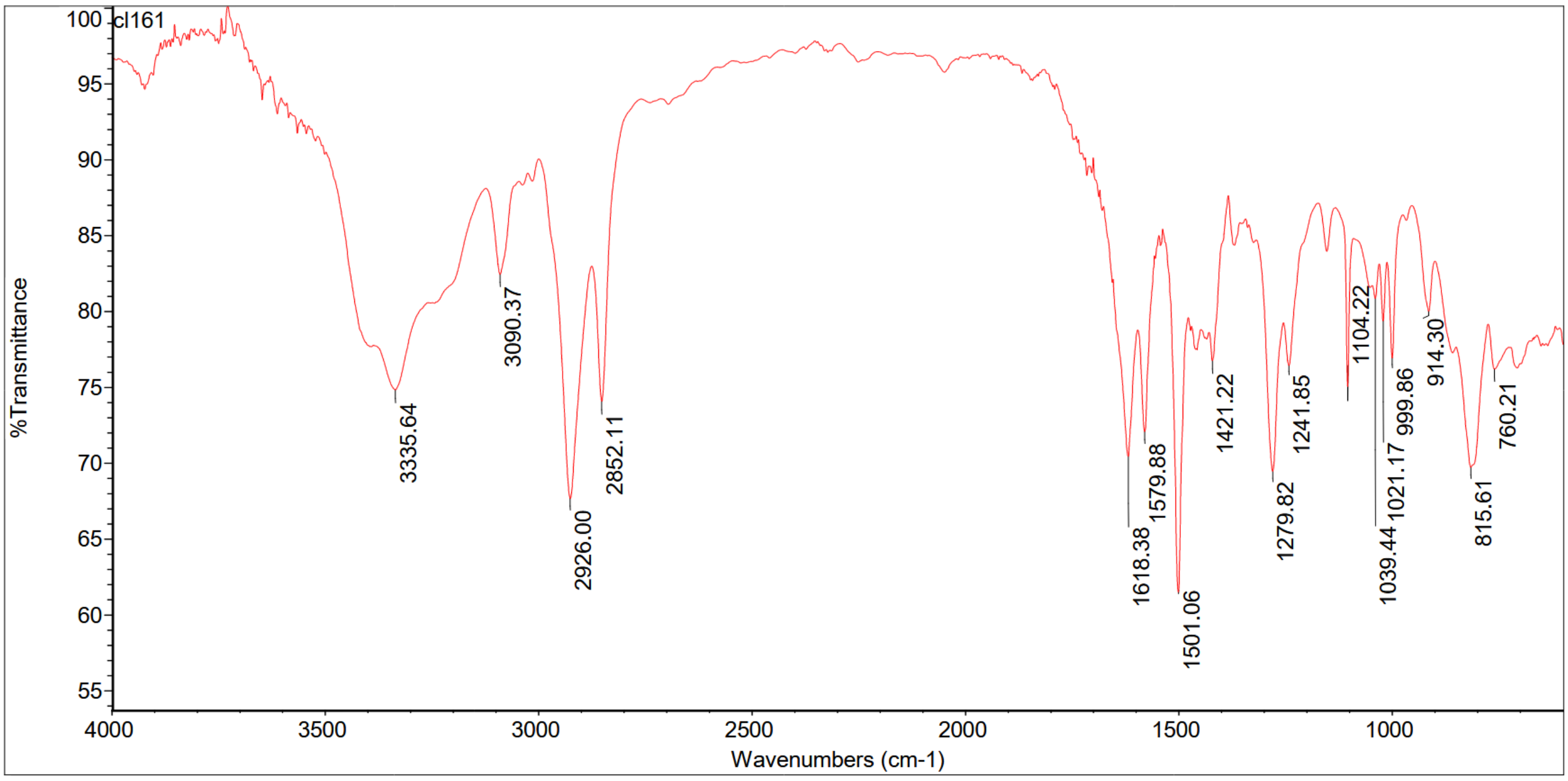

Figure S117. Infrared spectrum of **4e**.

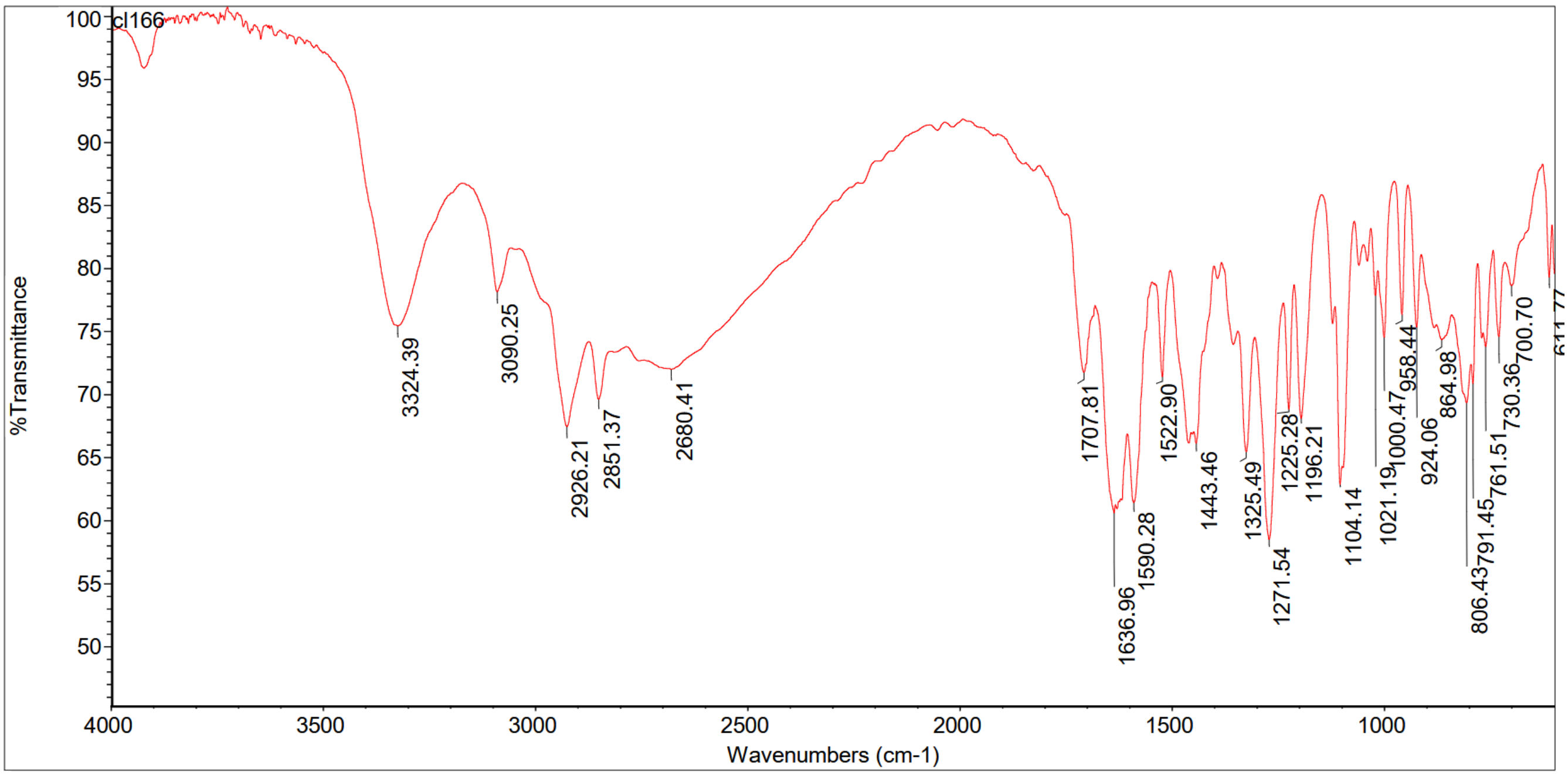

Figure S118. Infrared spectrum of **5e**.

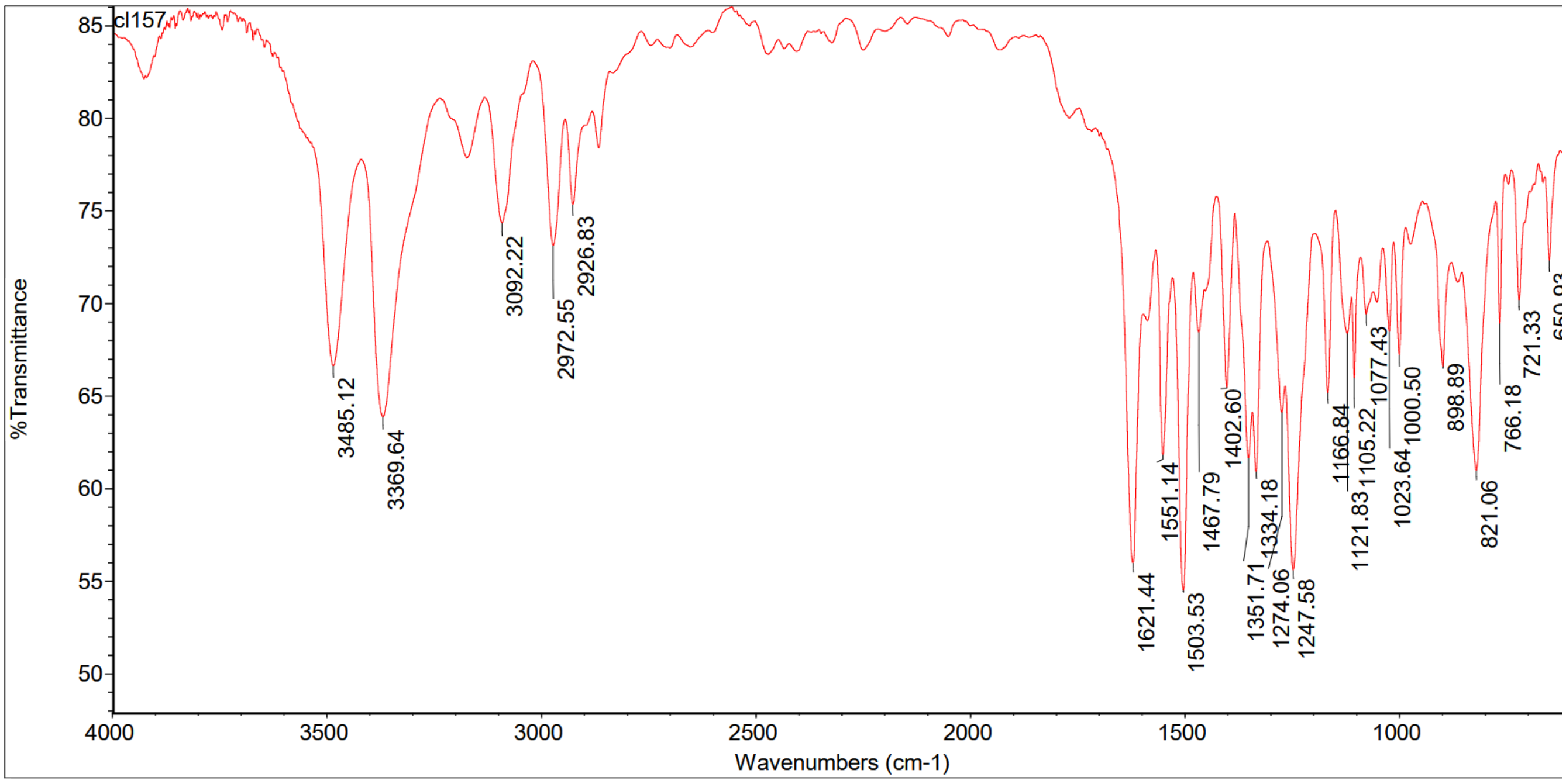

Figure S119. Infrared spectrum of **3f**.

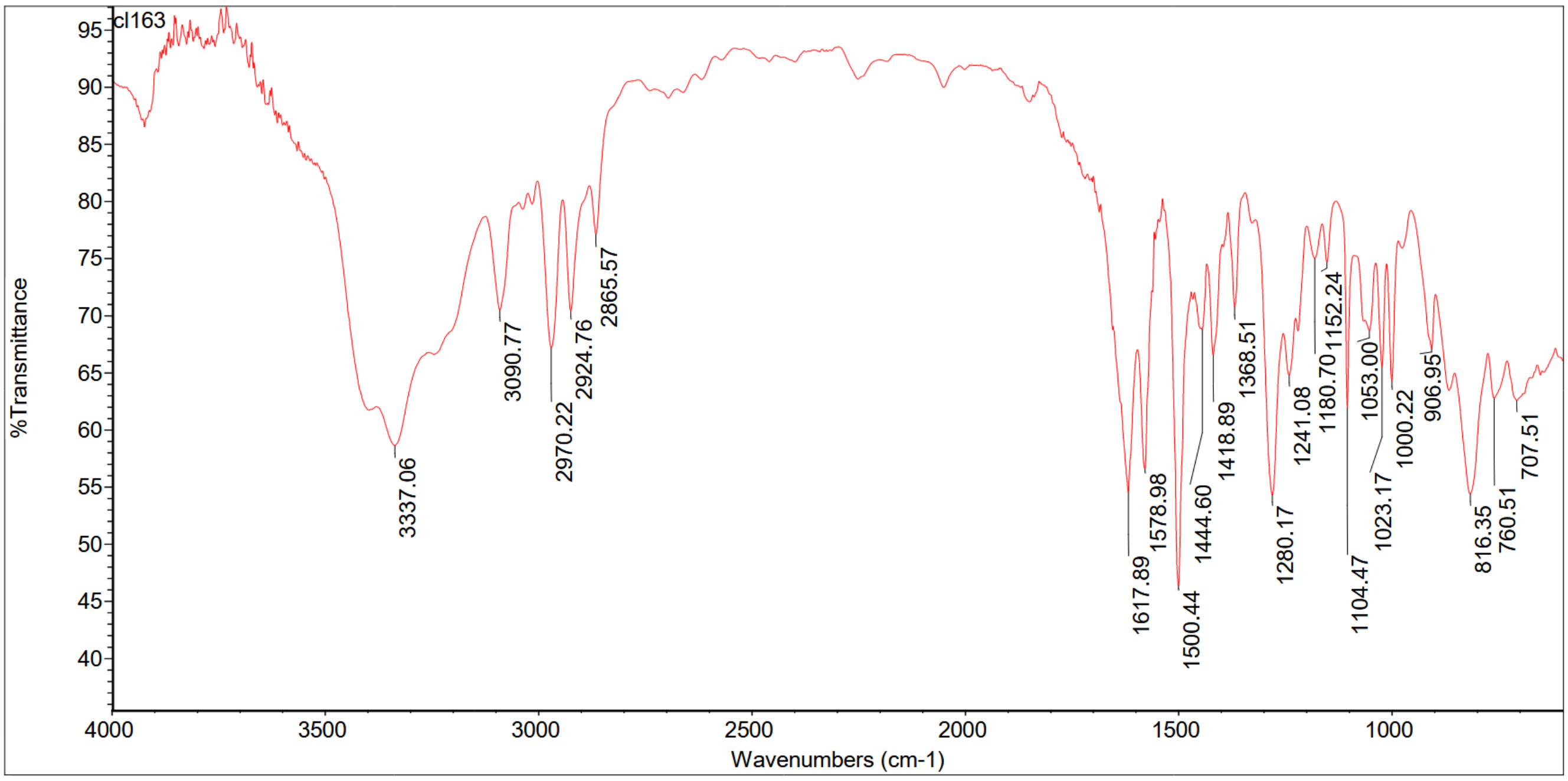

Figure S120. Infrared spectrum of **4f**.

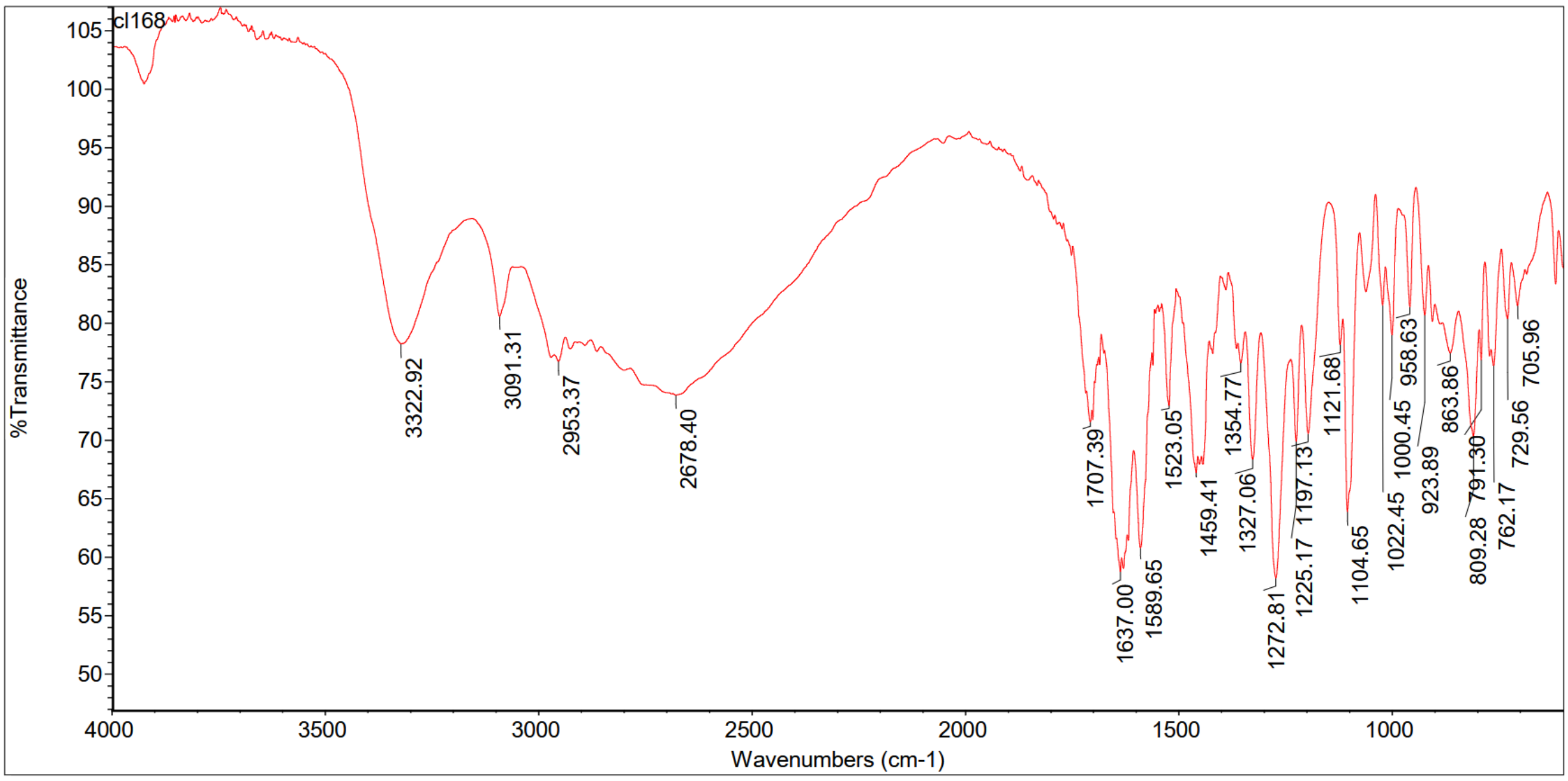

Figure S121. Infrared spectrum of **5f**.

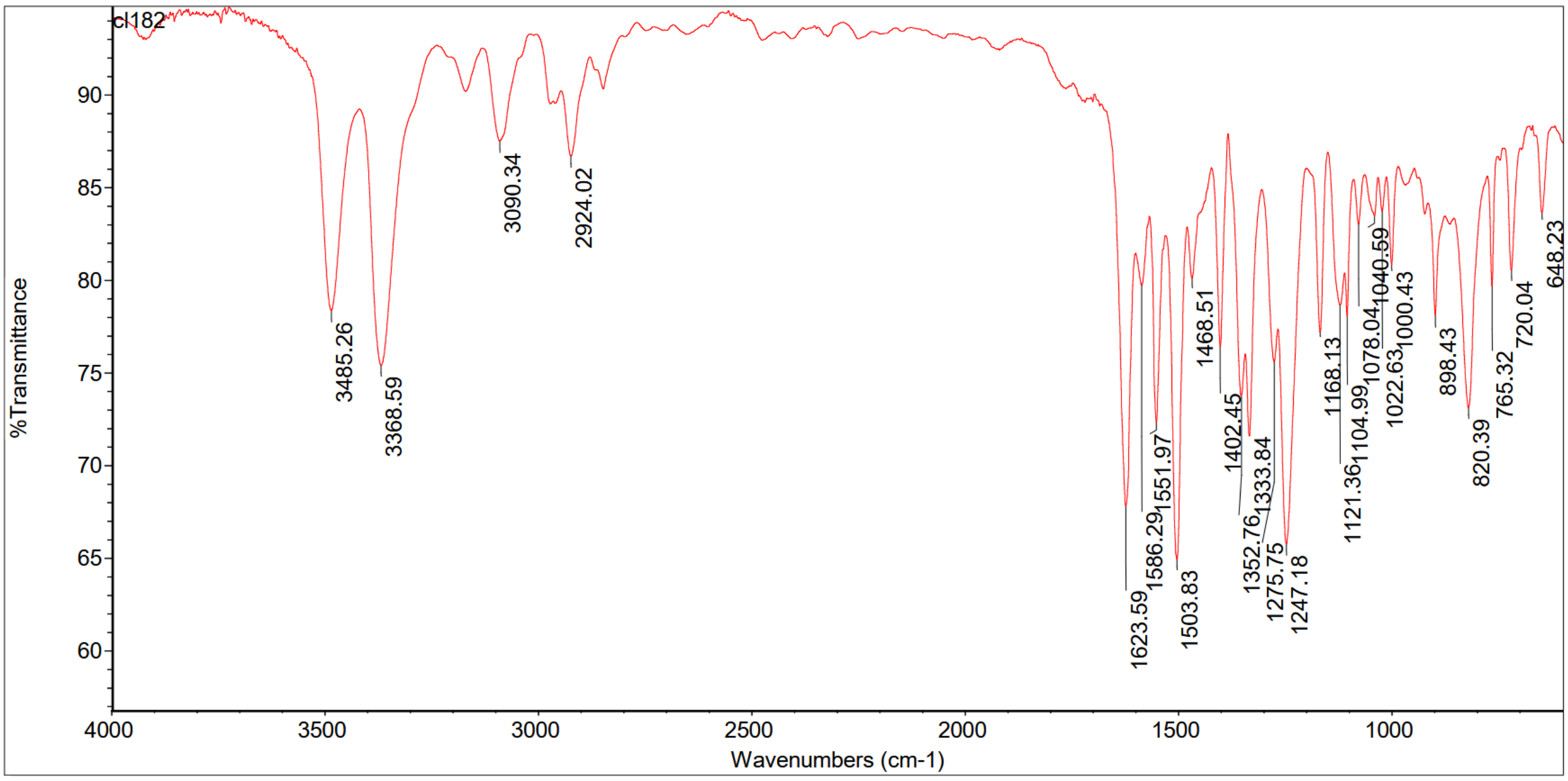

Figure S122. Infrared spectrum of **3g**.

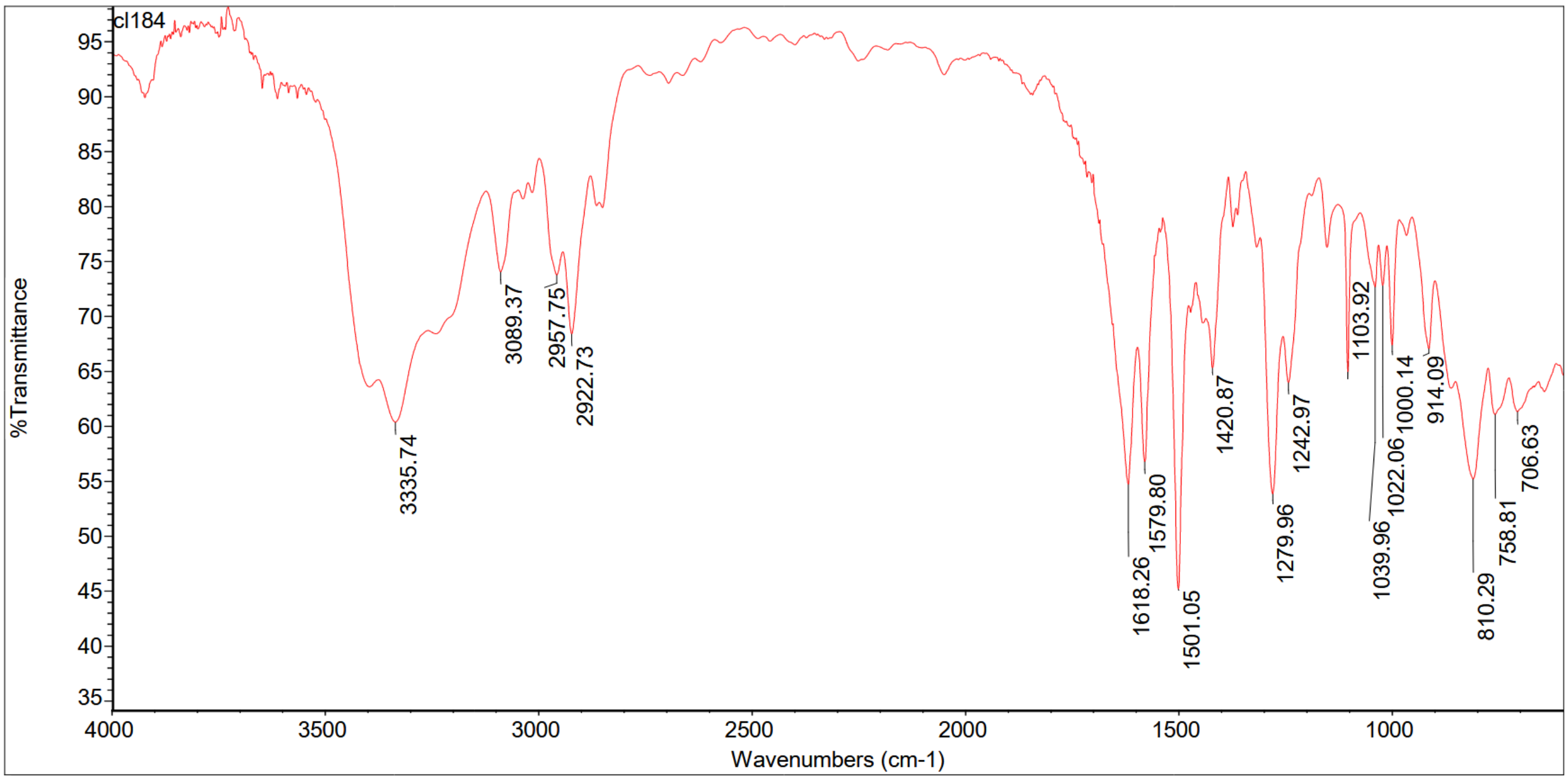

Figure S123. Infrared spectrum of **4g**.

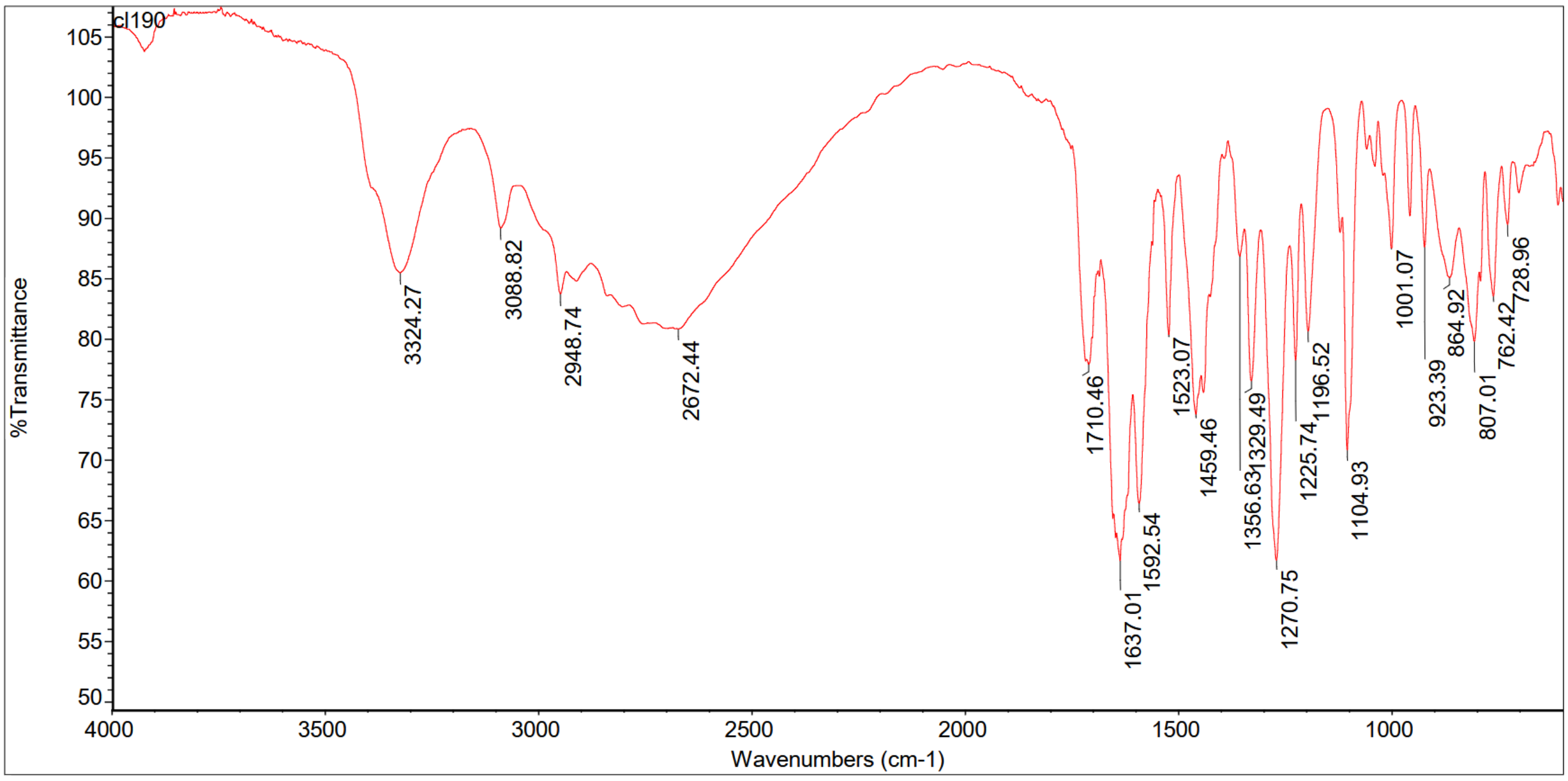

Figure S124. Infrared spectrum of **5g**.

1.8. X-ray crystallography data

Single crystal X-ray diffraction data were collected at 160.0(1) K on a Rigaku OD Synergy/Hypix diffractometer using the Cu Kα radiation (λ = 1.54184 Å) from a dual wavelength X-ray source and an Oxford Instruments Cryojet XL cooler. The selected suitable single crystal was mounted using polybutene oil on a flexible loop fixed on a goniometer head and immediately transferred to the diffractometer. Pre-experiment, data collection, data reduction and analytical absorption correction[11] were performed with the program suite CrysAlisPro.[12] Using Olex2,[13] the structure was solved with the SHELXT[14] small molecule structure solution program and refined with the SHELXL[15] program package by full-matrix least-squares minimization on F^2. PLATON[16] was used to check the result of the X-ray analysis. For more details about the data collection and refinement parameters, see the tables below.

There are two partial molecules in the asymmetric unit. Most of the atoms reside on mirror planes with a site-occupancy factor of 0.5.

Table S1. Crystal data and structure refinement for **5d.**

Identification code	**5d**
Empirical formula	$C_{20}H_{25}N_3O_2S$
Formula weight	371.49
Temperature/K	160.0(1)
Crystal system	monoclinic
Space group	C2/m
a/Å	49.1582(6)
b/Å	6.77525(8)
c/Å	10.97866(11)
α/°	90
β/°	94.6368(11)
γ/°	90
Volume/Å^3	3644.58(7)
Z	8

ρ_{calc}g/cm^3	1.354
μ/mm^{-1}	1.737
F(000)	1584.0
Crystal size/mm^3	0.11 × 0.1 × 0.04
Radiation	Cu Kα (λ = 1.54184)
2Θ range for data collection/°	7.216 to 158.978
Index ranges	-62 ≤ h ≤ 60, -7 ≤ k ≤ 8, -13 ≤ l ≤ 14
Reflections collected	21560
Independent reflections	4233 [R_{int} = 0.0216, R_{sigma} = 0.0168]
Data/restraints/parameters	4233/0/307
Goodness-of-fit on F^2	1.044
Final R indexes [I>=2σ (I)]	R_1 = 0.0460, wR_2 = 0.1393
Final R indexes [all data]	R_1 = 0.0489, wR_2 = 0.1421
Largest diff. peak/hole / e Å^{-3}	0.40/-0.54

Table S2. Fractional Atomic Coordinates (×10^4) and Equivalent Isotropic Displacement Parameters (Å^2×10^3) for **5d.** U_{eq} is defined as 1/3 of the trace of the orthogonalised U_{IJ} tensor.

**Atom**	***x***	***y***	***z***	**U(eq)**
S1	3910.3(2)	5000	9418.8(5)	47.6(2)
O1	1929.8(3)	5000	8863.1(14)	31.9(4)
O2	2221.3(3)	5000	10562.9(14)	34.8(4)
N1	2373.6(4)	5000	8636.9(17)	30.9(4)
N2	2746.0(4)	5000	10191.4(17)	30.0(4)
N3	2840.4(4)	5000	8222.7(17)	29.0(4)
C1	1707.8(4)	5000	9651(2)	34.2(5)
C2	2178.3(4)	5000	9453(2)	28.5(5)
C3	2649.0(4)	5000	8998(2)	28.3(4)
C4	3027.8(4)	5000	10203(2)	28.1(4)

Table S2. Fractional Atomic Coordinates (×10[4]) and Equivalent Isotropic Displacement Parameters (Å[2]×10[3]) for **5d.** U_{eq} is defined as 1/3 of the trace of the orthogonalised U_{IJ} tensor.

**Atom**	***x***	***y***	***z***	**U(eq)**
C5	3083.1(4)	5000	8969.7(19)	27.0(4)
C6	3353.0(4)	5000	8659(2)	29.5(5)
C7	3557.7(4)	5000	9605(2)	30.3(5)
C8	3496.7(5)	5000	10840(2)	30.9(5)
C9	3230.1(5)	5000	11161(2)	31.1(5)
C10	3931.9(4)	5000	7776.3(19)	28.3(4)
C11	4231.7(4)	5000	7490.7(18)	22.1(4)
C12	4241.2(4)	5000	6096.4(18)	24.3(4)
C13	4537.7(4)	5000	5761.5(19)	29.8(5)
C14	4685.0(3)	6840(3)	6284.7(14)	35.4(4)
C15	4678.8(3)	6844(3)	7682.9(14)	32.4(4)
C16	4381.5(3)	6848(2)	8004.0(13)	28.8(3)
C17	4822.4(5)	5000	8216(2)	36.5(5)
S2	1205.1(2)	5000	5056.8(5)	29.62(16)
O3	3187.5(3)	5000	5394.6(14)	30.7(3)
O4	2899.9(3)	5000	3673.0(14)	32.4(4)
N4	2743.8(4)	5000	5588.9(18)	27.5(4)
N5	2363.2(4)	5000	4060.6(17)	28.8(4)
N6	2281.5(4)	5000	6041.5(16)	26.3(4)
C18	3415.8(5)	5000	4657(2)	34.0(5)
C19	2941.0(4)	5000	4781(2)	26.3(4)
C20	2468.1(4)	5000	5242.9(19)	25.2(4)
C21	2081.5(4)	5000	4082(2)	27.2(4)
C22	2034.3(4)	5000	5326.0(19)	24.2(4)
C23	1768.1(4)	5000	5685.3(19)	24.9(4)
C24	1555.2(4)	5000	4771(2)	26.4(4)

Table S2. Fractional Atomic Coordinates (×10[4]) and Equivalent Isotropic Displacement Parameters (Å[2]×10[3]) for **5d.** U_{eq} is defined as 1/3 of the trace of the orthogonalised U_{IJ} tensor.

**Atom**	***x***	***y***	***z***	**U(eq)**
C25	1607.2(5)	5000	3529(2)	31.7(5)
C26	1871.2(5)	5000	3167(2)	33.6(5)
C27	1212.6(4)	5000	6718.0(19)	26.5(4)
C28	924.7(4)	5000	7168.9(18)	23.0(4)
C29	954.2(4)	5000	8577.6(18)	26.2(4)
C30	672.0(4)	5000	9085.2(18)	26.9(4)
C31	514.0(3)	3154(2)	8641.2(13)	28.4(3)
C32	479.7(3)	3155(2)	7239.2(13)	26.2(3)
C33	762.8(3)	3156(2)	6737.5(13)	26.2(3)
C34	322.1(4)	5000	6794.9(19)	27.4(4)

Table S3. Anisotropic Displacement Parameters (Å[2]×10[3]) for **5d**. The Anisotropic displacement factor exponent takes the form: $-2\pi^2[h^2a^{*2}U_{11}+2hka^*b^*U_{12}+\ldots]$.

**Atom**	**U_{11}**	**U_{22}**	**U_{33}**	**U_{23}**	**U_{13}**	**U_{12}**
S1	21.9(3)	100.2(6)	21.2(3)	0	4.5(2)	0
O1	21.1(7)	48.5(9)	26.7(8)	0	5.5(6)	0
O2	24.8(8)	55.3(10)	24.9(8)	0	5.9(6)	0
N1	21.8(9)	49.3(12)	22.2(9)	0	4.9(7)	0
N2	22.5(9)	46.4(11)	21.7(9)	0	5.4(7)	0
N3	21.6(8)	42.5(10)	23.5(9)	0	5.1(7)	0
C1	21.7(10)	51.0(14)	30.7(11)	0	7.4(8)	0
C2	22.3(10)	37.5(11)	26.2(10)	0	5.0(8)	0
C3	22.5(10)	38.7(11)	24.3(10)	0	4.9(8)	0
C4	23.4(10)	38.7(12)	23.1(10)	0	7.0(8)	0
C5	24.0(10)	35.0(11)	22.2(10)	0	4.4(8)	0

Table S3. Anisotropic Displacement Parameters (Å^2×10^3) for **5d**. The Anisotropic displacement factor exponent takes the form: $-2\pi^2[h^2a^{*2}U_{11}+2hka^*b^*U_{12}+\ldots]$.

**Atom**	**U_{11}**	**U_{22}**	**U_{33}**	**U_{23}**	**U_{13}**	**U_{12}**
C6	23.3(10)	43.1(12)	22.8(10)	0	6.4(8)	0
C7	22.5(10)	42.0(12)	27.2(11)	0	7.1(8)	0
C8	26.3(11)	44.5(13)	21.9(10)	0	2.9(8)	0
C9	28.5(11)	44.8(13)	20.6(10)	0	6.6(8)	0
C10	22.9(10)	41.2(12)	21.1(10)	0	3.6(8)	0
C11	21.6(9)	26.4(10)	18.6(9)	0	2.7(7)	0
C12	24.2(10)	31.0(10)	17.6(9)	0	1.1(7)	0
C13	26.7(10)	44.6(13)	18.5(9)	0	4.4(8)	0
C14	30.9(8)	47.3(10)	28.5(8)	5.3(7)	5.9(6)	-10.5(7)
C15	29.4(8)	40.5(9)	27.2(7)	-4.4(6)	2.0(6)	-11.3(7)
C16	30.4(8)	31.5(8)	24.8(7)	-5.4(6)	3.0(6)	-1.7(6)
C17	23.3(10)	61.6(16)	24.1(10)	0	-0.4(8)	0
S2	21.1(3)	44.5(3)	23.6(3)	0	3.57(19)	0
O3	22.3(7)	41.2(9)	29.5(8)	0	7.4(6)	0
O4	27.2(8)	44.1(9)	27.0(8)	0	8.4(6)	0
N4	22.2(9)	36.8(10)	24.2(9)	0	6.0(7)	0
N5	23.9(9)	39.6(10)	23.7(9)	0	7.0(7)	0
N6	21.5(8)	32.5(9)	25.2(9)	0	4.8(7)	0
C18	24.1(10)	40.8(12)	38.4(12)	0	10.7(9)	0
C19	25.4(10)	25.6(10)	28.8(10)	0	7.5(8)	0
C20	23.7(10)	27.4(10)	25.1(10)	0	5.1(8)	0
C21	25.5(10)	32.5(11)	24.4(10)	0	7.7(8)	0
C22	24.5(10)	26.1(10)	22.5(10)	0	5.4(8)	0
C23	23.3(10)	29.1(10)	22.8(9)	0	4.4(8)	0
C24	23.8(10)	28.2(10)	27.8(10)	0	5.6(8)	0
C25	27.5(11)	43.2(13)	24.4(10)	0	1.5(8)	0

Table S3. Anisotropic Displacement Parameters (Å^2×10^3) for **5d**. The Anisotropic displacement factor exponent takes the form: $-2\pi^2[h^2a^{*2}U_{11}+2hka^*b^*U_{12}+\ldots]$.

**Atom**	**U_{11}**	**U_{22}**	**U_{33}**	**U_{23}**	**U_{13}**	**U_{12}**
C26	30.1(11)	49.6(14)	21.6(10)	0	5.5(8)	0
C27	22.3(10)	32.9(11)	24.4(10)	0	2.4(8)	0
C28	20.5(9)	27.1(10)	21.8(9)	0	3.1(7)	0
C29	24.6(10)	32.8(10)	20.9(9)	0	-0.3(8)	0
C30	27.8(10)	35.6(11)	17.5(9)	0	3.3(7)	0
C31	29.2(7)	31.7(8)	25.0(7)	4.6(6)	6.1(5)	-1.6(6)
C32	24.6(7)	30.3(7)	24.2(7)	-2.3(6)	4.4(5)	-4.4(6)
C33	24.3(7)	29.1(7)	25.5(7)	-3.9(6)	4.4(5)	-1.0(6)
C34	21.0(9)	38.5(11)	22.8(10)	0	2.4(8)	0

Table S4. Bond Lengths for **5d.**

**Atom**	**Atom**	**Length/Å**	**Atom**	**Atom**	**Length/Å**
S1	C7	1.761(2)	S2	C24	1.774(2)
S1	C10	1.815(2)	S2	C27	1.821(2)
O1	C1	1.446(2)	O3	C18	1.435(3)
O1	C2	1.336(3)	O3	C19	1.338(3)
O2	C2	1.219(3)	O4	C19	1.217(3)
N1	C2	1.366(3)	N4	C19	1.366(3)
N1	C3	1.380(3)	N4	C20	1.378(3)
N2	C3	1.358(3)	N5	C20	1.357(3)
N2	C4	1.384(3)	N5	C21	1.387(3)
N3	C3	1.318(3)	N6	C20	1.319(3)
N3	C5	1.392(3)	N6	C22	1.393(3)
C4	C5	1.402(3)	C21	C22	1.404(3)
C4	C9	1.388(3)	C21	C26	1.382(3)

Table S4. Bond Lengths for **5d.**

Atom	Atom	Length/Å	Atom	Atom	Length/Å
C5	C6	1.396(3)	C22	C23	1.397(3)
C6	C7	1.386(3)	C23	C24	1.390(3)
C7	C8	1.413(3)	C24	C25	1.407(3)
C8	C9	1.384(3)	C25	C26	1.388(3)
C10	C11	1.532(3)	C27	C28	1.537(3)
C11	C12	1.535(3)	C28	C29	1.542(3)
C11	C16	1.5363(18)	C28	$C33^{1}$	1.5356(18)
C11	$C16^{1}$	1.5362(18)	C28	C33	1.5356(18)
C12	C13	1.531(3)	C29	C30	1.536(3)
C13	C14	1.530(2)	C30	C31	1.5308(19)
C13	$C14^{1}$	1.530(2)	C30	$C31^{1}$	1.5308(19)
C14	C15	1.538(2)	C31	C32	1.5348(19)
C15	C16	1.531(2)	C32	C33	1.5376(19)
C15	C17	1.528(2)	C32	C34	1.5297(19)

^{1}+X,1-Y,+Z

Table S5. Bond Angles for **5d**.

Atom	Atom	Atom	Angle/˚	Atom	Atom	Atom	Angle/˚
C7	S1	C10	104.61(10)	C24	S2	C27	103.63(10)
C2	O1	C1	114.49(17)	C19	O3	C18	115.69(18)
C2	N1	C3	122.50(19)	C19	N4	C20	123.71(19)
C3	N2	C4	106.36(18)	C20	N5	C21	106.66(18)
C3	N3	C5	104.00(18)	C20	N6	C22	104.31(18)
O1	C2	N1	110.21(19)	O3	C19	N4	109.54(18)
O2	C2	O1	124.2(2)	O4	C19	O3	125.02(19)
O2	C2	N1	125.5(2)	O4	C19	N4	125.4(2)
N2	C3	N1	122.48(19)	N5	C20	N4	123.57(19)

Table S5. Bond Angles for **5d**.

Atom	Atom	Atom	Angle/˚	Atom	Atom	Atom	Angle/˚
N3	C3	N1	123.3(2)	N6	C20	N4	122.6(2)
N3	C3	N2	114.20(19)	N6	C20	N5	113.86(19)
N2	C4	C5	105.27(19)	N5	C21	C22	105.08(18)
N2	C4	C9	131.5(2)	C26	C21	N5	132.6(2)
C9	C4	C5	123.2(2)	C26	C21	C22	122.3(2)
N3	C5	C4	110.16(18)	N6	C22	C21	110.08(18)
N3	C5	C6	129.95(19)	N6	C22	C23	129.45(19)
C6	C5	C4	119.9(2)	C23	C22	C21	120.47(19)
C7	C6	C5	117.61(19)	C24	C23	C22	117.63(19)
C6	C7	S1	125.08(17)	C23	C24	S2	123.83(17)
C6	C7	C8	121.4(2)	C23	C24	C25	120.95(19)
C8	C7	S1	113.50(17)	C25	C24	S2	115.22(16)
C9	C8	C7	121.5(2)	C26	C25	C24	121.7(2)
C8	C9	C4	116.29(19)	C21	C26	C25	116.9(2)
C11	C10	S1	109.75(14)	C28	C27	S2	112.20(14)
C10	C11	C12	108.15(15)	C27	C28	C29	107.98(16)
C10	C11	C16[1]	111.43(11)	C33	C28	C27	111.63(10)
C10	C11	C16	111.43(11)	C33[1]	C28	C27	111.63(11)
C12	C11	C16[1]	108.27(11)	C33	C28	C29	108.29(11)
C12	C11	C16	108.27(11)	C33[1]	C28	C29	108.29(11)
C16[1]	C11	C16	109.18(17)	C33	C28	C33[1]	108.91(16)
C13	C12	C11	110.22(16)	C30	C29	C28	110.45(16)
C14	C13	C12	109.91(12)	C31	C30	C29	109.53(11)
C14[1]	C13	C12	109.91(12)	C31[1]	C30	C29	109.53(11)
C14[1]	C13	C14	109.12(19)	C31	C30	C31[1]	109.56(17)
C13	C14	C15	109.22(14)	C30	C31	C32	109.33(13)
C16	C15	C14	109.05(12)	C31	C32	C33	109.27(12)

Table S5. Bond Angles for **5d**.

Atom	Atom	Atom	Angle/˚	Atom	Atom	Atom	Angle/˚
C17	C15	C14	109.57(15)	C34	C32	C31	109.38(13)
C17	C15	C16	109.47(14)	C34	C32	C33	109.57(13)
C15	C16	C11	110.62(13)	C28	C33	C32	110.58(12)
C15	C17	C15[1]	109.68(18)	C32	C34	C32[1]	109.64(16)

[1]+X,1-Y,+Z

Table S6. Hydrogen Bonds for **5d**.

D	H	A	d(D-H)/Å	d(H-A)/Å	d(D-A)/Å	D-H-A/°
N1	H1	N6	0.84(3)	2.01(3)	2.849(3)	175(3)
N2	H2	O2	0.90(4)	2.06(3)	2.644(2)	121(3)
N4	H4	N3	0.84(3)	2.06(3)	2.892(3)	175(3)

Table S7. Torsion Angles for **5d**.

A	B	C	D	Angle/˚	A	B	C	D	Angle/˚
S1	C7	C8	C9	180.000(1)	S2	C24	C25	C26	180.000(0)
S1	C10	C11	C12	180.000(0)	S2	C27	C28	C29	180.000(0)
S1	C10	C11	C16	-61.12(11)	S2	C27	C28	C33[1]	-61.08(12)
S1	C10	C11	C16[1]	61.12(11)	S2	C27	C28	C33	61.08(12)
N2	C4	C5	N3	0.000(0)	N5	C21	C22	N6	0.000(0)
N2	C4	C5	C6	180.000(0)	N5	C21	C22	C23	180.000(0)
N2	C4	C9	C8	180.000(1)	N5	C21	C26	C25	180.000(0)
N3	C5	C6	C7	180.000(1)	N6	C22	C23	C24	180.000(0)
C1	O1	C2	O2	0.000(1)	C18	O3	C19	O4	0.000(0)
C1	O1	C2	N1	180.000(0)	C18	O3	C19	N4	180.000(0)
C2	N1	C3	N2	0.000(1)	C19	N4	C20	N5	0.000(0)
C2	N1	C3	N3	180.000(0)	C19	N4	C20	N6	180.000(0)

Table S7. Torsion Angles for **5d**.

A	B	C	D	Angle/˚	A	B	C	D	Angle/˚
C3	N1	C2	O1	180.000(0)	C20	N4	C19	O3	180.000(0)
C3	N1	C2	O2	0.000(1)	C20	N4	C19	O4	0.000(0)
C3	N2	C4	C5	0.000(0)	C20	N5	C21	C22	0.000(0)
C3	N2	C4	C9	180.000(1)	C20	N5	C21	C26	180.000(0)
C3	N3	C5	C4	0.000(0)	C20	N6	C22	C21	0.000(0)
C3	N3	C5	C6	180.000(1)	C20	N6	C22	C23	180.000(0)
C4	N2	C3	N1	180.000(0)	C21	N5	C20	N4	180.000(0)
C4	N2	C3	N3	0.000(0)	C21	N5	C20	N6	0.000(0)
C4	C5	C6	C7	0.000(1)	C21	C22	C23	C24	0.000(0)
C5	N3	C3	N1	180.000(0)	C22	N6	C20	N4	180.000(0)
C5	N3	C3	N2	0.000(0)	C22	N6	C20	N5	0.000(0)
C5	C4	C9	C8	0.000(1)	C22	C21	C26	C25	0.000(0)
C5	C6	C7	S1	180.000(0)	C22	C23	C24	S2	180.000(0)
C5	C6	C7	C8	0.000(1)	C22	C23	C24	C25	0.000(0)
C6	C7	C8	C9	0.000(1)	C23	C24	C25	C26	0.000(0)
C7	S1	C10	C11	180.000(0)	C24	S2	C27	C28	180.000(0)
C7	C8	C9	C4	0.000(1)	C24	C25	C26	C21	0.000(0)
C9	C4	C5	N3	180.000(1)	C26	C21	C22	N6	180.000(0)
C9	C4	C5	C6	0.000(1)	C26	C21	C22	C23	0.000(0)
C10	S1	C7	C6	0.000(0)	C27	S2	C24	C23	0.000(0)
C10	S1	C7	C8	180.000(0)	C27	S2	C24	C25	180.000(0)
C10	C11	C12	C13	180.000(0)	C27	C28	C29	C30	180.000(0)
C10	C11	C16	C15	-178.65(13)	C27	C28	C33	C32	177.93(13)
C11	C12	C13	C14	-60.05(12)	C28	C29	C30	C31[1]	-60.09(11)
C11	C12	C13	C14[1]	60.05(12)	C28	C29	C30	C31	60.09(11)
C12	C11	C16	C15	-59.83(16)	C29	C28	C33	C32	59.19(16)
C12	C13	C14	C15	59.70(18)	C29	C30	C31	C32	-59.97(17)

Table S7. Torsion Angles for **5d**.

A	B	C	D	Angle/˚	A	B	C	D	Angle/˚
C13	C14	C15	C16	-59.63(18)	C30	C31	C32	C33	59.94(16)
C13	C14	C15	C17	60.18(18)	C30	C31	C32	C34	-59.99(16)
C14[1]	C13	C14	C15	-60.9(2)	C31[1]	C30	C31	C32	60.19(19)
C14	C15	C16	C11	60.56(17)	C31	C32	C33	C28	-60.34(16)
C14	C15	C17	C15[1]	-59.5(2)	C31	C32	C34	C32[1]	60.10(19)
C16	C11	C12	C13	59.12(11)	C33[1]	C28	C29	C30	58.97(11)
C16[1]	C11	C12	C13	-59.13(11)	C33	C28	C29	C30	-58.97(11)
C16[1]	C11	C16	C15	57.83(19)	C33[1]	C28	C33	C32	-58.36(19)
C16	C15	C17	C15[1]	60.1(2)	C33	C32	C34	C32[1]	-59.65(19)
C17	C15	C16	C11	-59.31(17)	C34	C32	C33	C28	59.48(16)

[1]+X,1-Y,+Z

Table S8. Hydrogen Atom Coordinates (Å×10^4) and Isotropic Displacement Parameters (Å^2×10^3) for **5d**.

Atom	*x*	*y*	*z*	U(eq)
H1	2336(7)	5000	7870(30)	48(9)
H2	2641(7)	5000	10830(30)	53(9)
H1A	1533.42	5000	9150.56	51
H1B	1719.81	3818.99	10167.57	51
H1C	1719.81	6181.01	10167.57	51
H6	3394.85	5000	7829.15	35
H8	3641.95	5000	11465.96	37
H9	3187.96	5000	11990.02	37
H10A	3839.1	3816.38	7413.84	34
H10B	3839.1	6183.62	7413.84	34
H12A	4146.09	3816.84	5746.26	29

Table S8. Hydrogen Atom Coordinates (Å×10^4) and Isotropic Displacement Parameters (Å^2×10^3) for **5d**.

Atom	*x*	*y*	*z*	U(eq)
H12B	4146.09	6183.16	5746.26	29
H13	4540.57	4999.98	4851.76	36
H14A	4594.13	8040.74	5936.37	42
H14B	4876.41	6840.87	6064.98	42
H15	4773.55	8049.24	8029.56	39
H16A	4288.37	8043.8	7660.49	35
H16B	4375.75	6883.63	8903.09	35
H17A	4818.9	4999.99	9116.48	44
H17B	5015.42	5000.01	8019.19	44
H4	2783(6)	5000	6340(30)	38(8)
H5	2469(7)	5000	3380(30)	52(9)
H18A	3585.94	5000	5188.05	51
H18B	3408.58	3818.99	4140.45	51
H18C	3408.58	6181.01	4140.45	51
H23	1733.8	5000	6524.49	30
H25	1457.84	5000	2923.41	38
H26	1905.79	5000	2327.64	40
H27A	1312.69	3818.76	7041.27	32
H27B	1312.69	6181.24	7041.27	32
H29A	1057.52	3817.06	8874.84	31
H29B	1057.52	6182.94	8874.85	31
H30	694.79	5000.01	9998.57	32
H31A	332.54	3141.93	8972.3	34
H31B	614.33	1956.89	8935.54	34
H32	376.66	1949.81	6946.01	31
H33A	864.27	1957.86	7021.89	31
H33B	741.56	3128.64	5833.21	31

Table S8. Hydrogen Atom Coordinates (Å×10^4) and Isotropic Displacement Parameters (Å^2×10^3) for **5d**.

Atom	*x*	*y*	*z*	U(eq)
H34A	139.38	5000.07	7113.56	33
H34B	297.32	5000	5890.99	33

Table S9. Atomic Occupancy for **5d**.

Atom	*Occupancy*	Atom	*Occupancy*	Atom	*Occupancy*
H1B	0.5	H1C	0.5	H10A	0.5
H10B	0.5	H12A	0.5	H12B	0.5
H18B	0.5	H18C	0.5	H27A	0.5
H27B	0.5	H29A	0.5	H29B	0.5

Crystal data

$C_{20}H_{25}N_3O_2S$ (M = 371.49 g/mol): monoclinic, space group C2/m (no. 12), a = 49.1582(6) Å, b = 6.77525(8) Å, c = 10.97866(11) Å, β = 94.6368(11)°, V = 3644.58(7) Å^3, Z = 8, T = 160.0(1) K, μ(Cu Kα) = 1.737 mm^{-1}, *Dcalc* = 1.354 g/cm^3, 21560 reflections measured (7.216° ≤ 2Θ ≤ 158.978°), 4233 unique (R_{int} = 0.0216, R_{sigma} = 0.0168) which were used in all calculations. The final R_1 was 0.0460 (I > 2σ(I)) and wR_2 was 0.1421 (all data).

Refinement model description

Number of restraints - 0, number of constraints - unknown.

Details:

1. Fixed Uiso

At 1.2 times of:

All C(H) groups, All C(H,H) groups

At 1.5 times of:

All C(H,H,H) groups

2. Others

Fixed Sof: H1B(0.5) H1C(0.5) H10A(0.5) H10B(0.5) H12A(0.5) H12B(0.5) H18B(0.5) H18C(0.5) H27A(0.5) H27B(0.5) H29A(0.5) H29B(0.5)

3.a Ternary CH refined with riding coordinates:

C13(H13), C15(H15), C30(H30), C32(H32)

3.b Secondary CH2 refined with riding coordinates:

C10(H10A,H10B), C12(H12A,H12B), C14(H14A,H14B), C16(H16A,H16B), C17(H17A, H17B), C27(H27A,H27B), C29(H29A,H29B), C31(H31A,H31B), C33(H33A,H33B), C34(H34A,H34B)

3.c Me refined with riding coordinates:

C1(H1A,H1B,H1C), C18(H18A,H18B,H18C)

3.d Aromatic/amide H refined with riding coordinates:

C6(H6), C8(H8), C9(H9), C23(H23), C25(H25), C26(H26)

1.9. Molecular docking

Molecular docking was performed with AutoDock4.2[17–19] and AutoDockTools (ADT).[19] The docking procedure was adapted from previously reported tutorials.[20]

The structure of the protein was taken from a previously developed homology model of *H. contortus* β-tubulin isotype β12-16 homology docked with albendazole sulfoxide by Robinson and coworkers (Model Archive ID: ma-cso9w). Albendazole sulfoxide was removed with PyMOL and the resulting protein model was saved as a .PDB file.

Models of the organic and organometallic derivatives were built using Avogadro2 and previously reported fragments (ferrocene, CCDC: 960880; albendazole, CCDC: 1828463),[21,22] non polar hydrogens were removed and geometry optimization was performed in Avogadro2 using MMFF94 force field and the geometry of the ferrocenyl fragment was frozen to avoid highly distorted structures. The resulting .PDB files were converted to .PDBQT files with ADT.

ADT was used to prepare the protein for docking, polar hydrogens were added as well as Kollman charges. The ligand was loaded in ADT, the root of the torsion tree detected, and the rotatable / non-rotatable bonds were checked manually for each ligand. Grid was prepared with the previously prepared enzyme as the macromolecule, the map type was set to the ligand. The grid box parameters were chosen to fit the pocket of the enzyme (Center of the grid box: x, 7 Å; y, -8 Å; z, -1 Å. Number of points: x, 50; y, 44; z, 44) and the grid was generated with AutoGrid in ADT. Docking parameters were set in ADT using the prepared enzyme as a macromolecule and the prepared ligand. Docking was performed with a Lamarckian Genetic Algorithm (Number of GA runs: 50; Population size: 150; Maximum number of evals: 2500000; Maximum number of generation: 27000).

Validation of the docking protocol was performed with albendazole sulfoxide extracted from the model. The docking resulted in a binding eergy of -9.43 kcal/mol and RMSD of 1.49 Å.

Outcome of the docking calculation was analysed using ADT. The binding poses were selected as the lowest energy conformation in the lowest energy cluster and the conformation was saved as a .PDB file. Figures were prepared using PyMOL.

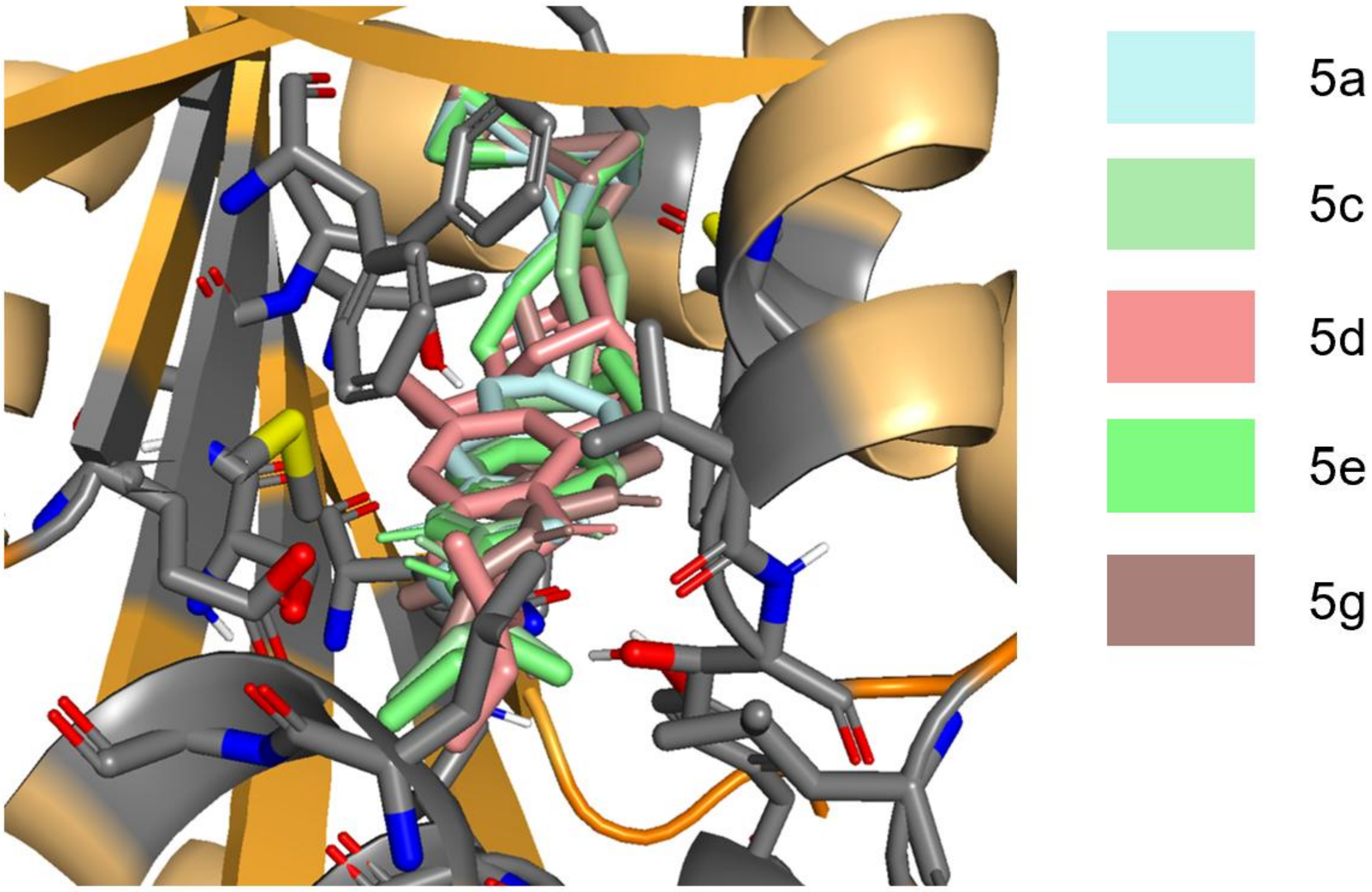

Figure S125. Overlap of the docking poses of compounds **5a**, **5c**, **5d**, **5e** and **5g**.

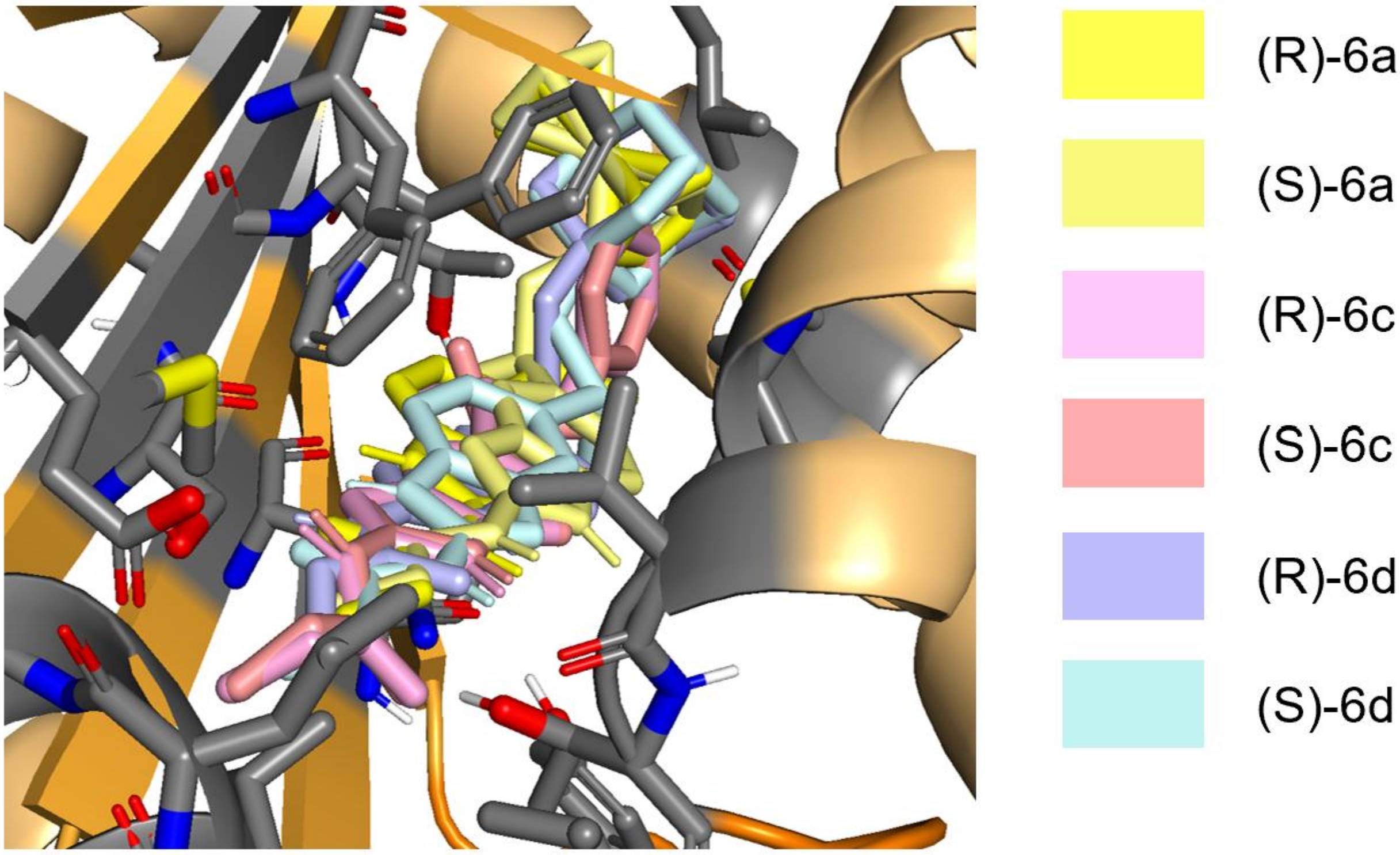

Figure S126. Overlap of the docking poses of compounds **6a**, **6c** and **6d**.

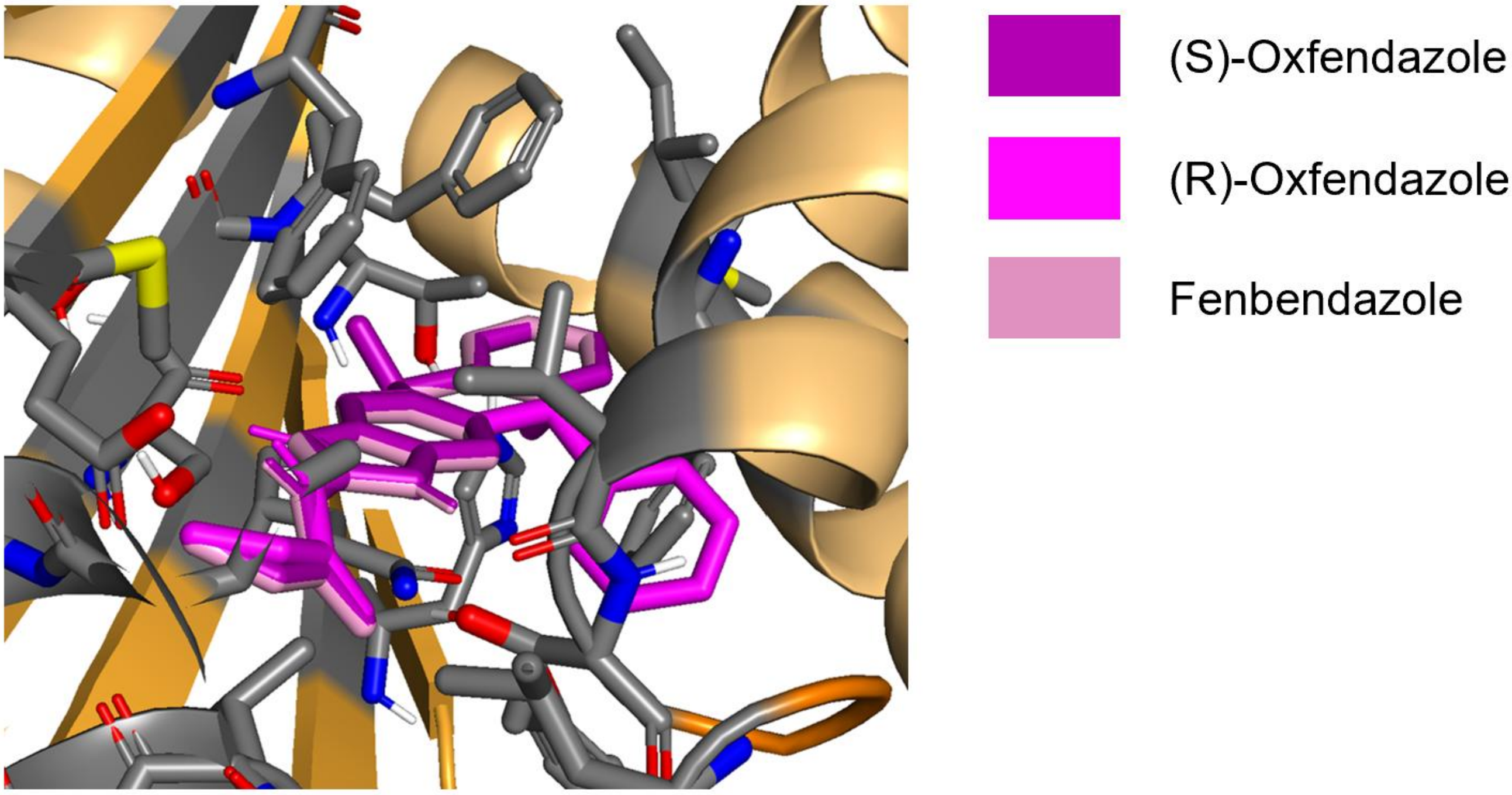

Figure S127. Overlap of the docking poses of oxfendazole and fenbendazole.

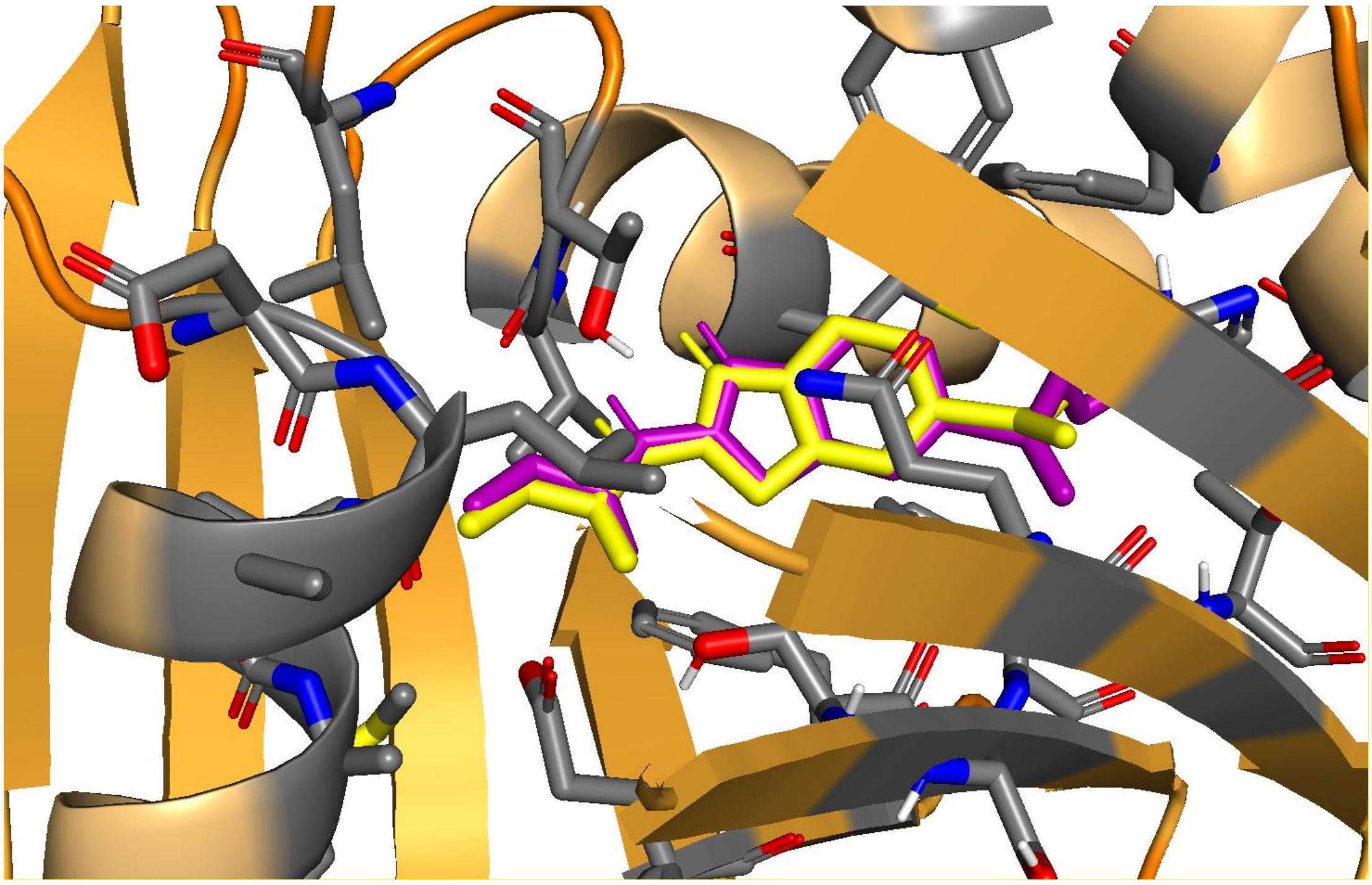

Figure S128. Overlap of the docking poses of albendazole sulfoxide before (yellow) and after (purple) docking.

2. Experimental section in biology

2.1. Production and preparation of *C. elegans* and *H. contortus* larvae for bioassays

C. elegans (N2 – wildtype Bristol strain) was cultured under standard laboratory conditions at 20 °C on nematode growth media (NGM) agar plates, seeded with *Escherichia coli* OP50 as a food source.[11] Gravid adult worms were harvested from NGM plates, washed with sterile M9 buffer and then treated with a sterile 0.4 % (*v*/*v*) NaClO with 170 mM sodium hydroxide for 4–8 min at 22–24 °C (room temperature) to release eggs, with intermittent shaking.[11,12] Eggs were immediately collected and washed five times in 15 mL of sterile M9 buffer (centrifugation at 500 × *g*, 2 min). After washing, the egg pellet was suspended in 8 mL of M9 buffer in a 15 mL tube and gently agitated for 24 h at 22–24 °C, allowing first-stage larvae (L1s) to hatch and enter diapause. Approximately 48 h prior to screening, synchronised *C. elegans* L1s were transferred on to NGM plates containing 500 µL of *E. coli* OP50 (~ 3,000 larvae per plate) and allowed to develop to fourth-stage larvae (L4s) at 20 °C. On the day of the assay, L4s were collected from plates and washed twice with sterile M9 buffer (centrifugation at 500 x *g*, 2 min) to remove *E. coli* OP50, and then resuspended to a density of 100 larvae per 50 µL in sterile (autoclaved) lysogeny broth (LB), supplemented with final concentrations of 100 IU/mL of penicillin, 100 µg/mL of streptomycin and 0.25 µg/ml of amphotericin B (Fungizone®, cat. No. 15240-062, Gibco, Thermo Fisher Scientific, Waltham, MA, USA) – designated LB*.[13]

H. contortus (Haecon-5 strain) was maintained in experimental sheep according to established protocols[14,15] in compliance with institutional animal ethics guidelines (permit no. 23983; University of Melbourne) and Australian regulations. Helminth-free Merino sheep (six months old; male) were orally inoculated with 7,000 third-stage larvae (L3s). Faecal samples, containing *H. contortus* eggs, were collected daily from sheep with patent infection, commencing four weeks post-infection. These eggs were incubated at 27 °C and > 90% relative humidity for seven days to develop into L3 larvae. The developed larvae were then collected, suspended in tap water, and filtered through two layers of nylon mesh (pore size: 20 µm; Sefar, Australia) to eliminate debris or dead larvae. Filtered larvae were stored in sterile 0.9% NaCl solution (at a density of 2,000 L3s per mL) at 11 °C. Immediately prior to use, L3s were exsheathed and sterilised by incubation in 0.15% (*v/v*) NaClO at 38 °C for 20 min with gentle shaking. Post-treatment, exsheathed L3s (xL3s) were immediately washed five times in 50 mL of sterile

0.9% NaCl solution (centrifugation at 800 x *g*, 5 min) at room temperature (22–24 °C); following the final wash, xL3s were resuspended to a density of 300 larvae per 50 µL in sterile LB*.[14,15]

2.2. Bioassay for the evaluation of anthelmintic activity using larvae of *C. elegans* or *H. contortus*

The dose-response assay for *C. elegans* L4s and *H. contorus* xL3s followed well-established protocols.[13-15] Test compounds were assessed individually (9-point, 2-fold serial dilution in LB*; from 50 µM to 0.19 µM), with reference to the four positive-control compounds – monepantel (Zolvix™; Elanco, Australia) and moxidectin (Cydectin®; Virbac, France) were used as assay controls, and oxfendazole and fenbendazole were used as benzimidazole-derivative controls. A solution of LB* + 0.25 % (*v*/*v*) dimethylsulfoxide (DMSO) was used as a negative control. *C. elegans* L4s and *H. contorus* xL3s were in flat-bottom 96-well microplates (cat. no. 3596; Corning, Corning, NY, USA), at a density of 100 *C. elegans* L4s or 300 *H. contorus* xL3s in a final volume of 100 µL with the prepared compound solutions. Plates were incubated for 40 h at 20 ˚C (*C. elegans*) or 168 h at 38 °C, 10% (*v*/*v*) CO_2 and > 90% relative humidity (*H. contortus*). At the end of each incubation periods, worm motility was measured using a WMicroTracker ONE unit (Phylumtech, Sunchales, Santa Fe, Argentina). For *H. contortus*, the development and phenotypic alterations of xL3s was also assessed.[14] Compound concentrations were log_{10}-transformed and normalised motility response were fitted using nonlinear regression with a constrained four-parameter variable-slope concentration-response model in GraphPad Prism (v10.2.2; GraphPad Software, San Diego, CA, USA), with the top parameter fixed at 100%. IC_{50} values were estimated from at least three independent experiments. Statistical differences in larval motility among treatment groups were analysed using one-way ANOVA followed by Tukey's multiple comparison test. Differences were considered statistically significant at $P < 0.05$.

2.3. Ethics for NTS, S. mansoni, T. muris, H. polygyrus, N. americanus, A. ceylanicum and S. ratti.

The below described *in vitro* studies with rodents involved, were conducted at the Swiss Tropical and Public Health Institute (Swiss TPH) and had the approval by the corresponding veterinary authorities of the Canton Basel-Landschaft (permission no. 545) based on Swiss cantonal and national regulations. Rodents were kept the entire time in IVC Cages "Tecniplast" with soft wood chip bedding, mouse house,

chewing wood and household paper. The climate for the animals is provided by SCANCLIME machines. Temperature: 22°C +/- 2; Humidity 50% +/- 10%. Artificial lighting is used with a circadian cycle of 12 hours of light (6 a.m. - 6 p.m.). An automatic standby power supply will be brought into operation should the main supply fail.

2.4. *In vitro* evaluation on newly transformed schistosomula (NTS)

To obtain newly transformed schistosomula (NTS) the lifecycle of *S. mansoni* (strain originates from West Africa, Liberia) is maintained at the Swiss TPH. As previously described, cercariae are collected from infected *Biomphalaria glabrata* snails and then mechanically transformed into NTS.[16] The drug assays were performed the next day in medium M199 (Gibco, Waltham MA, USA) supplemented with 5 % v/v horse serum (Gibco, Waltham, MA, USA), 1 % Mäser antibiotics mixture, and 1 % penicillin 10,000 U/mL, and streptomycin 10 mg/mL solution (Sigma-Aldrich, Buchs, Switzerland). The assay was performed in a 96-well plate, with 30−40 NTS per well in a final volume of 250 µL. NTS incubated with no more than 1 % DMSO served as control. Microscopic evaluation was performed after 72 h of incubation of the parasites at 37 °C and 5 % CO_2. Their overall viability was scored from 0 (death) to 3 (full viability) by a phenotypic screening method based on their motility, morphology, and granularity. All 10 compounds as well as oxfendazole and fenbendazole were initially tested at 10 µM in triplicate on NTS and repeated once, identified hits with a mean drug effect of ≥ 60 % at 10 µM were further evaluated at 1 µM and on adult worms.

2.5. *In vitro* evaluation on adult *S. mansoni*

Four-week-old female NMRI mice (Charles River, Sulzfeld, Germany) were subcutaneously infected with approximately 100 cercariae to establish the *S. mansoni* infection. Worms were allowed to develop for 7 weeks into the adult stage, before they were extracted from the mesenteric veins of the euthanized mice. The adult worms were placed into RPMI 1640 (Gibco, Waltham MA, USA) medium supplemented with 1 % penicillin/streptomycin mixture and 5 % horse serum at 37 °C and 5 % CO_2. All 10 compounds and oxfendazole and fenbendazole were initially tested twice at 10 µM and the drug sensitivity assay was set up in a 24-well plate with two wells containing each three pairs of *S. mansoni* worms per

compound and concentration and repeated once. The final volume per well was 2 mL, containing the culture medium and the corresponding amount of drug dissolved in DMSO. Additionally, 2 wells with negative controls containing only 1 % DMSO and the culture medium were included. After an incubation period of 72 h at 37 °C and 5 % CO_2, the worms were microscopically evaluated. The drug effect was determined 72 h post-exposure based on the phenotype of the adult worms, using a viability scale ranging from three referring to viable, motile worms with no change in their tegument to zero standing for the death of the worms. Active compounds (mean drug effect of ≥ 60 %) at 10 µM were further evaluated at 1 µM.

2.6. *In vitro* evaluation on adult *T. muris*

The life cycle of *T. muris* is established and maintained at Swiss TPH. four-week-old female C57BL/6NRj mice (Janvier labs, Le Genest-Saint-Isle, France) were infected *per os* with 200–250 embryonated *T. muris* eggs. Dexamethasone (Sigma-Aldrich, Buchs, Switzerland) (1 mg/L) was added to the drinking water until 2 days before treatment to facilitate a sustainable infection. To obtain the adult worms for the *in vitro* studies, the infected mice were sacrificed 41 days after infection with CO_2, and their intestines were dissected. For the *in vitro* evaluation, 2 to 3 adult worms were incubated in 24-well plates for 72 h at 37 °C and 5 % CO_2 with the compound in a final volume of 2.5 mL. The adult worms were placed into RPMI 1640 (Gibco, Waltham MA, USA) medium supplemented with 1 % penicillin/streptomycin mixture and 5 % horse serum at 37 °C and 5 % CO_2. All 10 compounds and oxfendazole and fenbendazole were initially tested twice at 10 µM and repeated once. The drug effect was determined 72 h post-exposure based on the phenotype of the adult worms, using a viability scale ranging from three referring to viable, motile worms with no change in their tegument to zero standing for the death of the worms. Wells containing 1 % DMSO corresponding to the drug concentration tested, were included in every test and served as negative controls.

2.7. *In vitro* evaluation on *H. polygyrus* (L3)

Two weeks post infection the feces from the *H. polygyrus* infected mice were collected and the eggs were extracted by filtration and centrifugation. The *H. polygyrus* eggs were washed and then kept in darkness on agar plates at room temperature for 8 to 10 days, so the larvae can hatch and develop into

the third stage. For the *in vitro* assays with third stage *H. polygyrus* larvae, 30-40 L3 were placed in a total volume of 200-250 µL, containing 100 µM of the compound in RPMI 1640 medium supplemented with 5 % amphotericin B (250 µg/mL), 1 % penicillin (10,000 U/mL) and streptomycin (10 mg/mL) solution. All 10 compounds and oxfendazole and fenbendazole were initially tested in triplicates and repeated once at 10 µM. Wells containing 1 % DMSO served as negative controls and were included in every assay. *H. polygyrus* were kept at room temperature in a dark box, up to 72 h. For evaluating the L3 assays, the larvae were stimulated, if necessary, by the addition of 80 µL hot water (≈ 80 °C) and the percentage of survival was determined by the ratio of moving larvae to the total number of larvae present in the well.

2.8. In vitro evaluation on N. americanus (L3) and A. ceylanicum L3

Hamsters were infected subcutaneously with 150 *N. americanus* L3 and 140 *A. ceylanicum* L3. The feces of infected hamsters were filtered to obtain hookworm eggs which were then cultivated on an agar plate for 8–10 days in the dark at room temperature to obtain larvae (L3). 30-40 L3 were incubated in Hanks' balanced salt solution (HBSS; Gibco, Waltham MA, USA) supplemented with 10 % amphotericin B and 1 % penicillin (10,000 U/mL) and streptomycin (10 mg/mL) solution in 96 well plates. All 10 compounds and oxfendazole and fenbendazole were initially tested in triplicates and repeated once at 10 µM. *N. americanus* L3 and *Ancylostoma* L3 were kept in the dark at room temperature after which the effect of the drugs was evaluated. For this, the total number of L3 per well was determined. Then, as the wells were stimulated by the addition of 80 µL hot water (≈ 80 °C) and if needed by vigorous up and down pipetting and the larvae that responded (the moving worms) were counted. The proportion of larval death was determined, and the percentage of survival was determined by the ratio of moving larvae to the total number of larvae present in the well.

2.9. *In vitro* evaluation on *S. ratti* (L3)

Strongyloides ratti L3 were obtained from infected rats by the cultivation of Baermann method, gained by repeated filtration and centrifugation of the infected feces. 30-40 L3 larvae were placed in each well of a 96-well plate with culture medium (phosphate-buffered saline (PBS) supplemented with 1 %

penicillin (10,000 U/ml) solution) and the test compound (10 µM) for a final well volume of 200-250 µl. All 10 compounds and oxfendazole and fenbendazole were initially tested in triplicates and repeated once at 10 µM. Larvae incubation with no more than 1 % DMSO served as control. *Strongyloides ratti* were kept at room temperature in a dark box, up to 72h. For evaluating the L3 assay, the larvae were stimulated, if necessary, by the addition of 80 µL hot water (≈80 °C) and the percentage of survival was determined by the ratio of moving larvae to the total number of larvae present in the well.

2.10. Ethics, strain maintenance and culture of *E. multilocularis*

The *E. multilocularis* strain H95 was maintained in female BALB/c mice (Charles River Laboratories, Sulzheim, Germany) as previously described by Kaethner et al..[23] Animals were housed under standardized environmental conditions comprising a 12 h light/dark cycle, a temperature of 21–23 °C, and a relative humidity of 45–55 %. Food and water were provided *ad libitum*. Cages were enriched with nesting materials (Plexx, Elst, Netherlands), mouse shelters (Tecniplast, Gams, Switzerland), and tunnels (Zoonlab, Castrop-Rauxel, Germany) to promote natural behavior. All experimental procedures were approved by the Animal Welfare Committee of the Canton of Bern (license numbers BE2/2022 and BE47/2025) and conducted in accordance with the Swiss Federal Act on Animal Protection (TSchV, SR455). *In vitro* culture of *E. multilocularis* was performed as described by Kaethner et al..[23]

2.11. *In vitro* evaluation on *E. multilocularis* germinal layer cells

Germinal layer cells were isolated as described by Kaethner et al., 2023.[23] Conditioned medium (cDMEM) was prepared by incubating 1×10^6 and 10×10^6 Rat Hepatoma cells (H-4-II-E) in DMEM supplemented with 10 % FBS, penicillin (100 U/mL), streptomycin (100 µg/mL), and tetracycline (5 µg/mL) for six and four days, respectively, at 37 °C with 5 % CO_2. The supernatants were combined (1:1) and sterile-filtered. *E. multilocularis* metacestode vesicles (> 6 months old) were washed with PBS, exposed to distilled H_2O for 2 min, and mechanically disrupted. The resulting pellet was resuspended in seven volumes of pre-warmed 0.05 % trypsin/0.02 % EDTA and incubated for 30 min at 37 °C. Germinal layer cells were released by vigorous shaking, passed through a 30 µm nylon mesh (Sefar AG, Heiden,

Switzerland), and separated from calcareous corpuscles by centrifugation at 50 × g for 30 sec at room temperature. Cells were pelleted by centrifugation at 600 × g for 10 min and resuspended in cDMEM. A 1:100 dilution of the suspension in PBS was used to determine OD_{600}. An OD_{600} of 0.1 was defined as 1 arbitrary unit (AU) per µL of the undiluted suspension, corresponding to 0.97 ± 0.11 µg total protein as quantified by the Pierce™ BCA Protein Assay Kit (Fisher Scientific AG, Reinach, Switzerland). Germinal layer cells (1,000 AU per 5 mL cDMEM) were cultured overnight at 37 °C under humidified nitrogen. The following day, 2,000 AU were pooled and further incubated for 3 h under identical conditions prior to downstream applications.

Viability of isolated GL cells was evaluated as detailed by seeding 15 AU of germinal layer cells per well in black 384-well plates. Compounds were added at three different concentrations each, tested in four technical replicates each: **5a**, **6a**, **5b**, **5c**, **6c**, **5d**, **6d**, **5e**, **5f**, **5g** at 50 µM, 20µM, 10µM , and oxfendazole and fenbendazole at 10 µM, 1 µM, 0.1 µM tested in technical duplicates. Cells were cultured for 5 days at 37 °C under microaerobic conditions (85 % N_2, 10 % CO_2, 5 % O_2). DMSO at equivalent concentrations served as solvent control. Viability was assessed using the CellTiter-Glo® assay, and luminescence was recorded with a HIDEX Sense microplate reader. Results are expressed as mean relative viabilities compared to DMSO controls (100%), and SDs are given.

2.12. *In vitro* evaluation on *E. multilocularis* metacestode vesicles

To assess drug-induced damage in intact metacestode vesicles, the phosphoglucose isomerase release assay was performed as previously described (Stadelmann et al., 2010),[24] with minor modifications. Metacestode vesicles aged 8–12 weeks were purified using 2 % sucrose and washed in PBS. The purified vesicles were suspended in two volumes of phenol red-free DMEM supplemented with penicillin (100 U/mL) and streptomycin (100 µg/mL). Aliquots of 1 mL were transferred into 48-well plates (Huberlab, Aesch, Switzerland), each well containing 20-30 metacestode vesicles. Compounds **5a**, **6a**, **5b**, **5c**, **6c**, **5d**, **6d**, **5e**, **5f**, **5g** were added at 50 µM, 20 µM, and 10 µM, whereas oxfendazole and fenbendazole were added at final concentrations of 10 µM, 1 µM, and 0.1 µM, all in technical triplicates and the assays were performed two times independently. Plates were incubated at 37 °C in a humidified atmosphere containing 5 % CO_2. Supernatants were collected after five and twelve days, and vesicle morphology was documented with a Nikon SMZ18 stereo microscope (Nikon, Basel, Switzerland) at 7.5× magnification. Phosphoglucose activity in supernatants was measured using a HIDEX Sense microplate

reader (Hidex, Turku, Finland). Data was normalized to the Triton X-100 control (100%) after subtraction of DMSO background values. Mean values and corresponding SDs of biological duplicates were calculated.

2.13. *L. infantum* parasites

Leishmania infantum promastigotes (MHOM/MA(BE)/67/ITMAP2603) were maintained at 28°C in BHI-Tryptose medium supplemented with 10% heat-inactivated FBS (fetal bovine serum) and streptomycin (100 $\mu g \cdot mL^{-1}$), penicillin (10 $\mu g \cdot mL^{-1}$). Cultures were subcultured every 72 h to maintain cells in the exponential growth phase.[25–27]

2.14. In vitro screening against promastigotes of L. infantum

The *in vitro* activity of the compounds on *L. infantum* extracellular forms was assessed as follows: Promastigotes (10^6 parasites/well) were seeded BHI medium (100 µL/well), in black 96-well plates, with the desired compound concentrations in culture medium (100 µL/well). The compounds were dissolved in DMSO to reach at 10 concentrations (stock solutions). Working solutions with the different tested concentrations were prepared by diluting the stock solutions, fixing the concentration of DMSO at 1%, which is non-toxic for the protozoa. The plates were incubated for 22 h at 28 ^oC. Amphotericin B (0.1µM) was used as a reference drug. The parasites' viability was evaluated using resazurin, a virtually non-fluorescent, blue-colored compound that the mitochondrion of living cells reduces to resofurin which is highly fluorescent. Briefly, a solution of resazurin (2 mg/mL) was added to each well (50 µL/well) and the plates were incubated for 4 hours at 37 ^oC or 28 ^oC. A fluorescence assay (excitation 530 nm/emission 590 nm) allowed for the evaluation of the parasites' viability using a Thermo Scientific Varioskan Flash Multimode spectrofluorimeter.[25,26] Statistical analysis was performed using two-way ANOVA test. Differences relative to parent drugs were considered significant at $p<0.05$ (**5a**, **6a**, **5f**); $p<0.0001$ (**6d**).

2.15. *L. infantum* promastigotes proliferation

The *in vitro* antiproliferative activity of the compounds on *Leishmania infantum* promastigotes was assessed as follows: Parasites (10^5 parasites/well) were seeded in BHI medium (100 µL/well), in colorless 96-well plates, with increasing compound concentrations (at least 7 concentration values) in BHI medium (100 µL/well). The plates were incubated for 5 days at 28 ºC in a 5% CO_2 atmosphere. The parasites' proliferation was evaluated by measuring absorbance at 595 nm in a Thermo Scientific Varioskan Flash Multimode spectrophotometer, during the time incubation with compounds.[28,29]

2.16. Murine macrophages J774

Mouse macrophages (cell line J774, ATCC® CCL81) were cultured in Dulbecco's Modified Eagle Medium (DMEM) supplemented with 10% (v/v) FBS, and antibiotics (10 units/mL penicillin, and 10 µg/mL streptomycin) by incubation at 37 °C in a humidified atmosphere containing 5% CO_2 and 95% air. The experimental protocol for determining viability is identical to that previously derscribe method. It uses the WST-1 reagent (Roche) (2-(4-iodophenyl)-3-(4-nitrophenyl)-5-(2,4-disulfophenyl)-2H-tetrazolium sodium salt). The compounds' cytotoxicity was assessed at different concentrations. The control included cells cultured in the presence of DMSO (≤1% v/v). All samples were tested in triplicate. The absorbance at 450 nm, corresponding to the formazan dye produced by metabolically active cells, was quantified using an EL 800-microplate reader. Data analysis was performed using GraphPad Prism 8.0.1 software.[26,29]

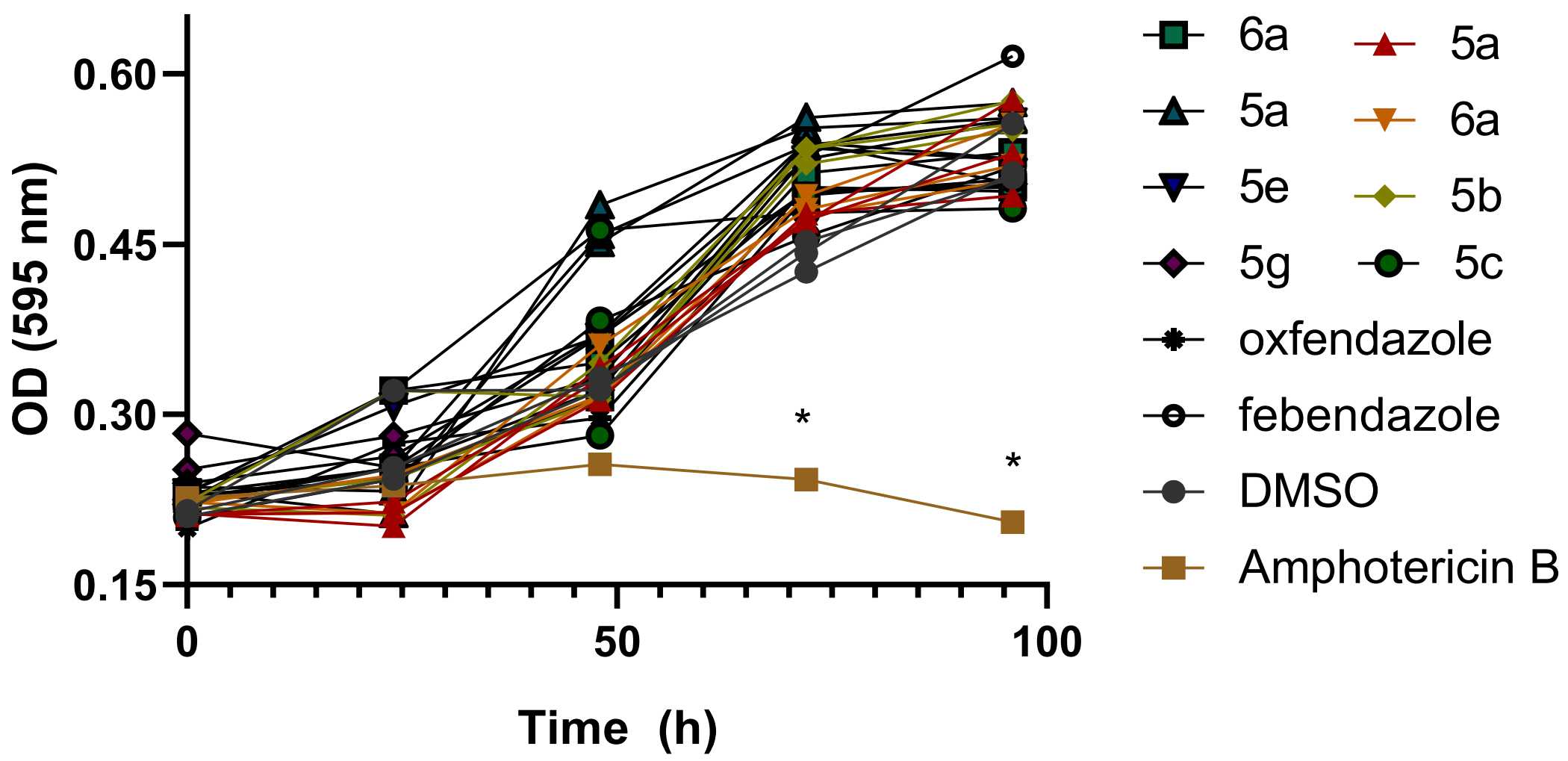

Figure S129. Growth of *L. infantum* promastigotes in the presence of benzimidazole derivatives. Parasites were incubated with the indicated compounds at 50 µM over a 5-day period. The optical density (OD) was monitored daily at 595 nm as a measure of parasite proliferation. Amphotericin (0.5 µM) was employed as a control. Data points represent the mean ± standard deviation (SD) of three independent experiments performed in triplicate. The growth curves of the inactive derivatives were indistinguishable from the negative control (DMSO 1 %), indicating the absence of leishmanostatic activity for these compounds.

2.17. RPE-1 Cells culture

Retinal pigment epithelium (RPE-1) cells (RRID: CVCL_4388) were grown using DMEM/F-12 medium supplemented with 10 % FBS and 1 % Penstrep. The cells were cultivated and maintained in a cell culture incubator at 37 °C with 5 % CO_2 and 20 % O_2 atmosphere.

2.18. Cell viability assay (RPE-1)

Cytotoxicity of the compounds was assessed by measuring the cell viability using a fluorometric resazurin assay. The cultivated cells were seeded in triplicate in 96 well plates with a density of 4000 cells per well in 100µL of media. After 24h, the medium was removed and the cells were treated with increasing concentrations of the complex diluted in cell media achieving a total volume of 100 µL at 37 °C with 5 % CO_2 and 20 % O_2 atmosphere and the cells were cultivated for 48 h. The media was changed with fresh media containing resazurin (0.2 mg/mL). After 4 h of incubation at 37 °C with 5 % CO_2 and 20 % O_2 atmosphere, the fluorescence signal of resorufin product was measured (λ_{ex}: 540 nm and λ_{em}: 590 nm)

in a BioTek®. IC_{50} values were then calculated using GraphPad Prism software. Concentrations were converted to log_{10} scale, normalization was performed with respect to empty wells (no cells) for the lowest values, nonlinear regression was performed (log(inhibitor) vs response – variable slope, four parameters). Each experiment was performed three times independently, and an average IC_{50} value (in µM) was reported with a standard deviation.

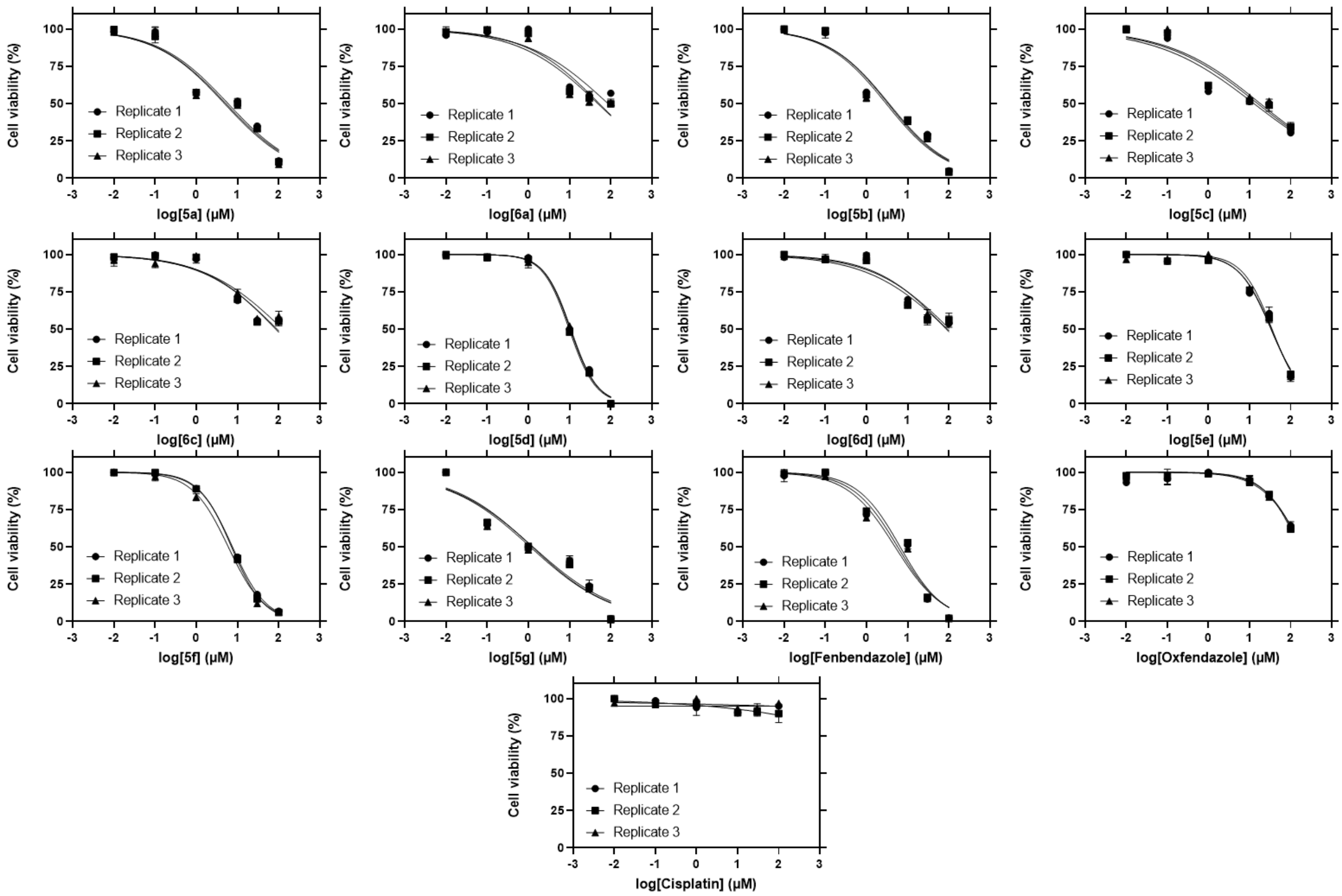

Figure S130. Cell viability curves for cytotoxicity assays of **5a-g**, **6a,c-d**, oxfendazole, fenbendazole and cisplatin toward RPE-1 cells.

2.19. Biological drawings

Image(s) provided by Servier Medical Art (https.//smart.servier.com),
licensed under CC BY 4.0 (https.//creativecommons.org/licenses/by/4.0/).

3. References

(1) Gottlieb, H. E.; Kotlyar, V.; Nudelman, A. NMR Chemical Shifts of Common Laboratory Solvents as Trace Impurities. *J. Org. Chem.* **1997**, *62* (21), 7512–7515. https://doi.org/10.1021/jo971176v.

(2) Zou, L.; Braegelman, A. S.; Webber, M. J. Spatially Defined Drug Targeting by in Situ Host–Guest Chemistry in a Living Animal. *ACS Cent. Sci.* **2019**, *5* (6), 1035–1043. https://doi.org/10.1021/acscentsci.9b00195.

(3) Lin, Y.; Scalese, G.; Bulman, C. A.; Vinck, R.; Blacque, O.; Paulino, M.; Ballesteros-Casallas, A.; Pérez Díaz, L.; Salinas, G.; Mitreva, M.; Weil, T.; Cariou, K.; Sakanari, J. A.; Gambino, D.; Gasser, G. Antifungal and Antiparasitic Activities of Metallocene-Containing Fluconazole Derivatives. *ACS Infect. Dis.* **2024**, *10* (3), 938–950. https://doi.org/10.1021/acsinfecdis.3c00577.

(4) Khrushcheva, N. S.; Sokolov, V. I. Synthesis of Versatile Intermediates of the Ferrocene Series: Reductive Amination of Ferrocenecarbaldehyde. *Russian Chemical Bulletin* **2004**, *53* (4), 830–833. https://doi.org/10.1023/B:RUCB.0000037851.47573.4b.

(5) Grossel, M. C.; Hamilton, D. G.; Fuller, J. I.; Millan-Barios, E. Alkali-Metal Binding Properties of Simple Ferrocenyl- and Ruthenocenyl-Substituted Aza-Crown Ethers. *J. Chem. Soc., Dalton Trans.* **1997**, No. 19, 3471–3477. https://doi.org/10.1039/a703597g.

(6) Johnson, J.; Murphy, E. Simplified Synthesis of Air-Stable Copper-Complexed Josiphos Ligand via Ugi's Amine: Complete Preparation and Analysis from Ferrocene. October 20, 2022. https://doi.org/10.26434/chemrxiv-2022-f3p0d.

(7) Lednicer, S. D.; Hauser, C. R.; McKusick, C. B. C.; Vest, R. D. [Iron, [(Cyanomethyl)Cyclopentadienyl]Cyclopentadienyl]. *Organic Syntheses* **1973**, *5*, 578. https://doi.org/10.15227/orgsyn.040.0045.

(8) Chatelain, G.; Meyer, A.; Morvan, F.; Vasseur, J.-J.; Chaix, C. Electrochemical Detection of Nucleic Acids Using Pentaferrocenyl Phosphoramidate α-Oligonucleotides. *New J. Chem.* **2011**, *35* (4), 893. https://doi.org/10.1039/c0nj00902d.

(9) Lewtak, J. P.; Landman, M.; Fernández, I.; Swarts, J. C. A DFT-Elucidated Comparison of the Solution-Phase and SAM Electrochemical Properties of Short-Chain Mercaptoalkylferrocenes: Synthetic and Spectroscopic Aspects, and the Structure of Fc–CH_2CH_2–S–S–CH_2CH_2–Fc. *Inorg. Chem.* **2016**, *55* (5), 2584–2596. https://doi.org/10.1021/acs.inorgchem.5b02936.

(10) Yang, Y.; Xie, Z.; Wu, C. Novel Synthesis and Characterization of Side-Chain Ferrocene-Containing Polymers. *Macromolecules* **2002**, *35* (9), 3426–3432. https://doi.org/10.1021/ma011772y.

(11) Clark, R. C.; Reid, J. S. The Analytical Calculation of Absorption in Multifaceted Crystals. *Acta Cryst A* **1995**, *51* (6), 887–897. https://doi.org/10.1107/S0108767395007367.

(12) CrysAlisPro, 2024.

(13) Dolomanov, O. V.; Bourhis, L. J.; Gildea, R. J.; Howard, J. a. K.; Puschmann, H. OLEX2: A Complete Structure Solution, Refinement and Analysis Program. *J Appl Cryst* **2009**, *42* (2), 339–341. https://doi.org/10.1107/S0021889808042726.

(14) Sheldrick, G. M. SHELXT – Integrated Space-Group and Crystal-Structure Determination. *Acta Cryst A* **2015**, *71* (1), 3–8. https://doi.org/10.1107/S2053273314026370.

(15) Sheldrick, G. M. Crystal Structure Refinement with SHELXL. *Acta Cryst C* **2015**, *71* (1), 3–8. https://doi.org/10.1107/S2053229614024218.

(16) Spek, A. L. Structure Validation in Chemical Crystallography. *Acta Cryst D* **2009**, *65* (2), 148–155. https://doi.org/10.1107/S090744490804362X.

(17) Goodsell, D. S.; Olson, A. J. Automated Docking of Substrates to Proteins by Simulated Annealing. *Proteins: Structure, Function, and Bioinformatics* **1990**, *8* (3), 195–202. https://doi.org/10.1002/prot.340080302.
(18) Morris, G. M.; Goodsell, D. S.; Halliday, R. S.; Huey, R.; Hart, W. E.; Belew, R. K.; Olson, A. J. Automated Docking Using a Lamarckian Genetic Algorithm and an Empirical Binding Free Energy Function. *Journal of Computational Chemistry* **1998**, *19* (14), 1639–1662. https://doi.org/10.1002/(SICI)1096-987X(19981115)19:14%3C1639::AID-JCC10%3E3.0.CO;2-B.
(19) Morris, G. M.; Huey, R.; Lindstrom, W.; Sanner, M. F.; Belew, R. K.; Goodsell, D. S.; Olson, A. J. AutoDock4 and AutoDockTools4: Automated Docking with Selective Receptor Flexibility. *Journal of Computational Chemistry* **2009**, *30* (16), 2785–2791. https://doi.org/10.1002/jcc.21256.
(20) Forli, S.; Huey, R.; Pique, M. E.; Sanner, M. F.; Goodsell, D. S.; Olson, A. J. Computational Protein–Ligand Docking and Virtual Drug Screening with the AutoDock Suite. *Nat Protoc* **2016**, *11* (5), 905–919. https://doi.org/10.1038/nprot.2016.051.
(21) Paliwoda, D.; Kowalska, K.; Hanfland, M.; Katrusiak, A. U-Turn Compression to a New Isostructural Ferrocene Phase. *J. Phys. Chem. Lett.* **2013**, *4* (23), 4032–4037. https://doi.org/10.1021/jz402254b.
(22) Surov, A. O.; Vasilev, N. A.; Vener, M. V.; Parashchuk, O. D.; Churakov, A. V.; Magdysyuk, O. V.; Perlovich, G. L. Pharmaceutical Salts of Fenbendazole with Organic Counterions: Structural Analysis and Solubility Performance. *Crystal Growth & Design* **2021**, *21* (8), 4516–4530. https://doi.org/10.1021/acs.cgd.1c00413.
(23) Kaethner, M.; Preza, M.; Kaempfer, T.; Zumstein, P.; Tamponi, C.; Varcasia, A.; Hemphill, A.; Brehm, K.; Lundström-Stadelmann, B. Establishment and Application of Unbiased in Vitro Drug Screening Assays for the Identification of Compounds against Echinococcus Granulosus Sensu Stricto. *PLoS Negl Trop Dis* **2023**, *17* (8), e0011343. https://doi.org/10.1371/journal.pntd.0011343.
(24) Stadelmann, B.; Scholl, S.; Müller, J.; Hemphill, A. Application of an in Vitro Drug Screening Assay Based on the Release of Phosphoglucose Isomerase to Determine the Structure–Activity Relationship of Thiazolides against Echinococcus Multilocularis Metacestodes. *J Antimicrob Chemother* **2010**, *65* (3), 512–519. https://doi.org/10.1093/jac/dkp490.
(25) Mármol, C. D.; Scalese, G.; Moreira, R.; Veiga, N.; Machado, I.; Faccio, R.; Lima, A.; A. Peralta, R.; Pérez-Díaz, L.; Gambino, D. Exploring a Series of Multifunctional Mn(i) Tricarbonyls as Prospective Agents against Trypanosomatid Parasites: A Comparative Study with the Re(i) Analogues. *Dalton Transactions* **2025**, *54* (16), 6495–6516. https://doi.org/10.1039/D5DT00241A.
(26) Zhong, S.; Kalamatianou, A.; Scalese, G.; O'Beirne, C.; Redrado, M.; Blacque, O.; Picot, C.; Tomasoni, C.; Logé, C.; Albassier, M.; Ourliac-Garnier, I.; Pagniez, F.; Le Pape, P.; Pérez-Díaz, L.; Gambino, D.; Comini, M. A.; Cariou, K.; Gasser, G. Synthesis and Biological Evaluation of Itraconazole Derivatives: Design in an Old Scaffold. *Inorg. Chem.* **2025**, *64* (31), 16192–16203. https://doi.org/10.1021/acs.inorgchem.5c02730.
(27) Rivas, F.; Del Mármol, C.; Scalese, G.; Pérez-Díaz, L.; Machado, I.; Blacque, O.; Medeiros, A.; Comini, M.; Gambino, D. New Multifunctional Ru(II) Organometallic Compounds Show Activity against *Trypanosoma Brucei* and *Leishmania Infantum*. *Journal of Inorganic Biochemistry* **2022**, *237*, 112016. https://doi.org/10.1016/j.jinorgbio.2022.112016.
(28) Del Carpio, E.; Hernández, L.; Lubes, V.; Jourdan, F.; Cerecetto, H.; Scalese, G.; Gambino, D.; Romero, A. H. Current Developments of Metal- and Metalloid-Based Quinoline Compounds as Leishmanicidal Agents. *Front. Chem.* **2025**, *13*. https://doi.org/10.3389/fchem.2025.1586044.
(29) Scalese, G.; Pérez, N.; Pereyra, J.; Sanabria, Y.; Blacque, O.; Machado, I.; Pérez-Díaz, L.; Gambino, D. Broadening the Chemical Diversity of Oxidovanadium(V) Complexes for Targeting Neglected

Tropical Diseases. *Journal of Inorganic Biochemistry* **2025**, *269*, 112891. https://doi.org/10.1016/j.jinorgbio.2025.112891.
